# Changes in the global, regional, and national burdens of NAFLD from 1990 to 2019: A systematic analysis of the global burden of disease study 2019

**DOI:** 10.3389/fnut.2022.1047129

**Published:** 2022-12-21

**Authors:** Dan Wang, Yanbing Xu, Zizhao Zhu, Yanliang Li, Xiaowen Li, Yike Li, Hui Shen, Wei Wu, Yazhuo Liu, Cheng Han

**Affiliations:** ^1^Department of Clinical Nutrition and Metabolism, Affiliated Zhongshan Hospital of Dalian University, Dalian, China; ^2^Department of General Surgery, The Sixth People’s Hospital of Shenyang, Shenyang, China; ^3^Departments of Ophthalmology and Visual Sciences, University of Illinois at Chicago, Chicago, IL, United States

**Keywords:** non-alcoholic fatty liver disease, GBD study, sociodemographic index, risk factors, economic burden

## Abstract

**Background:**

Understanding the burdens and trends of non-alcoholic fatty liver disease (NAFLD) is necessary for developing effective intervention strategies. In this study, Global Burden of Disease (GBD) 2019 study data were extracted and analyzed to elucidate trends of NAFLD.

**Methods:**

The prevalence, incidence, disability-adjusted life year (DALY), and death rates of NAFLD in geographic populations worldwide from 1990 to 2019 were extracted from the GBD 2019 study data. The global temporal trend of NAFLD from 1990 to 2019 was evaluated using estimated annual percentage changes (EAPCs) and age-standardized rates.

**Results:**

Globally, between 1999 and 2019, the age-standardized prevalence rate of NAFLD increased, with EAPCs of 0.77 [95% CI (0.69, 0.85)], whereas the DALY and Death rates decreased, with EAPCs of –0.82 [95% CI (–0.92, –0.71)], and –0.67 [95% CI (–0.76, –0.58)], respectively. Geographically, the age-standardized prevalence rate showed the most serious upward trend in high-income North America with an EAPC of 0.98 [95% CI (0.95, 1.02)], and the age-standardized incidence rate showed an upward trend in Central Asia with an EAPC of 3.17 [95% CI (2.2, 2.49)]. The most significant upward trend of DALY and death rates appeared in Eastern Europe, with EAPCs of 4.06 [95% CI (3.31, 4.82)], and 3.36 [95% CI (2.77, 3.96)], respectively. At the country level, the age-standardized rates showed an upward trend in Armenia, Belarus, and Republic of Korea. Regarding age groups, the percentage change of prevalence was the highest in the 40 to 44 group [0.29 (0.26, 0.34)] from 1990 to 2019; the percentage change of incidence was the highest in the 85 to 89 group [0.46 (0.12, 0.71)] from 1990 to 2019; the percentage change of DALY was the highest in the 80 to 84 group [0.25 (0.11, 0.39)] from 1990 to 2019; and the percentage change of death rate was the highest in the 15 to 19 group [0.36 (0.17, 0.60)] from 1990 to 2019. The percentage change of prevalence of liver cancer due to NASH was the highest in the group of 85 to 89, whereas those of incidence, DALY, and death were the highest in the group above 95 from 1990 to 2019. Regarding the sociodemographic index (SDI), the highest age-standardized prevalence, incidence, and Death rates of NAFLD occurred in middle-SDI countries, and the highest DALY rates of NAFLD occurred in low-SDI countries.

**Conclusion:**

Global NAFLD burdens have increased since 1990. Our findings provide a reference for policymakers to reduce the burden of NAFLD, especially in middle and low-SDI countries.

## Introduction

In recent years, as the number of patients with obesity and type 2 diabetes have increased worldwide, the epidemiological trends of non-alcoholic fatty liver disease (NAFLD) have changed in both developing and developed countries (1). NAFLD is one of the leading causes of liver disease throughout the world. Patients with NAFLD have a certain probability (at least 20 to 30%) of conversion to non-alcoholic steatohepatitis (NASH), which may lead to cirrhosis and other related complications, including hepatocellular carcinogenesis (2). The difference between NASH and NAFLD is that NASH is a progressive form of NAFLD, characterized by liver steatosis, inflammation, hepatocellular injury, and different degrees of fibrosis, while NAFLD is simply steatosis (3). A study by Huang et al. shows that the prevalence of NASH may increase by 56% between 2016 and 2030 in many countries, such as China, Japan, the United States, and the United Kingdom (4). The number of NASH-related liver deaths is projected to increase by 178% by 2030 according to a recent study (5). The increased prevalence of NASH is associated with an increased risk of cardiovascular disease, and it is also associated with increases in cardiovascular and liver-related mortality (2). It has been estimated that 5% to 10% of NAFLD patients die from cardiovascular diseases. Individuals with NAFLD have been shown to have a two-fold increased risk of cardiovascular diseases (6, 7). In the US, for medicare recipients and female patients older than 54 years, NASH is the first indication of liver transplantation issues (2). Additionally, NAFLD is anticipated to be a significant risk factor for hepatocellular carcinoma. It has been estimated that people with NAFLD have an incidence of hepatocellular carcinoma of 0.44 per 1000 person-years (8). Notably, a total of 830,180 deaths in 2020 were attributable to liver cancer, and liver cancer ranks third among cancer-related deaths (9, 10). Given this situation, we need to determine the prevalence of NAFLD around the globe and calculate the trajectories of the burdens of NAFLD over time, so as to inform policymakers across the globe.

In this study, we for the first time analyzed the global, regional, and national prevalence, incidence, DALYs, and death rates of NAFLD from 1990 to 2019 based on the data obtained from the GBD 2019, intending to provide a comparable and comprehensive analysis of NAFLD burdens in terms of age-standardized rates by age and sociodemographic index (SDI). Our results provide an instrumental guide to raise awareness of NAFLD and may will play an invaluable role in the establishment of updated prevention strategies.

## Materials and methods

### Data sources

The Global Health Data Exchange GBD Results Tool^1^ was used to extract information on the prevalence of NAFLD in 204 nations and territories between 1990 and 2019 (date of data extraction, September 5, 2022). GHDx is the world’s most comprehensive catalog of surveys, censuses, vital statistics, and other health-related data. The GBD 2019 incorporates all current epidemiological data sources, updated standard operating procedures, and a thorough assessment of health losses that take into consideration 369 diseases and injuries and 87 risk factors across 204 nations and territories (11, 12). In other words, the GBD 2019 can be used to display the handled information by standardizing devices, allowing the assessment of each variable of interest, including age, area, or year. The Cause of Death Ensemble model (CODEm), which serves as a versatile modeling program, can generate death estimates for all locations across the time series according to covariate data and geospatial linkages (13). Considering that not all diseases were assessed in some countries, DisMod-MR 2.1, a Bayesian meta-regression tool, was used in the GBD 2019 study as the primary estimation method to ensure the consistency between the incidence, prevalence, and mortality rates for each disease (14). Spatiotemporal Gaussian process regression (ST-GPR) is a collection of regression techniques that uses relationship analysis between different locations and periods to obtain specific metrics of interest, such as death rates or risk factor exposures. Based on GBD 2019 data, SDI represents a composite average of per capita income, education level, and fertility rate (15). The SDI scales from 0 to 1, with 1 denoting the region with the highest per capita income, the highest number of years of schooling, and the lowest fertility rate (15). Based on these SDI scores, countries were categorized into five groups: high, high-middle, middle, low-middle and low levels of development. The risk factors in GBD are organized into four levels, from the broadest (Level 1) to the most specific (Level 4). We assessed the Level 2 risks. Because this study used publicly available data, it did not require ethical approval.

### Definition

Fatty liver refers to abnormal lipid accumulation in the parenchymal cells of the liver (16). NAFLD is defined as liver steatosis of more than 5% unrelated to other causes such as alcohol intake, intake of certain drugs, or other established liver disease (17). NASH is considered the progressive form of NAFLD and is characterized by liver steatosis, inflammation, hepatocellular injury and different degrees of fibrosis (3). Disability-adjusted life year (DALY) is a universal metric that allows researchers to compare very different populations and health conditions across time. DALYs equal the sum of years of life lost (YLLs) and years lived with disability (YLDs) (18).

### Data analysis

Descriptive analyses were performed to describe the NAFLD burden based on age, year, and location. Age-standardized rates were calculated using the GBD study’s world standard population. GBD estimates of the burden of disease were reported using 95% uncertainty intervals (UI), including true values of parameters with 95% probability (19). To evaluate long-term trends, we evaluated EAPCs using linear regression analysis. The ASR (per 100,000 population) was calculated following the direct method by summing up the products of the age-specific rates (*a*_*i*_, where *i*denotes the *i^th^* age class) and the number of persons (or weight) (*w*_*i*_) in the same age subgroup *i*of the chosen reference standard population, and then dividing that by the sum of standard population weights, i.e., $A\,S\,R=\frac{\sum_{i=1}^{A}a_{i}\,w_{i}}{\sum_{i=1}^{A}w_{i}}\times 100,000$. EAPC was used to assess trends in ASR over time. A regression line was fitted to the natural logarithm of the rates *i*.*e*., *y* = α+β*x*+ε, where *y* = *ln*(*ASR*), and *x* = calendar year. The EAPC was calculated as 100 ×(*exp*(β)− 1) and its 95% confidence interval (CI) was obtained from the linear regression model (20). When ASR shows an increasing trend, the value of EAPC and the lower boundary of the 95% CI are both greater than 0. However, when ASR shows a downward trend, the EAPC value and the upper boundary of the 95%CI are lower than 0. In addition, if ASR has a constant trend, the 95% CI of the EAPC contains 0 (21). The associations between the prevalence, incidence, DALY, and death rates of NAFLD and the SDI were examined using a smooth spline model. All statistical analyses were performed using GraphPad Prism (Version 9.3.1) and RStudio Software (Version 4.1.2).

## Results

### Age-standardized rates of NAFLD globally, regionally, and nationally

#### Prevalence

**FIGURE 1:**
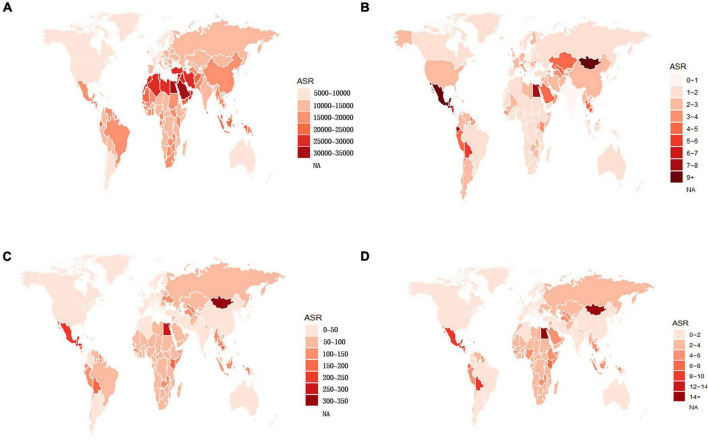
Global age-standardized **(A)** prevalence, **(B)** incidence, **(C)** DALY, and **(D)** death rates of NAFLD in 2019. NAFLD, non-alcoholic fatty liver disease; DALY, disability-adjusted life years.

**FIGURE 2:**
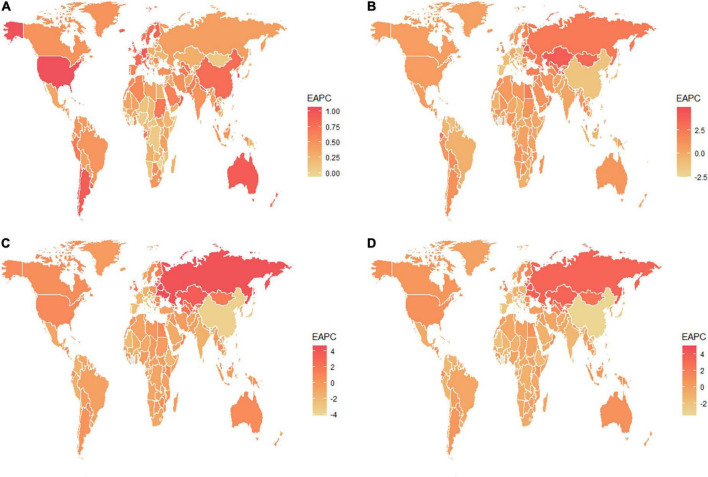
The estimated annual percentage changes of the global age-standardized **(A)** prevalence, **(B)** incidence, **(C)** DALY, and **(D)** death rates from 1990 to 2019. EAPC, estimated annual percentage change; DALY, disability-adjusted life years.

Globally, the age-standardized prevalence rate (ASPR) of NAFLD increased from 12,065 [95% UI (10,779, 13,536)] per 100,000 population in 1990 to 15,023 [95% UI (13,493, 16,764)] per 100,000 population in 2019 (Table 1). During the period from 1990 to 2019, there was an upward trend in global ASPR, with an EAPC of 0.77 [95% CI (0.69, 0.85)] (Table 1). Cirrhosis and other chronic liver diseases caused by NAFLD had ASPR of 0.77 [95% CI (0.69, 0.85)] from 1990 to 2019, respectively (Supporting Supplementary Figure 3 and Table 1). Regionally, based on ASPRs for 21 GBD regions in 2019, the highest rates appeared in North Africa and the Middle East [27,749 (25,410, 30,284)], Southeast Asia [18,299 (16,509, 20,310)], and Southern Sub-Saharan Africa [18,076 (16,348, 19,988)] (Figure 1A and Supporting Supplementary Table 4). Moreover, the ASPR showed significant increasing trends in high-income North America, Australasia, Central Asia, Eastern Europe, Western Europe, and Southern Latin America. Furthermore, the prevalence rate was the highest in middle SDI regions and the lowest in high SDI regions in 2019 (Supporting Supplementary Figure 2 and Supporting Supplementary Table 6). We also observed at the country level that Egypt [34,518 (31,798, 37,254)], Qatar [33,318 (30,578, 36,169)], Kuwait [31,675 (29,116, 34,531)], and The Republic of the United Arab Emirates [30,542 (27,979, 33,329)] had the highest prevalence rates per 100,000 population in 2019 (Figure 1A and Supporting Supplementary Table 3). In addition, The United States of America, Equatorial Guinea, Taiwan, The Republic of Korea, and Germany showed significant upward trends (Figure 2A and Supporting Supplementary Table 2). Regarding age groups, the ASPR was the highest between 75 and 79 years [33,581 (25,536, 42,132)], and the prevalence of cases was the highest between the ages of 45 and 49 years [126979,428 (95628,014, 160296,458)] in 2019 (Supporting Supplementary Table 5). Moreover, the percentage changes were the highest in the 40 to 44 year group [0.29 (0.26, 0.34)] from 1990 to 2019 (Supporting Supplementary Table 7).

**TABLE 1:** Age-standardized prevalence rate (ASPR) for NAFLD, cirrhosis, and liver cancer in 1990 and 2019 for both sexes, and estimated annual percentage changes (EAPCs) by Global Burden of Disease (GBD) region.

NAFLD				NAFLD			NASH		
				Cirrhosis and other chronic liver diseases due to NAFLD			Liver cancer due to NASH		
		
Location	ASPR in 1990	ASPR in 2019	EAPC between 1990 and 2019	ASPR in 1990	ASPR in 2019	EAPC between 1990 and 2019	ASPR in 1990	ASPR in 2019	EAPC between 1990 and 2019
Global	12,065.63 (10,779.51–13,536.97)	15,023.47 (13,493.73–16,764.84)	0.77 (0.69–0.85)*	12,065.15 (10,779.06–13,536.49)	15,022.9 (13,493.19–16,764.24)	0.77 (0.69–0.85)*	0.48 (0.39–0.57)	0.57 (0.47–0.7)	0.13 (−0.1-0.36)
Andean Latin America	11,762.73 (10,558.43–13,129.24)	13,690.02 (12,377.04–15,086.39)	0.5 (0.48–0.53)*	11,762.35 (10,558–13,128.82)	13,689.71 (12,376.75–15,086.12)	0.5 (0.48–0.53)*	0.38 (0.26–0.53)	0.31 (0.22–0.45)	−1.15 (−1.56–0.74)*
Australasia	7,267.3 (6,458.28–8,167.64)	9,444.78 (8,455.94–10,478.72)	0.92 (0.85–1)*	7,267.12 (6,458.1–8,167.5)	9,444.14 (8,455.34–10,478.02)	0.92 (0.85–1)*	0.17 (0.13–0.23)	0.64 (0.45–0.88)	4.87 (4.42–5.33)*
Caribbean	14,537.3 (13,031.15–16,203.49)	16,169.79 (14,591.34–17,866.81)	0.4 (0.38–0.42)*	14,536.81 (13,030.79–16,202.96)	16,169.48 (14,591.11–17,866.53)	0.4 (0.38–0.42)*	0.49 (0.35–0.67)	0.31 (0.21–0.44)	−1.47 (−2.27–0.66)*
Central Asia	12,301.68 (11,056.51–13,710.28)	14,150.99 (12,737.69–15,748.83)	0.5 (0.46–0.53)*	12,301.51 (11,056.35–13,710.1)	14,150.39 (12,737.16–15,748.23)	0.5 (0.46–0.53)*	0.17 (0.12–0.24)	0.6 (0.43–0.85)	3.97 (3.46–4.48)*
Central Europe	10,680.15 (9,633.41–11,859.76)	11,895.12 (10,755.07–13,109.79)	0.34 (0.33–0.35)*	10,679.78 (9,633.05–11,859.42)	11,894.85 (10,754.82–13,109.51)	0.34 (0.33–0.35)*	0.37 (0.29–0.46)	0.27 (0.2–0.37)	−0.74 (−1.13-−0.34)*
Central Latin America	14,593.3 (13,114–16,254.57)	16,618.31 (14,959.82–18,388.89)	0.43 (0.42–0.44)*	14,593.05 (13,113.76–16,254.24)	16,617.99 (14,959.55–18,388.64)	0.43 (0.42–0.44)*	0.25 (0.19–0.33)	0.32 (0.25–0.42)	1.11 (0.81–1.41)*
Central Sub-Saharan Africa	12,574.1 (11,111.2–14,247.91)	13,331.37 (11,795.61–15,097.48)	0.16 (0.15–0.18)*	12,573.94 (11,111.07–14,247.76)	13,331.21 (11,795.46–15,097.36)	0.16 (0.15–0.18)*	0.16 (0.11–0.23)	0.16 (0.11–0.24)	−0.13 (−0.2-−0.05)*
East Asia	12,538.49 (11,109.64–14,232.15)	15,681.5 (14,023.07–17,552.75)	0.81 (0.53–1.08)*	12,537.44 (11,108.5–14,230.81)	15,680.86 (14,022.56–17,552.19)	0.81 (0.53–1.08)*	1.04 (0.82–1.28)	0.65 (0.51–0.8)	−3.15 (−3.94−2.36)*
Eastern Europe	11,026.17 (9,932.2–12,225.31)	12,295.31 (11,098.7–13,601.99)	0.38 (0.37–0.4)*	11,026.06 (9,932.09–12,225.2)	12,295.07 (11,098.49–13,601.74)	0.38 (0.37–0.4)*	0.11 (0.09–0.12)	0.24 (0.19–0.28)	3.3 (3.05–3.55)*
Eastern Sub-Saharan Africa	12,556.46 (11,140.38–14,168.85)	13,709.43 (12,222.77–15,370.64)	0.29 (0.28–0.3)*	12,556.15 (11,140.04–14,168.49)	13,709.06 (12,222.3–15,370.26)	0.29 (0.28–0.3)*	0.31 (0.23–0.42)	0.37 (0.27–0.5)	0.46 (0.36–0.55)*
High-income Asia Pacific	6,835.48 (6,080.84–7,694.95)	7,673.15 (6,831.48–8,630.74)	0.56 (0.45–0.67)*	6,834.65 (6,080.03–7,694.13)	7,671.66 (6,829.94–8,629.15)	0.56 (0.45–0.67)*	0.83 (0.69–0.97)	1.49 (1.15–1.94)	1.66 (1.14–2.18)*
High-income North America	7,263.2 (6,424.71–8,251.36)	9,396.67 (8,404.82–10,543.35)	0.98 (0.95–1.02)*	7,262.95 (6,424.45–8,251.1)	9,395.9 (8,403.86–10,542.43)	0.98 (0.95–1.02)*	0.25 (0.22–0.29)	0.77 (0.61–0.96)	4.1 (3.77–4.43)*
North Africa and Middle East	24,422.08 (22,219.39–26,866.87)	27,749.31 (25,410.63–30,284.09)	0.47 (0.46–0.49)*	2,4421.59 (22,218.89–26,866.5)	27,748.49 (25,409.87–30,283.48)	0.47 (0.46–0.49)*	0.49 (0.35–0.69)	0.82 (0.59–1.16)	2.06 (1.85–2.27)*
Oceania	15,748.11 (14,076.86–17,647.44)	16,869.48 (15,133.71–18,727.13)	0.18 (0.13–0.23)*	15,747.83 (14,076.6–17,647.18)	16,869.18 (15,133.44–18,726.79)	0.18 (0.13–0.23)*	0.28 (0.19–0.4)	0.29 (0.2–0.42)	0.28 (0.19–0.36)*
South Asia	12,250.88 (10,869.31–13,935.44)	14,514.16 (12,969.48–16,400.89)	0.54 (0.43–0.64)*	12,250.64 (10,869.08–13,935.19)	14,513.87 (12,969.2–16,400.6)	0.54 (0.43–0.64)*	0.25 (0.2–0.31)	0.29 (0.23–0.36)	0.44 (0.37–0.51)*
Southeast Asia	16,105.97 (14,459.64–18,017.03)	18,299.95 (16,509.31–20,310.25)	0.45 (0.44–0.47)*	16,105.48 (14,458.92–18,016.63)	18,299.21 (16,508.63–20,309.58)	0.45 (0.44–0.47)*	0.48 (0.37–0.64)	0.73 (0.52–1.01)	1.52 (1.44–1.6)*
Southern Latin America	6,617.38 (5,889.06–7,492.52)	8,602.74 (7,719.55–9,564.43)	0.92 (0.82–1.01)*	6,617.27 (5,888.96–7,492.41)	8,602.52 (7,719.35–9,564.23)	0.92 (0.82–1.01)*	0.11 (0.08–0.17)	0.23 (0.15–0.34)	3.11 (2.9–3.33)*
Southern Sub-Saharan Africa	16,003.49 (14,363.86–17,873.1)	18,076.66 (16,348.67–19,998.37)	0.41 (0.4–0.42)*	16,002.88 (14,363.35–17,872.58)	18,075.91 (16,347.92–19,997.66)	0.41 (0.4–0.42)*	0.62 (0.41–1.01)	0.75 (0.61–0.92)	0.18 (–0.32–0.69)
Tropical Latin America	13,164.99 (11,825.59–14,662.83)	15,241.34 (13,723.31–16,819.07)	0.49 (0.48–0.5)*	13,164.88 (11,825.48–14,662.71)	15,241.19 (13,723.17–16,818.9)	0.49 (0.48–0.5)*	0.11 (0.09–0.13)	0.15 (0.13–0.18)	1.64 (1.43–1.84)*
Western Europe	7,881.02 (7,036.73–8,876.98)	9,933.37 (8,932.6–11,044.68)	0.81 (0.74–0.89)*	7,880.8 (7,036.42–8,876.77)	9,932.86 (8,931.95–11,044.15)	0.81 (0.74–0.89)*	0.22 (0.17–0.28)	0.52 (0.39–0.69)	3.12 (2.82–3.43)*
Western Sub-Saharan Africa	13,163.5 (11,718.59–14,781.21)	14,284.25 (12,757.25–15,953.21)	0.22 (0.18–0.26)*	13,163.1 (11,718.18–14,780.8)	14,283.82 (12,756.86–15,952.84)	0.22 (0.18–0.26)*	0.4 (0.29–0.53)	0.43 (0.32–0.58)	0.15 (0.08–0.21)*

*Indicates a *P*-value less than 0.05.

#### Incidence

Globally, compared to 1990, the incidence rate of NAFLD increased from 1.94 [95% UI (1.38, 2.77)] per 100,000 population to 2.08 [95% UI (1.52, 2.93)] per 100,000 population in 2019 (Supporting Supplementary Table 1). From 1990 to 2019, there was an increase in the global age-standardized incidence rate (ASIR), with an EAPC of 0.1 [95% CI (–0.03, 0.23)] (Supporting Supplementary Table 2). Among 21 GBD regions, Central Latin America [6.88 (4.70, 9.98)] and Andean Latin America [5.62 (3.86, 7.97)] had the highest ASIRs per 100,000 population in 2019 (Figure 1B and Supporting Supplementary Table 4). In addition, the ASIR showed significant increasing trends in Central Asia, Eastern Europe, Andean Latin America, North Africa and Middle East, and Australasia. As shown in Supporting Supplementary Figure 2 and Supporting Supplementary Table 6, middle SDI regions had the highest incidence rate in 2019, and low SDI regions had the lowest incidence rate. Among countries, Mongolia [12.65 (9.33, 17.00)], Mexico [9.53 (6.29-14.04)], Guatemala [9.06 (6.10, 13.19)], and Egypt [7.63 (5.26, 11.06)] reported the highest incidence rates per 100,000 population in 2019 (Figure 1B and Supporting Supplementary Table 3). In addition, Armenia, Kazakhstan, Belarus, Tajikistan, Mongolia, and Lithuania (EAPC > 3) displayed significant upward trends (Figure 2B and Supporting Supplementary Table 2). Regarding age groups, the ASIR was the highest in the group above 95 years [5.86 (2.52, 14.61)], and the incidence rate was the highest in the age between 45 and 49 years [27464 (11,404, 53,657)] in 2019 (Supporting Supplementary Table 5). Moreover, the percentage changes were the highest in the 85 to 89 year group [0.46 (0.12, 0.71)] from 1990 to 2019 (Supporting Supplementary Table 7).

#### DALY

**FIGURE 3:**
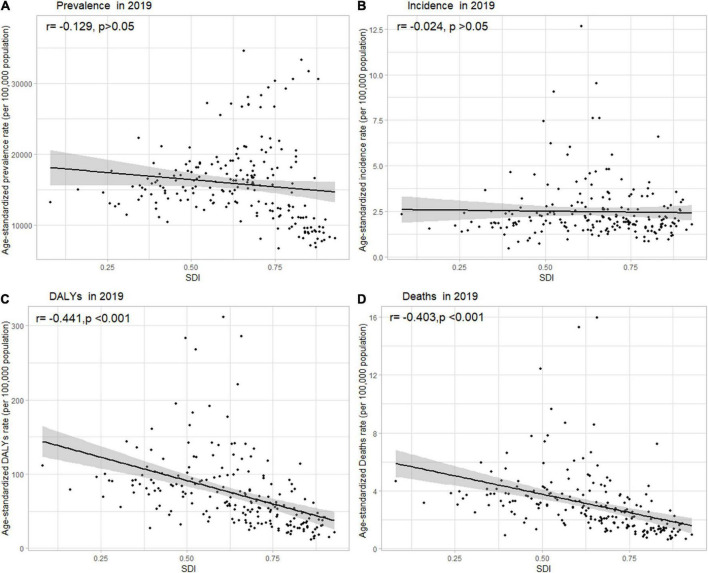
Age-standardized **(A)** prevalence, **(B)** incidence, **(C)** DALY, and **(D)** death rates of NAFLD in 204 countries and territories by SDI, 2019. Note: The gray circles represent countries that were available in SDI data; DALY, disability-adjusted life years; NAFLD, non-alcoholic fatty liver disease; SDI, sociodemographic index.

Globally, the age-standardized DALY rates were found to be declining for NAFLD, from 63.27 (95% UI [48.58, 93.52]) per 100,000 population in 1990 down to 53.33 (95% UI [40.73, 68.29]) per 100,000 population in 2019 (Supporting Supplementary Table 1). Global DALY rates decreased from 1990 to 2019, with an EAPC of –0.82 (95% CI [–0.92, –0.71]) (Supporting Supplementary Table 2). Regionally, based on age-standardized DALY rates for 21 GBD regions, Central Latin America [161 (119, 212)], Andean Latin America [127 (86, 179)], and Central Asia [112 (82, 151)] had the highest rates per 100,000 population in 2019 (Figure 1C and Supporting Supplementary Table 4). We found that only Eastern Europe, Central Asia, high-income North America, and Australasia showed upward trends, while other regions had downward trends. As shown in Supporting Figure 2 and Supporting Supplementary Table 6, low SDI countries had the highest DALY rates, while high SDI countries had the lowest DALY rates. At the country level, the highest DALY rates per 100,000 population were observed in Mongolia [311 (218, 218)], Egypt [286 (183, 425)], Honduras [283 (175, 421)], and Guatemala [268 (179, 389)] in 2019 (Figure 1C and Supporting Supplementary Table 3). In addition, Belarus, Ukraine, Russian Federation and Lithuania showed increasing trends, while Hungary, China, and Italy all had downward trends (Figure 2C and Supporting Supplementary Table 2). In Figure 3, we illustrate the relationship between age-standardized rates at the country level and SDI scores. As the SDI index value increased, the DALY rates decreased linearly (*r* = –0.441, *p* < 0.001). Regarding age groups, the ASPR was the highest between 75 and 79 years [33,581 (25,536, 42,132)], and the prevalence of cases was the highest between the ages of 45 and 49 years [126979,428 (95628,014, 160296,458)] in 2019 (Supporting Supplementary Table 5). Moreover, the percentage changes were the highest in the 40 to 44 year group [0.29 (0.26, 0.34)] from 1990 to 2019 (Supporting Supplementary Table 7).

#### Death

Globally, age-standardized deaths rates were found to be declining for NAFLD, from 2.39 [95% UI (1.84, 3.05)] in 1990 to 2.09 [95% UI (1.61, 2.60)] in 2019 (Supporting Supplementary Table 1). Global death rates decreased from 1990 to 2019, with an EAPC of –0.67 [95% CI (–0.76, –0.58)] (Supporting Supplementary Table 2). Geographically, in terms of the age-standardized death rate for 21 BGD regions, Central Latin America [6.24 (4.65, 8.02)], Andean Latin America [5.68 (3.93, 7.71)], and Eastern Sub-Saharan Africa [4.40 (3.14, 5.94)] reached the highest value per 100,000 population in 2019 (Figure 1D and Supporting Supplementary Table 4). Moreover, the ASPR showed significant upward trends in Eastern Europe, Central Asia, High-income North America, Australasia, and Southern Latin America. At the country level, Egypt had the highest deaths rates per 100,000 population [15.98 (10.07, 24.33)] in 2019, followed by Mongolia [15.31 (10.92, 20.80)] and Honduras [12.43 (7.92, 18.24)] (Figure 1D and Supporting Supplementary Table 3). In addition, Armenia, Belarus, and Lithuania showed noteworthy increasing trends (Figure 2D and Supporting Supplementary Table 2). As depicted in Supporting Figure 2 and Supporting Supplementary Table 6, middle-SDI countries had the highest death rates, while high-SDI countries had the lowest death rates. In Figure 3, we illustrate the relationship between the age-standardized rates at the country level and SDI scores. As the SDI index values increased, the Death rates decreased linearly (*r* = –0.403, *p* < 0.001). Regarding age groups, the death rate was the highest in the group above 95 years [37.82 (18.98, 62.56)], and the death cases were the highest among patients between 65 and 69 years of age [20394 (139,60, 28,942)] in 2019 (Supporting Supplementary Table 5). In addition, the percentage changes were the highest in the 15 to 19 year group [0.36 (0.17, 0.60)] from 1990 to 2019 (Supporting Supplementary Table 7).

#### Behavioral and metabolic risk factors for NAFLD from GBD

**FIGURE 4:**
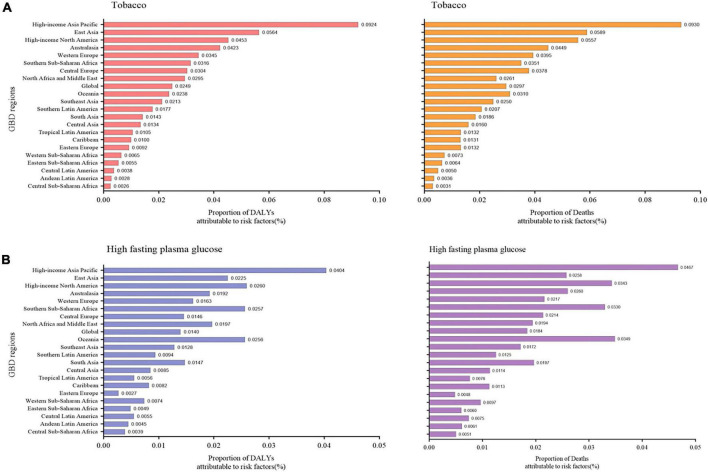
Proportion of NAFLD DALY and deaths attributable to **(A)** tobacco and **(B)** high fasting plasma glucose for 21 GBD regions, 2019. NAFLD, non-alcoholic fatty liver disease; DALY, disability-adjusted life years.

The significant increase in the prevalence of NAFLD has prompted researchers to explore risk factors for this disease. DALY percent and death percent are presented as a result of NAFLD. The geographic area most seriously affected by both smoking and high-fasting plasma glucose in 2019 is the high-income Asia Pacific region. For tobacco, the high-income Asia Pacific region showed the highest DALY percent [0.092 (0.052, 0.135)] in 2019, followed by East Asia [0.056 (0.029, 0.086)], and high-income North America [0.045 (0.023, 0.072)], which also displayed the highest death percent (Figure 4A). Regarding high-fasting plasma glucose, the highest DALY percent in 2019 was found in the high-income Asia Pacific region [0.040 (0.009, 0.0900)], high-income North America [0.026 (0.006, 0.058)], and Southern Sub-Saharan Africa [0.026 (0.006, 0.057)] (Figure 4B). In addition, the death percent was the highest in the high-income Asia Pacific region (0.047 [0.011, 0.102]), Oceania [0.035 (0.009, 0.079)], and high-income North America [0.034 (0.008, 0.075)] (Figure 4B).

## Discussion

Updated estimates of NAFLD prevalence, incidence, DALY, and death rates in 204 countries have been established by this analysis. This study also provides a comprehensive description of the burden of NAFLD, allowing direct comparisons across time and location. According to our study, the ASIR has increased over time at the same rate as ASPR, and DALY and death rates displayed downward trends in many regions. Recent increases in diabetes and obesity may be responsible for this phenomenon, as 90% of people with obesity and 50% of people with diabetes also have NAFLD (9). Since obesity is a major risk factor for NAFLD, the prevalence of NAFLD will rise in tandem with the global obesity epidemic, especially in China, where obesity is estimated to increase by 0.32% per year (22). Type 2 diabetes currently affects 69 million adults between the ages of 20 and 79. It is projected that this number will reach 123 million by 2040, which would mean a greater burden on those suffering from NAFLD (23). Concerning gender, males had a higher ASPR than females. The prevalence rates were the highest between the ages of 70 and 75 years. Moreover, we found that the number of female NAFLD patients aged 70 years or older was higher than the number of male NAFLD patients aged 70 years or older. There was a link between this finding and low estrogen levels after menopause. It has been suggested that estrogen may prevent NAFLD, and low estrogen levels may increase the risk of insulin resistance, cardiovascular disease, and the development of NAFLD (24). In addition to gender differences, we found that there were regional differences in NAFLD.

This study found that the burden of NAFLD varied greatly among regions and was significantly correlated with economic and social development. Among countries with higher SDI, the NAFLD burden decreased. Public health crises are likely to worsen in the future as the impact of NAFLD increases. Consistent with previous studies, NAFLD has been shown to result in a remarkable burden in Southeast Asia, North Africa, and the Middle East, as well as Southern Sub-Saharan Africa (25). Moreover, Central Asia, Central Latin America, and Andean Latin America also showed heavy burdens with regard to incidence and DALY rates. It is important to note that both Central and Southeast Asia faced a severe situation. The socioeconomic and dietary factors affecting the burden of NAFLD can differ considerably across Asia’s large and heterogeneous countries. We found that, in high-income Asia-Pacific countries, such as South Korea, Japan, and Singapore, there was a slight rise of prevalence rate and a decreasing trend of DALY rate for NAFLD between 1990 and 2019. The previously described link between obesity and NAFLD might explain this phenomenon (25). Japan has a lower obesity rate than other Asian countries (18). As body mass index (BMI), triglyceride, and LDL cholesterol levels increase linearly over time, the incidence of NAFLD has increased linearly as well. This suggests that many patients (BMI < 23 kg/m^2^) have very lean forms of this disease. In Asia, the prevalence of NAFLD is currently about 25%, with 8% to 19% of people considered to have lean NAFLD (26). Previous studies have reported conflicting findings on the severity of lean NAFLD; namely, some studies have indicated that compared with NAFLD in obese individuals, lean NAFLD is usually milder (27), and some studies have suggested that lean NAFLD patients are more likely to die and suffer from disability (28). Lean patients with NAFLD had a lower prevalence of metabolic syndrome (2%–48%) than overweight or obese patients (22%–64%) (29). It has been estimated that 7% of Americans and 25% to 30% of rural Asians have lean NAFLD (30, 31). Along with the association between NAFLD and BMI, there is also evidence of familial clustering of this disease. Familial clustering may be associated with up to 27% of cases of NAFLD (32, 33). Lean Asian NAFLD patients without metabolic syndrome are more likely to carry the *PNPLA3* rs738409 GG allele, which can account for the similar prevalence of NAFLD between Asians and Caucasians (29, 34, 35). There is also an association between the presence of the rs738409 G allele in the liver in adults and severe steatosis, NASH, and liver fibrosis (32, 36). Furthermore, we found that the High-income Asia Pacific region is the region most affected by tobacco and high-fasting blood glucose levels. This may be because half of the world’s tobacco is grown and consumed in Asia, and the number of smokers there is large (37). In the Asia-Pacific region, changing lifestyle is a top priority for treatment in many patients. However, there is still a need for further research on NAFLD in Asia, since genetic factors, lifestyles, and economic conditions differ widely across this region.

Notably, although the prevalence of NAFLD showed a slight increase, the incidence and DALY rates of NAFLD in China have declined overall. This could be because in 2009 China launched an ambitious healthcare reform initiative, and by 2015, 95% of its citizens were insured (23). This has made detecting and treating NAFLD at an early stage more likely. The high and increasing prevalence of NAFLD in Sub-Saharan Africa also came to our notice. Currently, Sub-Saharan Africa, a middle-to-lower income region, is experiencing economic growth and increasing urbanization (17). Moreover, Sub-Saharan Africa is one of many food-insecure regions, the overuse of cheap, low nutritional-value, high-calorie foods can lead to obesity and metabolic syndrome in patients in this region (17). The prevalence of NAFLD may also be affected by HIV infection, its therapies, and its metabolic consequences. Southern Sub-Saharan Africa has the highest HIV prevalence in the world (38). Unfortunately, NAFLD was not addressed in any written national strategy from any of the 29 European countries reviewed in a study published in 2019 (39). There is low awareness of NAFLD among the general public, high-risk groups, and non-liver specialists (39). NAFLD also imposes a huge economic burden. The economic burden of NAFLD was described in one study using a steady-state prevalence model (40). Approximately $62 billion ($1,584 per patient) of direct medical costs are expected in the US using this model, with more than 39 million Americans suffering from NAFLD at the time of publication. It is estimated that 30 million people in Germany, France, Italy, and the United Kingdom will suffer from NAFLD over the next decade, with an estimated annual cost of approximately €19 billion (ranging between €345 and €1,115 per patient) (40). The economic burden of NAFLD complication management has been estimated at $908 billion over ten years because of comorbidities such as cardiovascular disease, diabetes, hypertension, and hyperlipidemia (41). In addition to causing considerable economic burden, NAFLD also negatively impacts the health-related quality of life of patients. Efforts have been made to develop quality-of-life tools, such as the Chronic Liver Disease Questionnaire (CLDQ) NASH because cases of NAFLD and NASH are on the rise, as well as tools for measuring specific areas of the abdomen, symptoms, activity, mood, fatigue, and overall health (42, 43). Thus, NAFLD awareness should be increased among the general public and high-risk groups to assess the true prevalence of NAFLD and guide public health policies.

There is an urgent need for safe, effective, and widely applicable treatments for NAFLD and its severe complications. Unfortunately, drug therapies for NAFLD have limited effectiveness. The cornerstone of these therapies is changing lifestyle habits, and losing weight is the only proven treatment for NAFLD. Hepatic steatosis is improved by weight loss of 3–5%, but hepatitis can be reduced by weight loss of 5–10% (9). With even relatively small percentages of weight loss, significant reductions in liver fat percentage and improvements in insulin sensitivity, cardiometabolic risk factors, and long-term health have been shown to occur (44). There are several limitations worth noting in our study. First, the data from GBD are not original, but were collected from other institutions, such as cancer registries (19). An integrative meta-regression approach was adopted in GBD estimates that could comprehensively incorporate all dimensions of health data to remedy this limitation, accounting for spatial heterogeneity and heterogeneity in data sources and biases (19). Moreover, for some regions that lack the data sources, such as those in Sub-Saharan Africa, GBD estimates heavily rely on modeling processes, predicted covariates, and trends from the past or from neighboring countries, which results in some uncertainty (45). In addition, only data on two risk factors could be obtained from GBD 2019, which caused the insufficient comprehensiveness of our study. Finally, data on gender groups were not available, so we were not able to describe gender groups in detail.

In summary, the dramatic increase in the global prevalence of NAFLD indicates that it has become a new public health problem.

## Data availability statement

The original contributions presented in this study are included in the article/Supplementary material, further inquiries can be directed to the corresponding authors.

## Author contributions

DW, YX, and ZZ: conceptualization, supervision, methodology, data analysis, writing—original draft, writing—review and editing, and final approval. YaL, XL, YiL, HS, and WW: methodology, data analysis, writing—original draft, writing—review and editing, and final approval. YLu and CH: data analysis, writing—original draft, writing—review and editing, and final approval. All authors have read and agreed to the published version of the manuscript.

## Footnotes

1
http://ghdx.healthdata.org/gbd-results-tool
