# Supplementary material for: Changes in the global, regional, and national burdens of NAFLD from 1990 to 2019: A systematic analysis of the global burden of disease study 2019
Source: Front Nutr. 2022 Dec 21;9:1047129. doi: 10.3389/fnut.2022.1047129 (PMC9811393; doi:10.3389/fnut.2022.1047129)
Supplement: Supplementary file 1 [file Data_Sheet_1.docx]

**SUPPLEMENT**

**Supplementary Figure 1.** Global age-standardized prevalence, incidence, DALYs, and Death rate of NAFLD in both sexes from 1990 to 2019.

**Supplementary Figure 2.** Age-standardized prevalence, incidence, DALYs, and Death rate by SDI quintile for NAFLD from 1990 to 2019.

**Supplementary Figure 3.** The estimated annual percentage changes (EAPCs) of the age-standardized prevalence rate worldwide of cirrhosis (Cirrhosis and other chronic liver diseases due to NAFLD) and liver cancer (Liver cancer due to NASH) from 1990 to 2019.

**Supplementary Figure 4.** Global age-specific prevalence, incidence, DALYs and Death rate of NAFLD in 2019.

**Supplementary Figure 5.** Age-standardized prevalence, incidence, DALYs, and Death rate of NAFLD globally and in 21 GBD regions by SDI, 1990-2019.

**Supplementary Table 1.** Global age-standardized prevalence, incidence, DALYs, and Death rate of NAFLD in both sexes from 1990 to 2019.

**Supplementary Table 2.** The estimated annual percentage changes (EAPCs) of age-standardized prevalence, incidence, DALYs, and Death rate of NAFLD worldwide from 1990 to 2019.

**Supplementary Table 3.** Age-standardized prevalence, incidence, DALYs, and Death rate of NAFLD for 204 countries and territories in 2019.

**Supplementary Table 4.** Age-standardized prevalence, incidence, DALYs, and Death rate of NAFLD globally and for 21 GBD regions, 1990-2019.

**Supplementary Table 5.** Global age-specific prevalence, incidence, DALYs, and Death rate of NAFLD in 2019.

**Supplementary Table 6.** Age-standardized prevalence, incidence, DALYs, and Death rate of NAFLD grouped by SDI quintiles from 1990 to 2019.

**Supplementary Table 7.** Global age-specific prevalence, incidence, DALYs, and Death percentage of annual rate of change from 1990 to 2019.

**Supplementary Figure 1.** Global age-standardized prevalence, incidence, DALYs, and Death rate of NAFLD in males and females from 1990 to 2019.


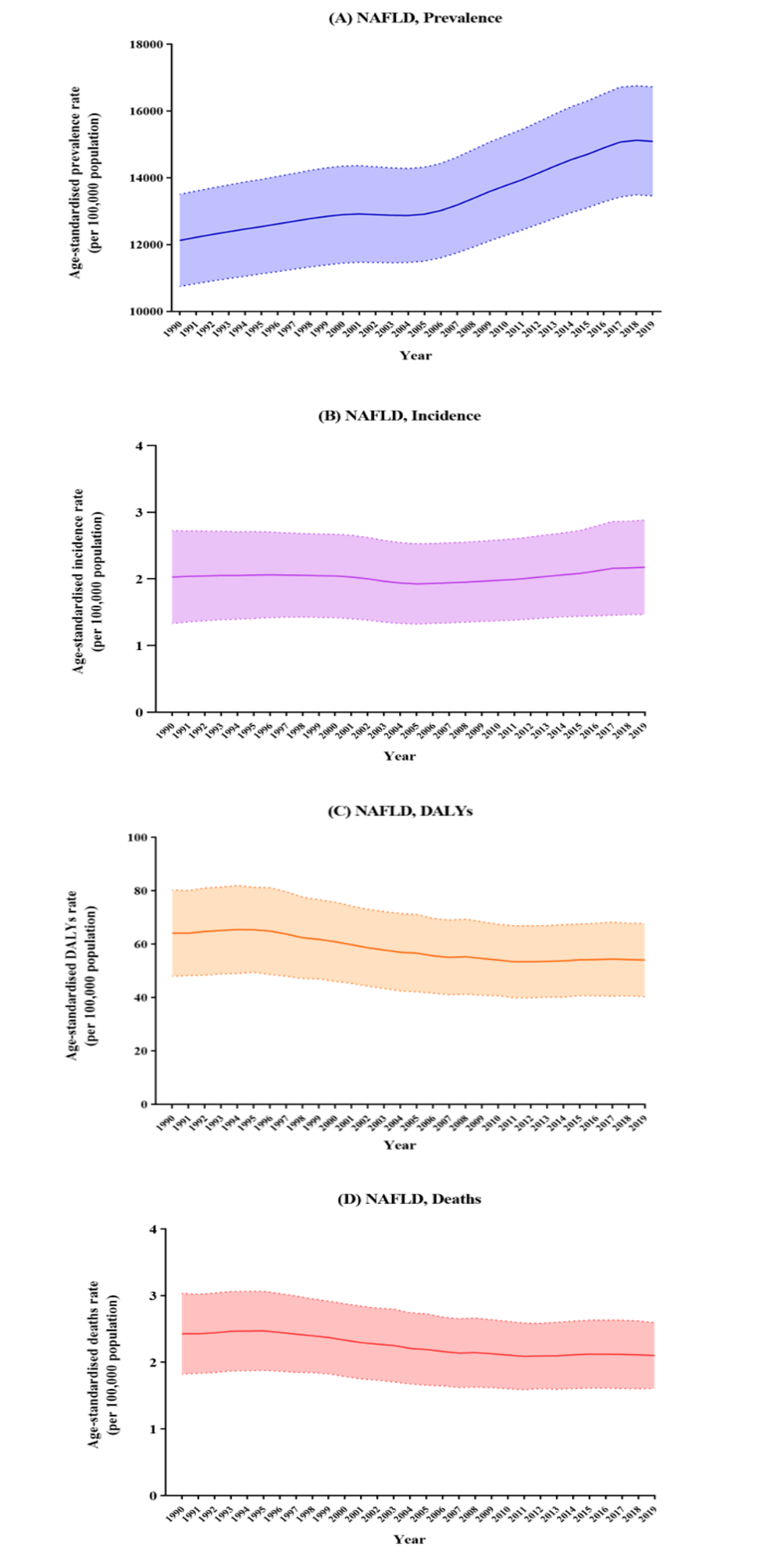


**Supplementary Figure 2.** Age-standardized prevalence, incidence, DALYs, and Death rate by SDI quintile for NAFLD from 1990 to 2019.


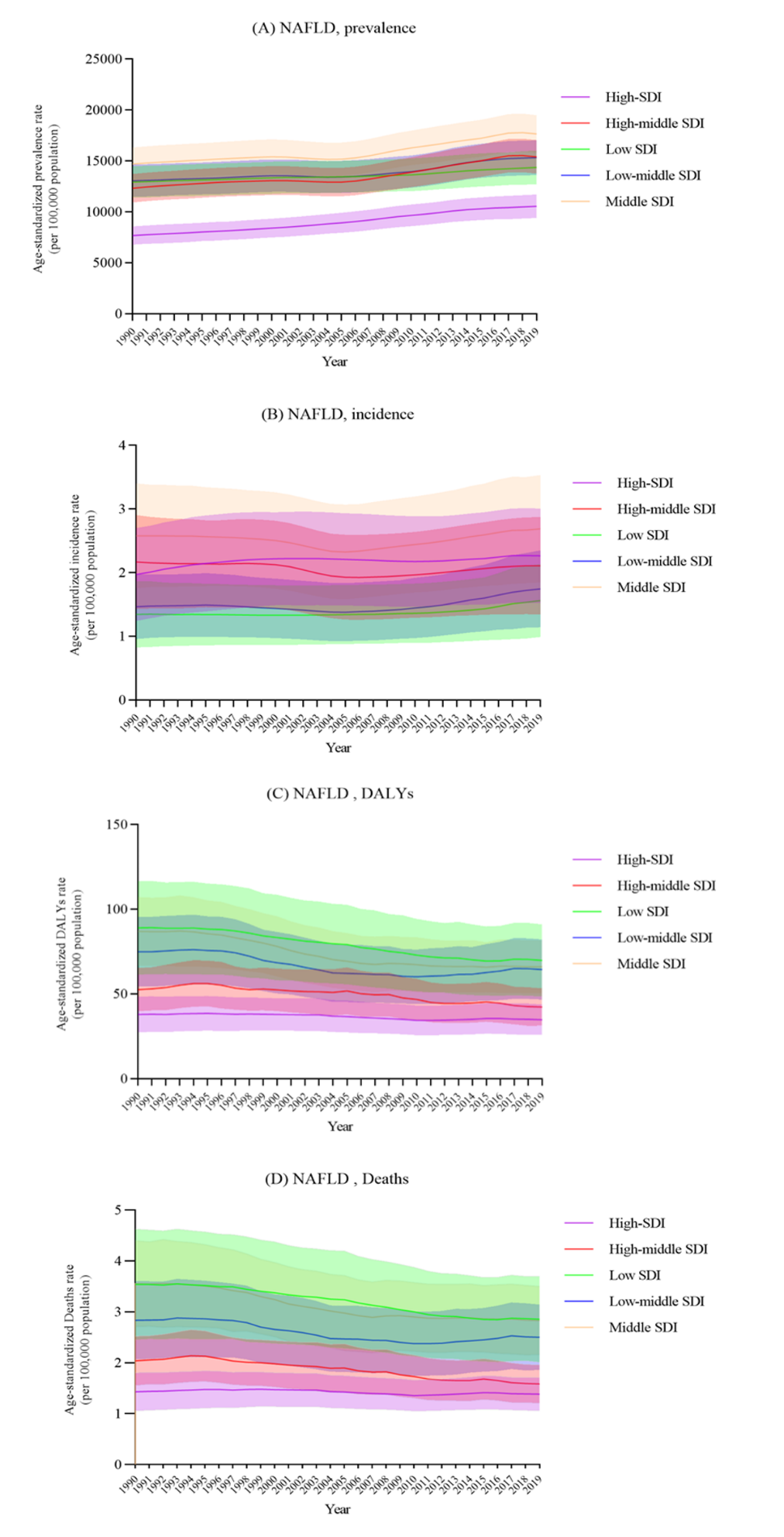


**Supplementary Figure 3.** The estimated annual percentage changes (EAPCs) of the age-standardized prevalence rate worldwide of cirrhosis and liver cancer from 1990 to 2019.


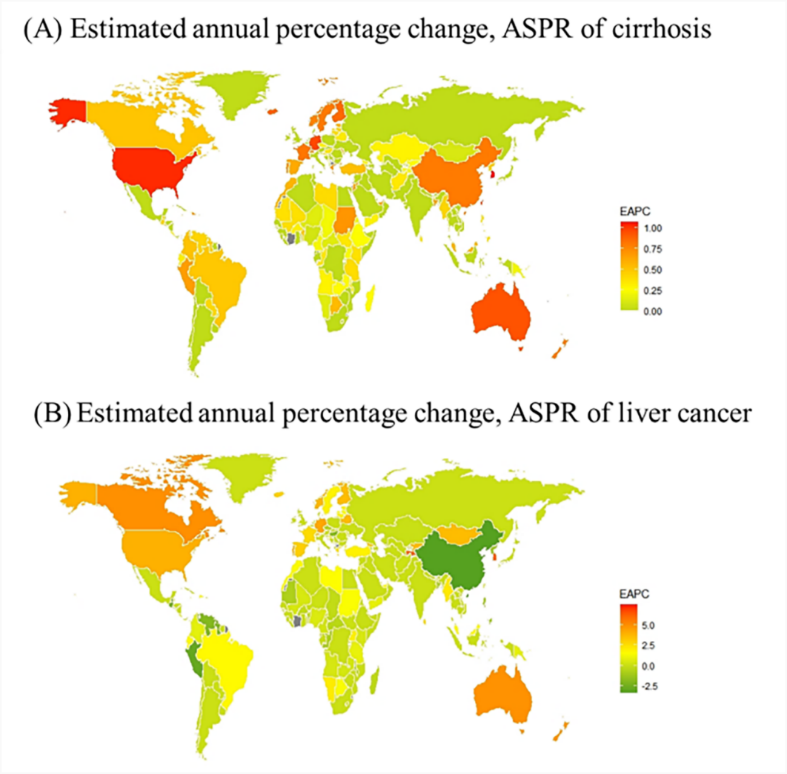


**Supplementary Figure 4.** Global age-specific prevalence, incidence, DALYs, and Death rate and number of NAFLD in 2019.


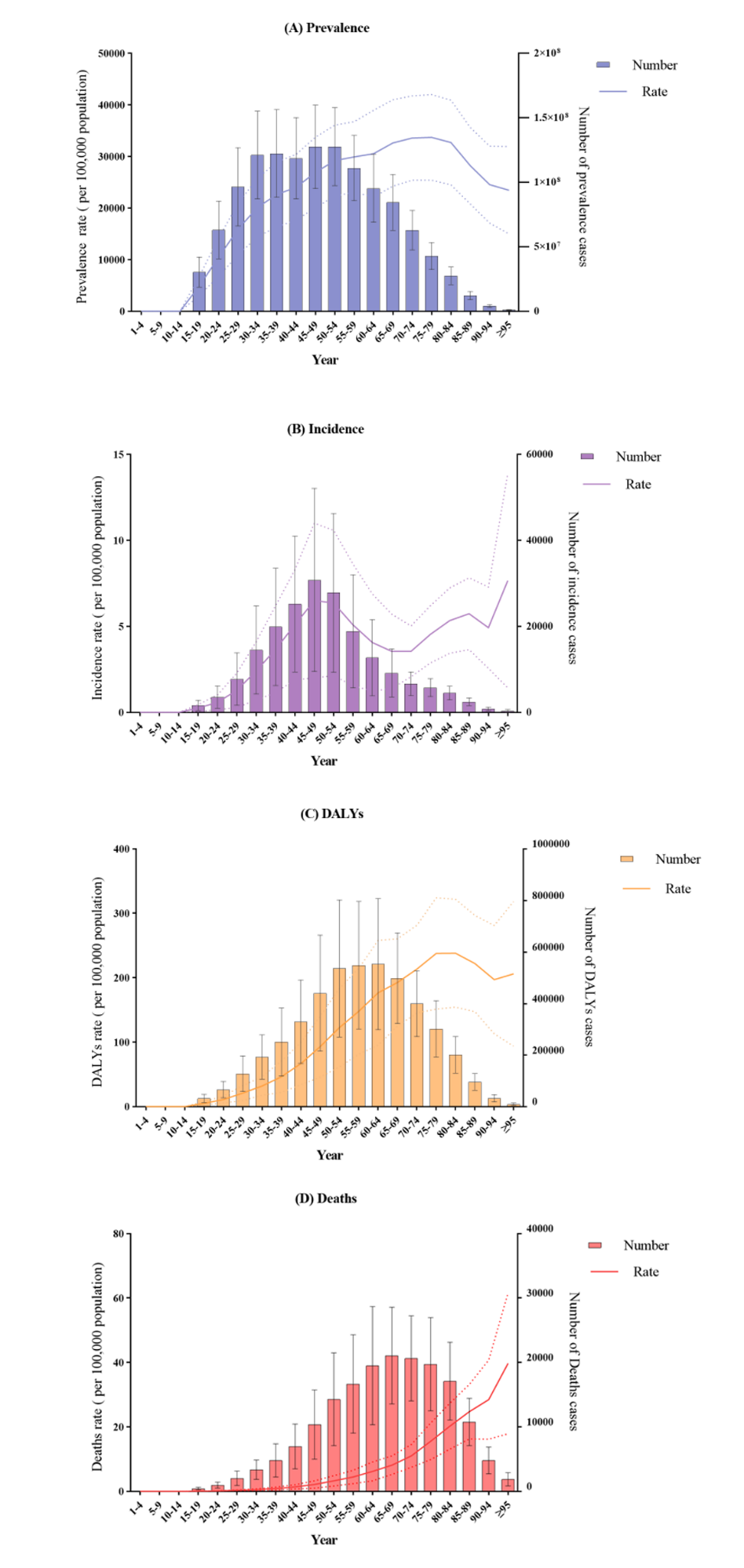


**Supplementary Figure 5.** Age-standardized prevalence, incidence, DALYs, and Death rate of NAFLD globally and in 21 GBD regions by SDI, 1990-2019.


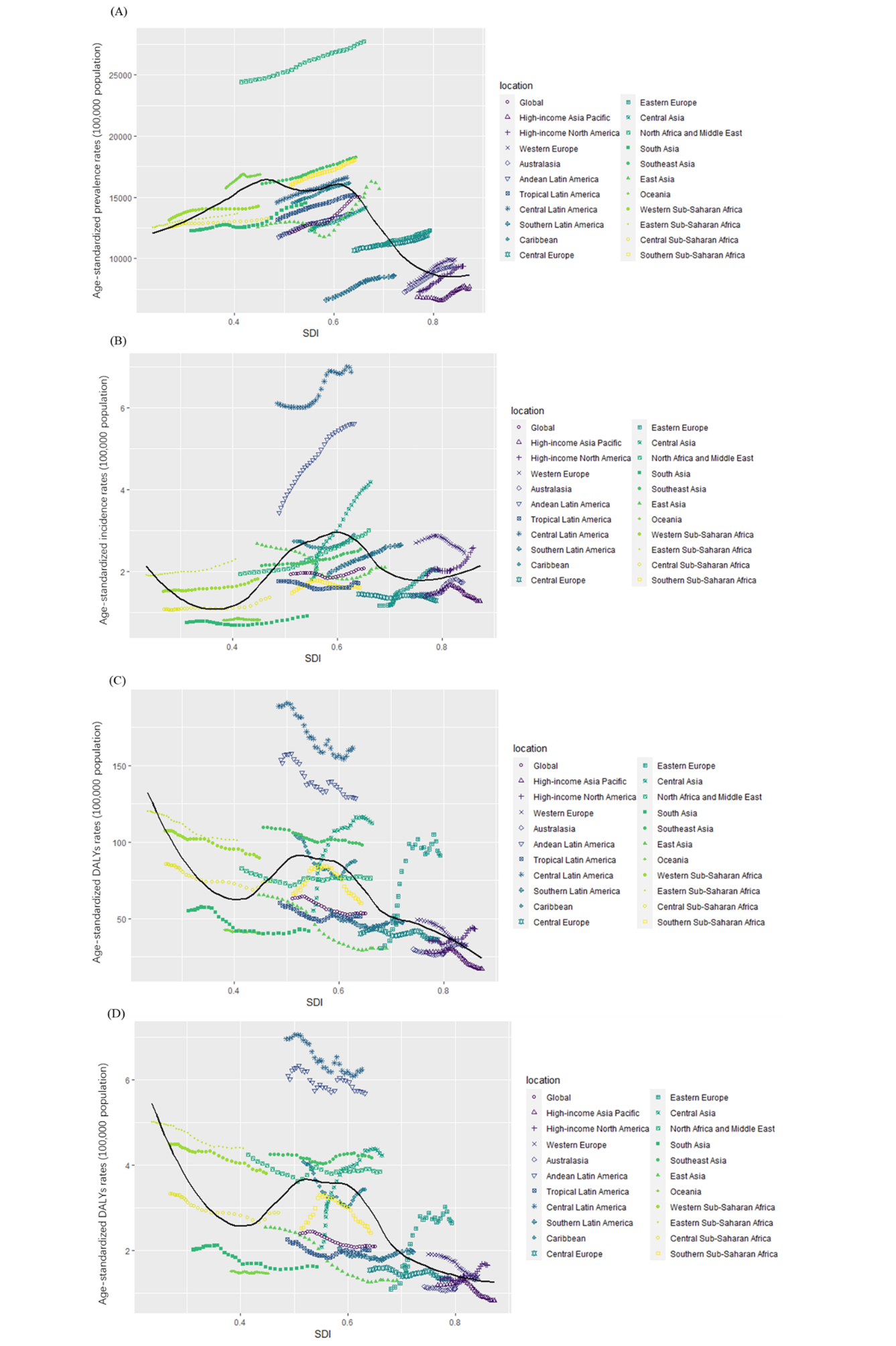


**Supplementary Table 1.** Global age-standardized prevalence, incidence, DALYs, and Death rate of NAFLD in both sexes from 1990 to 2019.

| Measure | Location | Year | Rate | 95% Upper UI | 95% Lower UI |
| --- | --- | --- | --- | --- | --- |
| Prevalence | Global | 1990 | 12065.6273 | 13536.97376 | 10779.51408 |
| Prevalence | Global | 1991 | 12158.39245 | 13637.19148 | 10866.7718 |
| Prevalence | Global | 1992 | 12246.28598 | 13731.69949 | 10947.98828 |
| Prevalence | Global | 1993 | 12330.19867 | 13828.42504 | 11019.94637 |
| Prevalence | Global | 1994 | 12409.05801 | 13915.40885 | 11087.3694 |
| Prevalence | Global | 1995 | 12482.51645 | 13987.744 | 11149.88329 |
| Prevalence | Global | 1996 | 12559.2526 | 14077.32428 | 11224.9455 |
| Prevalence | Global | 1997 | 12638.61403 | 14169.88886 | 11302.97119 |
| Prevalence | Global | 1998 | 12715.04477 | 14259.28422 | 11370.86386 |
| Prevalence | Global | 1999 | 12782.14117 | 14337.36341 | 11435.78723 |
| Prevalence | Global | 2000 | 12833.12183 | 14393.43783 | 11486.00299 |
| Prevalence | Global | 2001 | 12852.35681 | 14401.06555 | 11510.75332 |
| Prevalence | Global | 2002 | 12839.98156 | 14371.51812 | 11507.57556 |
| Prevalence | Global | 2003 | 12820.5031 | 14335.15901 | 11497.6967 |
| Prevalence | Global | 2004 | 12817.17068 | 14317.97028 | 11501.73063 |
| Prevalence | Global | 2005 | 12853.55156 | 14352.05084 | 11540.89133 |
| Prevalence | Global | 2006 | 12959.83423 | 14469.21966 | 11633.85259 |
| Prevalence | Global | 2007 | 13128.07935 | 14655.04248 | 11783.54563 |
| Prevalence | Global | 2008 | 13328.89125 | 14879.81131 | 11964.39647 |
| Prevalence | Global | 2009 | 13531.98346 | 15107.71463 | 12151.46453 |
| Prevalence | Global | 2010 | 13708.48074 | 15302.11761 | 12313.79574 |
| Prevalence | Global | 2011 | 13884.32381 | 15497.45279 | 12477.70716 |
| Prevalence | Global | 2012 | 14085.54905 | 15718.91856 | 12651.47731 |
| Prevalence | Global | 2013 | 14292.69612 | 15948.04344 | 12823.56223 |
| Prevalence | Global | 2014 | 14486.07608 | 16165.27806 | 12996.04836 |
| Prevalence | Global | 2015 | 14645.75962 | 16339.74825 | 13138.76873 |
| Prevalence | Global | 2016 | 14843.5792 | 16559.33418 | 13316.7408 |
| Prevalence | Global | 2017 | 15015.96007 | 16749.73423 | 13464.14904 |
| Prevalence | Global | 2018 | 15062.42283 | 16797.84659 | 13527.19461 |
| Prevalence | Global | 2019 | 15023.47346 | 16764.84154 | 13493.72762 |
| Incidence | Global | 1990 | 1.938241845 | 2.768330014 | 1.381595027 |
| Incidence | Global | 1991 | 1.946826337 | 2.76413417 | 1.402345728 |
| Incidence | Global | 1992 | 1.954296585 | 2.759298296 | 1.417642945 |
| Incidence | Global | 1993 | 1.960650421 | 2.755199413 | 1.436025788 |
| Incidence | Global | 1994 | 1.965975223 | 2.747268195 | 1.444182614 |
| Incidence | Global | 1995 | 1.970645338 | 2.752189829 | 1.455459926 |
| Incidence | Global | 1996 | 1.97358118 | 2.744677755 | 1.464643566 |
| Incidence | Global | 1997 | 1.973694161 | 2.733281206 | 1.470605092 |
| Incidence | Global | 1998 | 1.971278492 | 2.719559282 | 1.470143445 |
| Incidence | Global | 1999 | 1.966389655 | 2.713191152 | 1.467267472 |
| Incidence | Global | 2000 | 1.959080737 | 2.709819702 | 1.461313578 |
| Incidence | Global | 2001 | 1.942772133 | 2.693601161 | 1.45052228 |
| Incidence | Global | 2002 | 1.914628652 | 2.659808839 | 1.426498741 |
| Incidence | Global | 2003 | 1.883911318 | 2.616313897 | 1.397905276 |
| Incidence | Global | 2004 | 1.859380005 | 2.57998069 | 1.372776529 |
| Incidence | Global | 2005 | 1.849516465 | 2.562897228 | 1.361835085 |
| Incidence | Global | 2006 | 1.853227532 | 2.567510519 | 1.371065617 |
| Incidence | Global | 2007 | 1.861693669 | 2.577253491 | 1.383209073 |
| Incidence | Global | 2008 | 1.872974618 | 2.589504507 | 1.395131761 |
| Incidence | Global | 2009 | 1.88556702 | 2.603555614 | 1.40467187 |
| Incidence | Global | 2010 | 1.898134353 | 2.619265658 | 1.413404264 |
| Incidence | Global | 2011 | 1.913598422 | 2.638398156 | 1.423690695 |
| Incidence | Global | 2012 | 1.934823153 | 2.666340106 | 1.43892565 |
| Incidence | Global | 2013 | 1.958735621 | 2.698461059 | 1.457136779 |
| Incidence | Global | 2014 | 1.982623632 | 2.731039504 | 1.473894197 |
| Incidence | Global | 2015 | 2.003649807 | 2.762990029 | 1.483321523 |
| Incidence | Global | 2016 | 2.034196779 | 2.834227898 | 1.491107017 |
| Incidence | Global | 2017 | 2.061923251 | 2.903263935 | 1.509801649 |
| Incidence | Global | 2018 | 2.073072619 | 2.907385611 | 1.514556393 |
| Incidence | Global | 2019 | 2.07942373 | 2.925991658 | 1.517317984 |
| DALYs | Global | 1990 | 63.27695445 | 80.86191797 | 48.57578191 |
| DALYs | Global | 1991 | 63.40766764 | 80.47155246 | 48.73902102 |
| DALYs | Global | 1992 | 63.58901241 | 81.75094599 | 49.13441042 |
| DALYs | Global | 1993 | 64.21842754 | 81.96901359 | 49.47041377 |
| DALYs | Global | 1994 | 64.6269623 | 82.50147667 | 49.54493763 |
| DALYs | Global | 1995 | 64.44968144 | 81.91798756 | 50.07739315 |
| DALYs | Global | 1996 | 63.78457133 | 81.84482645 | 49.33681803 |
| DALYs | Global | 1997 | 62.89076152 | 80.18000974 | 48.55102172 |
| DALYs | Global | 1998 | 61.93091515 | 77.99970675 | 47.47336722 |
| DALYs | Global | 1999 | 61.08056302 | 77.06894748 | 47.49754847 |
| DALYs | Global | 2000 | 60.11542991 | 76.18292113 | 46.55468782 |
| DALYs | Global | 2001 | 58.99578891 | 74.87176602 | 45.82030848 |
| DALYs | Global | 2002 | 58.07382269 | 73.46443967 | 44.54984111 |
| DALYs | Global | 2003 | 57.24940926 | 72.59929773 | 43.65692518 |
| DALYs | Global | 2004 | 56.21810773 | 71.92393595 | 42.89886855 |
| DALYs | Global | 2005 | 55.97703892 | 71.54830512 | 42.63506383 |
| DALYs | Global | 2006 | 55.06090214 | 70.05009927 | 41.95297328 |
| DALYs | Global | 2007 | 54.56538511 | 69.45399536 | 41.46565428 |
| DALYs | Global | 2008 | 54.59242021 | 69.78404001 | 41.67176881 |
| DALYs | Global | 2009 | 53.93084502 | 68.84170285 | 41.30511097 |
| DALYs | Global | 2010 | 53.35614147 | 67.90144433 | 41.0423737 |
| DALYs | Global | 2011 | 52.82459074 | 67.22502064 | 40.27504018 |
| DALYs | Global | 2012 | 52.72877206 | 67.36748394 | 40.31195159 |
| DALYs | Global | 2013 | 52.96092066 | 67.39469881 | 40.52737199 |
| DALYs | Global | 2014 | 53.13555531 | 67.6987777 | 40.51264884 |
| DALYs | Global | 2015 | 53.45247784 | 67.97524216 | 41.21105647 |
| DALYs | Global | 2016 | 53.60313636 | 68.26292694 | 41.12070754 |
| DALYs | Global | 2017 | 53.77686116 | 68.72818884 | 40.88091026 |
| DALYs | Global | 2018 | 53.55825682 | 68.27580765 | 41.08285641 |
| DALYs | Global | 2019 | 53.3267928 | 68.28922947 | 40.7299943 |
| Deaths | Global | 1990 | 2.394932462 | 3.050516968 | 1.839684075 |
| Deaths | Global | 1991 | 2.401078877 | 3.036186737 | 1.847968356 |
| Deaths | Global | 1992 | 2.408936818 | 3.053527367 | 1.860573535 |
| Deaths | Global | 1993 | 2.434035325 | 3.075028144 | 1.88539382 |
| Deaths | Global | 1994 | 2.445671579 | 3.078471588 | 1.885082119 |
| Deaths | Global | 1995 | 2.441083947 | 3.082075902 | 1.894581314 |
| Deaths | Global | 1996 | 2.422579008 | 3.043328285 | 1.876488995 |
| Deaths | Global | 1997 | 2.397044713 | 3.005959548 | 1.857757255 |
| Deaths | Global | 1998 | 2.371227674 | 2.965732267 | 1.854978247 |
| Deaths | Global | 1999 | 2.345522099 | 2.93051409 | 1.839040504 |
| Deaths | Global | 2000 | 2.313005963 | 2.88768694 | 1.791408097 |
| Deaths | Global | 2001 | 2.276110633 | 2.85383574 | 1.758494759 |
| Deaths | Global | 2002 | 2.250023199 | 2.825531032 | 1.743642329 |
| Deaths | Global | 2003 | 2.224990198 | 2.811776003 | 1.716337659 |
| Deaths | Global | 2004 | 2.185592447 | 2.755834432 | 1.685690257 |
| Deaths | Global | 2005 | 2.17438608 | 2.741718793 | 1.663056051 |
| Deaths | Global | 2006 | 2.14161623 | 2.689165174 | 1.653753273 |
| Deaths | Global | 2007 | 2.123831227 | 2.66293538 | 1.630521776 |
| Deaths | Global | 2008 | 2.127440054 | 2.675255567 | 1.632349709 |
| Deaths | Global | 2009 | 2.107425763 | 2.646997418 | 1.627618117 |
| Deaths | Global | 2010 | 2.088179105 | 2.624011334 | 1.613397482 |
| Deaths | Global | 2011 | 2.072012071 | 2.599078636 | 1.596380689 |
| Deaths | Global | 2012 | 2.072016905 | 2.59826921 | 1.61591868 |
| Deaths | Global | 2013 | 2.082699635 | 2.609568773 | 1.603818531 |
| Deaths | Global | 2014 | 2.0913015 | 2.627903495 | 1.612916706 |
| Deaths | Global | 2015 | 2.101607283 | 2.640942179 | 1.620884052 |
| Deaths | Global | 2016 | 2.102886684 | 2.642039818 | 1.621802725 |
| Deaths | Global | 2017 | 2.103031339 | 2.638750882 | 1.610200798 |
| Deaths | Global | 2018 | 2.093932275 | 2.634348239 | 1.610761295 |
| Deaths | Global | 2019 | 2.087758117 | 2.599749087 | 1.613922804 |

**Supplementary Table 2.** The estimated annual percentage changes (EAPCs) of age-standardized prevalence, incidence, DALYs, and Death rate of NAFLD worldwide from 1990 to 2019.

| Location | Prevalence | | | Incidence | | | DALYs | | | Deaths | | |
| --- | --- | --- | --- | --- | --- | --- | --- | --- | --- | --- | --- | --- |
|  | APC | Upper CI | Lower CI | APC | Upper CI | Lower CI | APC | Upper CI | Lower CI | APC | Upper CI | Lower CI |
| Global | 0.77 | 0.8 | 0.7 | 0.1 | 0.2 | 0 | -0.82 | -0.7 | -0.9 | -0.7 | -0.6 | -0.7 |
| China | 0.81 | 1.1 | 0.5 | -1.28 | -0.9 | -1.7 | -3.54 | -3.1 | -3.9 | -3.16 | -2.8 | -3.5 |
| Afghanistan | 0.34 | 0.4 | 0.3 | 0.44 | 0.6 | 0.3 | -0.61 | -0.5 | -0.7 | -0.5 | -0.4 | -0.6 |
| Albania | 0.36 | 0.4 | 0.3 | -1.02 | -0.8 | -1.2 | -1.9 | -1.6 | -2.2 | -2.03 | -1.7 | -2.3 |
| Algeria | 0.58 | 0.6 | 0.5 | 0.87 | 1.1 | 0.6 | -0.61 | -0.4 | -0.8 | -0.42 | -0.2 | -0.6 |
| American Samoa | 0.2 | 0.4 | 0 | 0.45 | 0.6 | 0.3 | -0.1 | 0 | -0.2 | -0.04 | 0 | -0.1 |
| Andorra | 0.8 | 0.9 | 0.7 | 0.14 | 0.2 | 0.1 | -0.12 | -0.1 | -0.2 | -0.09 | 0 | -0.2 |
| Angola | 0.3 | 0.3 | 0.3 | 1.05 | 1.3 | 0.8 | -0.75 | -0.6 | -0.8 | -0.6 | -0.5 | -0.7 |
| Antigua and Barbuda | 0.45 | 0.5 | 0.4 | -0.2 | 0 | -0.4 | -1.26 | -0.9 | -1.7 | -1.03 | -0.6 | -1.4 |
| Argentina | 0.94 | 1 | 0.8 | 1.38 | 1.4 | 1.3 | 0.18 | 0.4 | -0.1 | 0.47 | 0.7 | 0.3 |
| Armenia | 0.66 | 0.7 | 0.6 | 4.99 | 5.4 | 4.6 | 3.96 | 4.7 | 3.3 | 5.04 | 5.8 | 4.2 |
| Australia | 0.94 | 1 | 0.9 | 1.05 | 1.2 | 0.9 | 0.75 | 1 | 0.5 | 0.93 | 1.2 | 0.7 |
| Austria | 0.88 | 0.9 | 0.8 | -0.69 | -0.6 | -0.8 | -1.9 | -1.8 | -2 | -1.39 | -1.3 | -1.5 |
| Azerbaijan | 0.49 | 0.5 | 0.4 | 2.36 | 2.6 | 2.1 | 0.53 | 1 | 0.1 | 1.21 | 1.7 | 0.7 |
| Bahamas | 0.35 | 0.4 | 0.3 | -0.85 | -0.7 | -1 | -1.49 | -1.2 | -1.8 | -1.49 | -1.2 | -1.8 |
| Bahrain | 0.49 | 0.5 | 0.4 | 1.23 | 1.5 | 1 | -1.2 | -0.9 | -1.5 | -0.72 | -0.4 | -1 |
| Bangladesh | 0.64 | 0.7 | 0.6 | 0.93 | 1 | 0.9 | -2.22 | -2 | -2.4 | -1.72 | -1.5 | -2 |
| Barbados | 0.33 | 0.3 | 0.3 | -0.11 | 0 | -0.2 | -1.02 | -0.8 | -1.2 | -0.81 | -0.6 | -1 |
| Belarus | 0.33 | 0.4 | 0.3 | 3.91 | 4.4 | 3.4 | 4.68 | 5.8 | 3.6 | 4.14 | 5.1 | 3.2 |
| Belgium | 0.8 | 0.9 | 0.7 | 0.11 | 0.2 | 0 | -0.49 | -0.4 | -0.6 | -0.33 | -0.2 | -0.4 |
| Belize | 0.52 | 0.6 | 0.4 | 0.52 | 0.6 | 0.5 | 0.3 | 0.6 | 0 | 0.02 | 0.3 | -0.2 |
| Benin | 0.47 | 0.5 | 0.4 | 0.24 | 0.5 | 0 | -0.65 | -0.6 | -0.8 | -0.67 | -0.6 | -0.8 |
| Bermuda | 0.31 | 0.3 | 0.3 | -1.52 | -1.3 | -1.8 | -3.48 | -3 | -4 | -3.51 | -3 | -4 |
| Bhutan | 0.84 | 0.9 | 0.8 | 2.04 | 2.2 | 1.9 | 0.6 | 0.8 | 0.5 | 1.11 | 1.3 | 1 |
| Bolivia (Plurinational State of) | 0.4 | 0.4 | 0.4 | 1.46 | 1.5 | 1.4 | -0.34 | -0.3 | -0.4 | 0.08 | 0.1 | 0 |
| Bosnia and Herzegovina | 0.61 | 0.7 | 0.6 | 0.82 | 1 | 0.7 | -0.05 | 0.1 | -0.2 | 0.48 | 0.7 | 0.3 |
| Botswana | 0.55 | 0.6 | 0.5 | 1.09 | 1.3 | 0.8 | -0.46 | -0.1 | -0.9 | -0.43 | 0 | -0.8 |
| Brazil | 0.49 | 0.5 | 0.5 | -0.25 | -0.1 | -0.4 | -0.49 | -0.3 | -0.7 | -0.31 | -0.1 | -0.5 |
| Brunei Darussalam | 0.55 | 0.6 | 0.5 | 0.2 | 0.4 | 0 | -0.1 | 0 | -0.3 | 0.09 | 0.3 | -0.1 |
| Bulgaria | 0.08 | 0.1 | 0 | 0.58 | 0.8 | 0.4 | 1.08 | 1.3 | 0.8 | 0.79 | 1 | 0.6 |
| Burkina Faso | 0.31 | 0.3 | 0.3 | 0.14 | 0.3 | -0.1 | -1.63 | -1.3 | -2 | -1.78 | -1.4 | -2.1 |
| Burundi | 0.25 | 0.3 | 0.2 | 0.5 | 0.6 | 0.4 | -1.5 | -1.3 | -1.7 | -1.41 | -1.2 | -1.6 |
| Cabo Verde | 0.45 | 0.5 | 0.4 | 2.86 | 3.4 | 2.4 | 1 | 1.4 | 0.6 | 1.5 | 2 | 1 |
| Cambodia | 0.28 | 0.3 | 0.3 | 0.64 | 0.8 | 0.4 | -0.46 | -0.3 | -0.6 | -0.02 | 0.2 | -0.2 |
| Cameroon | 0.22 | 0.2 | 0.2 | 0.32 | 0.4 | 0.2 | -1.08 | -0.9 | -1.2 | -1.19 | -1 | -1.3 |
| Canada | 0.51 | 0.6 | 0.5 | 0.74 | 0.8 | 0.7 | 0.56 | 0.7 | 0.5 | 0.62 | 0.7 | 0.5 |
| Central African Republic | 0.13 | 0.1 | 0.1 | 0.02 | 0.1 | -0.1 | -0.55 | -0.4 | -0.7 | -0.49 | -0.4 | -0.6 |
| Chad | 0.21 | 0.2 | 0.2 | -0.26 | -0.2 | -0.3 | -0.2 | -0.1 | -0.3 | -0.3 | -0.2 | -0.4 |
| Chile | 0.92 | 1 | 0.8 | 0.3 | 0.4 | 0.2 | -1.24 | -1 | -1.4 | -0.81 | -0.7 | -1 |
| Colombia | 0.51 | 0.5 | 0.5 | -0.43 | -0.4 | -0.5 | -1.56 | -1.4 | -1.7 | -1.31 | -1.1 | -1.5 |
| Comoros | 0.28 | 0.3 | 0.3 | 0.61 | 0.7 | 0.5 | -0.34 | -0.1 | -0.6 | -0.21 | 0 | -0.4 |
| Congo | 0.35 | 0.4 | 0.3 | 0.23 | 0.4 | 0.1 | -1 | -0.8 | -1.2 | -0.78 | -0.6 | -0.9 |
| Cook Islands | 0.55 | 0.6 | 0.5 | 0.34 | 0.4 | 0.2 | -0.32 | -0.3 | -0.4 | -0.31 | -0.2 | -0.4 |
| Costa Rica | 0.31 | 0.3 | 0.3 | 0.26 | 0.3 | 0.2 | -0.12 | 0.1 | -0.4 | 0.09 | 0.3 | -0.1 |
| Croatia | 0.39 | 0.4 | 0.4 | 0.06 | 0.3 | -0.1 | -1.39 | -1.2 | -1.6 | -0.94 | -0.8 | -1.1 |
| Cuba | 0.49 | 0.5 | 0.5 | -0.07 | 0.1 | -0.3 | -0.22 | 0.2 | -0.6 | -0.6 | -0.2 | -1 |
| Cyprus | 0.79 | 0.9 | 0.7 | -0.24 | -0.1 | -0.4 | -1.85 | -1.7 | -1.9 | -1.83 | -1.7 | -1.9 |
| Czechia | 0.3 | 0.3 | 0.3 | 0.08 | 0.2 | 0 | -0.17 | 0 | -0.3 | -0.22 | -0.1 | -0.4 |
| C么te d'Ivoire | 0.26 | 0.3 | 0.2 | -0.09 | 0.1 | -0.3 | -0.9 | -0.8 | -1 | -0.92 | -0.8 | -1.1 |
| Democratic People's Republic of Korea | 0.47 | 0.5 | 0.4 | -0.68 | -0.4 | -0.9 | -0.76 | -0.6 | -1 | -0.7 | -0.5 | -0.9 |
| Democratic Republic of the Congo | 0.08 | 0.1 | 0.1 | 0.52 | 0.7 | 0.3 | -0.78 | -0.7 | -0.9 | -0.87 | -0.7 | -1 |
| Denmark | 0.85 | 0.9 | 0.8 | 0.94 | 1.3 | 0.6 | -1.11 | -0.7 | -1.5 | -0.33 | 0.1 | -0.7 |
| Djibouti | 0.46 | 0.5 | 0.4 | 0.09 | 0.2 | -0.1 | -0.39 | -0.3 | -0.5 | -0.27 | -0.2 | -0.4 |
| Dominica | 0.38 | 0.4 | 0.3 | -1.09 | -0.9 | -1.3 | -1.52 | -1.3 | -1.8 | -1.45 | -1.2 | -1.7 |
| Dominican Republic | 0.71 | 0.8 | 0.7 | 0.9 | 1 | 0.8 | -0.59 | -0.1 | -1 | -0.33 | 0.1 | -0.8 |
| Ecuador | 0.31 | 0.4 | 0.3 | 2.93 | 3.2 | 2.7 | 0.43 | 0.6 | 0.2 | 1.13 | 1.4 | 0.9 |
| Egypt | 0.29 | 0.3 | 0.3 | 2.2 | 2.4 | 2 | 0.86 | 1.1 | 0.6 | 0.77 | 1 | 0.5 |
| El Salvador | 0.58 | 0.7 | 0.5 | 0.75 | 0.8 | 0.6 | -0.5 | -0.3 | -0.7 | -0.17 | 0 | -0.3 |
| Equatorial Guinea | 1.07 | 1.1 | 1 | 0.59 | 0.7 | 0.5 | -1.71 | -1.6 | -1.8 | -1.21 | -1.1 | -1.3 |
| Eritrea | 0.21 | 0.2 | 0.2 | 1.02 | 1.2 | 0.8 | 0.41 | 0.5 | 0.3 | 0.56 | 0.7 | 0.4 |
| Estonia | 0.56 | 0.6 | 0.5 | 1.85 | 2.2 | 1.5 | 2.03 | 2.8 | 1.3 | 1.96 | 2.7 | 1.3 |
| Eswatini | 0.2 | 0.4 | 0 | 2.84 | 3.4 | 2.3 | 1.84 | 2.4 | 1.2 | 1.78 | 2.4 | 1.2 |
| Ethiopia | 0.24 | 0.2 | 0.2 | 0.3 | 0.4 | 0.2 | -1.85 | -1.7 | -2 | -1.41 | -1.3 | -1.5 |
| Fiji | 0.46 | 0.5 | 0.4 | 0.75 | 0.8 | 0.7 | 0.33 | 0.4 | 0.3 | 0.43 | 0.5 | 0.4 |
| Finland | 0.87 | 1 | 0.8 | 1.86 | 2.2 | 1.5 | 1.49 | 1.9 | 1.1 | 1.59 | 2 | 1.2 |
| France | 0.82 | 0.9 | 0.8 | -0.91 | -0.8 | -1 | -1.89 | -1.8 | -2 | -1.6 | -1.5 | -1.7 |
| Gabon | 0.73 | 0.8 | 0.7 | 0.67 | 0.8 | 0.6 | -0.64 | -0.5 | -0.8 | -0.43 | -0.3 | -0.6 |
| Gambia | 0.29 | 0.3 | 0.3 | 1.03 | 1.2 | 0.8 | 0.39 | 0.7 | 0.1 | 0.35 | 0.6 | 0.1 |
| Georgia | 0.28 | 0.3 | 0.3 | 0.39 | 0.5 | 0.2 | 1.03 | 1.2 | 0.9 | 0.66 | 0.9 | 0.5 |
| Germany | 0.97 | 1 | 0.9 | 0.14 | 0.3 | 0 | -1.51 | -1.4 | -1.7 | -0.96 | -0.8 | -1.1 |
| Ghana | 0.52 | 0.6 | 0.5 | 0.08 | 0.2 | -0.1 | -1.12 | -1 | -1.3 | -1.13 | -1 | -1.3 |
| Greece | 0.72 | 0.8 | 0.6 | -0.95 | -0.8 | -1.1 | -0.99 | -0.7 | -1.2 | -1.61 | -1.4 | -1.8 |
| Greenland | 0.51 | 0.5 | 0.5 | 0.63 | 0.7 | 0.6 | -0.19 | -0.1 | -0.3 | 0.04 | 0.1 | 0 |
| Grenada | 0.48 | 0.5 | 0.4 | -0.45 | -0.2 | -0.7 | -1.3 | -1 | -1.6 | -1.25 | -0.9 | -1.5 |
| Guam | 0.57 | 0.6 | 0.5 | -0.19 | -0.1 | -0.3 | -0.4 | -0.2 | -0.6 | -0.97 | -0.7 | -1.3 |
| Guatemala | 0.42 | 0.4 | 0.4 | 0.84 | 0.9 | 0.8 | -0.66 | -0.4 | -0.9 | -0.39 | -0.2 | -0.6 |
| Guinea | 0.13 | 0.1 | 0.1 | 0.39 | 0.4 | 0.3 | 0.08 | 0.1 | 0 | 0.02 | 0.1 | -0.1 |
| Guinea-Bissau | 0.14 | 0.2 | 0.1 | -0.45 | -0.3 | -0.6 | -0.3 | -0.2 | -0.4 | -0.3 | -0.2 | -0.4 |
| Guyana | 0.47 | 0.5 | 0.4 | 0.23 | 0.4 | 0 | -0.42 | -0.2 | -0.7 | -0.47 | -0.2 | -0.8 |
| Haiti | 0.22 | 0.2 | 0.2 | 0.6 | 0.6 | 0.6 | -1.11 | -1 | -1.2 | -1.07 | -1 | -1.2 |
| Honduras | 0.4 | 0.4 | 0.4 | 0.34 | 0.4 | 0.2 | 0.62 | 0.7 | 0.5 | 1.19 | 1.4 | 1 |
| Hungary | 0.35 | 0.4 | 0.3 | -2.51 | -2.4 | -2.7 | -4.16 | -3.8 | -4.6 | -3.48 | -3.1 | -3.8 |
| Iceland | 0.89 | 1 | 0.8 | 0.66 | 0.7 | 0.6 | -0.73 | -0.6 | -0.8 | -0.48 | -0.4 | -0.6 |
| India | 0.5 | 0.6 | 0.4 | 0.32 | 0.7 | -0.1 | -1.47 | -1.1 | -1.8 | -1.24 | -1 | -1.5 |
| Indonesia | 0.49 | 0.5 | 0.5 | -0.49 | -0.4 | -0.6 | -0.28 | -0.2 | -0.4 | 0.42 | 0.5 | 0.3 |
| Iran (Islamic Republic of) | 0.53 | 0.7 | 0.4 | 1.19 | 1.3 | 1.1 | -0.28 | -0.1 | -0.4 | -0.27 | -0.1 | -0.4 |
| Iraq | 0.35 | 0.4 | 0.3 | 1.5 | 1.7 | 1.3 | -0.07 | 0.1 | -0.2 | 0.12 | 0.3 | -0.1 |
| Ireland | 0.86 | 0.9 | 0.8 | 1.49 | 1.7 | 1.3 | 1.28 | 1.7 | 0.9 | 1.26 | 1.6 | 0.9 |
| Israel | 0.97 | 1.1 | 0.9 | -0.18 | 0 | -0.3 | -0.96 | -0.7 | -1.2 | -0.76 | -0.5 | -1 |
| Italy | 0.73 | 0.8 | 0.6 | -1.25 | -1 | -1.5 | -3.24 | -3.1 | -3.4 | -2.9 | -2.7 | -3.1 |
| Jamaica | 0.58 | 0.6 | 0.5 | -0.29 | -0.1 | -0.5 | -0.87 | -0.5 | -1.2 | -0.71 | -0.4 | -1 |
| Japan | 0.3 | 0.4 | 0.2 | -0.86 | -0.7 | -1.1 | -2.65 | -2.4 | -2.9 | -2.1 | -1.8 | -2.4 |
| Jordan | 0.65 | 0.7 | 0.6 | 1.02 | 1.1 | 1 | -1.2 | -1 | -1.4 | -1 | -0.8 | -1.2 |
| Kazakhstan | 0.3 | 0.3 | 0.3 | 4.06 | 4.3 | 3.8 | 3.63 | 4.3 | 3 | 3.49 | 4 | 3 |
| Kenya | 0.38 | 0.4 | 0.4 | 0.28 | 0.5 | 0.1 | 0.44 | 0.5 | 0.3 | 0.48 | 0.6 | 0.4 |
| Kiribati | 0.23 | 0.4 | 0.1 | 0.12 | 0.2 | 0.1 | -0.83 | -0.8 | -0.9 | -0.72 | -0.7 | -0.8 |
| Kuwait | 0.48 | 0.5 | 0.4 | 2.6 | 2.9 | 2.3 | 0.96 | 1.6 | 0.3 | 1.43 | 2 | 0.8 |
| Kyrgyzstan | 0.26 | 0.3 | 0.3 | 1.83 | 2 | 1.7 | 1.42 | 1.9 | 0.9 | 1.29 | 1.8 | 0.8 |
| Lao People's Democratic Republic | 0.34 | 0.4 | 0.3 | 0.21 | 0.4 | 0 | -1.16 | -1 | -1.4 | -0.83 | -0.6 | -1 |
| Latvia | 0.42 | 0.4 | 0.4 | 2.05 | 2.4 | 1.7 | 2.3 | 3 | 1.6 | 2.01 | 2.6 | 1.4 |
| Lebanon | 0.52 | 0.6 | 0.5 | 1.98 | 2.2 | 1.8 | -0.32 | -0.1 | -0.5 | -0.27 | -0.1 | -0.4 |
| Lesotho | 0.47 | 0.5 | 0.4 | 1.87 | 2 | 1.8 | 1.92 | 2.1 | 1.7 | 1.85 | 2 | 1.7 |
| Liberia | 0.47 | 0.5 | 0.4 | 0.23 | 0.4 | 0.1 | -1.03 | -0.9 | -1.2 | -1.02 | -0.9 | -1.2 |
| Libya | 0.41 | 0.4 | 0.4 | 1 | 1.2 | 0.8 | 0.04 | 0.3 | -0.2 | 0.05 | 0.3 | -0.2 |
| Lithuania | 0.39 | 0.4 | 0.4 | 3.18 | 3.7 | 2.7 | 4.15 | 5.1 | 3.2 | 3.81 | 4.7 | 2.9 |
| Luxembourg | 0.72 | 0.8 | 0.7 | -0.97 | -0.7 | -1.2 | -2.03 | -1.9 | -2.1 | -1.74 | -1.6 | -1.8 |
| Madagascar | 0.26 | 0.3 | 0.2 | 0.98 | 1.2 | 0.7 | -0.4 | -0.3 | -0.5 | -0.35 | -0.3 | -0.4 |
| Malawi | 0.34 | 0.4 | 0.3 | 0.08 | 0.2 | 0 | -1.13 | -1 | -1.3 | -1.05 | -0.9 | -1.2 |
| Malaysia | 0.56 | 0.6 | 0.5 | 1.12 | 1.3 | 0.9 | 0.6 | 0.9 | 0.3 | 0.81 | 1.2 | 0.4 |
| Maldives | 0.73 | 0.8 | 0.7 | 0.99 | 1.2 | 0.8 | -1.37 | -1.1 | -1.6 | -0.79 | -0.6 | -1 |
| Mali | 0.38 | 0.4 | 0.4 | 0.92 | 1 | 0.8 | -1.22 | -1.1 | -1.4 | -1.27 | -1.1 | -1.4 |
| Malta | 0.95 | 1.1 | 0.8 | -0.24 | -0.2 | -0.3 | -1.13 | -1.1 | -1.2 | -1.22 | -1.1 | -1.3 |
| Marshall Islands | 0.36 | 0.4 | 0.3 | 0.09 | 0.2 | 0 | -0.57 | -0.5 | -0.6 | -0.57 | -0.5 | -0.6 |
| Mauritania | 0.38 | 0.4 | 0.3 | -0.31 | -0.1 | -0.5 | -2.04 | -1.9 | -2.1 | -2.01 | -1.9 | -2.1 |
| Mauritius | 0.57 | 0.6 | 0.6 | -0.56 | -0.4 | -0.7 | -1.68 | -1.5 | -1.9 | -1.45 | -1.3 | -1.6 |
| Mexico | 0.37 | 0.4 | 0.4 | 0.8 | 0.9 | 0.7 | -0.85 | -0.7 | -1 | -0.56 | -0.5 | -0.7 |
| Micronesia (Federated States of) | 0.27 | 0.4 | 0.1 | 0.29 | 0.4 | 0.2 | -0.53 | -0.5 | -0.6 | -0.42 | -0.3 | -0.5 |
| Monaco | 0.73 | 0.8 | 0.7 | 1.41 | 1.6 | 1.2 | 1.09 | 1.4 | 0.8 | 1.29 | 1.6 | 1 |
| Mongolia | 0.09 | 0.1 | 0 | 3.38 | 3.9 | 2.9 | 1.79 | 2.2 | 1.4 | 2.7 | 3.2 | 2.2 |
| Montenegro | 0.25 | 0.3 | 0.2 | 0.39 | 0.5 | 0.3 | 0.23 | 0.4 | 0 | 0.42 | 0.6 | 0.3 |
| Morocco | 0.57 | 0.6 | 0.5 | 1.3 | 1.6 | 1 | 0.16 | 0.4 | -0.1 | 0.27 | 0.5 | 0 |
| Mozambique | 0.15 | 0.2 | 0.1 | 0.98 | 1.2 | 0.8 | 0.2 | 0.3 | 0.1 | 0.11 | 0.2 | 0 |
| Myanmar | 0.43 | 0.5 | 0.4 | 0.87 | 1.1 | 0.7 | -0.01 | 0.2 | -0.2 | 0.18 | 0.3 | 0 |
| Namibia | 0.13 | 0.2 | 0.1 | 0.56 | 0.7 | 0.4 | -0.63 | -0.4 | -0.9 | -0.51 | -0.3 | -0.7 |
| Nauru | 0.15 | 0.2 | 0.1 | 0.38 | 0.5 | 0.3 | -0.23 | -0.2 | -0.3 | -0.17 | -0.1 | -0.2 |
| Nepal | 0.9 | 1 | 0.8 | 1.62 | 1.8 | 1.5 | 0.07 | 0.3 | -0.2 | 0.44 | 0.7 | 0.2 |
| Netherlands | 0.7 | 0.7 | 0.7 | 0.12 | 0.2 | 0 | -0.88 | -0.6 | -1.1 | -0.67 | -0.4 | -0.9 |
| New Zealand | 0.8 | 0.9 | 0.7 | 0.79 | 0.8 | 0.8 | -0.01 | 0.1 | -0.1 | 0.16 | 0.2 | 0.1 |
| Nicaragua | 0.45 | 0.5 | 0.4 | 1.45 | 1.7 | 1.2 | 1.1 | 1.3 | 1 | 1.45 | 1.6 | 1.3 |
| Niger | 0.13 | 0.2 | 0.1 | 0.15 | 0.2 | 0 | -1.01 | -0.8 | -1.2 | -1.03 | -0.9 | -1.2 |
| Nigeria | 0.07 | 0.1 | 0 | 0.95 | 1.1 | 0.8 | -0.27 | -0.1 | -0.4 | -0.14 | 0 | -0.3 |
| Niue | 0.53 | 0.6 | 0.4 | 0.63 | 0.8 | 0.4 | -0.34 | -0.2 | -0.5 | -0.12 | 0 | -0.3 |
| North Macedonia | 0.38 | 0.4 | 0.4 | 0.8 | 0.9 | 0.7 | 0.31 | 0.4 | 0.2 | 0.65 | 0.7 | 0.6 |
| Northern Mariana Islands | 0.25 | 0.4 | 0.1 | -0.38 | -0.2 | -0.5 | -1.65 | -1.5 | -1.8 | -1.49 | -1.4 | -1.6 |
| Norway | 0.77 | 0.8 | 0.7 | 0.98 | 1.2 | 0.7 | -0.86 | -0.8 | -0.9 | -0.5 | -0.4 | -0.6 |
| Oman | 0.89 | 0.9 | 0.9 | 2.16 | 2.4 | 1.9 | 0.81 | 1.1 | 0.5 | 1.18 | 1.4 | 0.9 |
| Pakistan | 0.64 | 0.7 | 0.6 | 0.1 | 0.1 | 0.1 | -0.51 | -0.4 | -0.6 | -0.44 | -0.3 | -0.6 |
| Palau | 0.45 | 0.6 | 0.3 | 0.9 | 1.1 | 0.7 | 0.28 | 0.4 | 0.1 | 0.27 | 0.4 | 0.1 |
| Palestine | 0.29 | 0.3 | 0.2 | 0.81 | 1 | 0.6 | -0.26 | -0.1 | -0.4 | -0.1 | 0.1 | -0.2 |
| Panama | 0.54 | 0.6 | 0.5 | 0.43 | 0.6 | 0.3 | 0.07 | 0.2 | -0.1 | 0.13 | 0.3 | 0 |
| Papua New Guinea | 0.2 | 0.2 | 0.2 | 0.58 | 0.6 | 0.5 | 0.34 | 0.4 | 0.3 | 0.51 | 0.6 | 0.4 |
| Paraguay | 0.42 | 0.4 | 0.4 | 0.56 | 0.8 | 0.3 | 0.68 | 1 | 0.4 | 0.61 | 0.9 | 0.4 |
| Peru | 0.66 | 0.7 | 0.6 | 0.98 | 1.1 | 0.9 | -1.49 | -1.3 | -1.7 | -1.13 | -0.9 | -1.3 |
| Philippines | 0.38 | 0.4 | 0.4 | 0.75 | 0.9 | 0.6 | -0.57 | -0.4 | -0.8 | -0.4 | -0.2 | -0.6 |
| Poland | 0.32 | 0.3 | 0.3 | -1.44 | -1.3 | -1.6 | 0.14 | 0.5 | -0.2 | -0.86 | -0.4 | -1.3 |
| Portugal | 0.74 | 0.8 | 0.6 | -2.04 | -1.9 | -2.2 | -2.91 | -2.8 | -3 | -2.69 | -2.5 | -2.8 |
| Puerto Rico | 0.55 | 0.6 | 0.5 | -1.14 | -1 | -1.2 | -2.57 | -2.3 | -2.9 | -2.27 | -2 | -2.5 |
| Qatar | 0.42 | 0.5 | 0.4 | 2.15 | 2.4 | 1.9 | 0.29 | 0.6 | 0 | 0.99 | 1.2 | 0.7 |
| Republic of Korea | 1.07 | 1.4 | 0.7 | 0.52 | 1.1 | 0 | -1.53 | -1 | -2.1 | -0.97 | -0.4 | -1.5 |
| Republic of Moldova | 0.57 | 0.6 | 0.5 | -0.12 | 0.2 | -0.5 | -0.31 | 0.2 | -0.8 | -0.36 | 0.1 | -0.8 |
| Romania | 0.53 | 0.5 | 0.5 | 1.01 | 1.3 | 0.7 | 0.52 | 0.9 | 0.1 | 0.69 | 1 | 0.4 |
| Russian Federation | 0.42 | 0.4 | 0.4 | 2.54 | 2.7 | 2.4 | 4.24 | 5.1 | 3.4 | 3.53 | 4.1 | 2.9 |
| Rwanda | 0.31 | 0.3 | 0.3 | 0.57 | 0.6 | 0.5 | -2.67 | -2.3 | -3 | -2.43 | -2.1 | -2.8 |
| Saint Kitts and Nevis | 0.37 | 0.4 | 0.3 | -1.13 | -0.8 | -1.4 | -2.54 | -2.1 | -3 | -2.25 | -1.8 | -2.7 |
| Saint Lucia | 0.49 | 0.5 | 0.4 | -0.78 | -0.5 | -1.1 | -2.1 | -1.6 | -2.6 | -2.24 | -1.7 | -2.8 |
| Saint Vincent and the Grenadines | 0.6 | 0.6 | 0.6 | -0.56 | -0.3 | -0.8 | -0.78 | -0.3 | -1.2 | -0.82 | -0.3 | -1.3 |
| Samoa | 0.04 | 0.2 | -0.1 | -0.11 | 0 | -0.2 | -0.7 | -0.7 | -0.8 | -0.69 | -0.6 | -0.8 |
| San Marino | 0.75 | 0.8 | 0.7 | 0.16 | 0.2 | 0.1 | 0.09 | 0.2 | 0 | 0.09 | 0.2 | -0.1 |
| Sao Tome and Principe | 0.31 | 0.3 | 0.3 | 0.22 | 0.4 | 0.1 | -0.75 | -0.5 | -1 | -0.63 | -0.4 | -0.8 |
| Saudi Arabia | 0.65 | 0.7 | 0.6 | 1.29 | 1.4 | 1.2 | -1.18 | -1 | -1.3 | -0.94 | -0.8 | -1.1 |
| Senegal | 0.2 | 0.2 | 0.2 | 0.42 | 0.6 | 0.2 | -0.46 | -0.2 | -0.7 | -0.53 | -0.3 | -0.7 |
| Serbia | 0.29 | 0.3 | 0.3 | 0.1 | 0.2 | 0 | -0.6 | -0.4 | -0.8 | -0.3 | -0.2 | -0.4 |
| Seychelles | 0.44 | 0.5 | 0.4 | 0.64 | 0.8 | 0.5 | 0.17 | 0.3 | 0 | 0.47 | 0.7 | 0.3 |
| Sierra Leone | 0.17 | 0.2 | 0.2 | 0.92 | 1.1 | 0.7 | -1.3 | -1.1 | -1.5 | -1.51 | -1.3 | -1.8 |
| Singapore | 0.66 | 0.7 | 0.6 | 0.12 | 0.2 | 0 | -1.05 | -1 | -1.1 | -0.34 | -0.2 | -0.4 |
| Slovakia | 0.28 | 0.3 | 0.2 | 0.14 | 0.3 | 0 | -0.04 | 0.2 | -0.3 | 0.03 | 0.2 | -0.2 |
| Slovenia | 0.48 | 0.5 | 0.4 | 0.18 | 0.3 | 0 | -1.42 | -1.2 | -1.6 | -0.79 | -0.6 | -1 |
| Solomon Islands | 0.31 | 0.4 | 0.2 | 0.22 | 0.4 | 0.1 | -0.04 | 0.1 | -0.2 | 0.01 | 0.2 | -0.1 |
| Somalia | 0.11 | 0.1 | 0.1 | 0.12 | 0.2 | 0 | -0.3 | -0.2 | -0.4 | -0.19 | -0.1 | -0.3 |
| South Africa | 0.48 | 0.5 | 0.5 | -0.14 | 0.1 | -0.4 | -0.87 | -0.3 | -1.4 | -0.54 | -0.1 | -1 |
| South Sudan | 0.35 | 0.4 | 0.3 | 0.32 | 0.4 | 0.3 | -0.31 | -0.2 | -0.4 | -0.17 | -0.1 | -0.2 |
| Spain | 0.62 | 0.7 | 0.5 | -1.43 | -1.3 | -1.5 | -2.37 | -2.3 | -2.5 | -2.11 | -2 | -2.2 |
| Sri Lanka | 0.35 | 0.4 | 0.3 | 0.48 | 0.6 | 0.3 | -1.64 | -1.1 | -2.1 | -0.99 | -0.6 | -1.4 |
| Sudan | 0.7 | 0.8 | 0.6 | 1.19 | 1.6 | 0.8 | -0.27 | -0.2 | -0.4 | -0.15 | -0.1 | -0.2 |
| Suriname | 0.53 | 0.5 | 0.5 | 0.02 | 0.1 | -0.1 | -1.07 | -0.8 | -1.3 | -0.88 | -0.6 | -1.1 |
| Sweden | 0.81 | 0.9 | 0.8 | 0.87 | 1 | 0.8 | 0.23 | 0.5 | 0 | 0.54 | 0.8 | 0.3 |
| Switzerland | 0.74 | 0.8 | 0.7 | 0.2 | 0.4 | 0 | -1.02 | -0.8 | -1.2 | -0.45 | -0.2 | -0.7 |
| Syrian Arab Republic | 0.26 | 0.3 | 0.2 | 1.54 | 1.6 | 1.5 | -0.09 | 0.1 | -0.3 | 0.14 | 0.3 | -0.1 |
| Taiwan (Province of China) | 0.98 | 1.1 | 0.9 | -0.96 | -0.3 | -1.6 | -1.4 | -0.9 | -1.9 | -1.52 | -1 | -2 |
| Tajikistan | 0.23 | 0.3 | 0.2 | 3.56 | 3.7 | 3.4 | 1.39 | 1.6 | 1.2 | 2.07 | 2.3 | 1.9 |
| Thailand | 0.71 | 0.7 | 0.7 | 1.56 | 1.7 | 1.4 | 0.52 | 0.6 | 0.4 | 0.66 | 0.8 | 0.6 |
| Timor-Leste | 0.17 | 0.2 | 0.2 | 0.8 | 0.9 | 0.7 | -0.32 | 0 | -0.6 | 0.05 | 0.3 | -0.2 |
| Togo | 0.23 | 0.3 | 0.2 | -0.08 | 0 | -0.2 | -1.28 | -1.1 | -1.5 | -1.34 | -1.2 | -1.5 |
| Tokelau | 0.54 | 0.6 | 0.5 | -0.01 | 0 | -0.1 | -0.95 | -0.9 | -1 | -0.78 | -0.7 | -0.9 |
| Tonga | 0.2 | 0.4 | 0 | 0.5 | 0.8 | 0.2 | 0.17 | 0.5 | -0.1 | 0.21 | 0.5 | -0.1 |
| Trinidad and Tobago | 0.48 | 0.5 | 0.5 | -0.85 | -0.6 | -1.1 | -1.61 | -1.3 | -1.9 | -1.67 | -1.4 | -2 |
| Tunisia | 0.5 | 0.5 | 0.5 | 1.28 | 1.4 | 1.2 | -0.24 | -0.2 | -0.3 | -0.15 | -0.1 | -0.2 |
| Turkey | 0.53 | 0.6 | 0.5 | 2.27 | 2.7 | 1.8 | -0.5 | -0.1 | -0.9 | 0.15 | 0.5 | -0.2 |
| Turkmenistan | 0.7 | 0.8 | 0.6 | 1.57 | 1.7 | 1.5 | 2.18 | 2.4 | 1.9 | 1.49 | 1.7 | 1.2 |
| Tuvalu | 0.4 | 0.5 | 0.3 | 0.25 | 0.3 | 0.2 | -0.57 | -0.4 | -0.7 | -0.54 | -0.4 | -0.7 |
| Uganda | 0.23 | 0.2 | 0.2 | 0.84 | 0.9 | 0.8 | -0.44 | -0.2 | -0.6 | -0.34 | -0.2 | -0.5 |
| Ukraine | 0.24 | 0.3 | 0.2 | 2.26 | 2.4 | 2.1 | 4.45 | 5.3 | 3.7 | 3.65 | 4.3 | 3 |
| United Arab Emirates | 0.63 | 0.7 | 0.6 | 1.13 | 1.4 | 0.9 | 0.11 | 0.6 | -0.4 | 0.01 | 0.6 | -0.5 |
| United Kingdom | 0.8 | 0.9 | 0.7 | 1.93 | 2.1 | 1.7 | 1.87 | 2.2 | 1.5 | 1.86 | 2.2 | 1.6 |
| United Republic of Tanzania | 0.4 | 0.4 | 0.4 | 0.56 | 0.6 | 0.5 | -0.56 | -0.4 | -0.7 | -0.49 | -0.3 | -0.7 |
| United States of America | 1.03 | 1.1 | 1 | 0.82 | 1 | 0.6 | 0.85 | 1.1 | 0.6 | 0.93 | 1.1 | 0.7 |
| United States Virgin Islands | 0.52 | 0.6 | 0.5 | 0.35 | 0.4 | 0.3 | -0.38 | -0.2 | -0.6 | -0.29 | -0.1 | -0.5 |
| Uruguay | 0.79 | 0.9 | 0.7 | 0.17 | 0.2 | 0.1 | -1.01 | -0.9 | -1.2 | -0.74 | -0.6 | -0.9 |
| Uzbekistan | 0.73 | 0.8 | 0.7 | 2.85 | 3.1 | 2.6 | 2.09 | 2.6 | 1.6 | 2.33 | 2.9 | 1.8 |
| Vanuatu | 0.36 | 0.4 | 0.3 | 0.36 | 0.4 | 0.3 | -0.3 | -0.2 | -0.4 | -0.28 | -0.2 | -0.4 |
| Venezuela (Bolivarian Republic of) | 0.44 | 0.5 | 0.4 | -0.84 | -0.7 | -1 | -0.99 | -0.7 | -1.2 | -0.98 | -0.7 | -1.2 |
| Viet Nam | 0.34 | 0.4 | 0.3 | 0.52 | 0.8 | 0.2 | -1.43 | -1 | -1.9 | -1.36 | -0.9 | -1.8 |
| Yemen | 0.3 | 0.3 | 0.3 | 0.85 | 1.1 | 0.6 | -0.6 | -0.5 | -0.7 | -0.59 | -0.5 | -0.6 |
| Zambia | 0.26 | 0.3 | 0.2 | 0.52 | 0.7 | 0.4 | -0.6 | -0.4 | -0.8 | -0.58 | -0.4 | -0.8 |
| Zimbabwe | -0.06 | 0 | -0.1 | 0.12 | 0.4 | -0.1 | 0.5 | 0.8 | 0.2 | 0.31 | 0.6 | 0.1 |

**Supplementary Table 3.** Age-standardized prevalence, incidence, DALYs，and Death rate of  NAFLD for 204 countries and territories in 2019.

| Measure | location | Rate | 95% Upper UI | 95% Lower UI |
| --- | --- | --- | --- | --- |
| Prevalence | China | 15705.17078 | 17576.32095 | 14042.19067 |
| Prevalence | Afghanistan | 22288.49609 | 24815.33692 | 20060.30123 |
| Prevalence | Albania | 16966.71937 | 18787.54086 | 15262.48913 |
| Prevalence | Algeria | 26699.53985 | 29383.93563 | 24286.55006 |
| Prevalence | American Samoa | 22396.89303 | 24679.73893 | 20313.41995 |
| Prevalence | Andorra | 9643.629135 | 10761.18814 | 8641.999383 |
| Prevalence | Angola | 13491.65657 | 15180.51365 | 11964.57 |
| Prevalence | Antigua and Barbuda | 15628.48573 | 17417.98295 | 13981.84899 |
| Prevalence | Argentina | 8022.570576 | 8945.429625 | 7210.273791 |
| Prevalence | Armenia | 14888.8342 | 16454.13231 | 13335.97514 |
| Prevalence | Australia | 9546.99655 | 10596.49934 | 8557.765362 |
| Prevalence | Austria | 8952.845163 | 10026.53623 | 7971.146713 |
| Prevalence | Azerbaijan | 15785.92153 | 17604.3206 | 14179.59516 |
| Prevalence | Bahamas | 16650.95161 | 18580.4396 | 14907.41043 |
| Prevalence | Bahrain | 30275.26915 | 32914.5693 | 27661.32718 |
| Prevalence | Bangladesh | 14786.24409 | 16807.71858 | 13099.56086 |
| Prevalence | Barbados | 17849.91908 | 19844.98349 | 15986.78531 |
| Prevalence | Belarus | 10926.57874 | 12120.17218 | 9793.402877 |
| Prevalence | Belgium | 8816.242804 | 9872.918214 | 7868.225845 |
| Prevalence | Belize | 18376.67784 | 20263.26324 | 16580.42829 |
| Prevalence | Benin | 14088.82017 | 15797.63299 | 12509.99891 |
| Prevalence | Bermuda | 18917.48656 | 20987.66444 | 17036.38553 |
| Prevalence | Bhutan | 16281.63254 | 18325.07778 | 14547.99373 |
| Prevalence | Bolivia (Plurinational State of) | 13040.34404 | 14467.12274 | 11748.82125 |
| Prevalence | Bosnia and Herzegovina | 18863.85084 | 20974.75951 | 16970.42953 |
| Prevalence | Botswana | 16210.8867 | 18172.01815 | 14353.24013 |
| Prevalence | Brazil | 15300.23706 | 16887.4972 | 13774.79289 |
| Prevalence | Brunei Darussalam | 8303.934014 | 9327.296744 | 7404.207372 |
| Prevalence | Bulgaria | 11159.82627 | 12350.19282 | 10052.28466 |
| Prevalence | Burkina Faso | 13065.03505 | 14661.62059 | 11665.55947 |
| Prevalence | Burundi | 12962.22725 | 14654.03434 | 11519.52802 |
| Prevalence | Cabo Verde | 16721.83291 | 18701.48702 | 14908.71159 |
| Prevalence | Cambodia | 17022.85609 | 19080.94077 | 15148.34234 |
| Prevalence | Cameroon | 17629.31496 | 19689.4377 | 15787.17672 |
| Prevalence | Canada | 6876.446669 | 7797.850068 | 6114.713526 |
| Prevalence | Central African Republic | 12630.40851 | 14325.31684 | 11169.79326 |
| Prevalence | Chad | 14590.90331 | 16388.91734 | 13043.08599 |
| Prevalence | Chile | 9434.294176 | 10491.04623 | 8417.716242 |
| Prevalence | Colombia | 13263.03099 | 14769.16823 | 11856.78909 |
| Prevalence | Comoros | 17710.34407 | 19794.77594 | 15873.23507 |
| Prevalence | Congo | 14341.72996 | 16099.05652 | 12827.01572 |
| Prevalence | Cook Islands | 21836.94944 | 24028.27855 | 19774.77209 |
| Prevalence | Costa Rica | 12719.82086 | 14090.52054 | 11433.88816 |
| Prevalence | Croatia | 12619.28578 | 13997.20865 | 11300.33491 |
| Prevalence | Cuba | 17035.5908 | 18929.46412 | 15265.24391 |
| Prevalence | Cyprus | 9053.211185 | 10139.40866 | 8028.150041 |
| Prevalence | Czechia | 11024.50761 | 12181.43014 | 9940.240466 |
| Prevalence | C么te d'Ivoire | 15527.27512 | 17352.70689 | 13857.5567 |
| Prevalence | Democratic People's Republic of Korea | 13489.69581 | 15356.75468 | 11969.32553 |
| Prevalence | Democratic Republic of the Congo | 13127.35694 | 14912.67171 | 11608.85831 |
| Prevalence | Denmark | 7731.508949 | 8658.219759 | 6887.877341 |
| Prevalence | Djibouti | 14399.70805 | 16165.36503 | 12751.99645 |
| Prevalence | Dominica | 16600.69352 | 18481.63633 | 14877.91314 |
| Prevalence | Dominican Republic | 14866.4488 | 16427.21614 | 13398.15217 |
| Prevalence | Ecuador | 20749.8193 | 22899.22588 | 18780.42426 |
| Prevalence | Egypt | 34518.01759 | 37254.41529 | 31798.97733 |
| Prevalence | El Salvador | 16079.85998 | 17963.98942 | 14413.02027 |
| Prevalence | Equatorial Guinea | 15981.72491 | 17854.07715 | 14188.29432 |
| Prevalence | Eritrea | 12261.66345 | 13894.78689 | 10837.46416 |
| Prevalence | Estonia | 10973.43099 | 12094.82124 | 9929.681163 |
| Prevalence | Eswatini | 19554.32857 | 21757.43789 | 17547.06937 |
| Prevalence | Ethiopia | 13518.20403 | 15236.23786 | 12018.5675 |
| Prevalence | Fiji | 20992.72757 | 23280.25809 | 18876.85253 |
| Prevalence | Finland | 7119.904909 | 8075.312661 | 6337.761478 |
| Prevalence | France | 8148.93456 | 9158.915414 | 7300.272993 |
| Prevalence | Gabon | 16461.11421 | 18402.43893 | 14602.15612 |
| Prevalence | Gambia | 16231.81554 | 18141.93696 | 14417.74781 |
| Prevalence | Georgia | 13296.38161 | 14691.19003 | 11965.96752 |
| Prevalence | Germany | 7887.516029 | 8799.916045 | 7064.590804 |
| Prevalence | Ghana | 15215.29848 | 16995.90361 | 13617.1021 |
| Prevalence | Greece | 9466.711667 | 10597.42203 | 8434.040077 |
| Prevalence | Greenland | 6680.99066 | 7591.183552 | 5909.81111 |
| Prevalence | Grenada | 16020.18639 | 17922.64882 | 14363.73021 |
| Prevalence | Guam | 19630.88867 | 21520.06902 | 17846.27557 |
| Prevalence | Guatemala | 18644.01362 | 20933.07465 | 16768.40218 |
| Prevalence | Guinea | 15411.27905 | 17341.69606 | 13721.62669 |
| Prevalence | Guinea-Bissau | 15198.05087 | 17323.60007 | 13480.05233 |
| Prevalence | Guyana | 16455.21774 | 18237.51206 | 14729.17597 |
| Prevalence | Haiti | 13719.72934 | 15427.05067 | 12253.02033 |
| Prevalence | Honduras | 18974.94766 | 21203.90357 | 17148.07825 |
| Prevalence | Hungary | 11996.10045 | 13246.17788 | 10821.66608 |
| Prevalence | Iceland | 9191.375405 | 10243.6275 | 8244.633856 |
| Prevalence | India | 14186.99876 | 16025.6035 | 12644.4209 |
| Prevalence | Indonesia | 21062.20558 | 23308.82214 | 19010.32139 |
| Prevalence | Iran (Islamic Republic of) | 28066.50481 | 30769.3285 | 25534.19154 |
| Prevalence | Iraq | 26914.78303 | 29559.81606 | 24381.45732 |
| Prevalence | Ireland | 10935.2975 | 12186.98145 | 9787.911771 |
| Prevalence | Israel | 15833.97007 | 17730.58485 | 14075.78427 |
| Prevalence | Italy | 14545.35803 | 16075.11005 | 13131.46608 |
| Prevalence | Jamaica | 17614.45061 | 19640.00098 | 15741.92504 |
| Prevalence | Japan | 7457.487461 | 8365.806624 | 6653.712887 |
| Prevalence | Jordan | 29382.06194 | 32200.84124 | 26827.93171 |
| Prevalence | Kazakhstan | 12937.28923 | 14270.24618 | 11644.96144 |
| Prevalence | Kenya | 16256.2946 | 18137.89797 | 14553.60272 |
| Prevalence | Kiribati | 18766.98175 | 20797.16334 | 16862.6825 |
| Prevalence | Kuwait | 31675.18998 | 34531.55476 | 29116.80773 |
| Prevalence | Kyrgyzstan | 14734.00895 | 16431.38372 | 13227.02689 |
| Prevalence | Lao People's Democratic Republic | 13511.53243 | 15022.01001 | 12134.26714 |
| Prevalence | Latvia | 11124.51453 | 12298.30416 | 10008.674 |
| Prevalence | Lebanon | 26547.1115 | 29106.26738 | 24218.17338 |
| Prevalence | Lesotho | 14511.43317 | 16209.99894 | 13046.5461 |
| Prevalence | Liberia | 16544.71854 | 18553.60369 | 14761.28713 |
| Prevalence | Libya | 28282.68915 | 31154.64078 | 25719.94347 |
| Prevalence | Lithuania | 11119.84893 | 12287.40831 | 10019.45859 |
| Prevalence | Luxembourg | 8861.093567 | 9890.532792 | 7913.600257 |
| Prevalence | Madagascar | 13089.39933 | 14781.63818 | 11565.7763 |
| Prevalence | Malawi | 15825.65876 | 17698.5242 | 14109.72816 |
| Prevalence | Malaysia | 22192.40602 | 24654.28706 | 20091.49887 |
| Prevalence | Maldives | 18523.11609 | 20660.80701 | 16547.4868 |
| Prevalence | Mali | 17622.79561 | 19825.81663 | 15714.35168 |
| Prevalence | Malta | 9765.235598 | 10913.38015 | 8756.762575 |
| Prevalence | Marshall Islands | 17258.15867 | 19262.26274 | 15442.77314 |
| Prevalence | Mauritania | 20896.92444 | 23315.83998 | 18755.72153 |
| Prevalence | Mauritius | 15595.23529 | 17268.60606 | 13971.08615 |
| Prevalence | Mexico | 17688.18658 | 19526.37004 | 15965.85715 |
| Prevalence | Micronesia (Federated States of) | 18872.18971 | 20958.39761 | 16897.79882 |
| Prevalence | Monaco | 10706.4262 | 11832.66349 | 9610.122918 |
| Prevalence | Mongolia | 12538.34642 | 14038.91592 | 11218.22942 |
| Prevalence | Montenegro | 12758.74088 | 14180.15317 | 11449.20363 |
| Prevalence | Morocco | 27157.63963 | 29829.4076 | 24654.99068 |
| Prevalence | Mozambique | 11459.35232 | 12929.03443 | 10211.21349 |
| Prevalence | Myanmar | 18130.97695 | 20289.93553 | 16378.71361 |
| Prevalence | Namibia | 13046.05548 | 14605.98854 | 11678.10561 |
| Prevalence | Nauru | 19069.87027 | 21164.59043 | 17105.46591 |
| Prevalence | Nepal | 14824.12776 | 16814.98585 | 13221.92998 |
| Prevalence | Netherlands | 9026.988962 | 10056.90184 | 8091.352178 |
| Prevalence | New Zealand | 8877.450502 | 9866.674649 | 7938.223514 |
| Prevalence | Nicaragua | 16539.45298 | 18469.75064 | 14852.72753 |
| Prevalence | Niger | 14968.46068 | 16870.34253 | 13285.45673 |
| Prevalence | Nigeria | 12493.3796 | 13926.42333 | 11138.61287 |
| Prevalence | Niue | 20579.80454 | 22861.65938 | 18539.81233 |
| Prevalence | North Macedonia | 13001.96979 | 14485.56406 | 11612.5767 |
| Prevalence | Northern Mariana Islands | 20692.15158 | 22814.38238 | 18643.78357 |
| Prevalence | Norway | 8250.488143 | 9173.701415 | 7389.942609 |
| Prevalence | Oman | 29220.04341 | 32000.91604 | 26720.28929 |
| Prevalence | Pakistan | 16924.67588 | 18972.47874 | 15181.01575 |
| Prevalence | Palau | 20883.14102 | 23064.39586 | 18752.88082 |
| Prevalence | Palestine | 25507.94734 | 28100.25804 | 23162.25667 |
| Prevalence | Panama | 16681.71802 | 18697.8735 | 14939.55145 |
| Prevalence | Papua New Guinea | 15976.83136 | 17789.54012 | 14273.48323 |
| Prevalence | Paraguay | 13080.04609 | 14544.11704 | 11717.8759 |
| Prevalence | Peru | 10452.75949 | 11558.47306 | 9408.948274 |
| Prevalence | Philippines | 14396.82853 | 15986.73267 | 12953.69192 |
| Prevalence | Poland | 10984.18259 | 12063.8082 | 10006.3771 |
| Prevalence | Portugal | 11966.24424 | 13383.54446 | 10682.49706 |
| Prevalence | Puerto Rico | 20524.16419 | 22510.34866 | 18614.90102 |
| Prevalence | Qatar | 33318.54448 | 36169.84626 | 30578.07482 |
| Prevalence | Republic of Korea | 8016.731665 | 9001.791593 | 7124.71373 |
| Prevalence | Republic of Moldova | 11679.89991 | 12912.53358 | 10488.45985 |
| Prevalence | Romania | 12388.87655 | 13644.85047 | 11144.61639 |
| Prevalence | Russian Federation | 12545.75315 | 13860.6974 | 11338.42529 |
| Prevalence | Rwanda | 10420.06424 | 11761.1698 | 9262.690488 |
| Prevalence | Saint Kitts and Nevis | 16982.40831 | 18815.36729 | 15317.84077 |
| Prevalence | Saint Lucia | 16303.53766 | 18164.07648 | 14582.02274 |
| Prevalence | Saint Vincent and the Grenadines | 16274.39446 | 18068.9187 | 14550.77345 |
| Prevalence | Samoa | 19422.44893 | 21539.80688 | 17482.09734 |
| Prevalence | San Marino | 9755.787831 | 10880.82477 | 8743.250348 |
| Prevalence | Sao Tome and Principe | 17452.31758 | 19553.42348 | 15584.50981 |
| Prevalence | Saudi Arabia | 30527.28293 | 33095.23369 | 27958.71568 |
| Prevalence | Senegal | 18705.97623 | 20942.55107 | 16747.60513 |
| Prevalence | Serbia | 12306.8595 | 13731.65824 | 11048.31873 |
| Prevalence | Seychelles | 20206.65266 | 22359.41459 | 18127.16594 |
| Prevalence | Sierra Leone | 15595.20345 | 17534.8062 | 13837.20865 |
| Prevalence | Singapore | 9081.918272 | 10174.93317 | 8040.026032 |
| Prevalence | Slovakia | 10163.7975 | 11213.17424 | 9140.864787 |
| Prevalence | Slovenia | 12728.71522 | 14107.5456 | 11464.56917 |
| Prevalence | Solomon Islands | 17174.10317 | 19142.18011 | 15278.16174 |
| Prevalence | Somalia | 13232.26464 | 14886.30082 | 11702.15562 |
| Prevalence | South Africa | 18979.47626 | 21015.02814 | 17184.88027 |
| Prevalence | South Sudan | 15006.83846 | 16868.97592 | 13314.83431 |
| Prevalence | Spain | 11305.91052 | 12672.94174 | 10138.74071 |
| Prevalence | Sri Lanka | 17016.23307 | 18958.35066 | 15309.47796 |
| Prevalence | Sudan | 18093.41167 | 20420.8103 | 16191.40502 |
| Prevalence | Suriname | 16930.42239 | 18945.26442 | 15148.05785 |
| Prevalence | Sweden | 9018.484916 | 10014.00696 | 8074.49022 |
| Prevalence | Switzerland | 8100.658267 | 9084.796256 | 7254.675895 |
| Prevalence | Syrian Arab Republic | 27116.11215 | 29739.60465 | 24690.73423 |
| Prevalence | Taiwan (Province of China) | 16553.78376 | 18619.4071 | 14765.62893 |
| Prevalence | Tajikistan | 12097.61433 | 13619.84787 | 10796.10079 |
| Prevalence | Thailand | 18376.46453 | 20431.52303 | 16488.41782 |
| Prevalence | Timor-Leste | 15443.92024 | 17338.91303 | 13809.61643 |
| Prevalence | Togo | 15253.30271 | 17206.0843 | 13440.14616 |
| Prevalence | Tokelau | 19068.99946 | 21201.22992 | 17136.94865 |
| Prevalence | Tonga | 19986.40188 | 22188.52976 | 17943.5596 |
| Prevalence | Trinidad and Tobago | 17998.20558 | 20003.11274 | 16100.52334 |
| Prevalence | Tunisia | 27005.94523 | 29695.46619 | 24451.14682 |
| Prevalence | Turkey | 26651.08355 | 29300.07674 | 24129.88764 |
| Prevalence | Turkmenistan | 15113.73365 | 16821.48409 | 13600.35248 |
| Prevalence | Tuvalu | 17939.32919 | 19934.92293 | 16009.21379 |
| Prevalence | Uganda | 11075.1244 | 12519.35496 | 9817.243466 |
| Prevalence | Ukraine | 12002.56115 | 13265.06201 | 10794.71614 |
| Prevalence | United Arab Emirates | 30542.22277 | 33329.77093 | 27979.3057 |
| Prevalence | United Kingdom | 9572.464465 | 10659.85091 | 8599.590682 |
| Prevalence | United Republic of Tanzania | 14488.26691 | 16269.34549 | 12914.21734 |
| Prevalence | United States of America | 9687.28871 | 10877.24023 | 8654.130112 |
| Prevalence | United States Virgin Islands | 18157.47982 | 19968.95438 | 16440.79469 |
| Prevalence | Uruguay | 11239.77921 | 12549.71962 | 10050.94174 |
| Prevalence | Uzbekistan | 14915.86493 | 16629.24564 | 13348.46678 |
| Prevalence | Vanuatu | 18500.90367 | 20613.16807 | 16608.00718 |
| Prevalence | Venezuela (Bolivarian Republic of) | 17166.91066 | 19118.75861 | 15345.12416 |
| Prevalence | Viet Nam | 14761.68932 | 16551.45431 | 13192.00444 |
| Prevalence | Yemen | 21117.5606 | 23574.78474 | 18992.68175 |
| Prevalence | Zambia | 15183.99537 | 17104.643 | 13586.85505 |
| Prevalence | Zimbabwe | 15358.68856 | 17349.65847 | 13744.06618 |
| Incidence | China | 3.23913817 | 4.728817423 | 2.218628108 |
| Incidence | Afghanistan | 2.320552946 | 3.4262732 | 1.630271537 |
| Incidence | Albania | 4.146124941 | 5.641294701 | 3.121368907 |
| Incidence | Algeria | 1.873274074 | 2.766167841 | 1.301740103 |
| Incidence | American Samoa | 1.688166849 | 2.421281763 | 1.176989998 |
| Incidence | Andorra | 2.547858165 | 3.696620642 | 1.72911071 |
| Incidence | Angola | 4.186451839 | 6.176800869 | 2.903819944 |
| Incidence | Antigua and Barbuda | 1.998884981 | 2.771018305 | 1.481062736 |
| Incidence | Argentina | 1.259962297 | 1.72052302 | 0.914335435 |
| Incidence | Armenia | 1.938799017 | 2.761757266 | 1.345789983 |
| Incidence | Australia | 4.074219158 | 5.873023504 | 2.791212038 |
| Incidence | Austria | 1.919985044 | 3.07283488 | 1.079919191 |
| Incidence | Azerbaijan | 1.77723454 | 2.522208375 | 1.257225445 |
| Incidence | Bahamas | 4.331227464 | 6.73166444 | 2.806701795 |
| Incidence | Bahrain | 2.174112006 | 3.147368526 | 1.530133378 |
| Incidence | Bangladesh | 2.408847872 | 3.337114485 | 1.720048157 |
| Incidence | Barbados | 4.577188848 | 6.836561515 | 3.025073644 |
| Incidence | Belarus | 2.14371927 | 3.036450278 | 1.490954802 |
| Incidence | Belgium | 1.894354946 | 2.681009348 | 1.314059997 |
| Incidence | Belize | 1.346755728 | 1.908950317 | 0.910693761 |
| Incidence | Benin | 2.359560961 | 3.309572851 | 1.672366067 |
| Incidence | Bermuda | 1.507970911 | 2.174702122 | 1.020391576 |
| Incidence | Bhutan | 2.875857549 | 4.433830313 | 1.814775446 |
| Incidence | Bolivia (Plurinational State of) | 2.00952819 | 3.029362873 | 1.257798571 |
| Incidence | Bosnia and Herzegovina | 3.755911043 | 5.320211456 | 2.575212866 |
| Incidence | Botswana | 2.499176714 | 3.700816954 | 1.69459191 |
| Incidence | Brazil | 1.798661682 | 2.577381119 | 1.23760799 |
| Incidence | Brunei Darussalam | 3.387459591 | 5.122929138 | 2.220785445 |
| Incidence | Bulgaria | 1.997197982 | 2.917253402 | 1.373457287 |
| Incidence | Burkina Faso | 1.546393864 | 2.238347608 | 1.040220995 |
| Incidence | Burundi | 2.623794144 | 3.888188355 | 1.785299241 |
| Incidence | Cabo Verde | 2.252106691 | 3.266006278 | 1.523267002 |
| Incidence | Cambodia | 3.211264505 | 4.738618173 | 2.131160541 |
| Incidence | Cameroon | 1.905638365 | 2.841478192 | 1.288282702 |
| Incidence | Canada | 1.914899503 | 2.811491831 | 1.319170781 |
| Incidence | Central African Republic | 2.211637317 | 3.262013612 | 1.49203809 |
| Incidence | Chad | 1.690548146 | 2.433710119 | 1.169871645 |
| Incidence | Chile | 1.329739709 | 1.843812731 | 0.954533089 |
| Incidence | Colombia | 2.499212248 | 3.452203917 | 1.797076392 |
| Incidence | Comoros | 5.269408625 | 7.6676461 | 3.522291298 |
| Incidence | Congo | 2.089289179 | 2.995776732 | 1.494834704 |
| Incidence | Cook Islands | 3.066901636 | 4.414656909 | 2.069338043 |
| Incidence | Costa Rica | 3.14155721 | 4.435970843 | 2.250430439 |
| Incidence | Croatia | 2.563298771 | 3.659017019 | 1.780480373 |
| Incidence | Cuba | 2.419421935 | 3.436179492 | 1.730102753 |
| Incidence | Cyprus | 4.222553421 | 6.460621545 | 2.682711212 |
| Incidence | Czechia | 3.244421874 | 4.720898165 | 2.148297495 |
| Incidence | C么te d'Ivoire | 2.602621499 | 3.968472951 | 1.69090867 |
| Incidence | Democratic People's Republic of Korea | 1.751380624 | 2.573229444 | 1.176708115 |
| Incidence | Democratic Republic of the Congo | 2.63638905 | 3.762126801 | 1.819658078 |
| Incidence | Denmark | 5.353572896 | 7.624990489 | 3.652310778 |
| Incidence | Djibouti | 3.053526122 | 4.506514043 | 2.037550156 |
| Incidence | Dominica | 3.463681204 | 5.077480827 | 2.323715586 |
| Incidence | Dominican Republic | 2.278180062 | 3.286274549 | 1.542743032 |
| Incidence | Ecuador | 7.505000722 | 10.98781975 | 5.039657189 |
| Incidence | Egypt | 1.414811711 | 2.043762647 | 0.958665507 |
| Incidence | El Salvador | 5.906594035 | 8.501269729 | 4.005558323 |
| Incidence | Equatorial Guinea | 5.767225005 | 8.395487268 | 3.894710315 |
| Incidence | Eritrea | 5.801157338 | 8.409276548 | 3.947677709 |
| Incidence | Estonia | 3.036086011 | 4.491120944 | 1.958745132 |
| Incidence | Eswatini | 1.712471427 | 2.527555878 | 1.156573696 |
| Incidence | Ethiopia | 0.691377648 | 1.041262944 | 0.469418085 |
| Incidence | Fiji | 10.13424066 | 14.92428382 | 6.614935668 |
| Incidence | Finland | 1.601247285 | 2.364405604 | 1.069221213 |
| Incidence | France | 0.928368234 | 1.401224031 | 0.606531683 |
| Incidence | Gabon | 2.116442084 | 3.005970296 | 1.498936886 |
| Incidence | Gambia | 1.563568463 | 2.326359134 | 1.057708639 |
| Incidence | Georgia | 0.579288892 | 0.89141686 | 0.3566379 |
| Incidence | Germany | 1.741604792 | 2.527238982 | 1.186024792 |
| Incidence | Ghana | 0.951687918 | 1.485578074 | 0.551041788 |
| Incidence | Greece | 1.150075654 | 1.697416619 | 0.775064007 |
| Incidence | Greenland | 0.951427536 | 1.437962801 | 0.597718676 |
| Incidence | Grenada | 2.081730666 | 2.960613593 | 1.403845381 |
| Incidence | Guam | 1.391835917 | 2.181377064 | 0.876754119 |
| Incidence | Guatemala | 1.31439957 | 1.823467915 | 0.929673793 |
| Incidence | Guinea | 1.490020432 | 2.079911202 | 1.065857422 |
| Incidence | Guinea-Bissau | 2.515577568 | 3.969547964 | 1.36413687 |
| Incidence | Guyana | 1.335637139 | 1.95626133 | 0.889135804 |
| Incidence | Haiti | 1.473402634 | 2.231793608 | 0.973862709 |
| Incidence | Honduras | 0.79216175 | 1.210085627 | 0.505834524 |
| Incidence | Hungary | 1.776539611 | 2.628024421 | 1.116519119 |
| Incidence | Iceland | 1.08400167 | 1.619686343 | 0.72520078 |
| Incidence | India | 0.831432314 | 1.223356445 | 0.568637313 |
| Incidence | Indonesia | 1.817377541 | 2.751455954 | 0.991693849 |
| Incidence | Iran (Islamic Republic of) | 0.98155678 | 1.446542168 | 0.680450177 |
| Incidence | Iraq | 2.340978199 | 3.19300479 | 1.69842085 |
| Incidence | Ireland | 1.313796068 | 1.946965448 | 0.885057313 |
| Incidence | Israel | 0.996554477 | 1.551767478 | 0.615139697 |
| Incidence | Italy | 0.834572683 | 1.22588799 | 0.571345364 |
| Incidence | Jamaica | 1.518909199 | 2.040667813 | 1.145527596 |
| Incidence | Japan | 4.152445622 | 5.719985739 | 2.934303343 |
| Incidence | Jordan | 1.176320947 | 1.742361833 | 0.807782188 |
| Incidence | Kazakhstan | 1.077033634 | 1.654699715 | 0.708750543 |
| Incidence | Kenya | 1.553290361 | 2.12772533 | 1.157526017 |
| Incidence | Kiribati | 1.157639885 | 1.673844163 | 0.822260565 |
| Incidence | Kuwait | 3.441449655 | 5.050217958 | 2.327099436 |
| Incidence | Kyrgyzstan | 4.121136146 | 5.933588512 | 2.833352337 |
| Incidence | Lao People's Democratic Republic | 1.070179725 | 1.591463846 | 0.727891933 |
| Incidence | Latvia | 1.507428445 | 2.208750835 | 1.007212277 |
| Incidence | Lebanon | 1.76601761 | 2.408687601 | 1.266950286 |
| Incidence | Lesotho | 2.198461341 | 2.976934008 | 1.586630652 |
| Incidence | Liberia | 3.164222979 | 4.505029923 | 2.245476731 |
| Incidence | Libya | 2.532813501 | 3.562363438 | 1.782090525 |
| Incidence | Lithuania | 2.349597146 | 3.350296457 | 1.666869969 |
| Incidence | Luxembourg | 0.36628591 | 0.568940458 | 0.225612909 |
| Incidence | Madagascar | 2.066127166 | 2.858483961 | 1.466887502 |
| Incidence | Malawi | 5.808435281 | 8.442271679 | 3.86971547 |
| Incidence | Malaysia | 2.207753781 | 3.178051807 | 1.527676747 |
| Incidence | Maldives | 3.159235518 | 4.675691488 | 2.053052837 |
| Incidence | Mali | 9.777964296 | 13.43832395 | 7.111517335 |
| Incidence | Malta | 1.751320506 | 2.571925073 | 1.181043984 |
| Incidence | Marshall Islands | 0.788235868 | 1.212979424 | 0.499853857 |
| Incidence | Mauritania | 1.429518061 | 1.976357077 | 1.022579614 |
| Incidence | Mauritius | 2.561762393 | 3.729676988 | 1.697238227 |
| Incidence | Mexico | 0.995113303 | 1.413541662 | 0.678172566 |
| Incidence | Micronesia (Federated States of) | 0.802779356 | 1.1866343 | 0.538337248 |
| Incidence | Monaco | 2.061265501 | 3.198644885 | 1.279776046 |
| Incidence | Mongolia | 1.048339568 | 1.548008643 | 0.711553391 |
| Incidence | Montenegro | 2.247305911 | 3.007398332 | 1.610046837 |
| Incidence | Morocco | 2.751328363 | 3.993032322 | 1.885993988 |
| Incidence | Mozambique | 1.907272605 | 2.675956203 | 1.360503005 |
| Incidence | Myanmar | 1.483960929 | 2.100919428 | 1.049954691 |
| Incidence | Namibia | 2.451663476 | 3.529465961 | 1.773948405 |
| Incidence | Nauru | 2.674028401 | 3.852763984 | 1.861920236 |
| Incidence | Nepal | 1.787668225 | 2.580254991 | 1.190454763 |
| Incidence | Netherlands | 1.469601851 | 2.113753405 | 1.03871495 |
| Incidence | New Zealand | 1.766675831 | 2.640734544 | 1.165720282 |
| Incidence | Nicaragua | 3.608283971 | 5.265258042 | 2.389537445 |
| Incidence | Niger | 0.905093133 | 1.355358674 | 0.601880129 |
| Incidence | Nigeria | 3.195035204 | 4.905486899 | 2.074249141 |
| Incidence | Niue | 1.742587394 | 2.519597452 | 1.181817095 |
| Incidence | North Macedonia | 6.844558649 | 10.78308217 | 4.097528188 |
| Incidence | Northern Mariana Islands | 2.348910009 | 2.938885053 | 1.833026648 |
| Incidence | Norway | 1.024366813 | 1.487416304 | 0.705156116 |
| Incidence | Oman | 3.283980993 | 4.825433076 | 2.187566637 |
| Incidence | Pakistan | 3.197543983 | 4.573229785 | 2.23813098 |
| Incidence | Palau | 1.732050709 | 2.403229244 | 1.287485882 |
| Incidence | Palestine | 1.155983804 | 1.728819226 | 0.768199635 |
| Incidence | Panama | 2.34407859 | 3.428407689 | 1.606860171 |
| Incidence | Papua New Guinea | 1.139168229 | 1.644562268 | 0.800595961 |
| Incidence | Paraguay | 3.673857402 | 5.291906153 | 2.522030145 |
| Incidence | Peru | 7.074218588 | 9.997266239 | 4.80537015 |
| Incidence | Philippines | 4.016344495 | 5.919889454 | 2.729817198 |
| Incidence | Poland | 4.712567151 | 6.775324846 | 3.194301623 |
| Incidence | Portugal | 3.004511174 | 4.447670319 | 1.977610004 |
| Incidence | Puerto Rico | 1.300773159 | 1.865108984 | 0.906084728 |
| Incidence | Qatar | 2.528784008 | 3.523062533 | 1.812208381 |
| Incidence | Republic of Korea | 4.414344501 | 6.243313272 | 3.108062884 |
| Incidence | Republic of Moldova | 1.51409533 | 2.147204269 | 1.052974368 |
| Incidence | Romania | 1.014759541 | 1.575000036 | 0.580862886 |
| Incidence | Russian Federation | 2.206499291 | 3.150633504 | 1.493399256 |
| Incidence | Rwanda | 2.469632915 | 3.788195989 | 1.582400089 |
| Incidence | Saint Kitts and Nevis | 2.126568496 | 3.125152574 | 1.439426843 |
| Incidence | Saint Lucia | 2.111352443 | 3.015612169 | 1.464758514 |
| Incidence | Saint Vincent and the Grenadines | 1.044386885 | 1.586838751 | 0.687464792 |
| Incidence | Samoa | 4.176707344 | 6.100601144 | 2.782539076 |
| Incidence | San Marino | 3.585008922 | 5.15678689 | 2.37875735 |
| Incidence | Sao Tome and Principe | 1.505393555 | 2.197646648 | 1.007313763 |
| Incidence | Saudi Arabia | 2.918156039 | 4.228069412 | 1.959652988 |
| Incidence | Senegal | 1.179704693 | 1.602747834 | 0.855720982 |
| Incidence | Serbia | 2.064143518 | 2.854214536 | 1.484540487 |
| Incidence | Seychelles | 4.126865626 | 6.21267782 | 2.674317368 |
| Incidence | Sierra Leone | 2.829029306 | 3.885464096 | 2.007083589 |
| Incidence | Singapore | 1.060673714 | 1.582659182 | 0.72523215 |
| Incidence | Slovakia | 2.859966292 | 4.043414829 | 2.022504149 |
| Incidence | Slovenia | 6.459724869 | 8.754952459 | 4.728606681 |
| Incidence | Solomon Islands | 1.993906735 | 2.769048712 | 1.39533352 |
| Incidence | Somalia | 1.144043514 | 1.702899309 | 0.766595515 |
| Incidence | South Africa | 1.128408552 | 1.730234965 | 0.698526099 |
| Incidence | South Sudan | 0.903818893 | 1.380219595 | 0.583818368 |
| Incidence | Spain | 4.760793866 | 6.921873418 | 3.232125821 |
| Incidence | Sri Lanka | 2.892815713 | 4.349158341 | 1.886394865 |
| Incidence | Sudan | 1.996146348 | 2.987483518 | 1.256120661 |
| Incidence | Suriname | 2.439361539 | 3.521918494 | 1.721226647 |
| Incidence | Sweden | 1.09747467 | 1.657909038 | 0.728055477 |
| Incidence | Switzerland | 1.864244591 | 2.720237887 | 1.224991461 |
| Incidence | Syrian Arab Republic | 4.408476841 | 6.733689598 | 2.862685808 |
| Incidence | Taiwan (Province of China) | 1.510812702 | 2.132127833 | 1.076543748 |
| Incidence | Tajikistan | 0.955392443 | 1.398182965 | 0.660614353 |
| Incidence | Thailand | 3.327323426 | 5.040735687 | 2.165158168 |
| Incidence | Timor-Leste | 2.341731893 | 3.791656286 | 1.393613432 |
| Incidence | Togo | 0.6687791 | 0.99902201 | 0.469167279 |
| Incidence | Tokelau | 2.101950328 | 2.784674585 | 1.578996232 |
| Incidence | Tonga | 1.115861324 | 1.602852351 | 0.799955866 |
| Incidence | Trinidad and Tobago | 4.399940885 | 6.627321347 | 2.858186777 |
| Incidence | Tunisia | 2.14253212 | 3.668221851 | 1.211698555 |
| Incidence | Turkey | 1.272932202 | 1.76805409 | 0.897402914 |
| Incidence | Turkmenistan | 1.366860095 | 1.872477054 | 0.988912467 |
| Incidence | Tuvalu | 3.006615274 | 4.692969669 | 1.927882484 |
| Incidence | Uganda | 1.724703303 | 2.482998997 | 1.184305746 |
| Incidence | Ukraine | 1.690499447 | 2.462668326 | 1.130373139 |
| Incidence | United Arab Emirates | 1.517796663 | 2.318690199 | 0.965624376 |
| Incidence | United Kingdom | 3.221805211 | 4.38856749 | 2.307954417 |
| Incidence | United Republic of Tanzania | 0.952506397 | 1.45983488 | 0.629273447 |
| Incidence | United States of America | 1.442487541 | 2.001293849 | 1.039640236 |
| Incidence | United States Virgin Islands | 1.232682908 | 1.731944303 | 0.869961223 |
| Incidence | Uruguay | 2.280461878 | 3.296683461 | 1.598272132 |
| Incidence | Uzbekistan | 1.145154661 | 1.58226359 | 0.813484829 |
| Incidence | Vanuatu | 2.246302461 | 3.051299823 | 1.631827152 |
| Incidence | Venezuela (Bolivarian Republic of) | 1.801700759 | 2.621058607 | 1.190699294 |
| Incidence | Viet Nam | 1.148776754 | 1.643941767 | 0.814176316 |
| Incidence | Yemen | 0.882636338 | 1.326963943 | 0.580762009 |
| Incidence | Zambia | 0.798995054 | 1.251344594 | 0.494572607 |
| Incidence | Zimbabwe | 2.20447412 | 2.917852835 | 1.663614149 |
| DALYs | China | 29.36713985 | 36.27909295 | 22.69527382 |
| DALYs | Afghanistan | 84.62451477 | 126.0879595 | 52.21684913 |
| DALYs | Albania | 24.85460752 | 35.65865093 | 17.09478729 |
| DALYs | Algeria | 37.35344686 | 52.21652796 | 25.69489444 |
| DALYs | American Samoa | 54.20864351 | 73.37637171 | 39.93661947 |
| DALYs | Andorra | 37.61923903 | 54.87405413 | 24.95385384 |
| DALYs | Angola | 85.96917775 | 126.3103956 | 55.16694644 |
| DALYs | Antigua and Barbuda | 60.86060448 | 82.25539712 | 43.26502315 |
| DALYs | Argentina | 42.78452986 | 59.52841581 | 30.03279862 |
| DALYs | Armenia | 60.92942595 | 81.9382266 | 44.33317977 |
| DALYs | Australia | 33.31645497 | 42.69177084 | 25.57587976 |
| DALYs | Austria | 35.92864838 | 48.86150913 | 25.86852969 |
| DALYs | Azerbaijan | 77.78151903 | 111.2344455 | 53.47848821 |
| DALYs | Bahamas | 78.80357524 | 110.8177686 | 54.21975773 |
| DALYs | Bahrain | 58.61073127 | 80.89752676 | 40.41267597 |
| DALYs | Bangladesh | 32.37072252 | 47.75623915 | 22.26081776 |
| DALYs | Barbados | 48.89559594 | 67.28681103 | 34.42215422 |
| DALYs | Belarus | 51.63137503 | 79.03515295 | 33.08268079 |
| DALYs | Belgium | 29.58740735 | 40.38752954 | 21.38910821 |
| DALYs | Belize | 107.9019132 | 149.3645481 | 75.54155541 |
| DALYs | Benin | 91.84822283 | 135.7800906 | 61.55061929 |
| DALYs | Bermuda | 29.05495113 | 40.78888846 | 20.53876391 |
| DALYs | Bhutan | 49.83967181 | 89.57185928 | 30.39158488 |
| DALYs | Bolivia (Plurinational State of) | 191.6757912 | 279.4674017 | 121.9923726 |
| DALYs | Bosnia and Herzegovina | 32.93752843 | 45.43062026 | 23.04077847 |
| DALYs | Botswana | 73.77180357 | 114.5530835 | 43.25346009 |
| DALYs | Brazil | 51.99057067 | 67.86959462 | 38.17032891 |
| DALYs | Brunei Darussalam | 26.83225385 | 35.47040409 | 20.04493223 |
| DALYs | Bulgaria | 44.92863056 | 65.41085868 | 29.62565452 |
| DALYs | Burkina Faso | 69.23964333 | 109.2057255 | 37.54140077 |
| DALYs | Burundi | 90.03592908 | 149.1469564 | 53.24591629 |
| DALYs | Cabo Verde | 86.67942709 | 114.3226817 | 63.25466526 |
| DALYs | Cambodia | 195.1376412 | 276.4802507 | 133.9037385 |
| DALYs | Cameroon | 69.15338357 | 112.6400973 | 40.34694837 |
| DALYs | Canada | 25.91084515 | 34.04713266 | 20.03287044 |
| DALYs | Central African Republic | 91.02256104 | 153.2461382 | 53.36830079 |
| DALYs | Chad | 96.38489515 | 139.4189848 | 64.07534144 |
| DALYs | Chile | 62.09180712 | 85.7330982 | 43.69267072 |
| DALYs | Colombia | 38.92247312 | 55.59171829 | 26.53963693 |
| DALYs | Comoros | 85.17107854 | 130.8114972 | 52.01232633 |
| DALYs | Congo | 80.44642437 | 122.1145908 | 50.35766897 |
| DALYs | Cook Islands | 53.45909494 | 72.21306266 | 38.35345393 |
| DALYs | Costa Rica | 103.7200556 | 148.5244652 | 69.8282428 |
| DALYs | Croatia | 30.16567604 | 44.08707839 | 20.47190086 |
| DALYs | Cuba | 55.21889286 | 80.12801018 | 37.23117558 |
| DALYs | Cyprus | 23.02201896 | 30.8046492 | 16.92311045 |
| DALYs | Czechia | 26.77018392 | 38.83876832 | 17.69422051 |
| DALYs | C么te d'Ivoire | 77.23750455 | 117.8208308 | 48.58421873 |
| DALYs | Democratic People's Republic of Korea | 49.75197972 | 71.93442089 | 31.95713465 |
| DALYs | Democratic Republic of the Congo | 69.38944762 | 104.6908315 | 43.14371636 |
| DALYs | Denmark | 30.14131696 | 41.45356331 | 21.27491068 |
| DALYs | Djibouti | 84.00478649 | 135.6045673 | 49.66554522 |
| DALYs | Dominica | 62.05972255 | 87.03454753 | 41.96453823 |
| DALYs | Dominican Republic | 138.8898813 | 205.4656547 | 86.16753354 |
| DALYs | Ecuador | 141.7171648 | 196.9125018 | 94.2442002 |
| DALYs | Egypt | 285.76104 | 425.290281 | 182.8790443 |
| DALYs | El Salvador | 142.4548117 | 210.6436615 | 92.92714689 |
| DALYs | Equatorial Guinea | 48.75575136 | 77.72665644 | 28.34021668 |
| DALYs | Eritrea | 132.1414219 | 203.4414958 | 83.49273105 |
| DALYs | Estonia | 47.20663795 | 72.49148438 | 29.87317414 |
| DALYs | Eswatini | 124.8476696 | 192.8946596 | 72.26129473 |
| DALYs | Ethiopia | 90.97446465 | 127.6151587 | 64.10797494 |
| DALYs | Fiji | 47.13733315 | 66.32900512 | 32.95191265 |
| DALYs | Finland | 40.16967453 | 56.18154939 | 27.88366822 |
| DALYs | France | 32.16979652 | 43.01913495 | 23.66656501 |
| DALYs | Gabon | 76.73455296 | 115.839604 | 47.75667765 |
| DALYs | Gambia | 160.6215069 | 227.4681982 | 108.2842568 |
| DALYs | Georgia | 63.29773782 | 92.82315793 | 42.74118152 |
| DALYs | Germany | 41.86603815 | 57.17509278 | 30.10450786 |
| DALYs | Ghana | 91.88824159 | 133.1111472 | 60.66394308 |
| DALYs | Greece | 21.17087396 | 28.09842177 | 15.77661359 |
| DALYs | Greenland | 42.66449656 | 61.43965644 | 28.80910553 |
| DALYs | Grenada | 74.21243883 | 102.1713654 | 52.69781236 |
| DALYs | Guam | 54.19443936 | 72.25174351 | 39.00270969 |
| DALYs | Guatemala | 268.0922012 | 389.0047029 | 179.0535816 |
| DALYs | Guinea | 143.5983606 | 204.3607595 | 97.42205036 |
| DALYs | Guinea-Bissau | 136.0661442 | 202.4364468 | 88.25390269 |
| DALYs | Guyana | 176.9558096 | 264.5121027 | 113.3002311 |
| DALYs | Haiti | 120.4524197 | 194.655541 | 59.66767491 |
| DALYs | Honduras | 283.3763168 | 421.4966909 | 174.6944231 |
| DALYs | Hungary | 31.97094271 | 46.8003615 | 20.95155283 |
| DALYs | Iceland | 16.11434743 | 21.30391177 | 11.91718958 |
| DALYs | India | 42.0077137 | 55.8175349 | 31.45959932 |
| DALYs | Indonesia | 120.2367394 | 161.3555582 | 86.22713507 |
| DALYs | Iran (Islamic Republic of) | 38.00679193 | 46.99189492 | 30.29767481 |
| DALYs | Iraq | 44.76469141 | 62.4386397 | 31.56571347 |
| DALYs | Ireland | 21.38542335 | 28.35178409 | 15.88774535 |
| DALYs | Israel | 23.05826239 | 30.07727038 | 17.54252143 |
| DALYs | Italy | 36.06864778 | 46.09122797 | 27.22008722 |
| DALYs | Jamaica | 33.54748012 | 47.67472016 | 22.77614867 |
| DALYs | Japan | 13.71680169 | 16.2790052 | 11.12930846 |
| DALYs | Jordan | 34.51654021 | 47.02109004 | 23.98356578 |
| DALYs | Kazakhstan | 97.82175501 | 137.430615 | 69.3797194 |
| DALYs | Kenya | 166.0928682 | 260.4741948 | 102.3597469 |
| DALYs | Kiribati | 123.0318892 | 195.9948842 | 70.9606969 |
| DALYs | Kuwait | 28.23124567 | 37.92544876 | 20.22814522 |
| DALYs | Kyrgyzstan | 96.60197556 | 137.1607723 | 65.53359759 |
| DALYs | Lao People's Democratic Republic | 75.91219276 | 112.4211786 | 47.94430309 |
| DALYs | Latvia | 45.03667323 | 67.64335695 | 29.63410512 |
| DALYs | Lebanon | 33.91030908 | 52.57013766 | 21.46816111 |
| DALYs | Lesotho | 108.8463598 | 170.9587326 | 64.82792843 |
| DALYs | Liberia | 98.21228806 | 145.9852525 | 63.16456543 |
| DALYs | Libya | 54.8654121 | 78.55737125 | 36.80384838 |
| DALYs | Lithuania | 66.00823136 | 98.97451826 | 42.25780651 |
| DALYs | Luxembourg | 31.41832511 | 42.9423889 | 21.92362937 |
| DALYs | Madagascar | 91.94433354 | 135.096928 | 59.37716901 |
| DALYs | Malawi | 109.5896637 | 157.6373321 | 73.22663289 |
| DALYs | Malaysia | 53.8137341 | 74.64060803 | 38.02080415 |
| DALYs | Maldives | 36.29958324 | 47.96895713 | 26.34814134 |
| DALYs | Mali | 99.90588198 | 146.4641711 | 68.21874371 |
| DALYs | Malta | 18.83443821 | 25.34328616 | 13.76732315 |
| DALYs | Marshall Islands | 91.30582009 | 147.7619763 | 53.11503343 |
| DALYs | Mauritania | 73.53043194 | 107.1786043 | 46.79012623 |
| DALYs | Mauritius | 48.81461676 | 69.12786306 | 33.34378134 |
| DALYs | Mexico | 220.8584835 | 292.0272895 | 158.9546283 |
| DALYs | Micronesia (Federated States of) | 94.85621656 | 161.1175538 | 50.06842932 |
| DALYs | Monaco | 42.12714776 | 58.335615 | 28.65163466 |
| DALYs | Mongolia | 311.4546864 | 426.9173648 | 217.7696922 |
| DALYs | Montenegro | 20.4598242 | 28.09978127 | 14.93520983 |
| DALYs | Morocco | 45.505879 | 64.82808909 | 30.58141406 |
| DALYs | Mozambique | 55.44993919 | 84.60129718 | 33.09604978 |
| DALYs | Myanmar | 123.5026227 | 181.2460421 | 79.36737141 |
| DALYs | Namibia | 61.58284566 | 95.16028806 | 36.31130134 |
| DALYs | Nauru | 98.83383471 | 159.5866168 | 53.72732542 |
| DALYs | Nepal | 57.10426503 | 85.50946272 | 36.76370586 |
| DALYs | Netherlands | 17.97130126 | 23.53031043 | 13.70685622 |
| DALYs | New Zealand | 25.41582754 | 30.61545813 | 20.78273775 |
| DALYs | Nicaragua | 182.4814199 | 253.2248581 | 126.5813279 |
| DALYs | Niger | 79.29341175 | 126.4288425 | 47.0284307 |
| DALYs | Nigeria | 93.32718705 | 148.2913885 | 56.37684209 |
| DALYs | Niue | 69.75544829 | 99.45802483 | 46.03623958 |
| DALYs | North Macedonia | 32.82368415 | 45.90999259 | 23.09835026 |
| DALYs | Northern Mariana Islands | 72.2659526 | 96.87158668 | 50.90160072 |
| DALYs | Norway | 15.34537886 | 19.43816338 | 12.02985018 |
| DALYs | Oman | 57.64920382 | 80.68401805 | 39.31918365 |
| DALYs | Pakistan | 45.24759312 | 67.8381349 | 28.98230859 |
| DALYs | Palau | 69.60919456 | 108.7686501 | 42.48173775 |
| DALYs | Palestine | 53.9390045 | 72.09851678 | 38.97438187 |
| DALYs | Panama | 62.60432242 | 92.37612129 | 41.95546059 |
| DALYs | Papua New Guinea | 27.28740599 | 41.47322833 | 16.60074468 |
| DALYs | Paraguay | 34.26578076 | 51.38953958 | 22.09286965 |
| DALYs | Peru | 105.4280719 | 158.3949532 | 68.03521623 |
| DALYs | Philippines | 59.46769101 | 78.7370248 | 43.24144733 |
| DALYs | Poland | 31.05152015 | 43.85546797 | 21.25378101 |
| DALYs | Portugal | 37.01787868 | 50.42300865 | 26.91134962 |
| DALYs | Puerto Rico | 79.97220031 | 117.498723 | 52.65267191 |
| DALYs | Qatar | 114.0249036 | 163.3052772 | 75.83218942 |
| DALYs | Republic of Korea | 27.84797181 | 36.8359388 | 21.23158193 |
| DALYs | Republic of Moldova | 133.9064759 | 194.2997159 | 88.95285134 |
| DALYs | Romania | 58.67477147 | 87.68922934 | 38.0706171 |
| DALYs | Russian Federation | 86.02978403 | 122.2787503 | 59.70078354 |
| DALYs | Rwanda | 100.5735837 | 141.4312129 | 68.8246453 |
| DALYs | Saint Kitts and Nevis | 86.23561772 | 121.7405979 | 57.13816608 |
| DALYs | Saint Lucia | 72.46556892 | 98.30290668 | 49.96850426 |
| DALYs | Saint Vincent and the Grenadines | 68.72426901 | 94.88535993 | 47.31895066 |
| DALYs | Samoa | 58.01763215 | 84.11084005 | 38.181106 |
| DALYs | San Marino | 38.8772667 | 64.63846588 | 22.44035957 |
| DALYs | Sao Tome and Principe | 101.3945063 | 151.2399266 | 61.31212754 |
| DALYs | Saudi Arabia | 76.7541128 | 103.8589779 | 52.61790471 |
| DALYs | Senegal | 72.54222922 | 111.3702625 | 45.91655005 |
| DALYs | Serbia | 23.61070634 | 33.70637277 | 16.35088577 |
| DALYs | Seychelles | 94.18653087 | 125.6643859 | 67.24671176 |
| DALYs | Sierra Leone | 74.70346914 | 116.7213154 | 47.29977969 |
| DALYs | Singapore | 11.75460262 | 15.94694182 | 8.561187172 |
| DALYs | Slovakia | 40.4992717 | 61.25434918 | 25.56346289 |
| DALYs | Slovenia | 34.55957251 | 49.86007135 | 22.76680206 |
| DALYs | Solomon Islands | 103.6241529 | 148.5583891 | 67.87041682 |
| DALYs | Somalia | 111.5815257 | 180.51814 | 68.31269446 |
| DALYs | South Africa | 51.07248764 | 63.03362943 | 40.96013332 |
| DALYs | South Sudan | 86.60533331 | 138.1300301 | 50.61082629 |
| DALYs | Spain | 25.98362458 | 34.73830553 | 19.47605106 |
| DALYs | Sri Lanka | 52.25438722 | 77.60537749 | 34.6435659 |
| DALYs | Sudan | 60.01227463 | 96.11769025 | 35.84038519 |
| DALYs | Suriname | 98.94058851 | 137.3127132 | 67.28513492 |
| DALYs | Sweden | 16.73434348 | 21.65711505 | 12.84344181 |
| DALYs | Switzerland | 21.42904419 | 28.34777364 | 15.90647953 |
| DALYs | Syrian Arab Republic | 56.93688192 | 81.98146318 | 38.30734138 |
| DALYs | Taiwan (Province of China) | 61.49861195 | 86.90002645 | 41.77422156 |
| DALYs | Tajikistan | 89.50036442 | 125.74234 | 62.03105066 |
| DALYs | Thailand | 123.9719719 | 180.665401 | 86.2548174 |
| DALYs | Timor-Leste | 73.77932926 | 116.2193163 | 43.27490365 |
| DALYs | Togo | 81.02108242 | 121.7943841 | 52.44988654 |
| DALYs | Tokelau | 57.25212863 | 82.96192662 | 37.83434952 |
| DALYs | Tonga | 126.1379961 | 177.3346564 | 89.82648781 |
| DALYs | Trinidad and Tobago | 55.46184728 | 82.14360166 | 36.06294549 |
| DALYs | Tunisia | 34.49831993 | 53.77194948 | 22.03742478 |
| DALYs | Turkey | 29.12988228 | 39.68139685 | 20.63975144 |
| DALYs | Turkmenistan | 140.9216048 | 204.9177279 | 95.48491392 |
| DALYs | Tuvalu | 75.75075964 | 118.6730523 | 47.0402279 |
| DALYs | Uganda | 87.85315605 | 121.7985305 | 57.98619073 |
| DALYs | Ukraine | 118.0300403 | 169.6633326 | 77.04265893 |
| DALYs | United Arab Emirates | 47.36363647 | 72.79908542 | 27.53398272 |
| DALYs | United Kingdom | 34.41644608 | 45.47386809 | 26.0323147 |
| DALYs | United Republic of Tanzania | 77.69396626 | 115.8264432 | 48.83275078 |
| DALYs | United States of America | 45.07039208 | 59.31070191 | 34.06401866 |
| DALYs | United States Virgin Islands | 91.74583367 | 130.0071598 | 63.11072525 |
| DALYs | Uruguay | 27.26465085 | 36.67315929 | 19.71623984 |
| DALYs | Uzbekistan | 140.5947355 | 195.8598708 | 97.51700012 |
| DALYs | Vanuatu | 84.14312768 | 127.1713003 | 50.57989053 |
| DALYs | Venezuela (Bolivarian Republic of) | 82.59300245 | 125.0974766 | 52.18463443 |
| DALYs | Viet Nam | 62.93322215 | 91.05910505 | 41.8309981 |
| DALYs | Yemen | 53.13786964 | 81.68293016 | 32.63537069 |
| DALYs | Zambia | 142.012526 | 208.0718453 | 93.88062497 |
| DALYs | Zimbabwe | 97.33691215 | 145.2231401 | 61.7458377 |
| Deaths | Afghanistan | 3.681749495 | 5.232886814 | 2.397786813 |
| Deaths | Albania | 1.14987645 | 1.619257768 | 0.771284021 |
| Deaths | Algeria | 1.976854948 | 2.767973598 | 1.349626191 |
| Deaths | American Samoa | 2.137279487 | 2.787527676 | 1.589838216 |
| Deaths | Andorra | 1.612409754 | 2.29061417 | 1.102630094 |
| Deaths | Angola | 3.466461674 | 5.082925958 | 2.289926924 |
| Deaths | Antigua and Barbuda | 2.678920442 | 3.553346327 | 1.933434786 |
| Deaths | Argentina | 1.783407524 | 2.407600064 | 1.260114054 |
| Deaths | Armenia | 3.135536377 | 4.255113579 | 2.218719122 |
| Deaths | Australia | 1.355624073 | 1.706916196 | 1.062231185 |
| Deaths | Austria | 1.429546301 | 1.91715451 | 1.034914771 |
| Deaths | Azerbaijan | 3.915715308 | 5.92585817 | 2.525879472 |
| Deaths | Bahamas | 2.9551606 | 4.019851322 | 2.07283421 |
| Deaths | Bahrain | 3.409608446 | 4.806141003 | 2.295221234 |
| Deaths | Bangladesh | 1.341904543 | 1.936432779 | 0.923537675 |
| Deaths | Barbados | 2.020091537 | 2.716604865 | 1.444590795 |
| Deaths | Belarus | 1.618023541 | 2.401883149 | 1.06055637 |
| Deaths | Belgium | 1.239776555 | 1.633081969 | 0.905908642 |
| Deaths | Belize | 4.083054792 | 5.434657277 | 2.911864453 |
| Deaths | Benin | 3.767915302 | 5.352903017 | 2.564527025 |
| Deaths | Bermuda | 1.264766293 | 1.704393729 | 0.91476307 |
| Deaths | Bhutan | 2.056107154 | 3.518830368 | 1.302605979 |
| Deaths | Bolivia (Plurinational State of) | 8.702488848 | 12.3534559 | 5.740649771 |
| Deaths | Bosnia and Herzegovina | 1.46501083 | 1.987211261 | 1.039403935 |
| Deaths | Botswana | 2.784789859 | 4.098515424 | 1.747353696 |
| Deaths | Brazil | 2.011802272 | 2.577605167 | 1.517843897 |
| Deaths | Brunei Darussalam | 1.196102661 | 1.58592264 | 0.887853185 |
| Deaths | Bulgaria | 1.564792405 | 2.240224977 | 1.060037878 |
| Deaths | Burkina Faso | 3.049434828 | 4.813654136 | 1.647929074 |
| Deaths | Burundi | 3.703996539 | 5.907127027 | 2.188904952 |
| Deaths | Cabo Verde | 3.814658497 | 5.019249549 | 2.819642398 |
| Deaths | Cambodia | 7.789749736 | 10.69033191 | 5.389344265 |
| Deaths | Cameroon | 2.763657039 | 4.453805277 | 1.654124457 |
| Deaths | Canada | 1.148412607 | 1.461143206 | 0.884275381 |
| Deaths | Central African Republic | 3.428154979 | 5.462673812 | 2.143450125 |
| Deaths | Chad | 3.839161752 | 5.508845122 | 2.62958544 |
| Deaths | Chile | 2.538722421 | 3.435696743 | 1.820155306 |
| Deaths | China | 1.24565554 | 1.529508416 | 0.976145915 |
| Deaths | Colombia | 1.800481242 | 2.520772749 | 1.227845535 |
| Deaths | Comoros | 3.648653669 | 5.464025423 | 2.193832379 |
| Deaths | Congo | 3.276493296 | 4.97328216 | 2.097684928 |
| Deaths | Cook Islands | 2.193801477 | 2.887175484 | 1.627119089 |
| Deaths | Costa Rica | 4.303563245 | 5.906050863 | 2.970961688 |
| Deaths | Croatia | 1.220824568 | 1.7205422 | 0.849843406 |
| Deaths | Cuba | 2.150527163 | 3.064919948 | 1.496725281 |
| Deaths | Cyprus | 1.2185414 | 1.653291942 | 0.875388428 |
| Deaths | Czechia | 0.979458008 | 1.384394095 | 0.674516278 |
| Deaths | C么te d'Ivoire | 3.176937061 | 4.827044844 | 2.03091372 |
| Deaths | Democratic People's Republic of Korea | 1.952699085 | 2.73607402 | 1.338132627 |
| Deaths | Democratic Republic of the Congo | 2.660963669 | 3.991050063 | 1.696102841 |
| Deaths | Denmark | 1.157294044 | 1.538637607 | 0.835379777 |
| Deaths | Djibouti | 3.632469524 | 5.698286413 | 2.180328281 |
| Deaths | Dominica | 2.541441827 | 3.464939615 | 1.766947556 |
| Deaths | Dominican Republic | 6.062049386 | 8.675534897 | 4.046074219 |
| Deaths | Ecuador | 6.639032302 | 9.079824195 | 4.511952784 |
| Deaths | Egypt | 15.97867884 | 24.3260753 | 10.06904928 |
| Deaths | El Salvador | 5.445313499 | 7.836276477 | 3.72521158 |
| Deaths | Equatorial Guinea | 2.070537944 | 3.226946562 | 1.250799458 |
| Deaths | Eritrea | 5.529435926 | 8.113375363 | 3.570035975 |
| Deaths | Estonia | 1.518868524 | 2.22679593 | 0.980646421 |
| Deaths | Eswatini | 4.81341449 | 7.022819864 | 2.940448949 |
| Deaths | Ethiopia | 4.11116561 | 5.71888766 | 2.876482633 |
| Deaths | Fiji | 1.889943212 | 2.566023209 | 1.359191985 |
| Deaths | Finland | 1.427802761 | 1.951326883 | 1.01855829 |
| Deaths | France | 1.311148565 | 1.718172179 | 0.981620437 |
| Deaths | Gabon | 3.193331917 | 4.883190879 | 1.945318008 |
| Deaths | Gambia | 6.624992886 | 9.20555814 | 4.552292681 |
| Deaths | Georgia | 2.211976799 | 3.065157712 | 1.553657725 |
| Deaths | Germany | 1.689813792 | 2.228171433 | 1.265610701 |
| Deaths | Ghana | 3.791531633 | 5.292976022 | 2.542576931 |
| Deaths | Greece | 0.962243833 | 1.231747404 | 0.722892014 |
| Deaths | Greenland | 1.526993987 | 2.115035815 | 1.082905481 |
| Deaths | Grenada | 2.930170858 | 3.895385293 | 2.134935953 |
| Deaths | Guam | 2.047096939 | 2.691149728 | 1.522013282 |
| Deaths | Guatemala | 9.663840625 | 13.38065222 | 6.743876203 |
| Deaths | Guinea | 5.956278328 | 8.426963314 | 4.107008209 |
| Deaths | Guinea-Bissau | 5.305703275 | 7.681542713 | 3.527982741 |
| Deaths | Guyana | 6.224681236 | 8.907813342 | 4.191450948 |
| Deaths | Haiti | 4.665799505 | 7.236216396 | 2.345954854 |
| Deaths | Honduras | 12.42854275 | 18.23891773 | 7.9226691 |
| Deaths | Hungary | 1.15224746 | 1.657139212 | 0.770793901 |
| Deaths | Iceland | 0.642281022 | 0.829234134 | 0.480367536 |
| Deaths | India | 1.596834132 | 2.06869284 | 1.208952045 |
| Deaths | Indonesia | 5.738897566 | 7.747231622 | 4.111927744 |
| Deaths | Iran (Islamic Republic of) | 1.924848524 | 2.418807893 | 1.513622982 |
| Deaths | Iraq | 2.028077649 | 2.747941652 | 1.452880623 |
| Deaths | Ireland | 0.877214142 | 1.128759356 | 0.656624864 |
| Deaths | Israel | 1.157017321 | 1.548250575 | 0.857026808 |
| Deaths | Italy | 1.685167461 | 2.134531955 | 1.267269753 |
| Deaths | Jamaica | 1.384331902 | 1.902422289 | 0.966800365 |
| Deaths | Japan | 0.682199429 | 0.819412169 | 0.549988711 |
| Deaths | Jordan | 1.790410464 | 2.450120479 | 1.232218048 |
| Deaths | Kazakhstan | 3.674420334 | 4.960556413 | 2.669962309 |
| Deaths | Kenya | 7.408353457 | 11.48564683 | 4.573439595 |
| Deaths | Kiribati | 4.557355425 | 6.747936536 | 2.950747905 |
| Deaths | Kuwait | 1.409812533 | 1.892133248 | 1.018107521 |
| Deaths | Kyrgyzstan | 3.258939015 | 4.479467853 | 2.307135822 |
| Deaths | Lao People's Democratic Republic | 3.032151328 | 4.36227263 | 2.032811176 |
| Deaths | Latvia | 1.412697239 | 2.04751205 | 0.960722211 |
| Deaths | Lebanon | 1.721393918 | 2.748444853 | 1.07824203 |
| Deaths | Lesotho | 4.191295705 | 6.236310331 | 2.672548774 |
| Deaths | Liberia | 4.130858496 | 5.888474194 | 2.765906605 |
| Deaths | Libya | 2.605906501 | 3.760007556 | 1.725424702 |
| Deaths | Lithuania | 1.953977053 | 2.873264644 | 1.286783134 |
| Deaths | Luxembourg | 1.275970801 | 1.706990684 | 0.911082918 |
| Deaths | Madagascar | 3.812102446 | 5.4334205 | 2.506165091 |
| Deaths | Malawi | 4.710663751 | 6.652824435 | 3.142857912 |
| Deaths | Malaysia | 2.491362759 | 3.37498953 | 1.752472183 |
| Deaths | Maldives | 1.755691645 | 2.348414653 | 1.262746174 |
| Deaths | Mali | 3.929372269 | 5.548802319 | 2.745769306 |
| Deaths | Malta | 0.74028839 | 0.967579144 | 0.544399789 |
| Deaths | Marshall Islands | 3.369277578 | 5.278779954 | 2.059132656 |
| Deaths | Mauritania | 3.149403973 | 4.361419919 | 2.135980532 |
| Deaths | Mauritius | 1.793721735 | 2.448490356 | 1.265211866 |
| Deaths | Mexico | 8.549327166 | 11.08574172 | 6.260990427 |
| Deaths | Micronesia (Federated States of) | 3.588352299 | 5.67876722 | 2.062842222 |
| Deaths | Monaco | 1.734991082 | 2.320941251 | 1.198348636 |
| Deaths | Mongolia | 15.30998906 | 20.8016744 | 10.92330223 |
| Deaths | Montenegro | 0.832951909 | 1.122176622 | 0.604505624 |
| Deaths | Morocco | 2.32818402 | 3.284060468 | 1.567392643 |
| Deaths | Mozambique | 2.506834349 | 3.792912623 | 1.498753401 |
| Deaths | Myanmar | 3.793446738 | 5.327901532 | 2.615331234 |
| Deaths | Namibia | 2.454759096 | 3.607730836 | 1.515053866 |
| Deaths | Nauru | 3.697502573 | 5.597645294 | 2.159191005 |
| Deaths | Nepal | 2.383013045 | 3.494386611 | 1.551582113 |
| Deaths | Netherlands | 0.889102802 | 1.149064562 | 0.667199493 |
| Deaths | New Zealand | 1.105565 | 1.333432635 | 0.906657936 |
| Deaths | Nicaragua | 7.805552784 | 10.59417884 | 5.460162329 |
| Deaths | Niger | 3.151020877 | 4.903084216 | 1.855487494 |
| Deaths | Nigeria | 4.080089997 | 6.265767481 | 2.547297767 |
| Deaths | Niue | 2.76820333 | 3.800171093 | 1.910078768 |
| Deaths | North Macedonia | 1.436962379 | 1.970088242 | 1.032442117 |
| Deaths | Northern Mariana Islands | 2.967309176 | 3.937340226 | 2.168831128 |
| Deaths | Norway | 0.673365483 | 0.855430244 | 0.525535315 |
| Deaths | Oman | 2.907818835 | 3.957401965 | 2.056760481 |
| Deaths | Pakistan | 1.800897392 | 2.819713274 | 1.154896049 |
| Deaths | Palau | 2.610956551 | 3.910974125 | 1.671938132 |
| Deaths | Palestine | 2.838086357 | 3.869906872 | 2.020720518 |
| Deaths | Panama | 2.760985678 | 3.911372441 | 1.908619414 |
| Deaths | Papua New Guinea | 0.907111283 | 1.321898608 | 0.581045672 |
| Deaths | Paraguay | 1.391289548 | 2.024084818 | 0.926827569 |
| Deaths | Peru | 4.516726097 | 6.65710857 | 2.926990545 |
| Deaths | Philippines | 2.383144037 | 3.126588245 | 1.75264693 |
| Deaths | Poland | 1.067275392 | 1.461227126 | 0.759712628 |
| Deaths | Portugal | 1.421799666 | 1.870592115 | 1.058631657 |
| Deaths | Puerto Rico | 3.265933509 | 4.666732581 | 2.178311686 |
| Deaths | Qatar | 7.221695615 | 10.61706181 | 4.810376877 |
| Deaths | Republic of Korea | 1.348202528 | 1.792680162 | 1.000313638 |
| Deaths | Republic of Moldova | 4.682246294 | 6.616180526 | 3.141660631 |
| Deaths | Romania | 2.162939667 | 3.150385264 | 1.426899821 |
| Deaths | Russian Federation | 2.549779172 | 3.455659892 | 1.825890238 |
| Deaths | Rwanda | 4.29377883 | 5.968703557 | 2.954880159 |
| Deaths | Saint Kitts and Nevis | 3.548443002 | 4.862452906 | 2.4880822 |
| Deaths | Saint Lucia | 2.911702629 | 3.899081145 | 2.069748872 |
| Deaths | Saint Vincent and the Grenadines | 2.600657332 | 3.433207083 | 1.867123155 |
| Deaths | Samoa | 2.213846382 | 3.069212552 | 1.540024394 |
| Deaths | San Marino | 1.813225843 | 2.825678531 | 1.079791889 |
| Deaths | Sao Tome and Principe | 4.29833971 | 6.528544787 | 2.591612106 |
| Deaths | Saudi Arabia | 4.331620866 | 6.115230406 | 2.924482938 |
| Deaths | Senegal | 2.96679853 | 4.49230957 | 1.856596882 |
| Deaths | Serbia | 0.979861175 | 1.352774402 | 0.698841431 |
| Deaths | Seychelles | 3.748155152 | 4.944435225 | 2.745758613 |
| Deaths | Sierra Leone | 3.004996052 | 4.595193002 | 1.938406374 |
| Deaths | Singapore | 0.616844283 | 0.845285041 | 0.438311204 |
| Deaths | Slovakia | 1.411317368 | 2.0517687 | 0.939416626 |
| Deaths | Slovenia | 1.379212997 | 1.957895934 | 0.92584309 |
| Deaths | Solomon Islands | 3.187493009 | 4.42738668 | 2.217316127 |
| Deaths | Somalia | 4.64105828 | 7.299519715 | 2.86620366 |
| Deaths | South Africa | 2.078095127 | 2.541215887 | 1.663232059 |
| Deaths | South Sudan | 3.786723037 | 5.846011022 | 2.282168497 |
| Deaths | Spain | 1.16982598 | 1.520717409 | 0.881665383 |
| Deaths | Sri Lanka | 2.190041852 | 3.093436224 | 1.447067069 |
| Deaths | Sudan | 2.93500478 | 4.840523911 | 1.779546266 |
| Deaths | Suriname | 3.973406107 | 5.416350419 | 2.773081665 |
| Deaths | Sweden | 0.740391432 | 0.946681464 | 0.562271239 |
| Deaths | Switzerland | 0.943792266 | 1.226095029 | 0.71341458 |
| Deaths | Syrian Arab Republic | 2.965786206 | 4.21585728 | 1.934543208 |
| Deaths | Taiwan (Province of China) | 2.596010211 | 3.562644761 | 1.806200446 |
| Deaths | Tajikistan | 3.499472962 | 4.920587323 | 2.410009331 |
| Deaths | Thailand | 4.921813186 | 6.916264067 | 3.416640337 |
| Deaths | Timor-Leste | 3.048382995 | 4.699614145 | 1.880162128 |
| Deaths | Togo | 3.295941481 | 4.886634553 | 2.136194878 |
| Deaths | Tokelau | 2.250821847 | 3.156192788 | 1.551914147 |
| Deaths | Tonga | 5.038589365 | 6.759342188 | 3.736976871 |
| Deaths | Trinidad and Tobago | 2.064743747 | 2.947711073 | 1.380316627 |
| Deaths | Tunisia | 1.740874289 | 2.607089646 | 1.094923135 |
| Deaths | Turkey | 1.517447398 | 2.096465736 | 1.058538535 |
| Deaths | Turkmenistan | 4.387917782 | 6.166024946 | 3.002930426 |
| Deaths | Tuvalu | 2.854769604 | 4.226634721 | 1.858636202 |
| Deaths | Uganda | 3.787192954 | 5.19164741 | 2.543399828 |
| Deaths | Ukraine | 3.170179468 | 4.486108096 | 2.125897341 |
| Deaths | United Arab Emirates | 2.222776844 | 3.358004362 | 1.293642775 |
| Deaths | United Kingdom | 1.287425814 | 1.615793852 | 1.013427613 |
| Deaths | United Republic of Tanzania | 3.300020888 | 4.872773033 | 2.029191571 |
| Deaths | United States of America | 1.709152588 | 2.182695379 | 1.310252966 |
| Deaths | United States Virgin Islands | 3.749689435 | 5.044176658 | 2.678111659 |
| Deaths | Uruguay | 1.184252834 | 1.541540368 | 0.871772029 |
| Deaths | Uzbekistan | 5.096455139 | 6.959757282 | 3.610731359 |
| Deaths | Vanuatu | 3.082012375 | 4.592806365 | 1.911123568 |
| Deaths | Venezuela (Bolivarian Republic of) | 3.262136776 | 4.775883225 | 2.126451403 |
| Deaths | Viet Nam | 2.812556449 | 3.982160841 | 1.874835856 |
| Deaths | Yemen | 2.517948116 | 3.946045502 | 1.530220612 |
| Deaths | Zambia | 5.889908052 | 8.376064957 | 3.940974538 |
| Deaths | Zimbabwe | 3.970370039 | 5.831141793 | 2.533012751 |

Supplementary Table 4. Age-standardized prevalence, incidence, DALYs, and Death rate of  NAFLLD globally and for 21 GBD regions, 1990-2019.

| Measure | Location | Year | Rate | 95% Upper UI | 95% Lower UI |
| --- | --- | --- | --- | --- | --- |
| Prevalence | Andean Latin America | 1990 | 11762.73029 | 13129.24414 | 10558.43253 |
| Prevalence | Andean Latin America | 1991 | 11839.51797 | 13205.36181 | 10643.63674 |
| Prevalence | Andean Latin America | 1992 | 11918.33101 | 13273.60793 | 10718.53249 |
| Prevalence | Andean Latin America | 1993 | 11997.06092 | 13320.44801 | 10795.62721 |
| Prevalence | Andean Latin America | 1994 | 12074.09389 | 13393.567 | 10867.1681 |
| Prevalence | Andean Latin America | 1995 | 12145.43014 | 13450.55617 | 10936.81786 |
| Prevalence | Andean Latin America | 1996 | 12226.00228 | 13539.81214 | 11006.43263 |
| Prevalence | Andean Latin America | 1997 | 12322.2657 | 13649.05767 | 11088.64155 |
| Prevalence | Andean Latin America | 1998 | 12423.87272 | 13783.43802 | 11205.35362 |
| Prevalence | Andean Latin America | 1999 | 12520.19732 | 13876.73095 | 11297.3458 |
| Prevalence | Andean Latin America | 2000 | 12600.18318 | 13959.83983 | 11362.12903 |
| Prevalence | Andean Latin America | 2001 | 12669.90483 | 14035.91939 | 11423.32657 |
| Prevalence | Andean Latin America | 2002 | 12738.08324 | 14113.3216 | 11483.79842 |
| Prevalence | Andean Latin America | 2003 | 12801.63818 | 14187.94971 | 11538.40197 |
| Prevalence | Andean Latin America | 2004 | 12860.3064 | 14257.96921 | 11603.68532 |
| Prevalence | Andean Latin America | 2005 | 12911.6082 | 14322.85113 | 11639.07236 |
| Prevalence | Andean Latin America | 2006 | 12957.58562 | 14360.06414 | 11687.22136 |
| Prevalence | Andean Latin America | 2007 | 13001.45631 | 14399.05676 | 11733.4989 |
| Prevalence | Andean Latin America | 2008 | 13044.35743 | 14437.6774 | 11776.93737 |
| Prevalence | Andean Latin America | 2009 | 13087.69386 | 14481.91582 | 11820.33842 |
| Prevalence | Andean Latin America | 2010 | 13130.85297 | 14513.04141 | 11853.95444 |
| Prevalence | Andean Latin America | 2011 | 13181.45117 | 14561.0218 | 11913.01385 |
| Prevalence | Andean Latin America | 2012 | 13241.30516 | 14607.37165 | 11988.8452 |
| Prevalence | Andean Latin America | 2013 | 13300.2165 | 14660.58757 | 12035.08918 |
| Prevalence | Andean Latin America | 2014 | 13359.19482 | 14720.61926 | 12089.73337 |
| Prevalence | Andean Latin America | 2015 | 13414.53729 | 14797.37063 | 12126.72585 |
| Prevalence | Andean Latin America | 2016 | 13491.11794 | 14871.54185 | 12196.94943 |
| Prevalence | Andean Latin America | 2017 | 13564.43486 | 14955.81756 | 12269.57375 |
| Prevalence | Andean Latin America | 2018 | 13624.29329 | 15024.8954 | 12327.60241 |
| Prevalence | Andean Latin America | 2019 | 13690.02064 | 15086.38958 | 12377.03993 |
| Prevalence | Australasia | 1990 | 7267.296757 | 8167.635128 | 6458.280499 |
| Prevalence | Australasia | 1991 | 7351.996726 | 8267.799223 | 6548.541258 |
| Prevalence | Australasia | 1992 | 7441.031918 | 8384.098806 | 6640.115492 |
| Prevalence | Australasia | 1993 | 7533.942151 | 8514.018388 | 6738.142873 |
| Prevalence | Australasia | 1994 | 7630.045231 | 8616.173144 | 6827.187685 |
| Prevalence | Australasia | 1995 | 7728.76009 | 8718.804588 | 6904.205531 |
| Prevalence | Australasia | 1996 | 7836.215513 | 8840.771171 | 7001.72571 |
| Prevalence | Australasia | 1997 | 7953.486662 | 8938.328346 | 7100.20934 |
| Prevalence | Australasia | 1998 | 8073.786943 | 9040.20483 | 7203.390111 |
| Prevalence | Australasia | 1999 | 8189.742723 | 9139.055147 | 7302.131994 |
| Prevalence | Australasia | 2000 | 8294.444328 | 9235.550611 | 7406.561407 |
| Prevalence | Australasia | 2001 | 8390.825857 | 9361.759323 | 7475.754678 |
| Prevalence | Australasia | 2002 | 8484.761711 | 9492.957475 | 7579.832495 |
| Prevalence | Australasia | 2003 | 8574.516197 | 9589.66461 | 7696.457013 |
| Prevalence | Australasia | 2004 | 8658.345312 | 9695.859886 | 7767.207493 |
| Prevalence | Australasia | 2005 | 8734.593572 | 9770.315867 | 7844.799421 |
| Prevalence | Australasia | 2006 | 8807.547777 | 9839.705869 | 7890.344457 |
| Prevalence | Australasia | 2007 | 8880.517631 | 9905.26798 | 7975.257012 |
| Prevalence | Australasia | 2008 | 8950.219341 | 9985.760527 | 8038.515964 |
| Prevalence | Australasia | 2009 | 9013.319144 | 10038.42872 | 8089.31559 |
| Prevalence | Australasia | 2010 | 9066.609654 | 10064.66808 | 8109.620839 |
| Prevalence | Australasia | 2011 | 9115.246616 | 10121.47855 | 8163.513482 |
| Prevalence | Australasia | 2012 | 9164.355343 | 10185.14127 | 8214.824054 |
| Prevalence | Australasia | 2013 | 9210.569811 | 10250.74862 | 8255.864092 |
| Prevalence | Australasia | 2014 | 9250.657754 | 10308.85068 | 8292.626379 |
| Prevalence | Australasia | 2015 | 9281.297053 | 10343.10057 | 8316.074312 |
| Prevalence | Australasia | 2016 | 9306.22818 | 10309.12067 | 8355.822291 |
| Prevalence | Australasia | 2017 | 9332.520182 | 10332.72592 | 8370.304532 |
| Prevalence | Australasia | 2018 | 9377.433267 | 10405.96139 | 8423.037779 |
| Prevalence | Australasia | 2019 | 9444.779207 | 10478.72197 | 8455.935448 |
| Prevalence | Caribbean | 1990 | 14537.30149 | 16203.49462 | 13031.15445 |
| Prevalence | Caribbean | 1991 | 14600.93668 | 16259.32624 | 13100.49686 |
| Prevalence | Caribbean | 1992 | 14662.68433 | 16326.88711 | 13152.72114 |
| Prevalence | Caribbean | 1993 | 14720.30959 | 16388.1201 | 13223.26744 |
| Prevalence | Caribbean | 1994 | 14771.27694 | 16456.73074 | 13280.62395 |
| Prevalence | Caribbean | 1995 | 14813.60627 | 16483.69858 | 13331.40126 |
| Prevalence | Caribbean | 1996 | 14850.44404 | 16527.74472 | 13354.3005 |
| Prevalence | Caribbean | 1997 | 14888.67777 | 16571.99016 | 13396.80798 |
| Prevalence | Caribbean | 1998 | 14929.41891 | 16619.38439 | 13454.15888 |
| Prevalence | Caribbean | 1999 | 14973.79388 | 16669.39173 | 13463.07907 |
| Prevalence | Caribbean | 2000 | 15023.32822 | 16685.78917 | 13483.24116 |
| Prevalence | Caribbean | 2001 | 15102.34879 | 16782.37962 | 13566.94849 |
| Prevalence | Caribbean | 2002 | 15219.98937 | 16881.69633 | 13685.30212 |
| Prevalence | Caribbean | 2003 | 15352.45163 | 17027.04992 | 13817.85831 |
| Prevalence | Caribbean | 2004 | 15476.21257 | 17183.36997 | 13941.27955 |
| Prevalence | Caribbean | 2005 | 15567.92705 | 17292.86121 | 13999.87427 |
| Prevalence | Caribbean | 2006 | 15632.66811 | 17340.56716 | 14059.83652 |
| Prevalence | Caribbean | 2007 | 15691.70195 | 17390.5913 | 14126.04852 |
| Prevalence | Caribbean | 2008 | 15744.37065 | 17441.60216 | 14178.46573 |
| Prevalence | Caribbean | 2009 | 15789.68947 | 17500.04699 | 14237.83716 |
| Prevalence | Caribbean | 2010 | 15832.38132 | 17557.56941 | 14284.89185 |
| Prevalence | Caribbean | 2011 | 15873.5415 | 17583.85936 | 14325.09713 |
| Prevalence | Caribbean | 2012 | 15913.36147 | 17613.29834 | 14361.9928 |
| Prevalence | Caribbean | 2013 | 15954.61092 | 17664.96122 | 14397.97734 |
| Prevalence | Caribbean | 2014 | 15993.92643 | 17698.24255 | 14424.35671 |
| Prevalence | Caribbean | 2015 | 16028.16803 | 17719.08863 | 14443.41488 |
| Prevalence | Caribbean | 2016 | 16078.78402 | 17790.71832 | 14483.1347 |
| Prevalence | Caribbean | 2017 | 16119.20151 | 17840.46451 | 14513.59597 |
| Prevalence | Caribbean | 2018 | 16141.10449 | 17839.61833 | 14548.28856 |
| Prevalence | Caribbean | 2019 | 16169.78708 | 17866.81139 | 14591.34159 |
| Prevalence | Central Asia | 1990 | 12301.68243 | 13710.27863 | 11056.50667 |
| Prevalence | Central Asia | 1991 | 12340.23227 | 13746.99786 | 11085.32748 |
| Prevalence | Central Asia | 1992 | 12377.88597 | 13782.68188 | 11113.1059 |
| Prevalence | Central Asia | 1993 | 12414.44074 | 13824.74072 | 11143.36251 |
| Prevalence | Central Asia | 1994 | 12448.22214 | 13851.1938 | 11178.91711 |
| Prevalence | Central Asia | 1995 | 12476.7197 | 13885.37092 | 11222.27469 |
| Prevalence | Central Asia | 1996 | 12507.18067 | 13925.72118 | 11244.50149 |
| Prevalence | Central Asia | 1997 | 12544.2667 | 13968.63521 | 11277.68967 |
| Prevalence | Central Asia | 1998 | 12584.87537 | 14023.92372 | 11315.60849 |
| Prevalence | Central Asia | 1999 | 12625.50476 | 14074.09658 | 11347.21605 |
| Prevalence | Central Asia | 2000 | 12661.34668 | 14118.51971 | 11390.71542 |
| Prevalence | Central Asia | 2001 | 12696.00779 | 14133.7947 | 11417.56404 |
| Prevalence | Central Asia | 2002 | 12733.62162 | 14169.44843 | 11446.99839 |
| Prevalence | Central Asia | 2003 | 12775.3858 | 14228.82946 | 11476.5422 |
| Prevalence | Central Asia | 2004 | 12822.22668 | 14277.35685 | 11526.20183 |
| Prevalence | Central Asia | 2005 | 12875.0049 | 14352.318 | 11570.11519 |
| Prevalence | Central Asia | 2006 | 12947.51013 | 14401.45297 | 11627.96256 |
| Prevalence | Central Asia | 2007 | 13041.83765 | 14488.38074 | 11712.90529 |
| Prevalence | Central Asia | 2008 | 13146.92377 | 14590.19989 | 11807.86532 |
| Prevalence | Central Asia | 2009 | 13252.28416 | 14697.45958 | 11909.00949 |
| Prevalence | Central Asia | 2010 | 13347.1521 | 14802.90907 | 11992.09151 |
| Prevalence | Central Asia | 2011 | 13441.33785 | 14914.57175 | 12071.29428 |
| Prevalence | Central Asia | 2012 | 13542.30102 | 15045.56661 | 12158.6456 |
| Prevalence | Central Asia | 2013 | 13643.39842 | 15176.50823 | 12240.59918 |
| Prevalence | Central Asia | 2014 | 13738.99169 | 15284.10184 | 12364.30769 |
| Prevalence | Central Asia | 2015 | 13823.6785 | 15356.46916 | 12437.64702 |
| Prevalence | Central Asia | 2016 | 13912.1577 | 15449.28924 | 12516.5049 |
| Prevalence | Central Asia | 2017 | 13993.31034 | 15534.75624 | 12605.82413 |
| Prevalence | Central Asia | 2018 | 14067.97505 | 15639.8861 | 12667.40753 |
| Prevalence | Central Asia | 2019 | 14150.98961 | 15748.82923 | 12737.68509 |
| Prevalence | Central Europe | 1990 | 10680.14572 | 11859.76467 | 9633.414703 |
| Prevalence | Central Europe | 1991 | 10746.36673 | 11922.19966 | 9690.030354 |
| Prevalence | Central Europe | 1992 | 10805.66578 | 11989.21074 | 9739.672713 |
| Prevalence | Central Europe | 1993 | 10859.10361 | 12041.6207 | 9784.168675 |
| Prevalence | Central Europe | 1994 | 10905.17043 | 12082.16795 | 9821.823806 |
| Prevalence | Central Europe | 1995 | 10940.73976 | 12113.54247 | 9853.716391 |
| Prevalence | Central Europe | 1996 | 10978.8828 | 12157.22061 | 9890.708766 |
| Prevalence | Central Europe | 1997 | 11027.78353 | 12219.74652 | 9947.508556 |
| Prevalence | Central Europe | 1998 | 11076.21542 | 12288.96173 | 10001.92857 |
| Prevalence | Central Europe | 1999 | 11114.08454 | 12326.40067 | 10040.96993 |
| Prevalence | Central Europe | 2000 | 11143.77117 | 12354.56805 | 10076.30293 |
| Prevalence | Central Europe | 2001 | 11169.66127 | 12372.96359 | 10090.65785 |
| Prevalence | Central Europe | 2002 | 11195.50114 | 12396.19158 | 10112.81384 |
| Prevalence | Central Europe | 2003 | 11220.86668 | 12437.32605 | 10127.56779 |
| Prevalence | Central Europe | 2004 | 11243.57166 | 12455.84446 | 10139.05838 |
| Prevalence | Central Europe | 2005 | 11265.43979 | 12476.78861 | 10155.35702 |
| Prevalence | Central Europe | 2006 | 11294.78319 | 12502.46116 | 10184.11467 |
| Prevalence | Central Europe | 2007 | 11332.51942 | 12542.43295 | 10231.16534 |
| Prevalence | Central Europe | 2008 | 11373.36392 | 12595.5042 | 10273.50325 |
| Prevalence | Central Europe | 2009 | 11415.29834 | 12629.16178 | 10308.85958 |
| Prevalence | Central Europe | 2010 | 11455.16952 | 12670.66386 | 10342.53562 |
| Prevalence | Central Europe | 2011 | 11499.6527 | 12714.23553 | 10382.09428 |
| Prevalence | Central Europe | 2012 | 11553.5004 | 12767.63749 | 10429.6894 |
| Prevalence | Central Europe | 2013 | 11609.18813 | 12822.36678 | 10481.56739 |
| Prevalence | Central Europe | 2014 | 11660.20609 | 12868.98525 | 10528.74176 |
| Prevalence | Central Europe | 2015 | 11701.09791 | 12906.78498 | 10568.6497 |
| Prevalence | Central Europe | 2016 | 11744.93286 | 12959.58465 | 10607.05203 |
| Prevalence | Central Europe | 2017 | 11787.64639 | 13017.40587 | 10618.53112 |
| Prevalence | Central Europe | 2018 | 11833.38842 | 13047.7295 | 10679.58771 |
| Prevalence | Central Europe | 2019 | 11895.11939 | 13109.7904 | 10755.06998 |
| Prevalence | Central Latin America | 1990 | 14593.29619 | 16254.56949 | 13114.00023 |
| Prevalence | Central Latin America | 1991 | 14692.5218 | 16361.99903 | 13214.66971 |
| Prevalence | Central Latin America | 1992 | 14790.68451 | 16463.4547 | 13308.84175 |
| Prevalence | Central Latin America | 1993 | 14885.01464 | 16561.73879 | 13394.20933 |
| Prevalence | Central Latin America | 1994 | 14972.73876 | 16652.96712 | 13473.84112 |
| Prevalence | Central Latin America | 1995 | 15051.49914 | 16734.55409 | 13545.28546 |
| Prevalence | Central Latin America | 1996 | 15132.01318 | 16827.78561 | 13629.06776 |
| Prevalence | Central Latin America | 1997 | 15219.67587 | 16888.77204 | 13720.41196 |
| Prevalence | Central Latin America | 1998 | 15305.55904 | 16968.24615 | 13800.49535 |
| Prevalence | Central Latin America | 1999 | 15381.11311 | 17072.75347 | 13870.84316 |
| Prevalence | Central Latin America | 2000 | 15438.06759 | 17154.8029 | 13919.58357 |
| Prevalence | Central Latin America | 2001 | 15484.06831 | 17188.58831 | 13957.18212 |
| Prevalence | Central Latin America | 2002 | 15530.8827 | 17222.30076 | 13991.0908 |
| Prevalence | Central Latin America | 2003 | 15576.8683 | 17245.52111 | 14039.50849 |
| Prevalence | Central Latin America | 2004 | 15620.2762 | 17288.23573 | 14089.01748 |
| Prevalence | Central Latin America | 2005 | 15660.26598 | 17339.34593 | 14119.3603 |
| Prevalence | Central Latin America | 2006 | 15709.3788 | 17395.76628 | 14160.106 |
| Prevalence | Central Latin America | 2007 | 15775.07021 | 17468.50871 | 14211.58046 |
| Prevalence | Central Latin America | 2008 | 15849.54187 | 17550.00072 | 14261.02481 |
| Prevalence | Central Latin America | 2009 | 15925.12701 | 17632.56976 | 14311.01199 |
| Prevalence | Central Latin America | 2010 | 15994.54616 | 17708.73771 | 14368.80464 |
| Prevalence | Central Latin America | 2011 | 16068.21801 | 17797.14724 | 14446.64858 |
| Prevalence | Central Latin America | 2012 | 16154.96891 | 17901.08581 | 14537.65095 |
| Prevalence | Central Latin America | 2013 | 16245.25789 | 17995.55313 | 14631.64584 |
| Prevalence | Central Latin America | 2014 | 16329.45426 | 18087.7064 | 14718.31023 |
| Prevalence | Central Latin America | 2015 | 16397.73327 | 18169.48548 | 14774.09094 |
| Prevalence | Central Latin America | 2016 | 16459.7355 | 18238.06106 | 14810.96271 |
| Prevalence | Central Latin America | 2017 | 16510.84372 | 18286.33944 | 14842.55196 |
| Prevalence | Central Latin America | 2018 | 16559.30824 | 18331.38541 | 14891.23863 |
| Prevalence | Central Latin America | 2019 | 16618.3121 | 18388.88828 | 14959.81728 |
| Prevalence | Central Sub-Saharan Africa | 1990 | 12574.10154 | 14247.90736 | 11111.20395 |
| Prevalence | Central Sub-Saharan Africa | 1991 | 12600.29532 | 14270.67396 | 11161.5903 |
| Prevalence | Central Sub-Saharan Africa | 1992 | 12627.65192 | 14267.96202 | 11188.69186 |
| Prevalence | Central Sub-Saharan Africa | 1993 | 12655.1575 | 14310.24888 | 11227.03386 |
| Prevalence | Central Sub-Saharan Africa | 1994 | 12681.92011 | 14342.06183 | 11248.09366 |
| Prevalence | Central Sub-Saharan Africa | 1995 | 12706.66378 | 14363.80334 | 11281.9088 |
| Prevalence | Central Sub-Saharan Africa | 1996 | 12738.06829 | 14386.34169 | 11295.42627 |
| Prevalence | Central Sub-Saharan Africa | 1997 | 12779.02454 | 14413.15623 | 11344.40052 |
| Prevalence | Central Sub-Saharan Africa | 1998 | 12821.6018 | 14448.10994 | 11365.07092 |
| Prevalence | Central Sub-Saharan Africa | 1999 | 12857.75129 | 14514.65808 | 11396.62645 |
| Prevalence | Central Sub-Saharan Africa | 2000 | 12879.1035 | 14561.64987 | 11403.46497 |
| Prevalence | Central Sub-Saharan Africa | 2001 | 12885.68268 | 14560.92073 | 11416.0594 |
| Prevalence | Central Sub-Saharan Africa | 2002 | 12884.82941 | 14558.87126 | 11427.19725 |
| Prevalence | Central Sub-Saharan Africa | 2003 | 12881.13584 | 14564.70788 | 11439.24903 |
| Prevalence | Central Sub-Saharan Africa | 2004 | 12878.97097 | 14570.33346 | 11451.21648 |
| Prevalence | Central Sub-Saharan Africa | 2005 | 12882.73643 | 14585.71799 | 11460.0601 |
| Prevalence | Central Sub-Saharan Africa | 2006 | 12895.40665 | 14577.83008 | 11473.34411 |
| Prevalence | Central Sub-Saharan Africa | 2007 | 12913.98258 | 14627.61577 | 11479.92549 |
| Prevalence | Central Sub-Saharan Africa | 2008 | 12935.77795 | 14640.06062 | 11491.74035 |
| Prevalence | Central Sub-Saharan Africa | 2009 | 12957.89334 | 14659.04866 | 11515.16956 |
| Prevalence | Central Sub-Saharan Africa | 2010 | 12977.39922 | 14696.49312 | 11526.48557 |
| Prevalence | Central Sub-Saharan Africa | 2011 | 12995.98542 | 14710.49685 | 11509.61221 |
| Prevalence | Central Sub-Saharan Africa | 2012 | 13015.9859 | 14712.91614 | 11522.78939 |
| Prevalence | Central Sub-Saharan Africa | 2013 | 13037.58483 | 14730.01116 | 11517.84274 |
| Prevalence | Central Sub-Saharan Africa | 2014 | 13060.8263 | 14759.23158 | 11561.34781 |
| Prevalence | Central Sub-Saharan Africa | 2015 | 13085.66287 | 14764.59963 | 11614.40117 |
| Prevalence | Central Sub-Saharan Africa | 2016 | 13150.62996 | 14837.84839 | 11654.55793 |
| Prevalence | Central Sub-Saharan Africa | 2017 | 13222.89858 | 14905.25909 | 11665.18425 |
| Prevalence | Central Sub-Saharan Africa | 2018 | 13273.71785 | 14999.91708 | 11739.77585 |
| Prevalence | Central Sub-Saharan Africa | 2019 | 13331.37174 | 15097.47641 | 11795.60784 |
| Prevalence | East Asia | 1990 | 12538.48616 | 14232.15213 | 11109.64047 |
| Prevalence | East Asia | 1991 | 12648.58321 | 14337.74252 | 11206.51977 |
| Prevalence | East Asia | 1992 | 12737.94852 | 14423.51211 | 11295.67348 |
| Prevalence | East Asia | 1993 | 12809.65461 | 14492.10027 | 11367.80895 |
| Prevalence | East Asia | 1994 | 12866.49223 | 14546.19218 | 11416.33269 |
| Prevalence | East Asia | 1995 | 12911.88551 | 14588.6453 | 11457.25594 |
| Prevalence | East Asia | 1996 | 12946.34811 | 14630.35257 | 11494.50303 |
| Prevalence | East Asia | 1997 | 12966.01099 | 14656.33358 | 11518.66958 |
| Prevalence | East Asia | 1998 | 12966.95588 | 14657.76906 | 11525.42433 |
| Prevalence | East Asia | 1999 | 12944.29826 | 14616.8702 | 11502.84401 |
| Prevalence | East Asia | 2000 | 12893.71417 | 14546.35498 | 11457.27048 |
| Prevalence | East Asia | 2001 | 12731.45018 | 14368.85714 | 11314.24398 |
| Prevalence | East Asia | 2002 | 12440.8832 | 14057.35688 | 11071.39437 |
| Prevalence | East Asia | 2003 | 12117.4997 | 13683.00877 | 10781.02513 |
| Prevalence | East Asia | 2004 | 11856.84712 | 13378.19088 | 10567.99625 |
| Prevalence | East Asia | 2005 | 11755.61578 | 13260.74814 | 10482.30991 |
| Prevalence | East Asia | 2006 | 11912.07563 | 13444.97431 | 10626.88402 |
| Prevalence | East Asia | 2007 | 12290.60237 | 13861.50189 | 10970.28407 |
| Prevalence | East Asia | 2008 | 12785.35042 | 14392.66678 | 11417.44032 |
| Prevalence | East Asia | 2009 | 13290.3475 | 14932.37882 | 11874.7786 |
| Prevalence | East Asia | 2010 | 13700.12428 | 15364.72965 | 12250.45848 |
| Prevalence | East Asia | 2011 | 14031.66182 | 15701.04635 | 12541.58668 |
| Prevalence | East Asia | 2012 | 14365.71587 | 16055.86153 | 12836.65876 |
| Prevalence | East Asia | 2013 | 14692.27968 | 16428.81994 | 13132.71757 |
| Prevalence | East Asia | 2014 | 15001.37393 | 16772.04078 | 13405.36959 |
| Prevalence | East Asia | 2015 | 15282.8866 | 17078.61767 | 13669.07221 |
| Prevalence | East Asia | 2016 | 15820.14282 | 17651.98918 | 14142.76747 |
| Prevalence | East Asia | 2017 | 16286.93964 | 18223.96215 | 14556.70836 |
| Prevalence | East Asia | 2018 | 16190.38285 | 18099.77079 | 14475.54382 |
| Prevalence | East Asia | 2019 | 15681.50439 | 17552.74902 | 14023.07437 |
| Prevalence | Eastern Europe | 1990 | 11026.16536 | 12225.30925 | 9932.197518 |
| Prevalence | Eastern Europe | 1991 | 11072.18543 | 12268.712 | 9977.242144 |
| Prevalence | Eastern Europe | 1992 | 11117.80762 | 12315.50745 | 10008.9104 |
| Prevalence | Eastern Europe | 1993 | 11157.35177 | 12353.79158 | 10046.9632 |
| Prevalence | Eastern Europe | 1994 | 11189.74395 | 12381.98227 | 10089.71255 |
| Prevalence | Eastern Europe | 1995 | 11214.95849 | 12398.22078 | 10109.63807 |
| Prevalence | Eastern Europe | 1996 | 11240.97067 | 12421.16448 | 10131.4321 |
| Prevalence | Eastern Europe | 1997 | 11273.07577 | 12457.44187 | 10146.2512 |
| Prevalence | Eastern Europe | 1998 | 11306.59726 | 12494.56162 | 10175.50691 |
| Prevalence | Eastern Europe | 1999 | 11339.00056 | 12514.43086 | 10208.44564 |
| Prevalence | Eastern Europe | 2000 | 11367.51242 | 12548.16663 | 10240.7921 |
| Prevalence | Eastern Europe | 2001 | 11398.74924 | 12589.66939 | 10279.38562 |
| Prevalence | Eastern Europe | 2002 | 11436.42935 | 12633.28455 | 10326.88614 |
| Prevalence | Eastern Europe | 2003 | 11476.04169 | 12679.03639 | 10375.42967 |
| Prevalence | Eastern Europe | 2004 | 11516.14205 | 12726.7135 | 10420.95932 |
| Prevalence | Eastern Europe | 2005 | 11554.31743 | 12773.57337 | 10459.37944 |
| Prevalence | Eastern Europe | 2006 | 11600.94226 | 12819.13291 | 10496.43868 |
| Prevalence | Eastern Europe | 2007 | 11661.42401 | 12883.53462 | 10547.48325 |
| Prevalence | Eastern Europe | 2008 | 11726.21044 | 12958.10405 | 10599.17259 |
| Prevalence | Eastern Europe | 2009 | 11789.81819 | 13031.77631 | 10651.54655 |
| Prevalence | Eastern Europe | 2010 | 11846.05386 | 13088.89881 | 10694.80499 |
| Prevalence | Eastern Europe | 2011 | 11903.94315 | 13151.04826 | 10749.02253 |
| Prevalence | Eastern Europe | 2012 | 11970.68877 | 13211.09594 | 10813.42464 |
| Prevalence | Eastern Europe | 2013 | 12037.10703 | 13281.74715 | 10877.61823 |
| Prevalence | Eastern Europe | 2014 | 12096.08872 | 13354.50084 | 10940.05551 |
| Prevalence | Eastern Europe | 2015 | 12140.44707 | 13411.62727 | 10978.00694 |
| Prevalence | Eastern Europe | 2016 | 12176.05867 | 13416.18501 | 11022.74026 |
| Prevalence | Eastern Europe | 2017 | 12208.17351 | 13465.00798 | 11061.01868 |
| Prevalence | Eastern Europe | 2018 | 12246.0106 | 13527.04789 | 11076.76697 |
| Prevalence | Eastern Europe | 2019 | 12295.31117 | 13601.99446 | 11098.69668 |
| Prevalence | Eastern Sub-Saharan Africa | 1990 | 12556.45659 | 14168.85157 | 11140.37722 |
| Prevalence | Eastern Sub-Saharan Africa | 1991 | 12580.47721 | 14200.84626 | 11158.95324 |
| Prevalence | Eastern Sub-Saharan Africa | 1992 | 12617.69904 | 14242.82382 | 11196.87621 |
| Prevalence | Eastern Sub-Saharan Africa | 1993 | 12661.19448 | 14291.61406 | 11243.60065 |
| Prevalence | Eastern Sub-Saharan Africa | 1994 | 12691.41391 | 14324.2729 | 11266.64879 |
| Prevalence | Eastern Sub-Saharan Africa | 1995 | 12713.43026 | 14342.7083 | 11298.30059 |
| Prevalence | Eastern Sub-Saharan Africa | 1996 | 12742.12645 | 14379.25722 | 11329.64628 |
| Prevalence | Eastern Sub-Saharan Africa | 1997 | 12790.77038 | 14436.25914 | 11376.86163 |
| Prevalence | Eastern Sub-Saharan Africa | 1998 | 12851.36385 | 14481.25756 | 11434.70235 |
| Prevalence | Eastern Sub-Saharan Africa | 1999 | 12909.86867 | 14524.56229 | 11490.05366 |
| Prevalence | Eastern Sub-Saharan Africa | 2000 | 12956.72045 | 14574.47878 | 11522.99923 |
| Prevalence | Eastern Sub-Saharan Africa | 2001 | 12997.04632 | 14616.8734 | 11557.34425 |
| Prevalence | Eastern Sub-Saharan Africa | 2002 | 13041.39604 | 14678.12747 | 11595.10224 |
| Prevalence | Eastern Sub-Saharan Africa | 2003 | 13085.73366 | 14733.8828 | 11634.19192 |
| Prevalence | Eastern Sub-Saharan Africa | 2004 | 13126.11103 | 14772.58787 | 11662.72732 |
| Prevalence | Eastern Sub-Saharan Africa | 2005 | 13158.19547 | 14826.16053 | 11687.51884 |
| Prevalence | Eastern Sub-Saharan Africa | 2006 | 13182.25799 | 14831.91106 | 11712.78513 |
| Prevalence | Eastern Sub-Saharan Africa | 2007 | 13204.96592 | 14850.05322 | 11737.26002 |
| Prevalence | Eastern Sub-Saharan Africa | 2008 | 13227.68535 | 14889.97791 | 11761.91708 |
| Prevalence | Eastern Sub-Saharan Africa | 2009 | 13251.35156 | 14904.05328 | 11780.79362 |
| Prevalence | Eastern Sub-Saharan Africa | 2010 | 13277.46526 | 14921.19551 | 11814.05886 |
| Prevalence | Eastern Sub-Saharan Africa | 2011 | 13309.68515 | 14952.60257 | 11841.1898 |
| Prevalence | Eastern Sub-Saharan Africa | 2012 | 13349.36367 | 14991.02032 | 11875.33443 |
| Prevalence | Eastern Sub-Saharan Africa | 2013 | 13392.82146 | 15013.7487 | 11913.34952 |
| Prevalence | Eastern Sub-Saharan Africa | 2014 | 13436.27584 | 15051.52512 | 11951.16161 |
| Prevalence | Eastern Sub-Saharan Africa | 2015 | 13474.70236 | 15099.3191 | 11989.35716 |
| Prevalence | Eastern Sub-Saharan Africa | 2016 | 13534.22624 | 15183.0347 | 12048.44064 |
| Prevalence | Eastern Sub-Saharan Africa | 2017 | 13596.27312 | 15235.74629 | 12090.06598 |
| Prevalence | Eastern Sub-Saharan Africa | 2018 | 13647.40571 | 15286.12389 | 12145.19031 |
| Prevalence | Eastern Sub-Saharan Africa | 2019 | 13709.42566 | 15370.63775 | 12222.77304 |
| Prevalence | Global | 1990 | 12065.6273 | 13536.97376 | 10779.51408 |
| Prevalence | Global | 1991 | 12158.39245 | 13637.19148 | 10866.7718 |
| Prevalence | Global | 1992 | 12246.28598 | 13731.69949 | 10947.98828 |
| Prevalence | Global | 1993 | 12330.19867 | 13828.42504 | 11019.94637 |
| Prevalence | Global | 1994 | 12409.05801 | 13915.40885 | 11087.3694 |
| Prevalence | Global | 1995 | 12482.51645 | 13987.744 | 11149.88329 |
| Prevalence | Global | 1996 | 12559.2526 | 14077.32428 | 11224.9455 |
| Prevalence | Global | 1997 | 12638.61403 | 14169.88886 | 11302.97119 |
| Prevalence | Global | 1998 | 12715.04477 | 14259.28422 | 11370.86386 |
| Prevalence | Global | 1999 | 12782.14117 | 14337.36341 | 11435.78723 |
| Prevalence | Global | 2000 | 12833.12183 | 14393.43783 | 11486.00299 |
| Prevalence | Global | 2001 | 12852.35681 | 14401.06555 | 11510.75332 |
| Prevalence | Global | 2002 | 12839.98156 | 14371.51812 | 11507.57556 |
| Prevalence | Global | 2003 | 12820.5031 | 14335.15901 | 11497.6967 |
| Prevalence | Global | 2004 | 12817.17068 | 14317.97028 | 11501.73063 |
| Prevalence | Global | 2005 | 12853.55156 | 14352.05084 | 11540.89133 |
| Prevalence | Global | 2006 | 12959.83423 | 14469.21966 | 11633.85259 |
| Prevalence | Global | 2007 | 13128.07935 | 14655.04248 | 11783.54563 |
| Prevalence | Global | 2008 | 13328.89125 | 14879.81131 | 11964.39647 |
| Prevalence | Global | 2009 | 13531.98346 | 15107.71463 | 12151.46453 |
| Prevalence | Global | 2010 | 13708.48074 | 15302.11761 | 12313.79574 |
| Prevalence | Global | 2011 | 13884.32381 | 15497.45279 | 12477.70716 |
| Prevalence | Global | 2012 | 14085.54905 | 15718.91856 | 12651.47731 |
| Prevalence | Global | 2013 | 14292.69612 | 15948.04344 | 12823.56223 |
| Prevalence | Global | 2014 | 14486.07608 | 16165.27806 | 12996.04836 |
| Prevalence | Global | 2015 | 14645.75962 | 16339.74825 | 13138.76873 |
| Prevalence | Global | 2016 | 14843.5792 | 16559.33418 | 13316.7408 |
| Prevalence | Global | 2017 | 15015.96007 | 16749.73423 | 13464.14904 |
| Prevalence | Global | 2018 | 15062.42283 | 16797.84659 | 13527.19461 |
| Prevalence | Global | 2019 | 15023.47346 | 16764.84154 | 13493.72762 |
| Prevalence | High-income Asia Pacific | 1990 | 6835.475434 | 7694.948818 | 6080.835605 |
| Prevalence | High-income Asia Pacific | 1991 | 6814.307468 | 7678.77371 | 6059.717455 |
| Prevalence | High-income Asia Pacific | 1992 | 6796.584366 | 7667.120636 | 6043.437875 |
| Prevalence | High-income Asia Pacific | 1993 | 6782.360616 | 7659.275934 | 6033.96267 |
| Prevalence | High-income Asia Pacific | 1994 | 6772.523739 | 7647.638962 | 6023.397684 |
| Prevalence | High-income Asia Pacific | 1995 | 6767.330977 | 7635.32797 | 6025.093337 |
| Prevalence | High-income Asia Pacific | 1996 | 6742.84018 | 7566.2971 | 6023.543903 |
| Prevalence | High-income Asia Pacific | 1997 | 6688.093437 | 7463.061241 | 6010.284475 |
| Prevalence | High-income Asia Pacific | 1998 | 6626.303971 | 7368.190008 | 5985.006152 |
| Prevalence | High-income Asia Pacific | 1999 | 6578.985476 | 7301.867102 | 5954.733718 |
| Prevalence | High-income Asia Pacific | 2000 | 6573.138561 | 7275.292455 | 5954.41582 |
| Prevalence | High-income Asia Pacific | 2001 | 6625.547608 | 7333.331019 | 6006.616582 |
| Prevalence | High-income Asia Pacific | 2002 | 6722.711172 | 7438.985734 | 6095.760112 |
| Prevalence | High-income Asia Pacific | 2003 | 6841.998089 | 7568.270996 | 6191.536425 |
| Prevalence | High-income Asia Pacific | 2004 | 6963.884941 | 7701.716762 | 6289.096145 |
| Prevalence | High-income Asia Pacific | 2005 | 7065.288581 | 7815.192148 | 6376.582783 |
| Prevalence | High-income Asia Pacific | 2006 | 7148.050538 | 7906.192732 | 6463.651038 |
| Prevalence | High-income Asia Pacific | 2007 | 7228.048163 | 7990.995319 | 6544.775618 |
| Prevalence | High-income Asia Pacific | 2008 | 7302.972993 | 8070.804876 | 6614.263737 |
| Prevalence | High-income Asia Pacific | 2009 | 7367.085541 | 8145.803233 | 6667.183253 |
| Prevalence | High-income Asia Pacific | 2010 | 7418.197813 | 8211.196468 | 6710.931711 |
| Prevalence | High-income Asia Pacific | 2011 | 7472.229255 | 8278.045148 | 6752.964281 |
| Prevalence | High-income Asia Pacific | 2012 | 7539.953937 | 8364.134115 | 6807.11288 |
| Prevalence | High-income Asia Pacific | 2013 | 7611.546061 | 8448.601299 | 6863.321081 |
| Prevalence | High-income Asia Pacific | 2014 | 7676.117646 | 8528.234902 | 6908.022486 |
| Prevalence | High-income Asia Pacific | 2015 | 7723.941644 | 8591.826603 | 6935.855752 |
| Prevalence | High-income Asia Pacific | 2016 | 7601.381439 | 8485.943809 | 6802.390671 |
| Prevalence | High-income Asia Pacific | 2017 | 7460.631563 | 8339.127323 | 6664.341188 |
| Prevalence | High-income Asia Pacific | 2018 | 7525.467469 | 8445.775594 | 6711.155613 |
| Prevalence | High-income Asia Pacific | 2019 | 7673.15192 | 8630.741043 | 6831.484146 |
| Prevalence | High-income North America | 1990 | 7263.201695 | 8251.36006 | 6424.707794 |
| Prevalence | High-income North America | 1991 | 7317.300853 | 8311.892232 | 6471.64639 |
| Prevalence | High-income North America | 1992 | 7374.501827 | 8379.372729 | 6528.452813 |
| Prevalence | High-income North America | 1993 | 7432.388688 | 8436.369928 | 6586.024404 |
| Prevalence | High-income North America | 1994 | 7488.245789 | 8490.002764 | 6640.15514 |
| Prevalence | High-income North America | 1995 | 7540.278482 | 8553.885129 | 6693.483294 |
| Prevalence | High-income North America | 1996 | 7607.723142 | 8628.044882 | 6746.641416 |
| Prevalence | High-income North America | 1997 | 7701.909265 | 8730.939518 | 6831.172242 |
| Prevalence | High-income North America | 1998 | 7806.880197 | 8846.682864 | 6928.059994 |
| Prevalence | High-income North America | 1999 | 7906.383239 | 8961.130397 | 7017.387628 |
| Prevalence | High-income North America | 2000 | 7984.56243 | 9047.075501 | 7083.41046 |
| Prevalence | High-income North America | 2001 | 8055.706902 | 9121.586295 | 7146.448597 |
| Prevalence | High-income North America | 2002 | 8140.861374 | 9213.059958 | 7223.387888 |
| Prevalence | High-income North America | 2003 | 8232.897148 | 9316.440095 | 7306.982816 |
| Prevalence | High-income North America | 2004 | 8324.491275 | 9419.734952 | 7397.905017 |
| Prevalence | High-income North America | 2005 | 8408.720019 | 9511.068586 | 7478.146791 |
| Prevalence | High-income North America | 2006 | 8486.378567 | 9588.273583 | 7545.792763 |
| Prevalence | High-income North America | 2007 | 8564.095368 | 9671.40968 | 7613.276155 |
| Prevalence | High-income North America | 2008 | 8642.57077 | 9756.395535 | 7684.807846 |
| Prevalence | High-income North America | 2009 | 8722.181194 | 9846.879253 | 7758.270583 |
| Prevalence | High-income North America | 2010 | 8803.598938 | 9938.46017 | 7830.924429 |
| Prevalence | High-income North America | 2011 | 8908.444453 | 10050.90525 | 7934.836014 |
| Prevalence | High-income North America | 2012 | 9040.818002 | 10192.45836 | 8062.628374 |
| Prevalence | High-income North America | 2013 | 9174.424137 | 10326.55855 | 8192.232808 |
| Prevalence | High-income North America | 2014 | 9282.705622 | 10430.05032 | 8297.603762 |
| Prevalence | High-income North America | 2015 | 9339.175445 | 10485.77765 | 8343.228435 |
| Prevalence | High-income North America | 2016 | 9347.517089 | 10490.05613 | 8367.368409 |
| Prevalence | High-income North America | 2017 | 9348.805507 | 10495.9375 | 8349.775821 |
| Prevalence | High-income North America | 2018 | 9366.09548 | 10514.16014 | 8372.297621 |
| Prevalence | High-income North America | 2019 | 9396.668055 | 10543.35474 | 8404.823193 |
| Prevalence | North Africa and Middle East | 1990 | 24422.07507 | 26866.86889 | 22219.39027 |
| Prevalence | North Africa and Middle East | 1991 | 24475.61145 | 26897.93503 | 22256.90428 |
| Prevalence | North Africa and Middle East | 1992 | 24531.12596 | 26943.28825 | 22309.60071 |
| Prevalence | North Africa and Middle East | 1993 | 24594.80495 | 26985.98584 | 22374.96292 |
| Prevalence | North Africa and Middle East | 1994 | 24667.28291 | 27067.5095 | 22448.56351 |
| Prevalence | North Africa and Middle East | 1995 | 24743.64855 | 27160.37477 | 22530.83867 |
| Prevalence | North Africa and Middle East | 1996 | 24834.18199 | 27240.33455 | 22610.19827 |
| Prevalence | North Africa and Middle East | 1997 | 24945.88924 | 27346.70525 | 22712.5663 |
| Prevalence | North Africa and Middle East | 1998 | 25069.23366 | 27459.81408 | 22824.43959 |
| Prevalence | North Africa and Middle East | 1999 | 25190.52435 | 27592.50782 | 22951.05363 |
| Prevalence | North Africa and Middle East | 2000 | 25294.97674 | 27710.7881 | 23034.40476 |
| Prevalence | North Africa and Middle East | 2001 | 25428.23915 | 27846.27887 | 23163.17691 |
| Prevalence | North Africa and Middle East | 2002 | 25621.63369 | 28045.54344 | 23351.60107 |
| Prevalence | North Africa and Middle East | 2003 | 25838.91554 | 28263.66152 | 23558.85363 |
| Prevalence | North Africa and Middle East | 2004 | 26037.11531 | 28450.66201 | 23756.44775 |
| Prevalence | North Africa and Middle East | 2005 | 26175.86201 | 28582.007 | 23889.873 |
| Prevalence | North Africa and Middle East | 2006 | 26285.48804 | 28713.83797 | 24012.17188 |
| Prevalence | North Africa and Middle East | 2007 | 26413.07111 | 28833.3394 | 24096.09808 |
| Prevalence | North Africa and Middle East | 2008 | 26550.93614 | 28970.9513 | 24224.66196 |
| Prevalence | North Africa and Middle East | 2009 | 26686.83466 | 29134.49235 | 24358.4087 |
| Prevalence | North Africa and Middle East | 2010 | 26804.85702 | 29274.68392 | 24483.62358 |
| Prevalence | North Africa and Middle East | 2011 | 26894.14237 | 29337.63901 | 24527.91298 |
| Prevalence | North Africa and Middle East | 2012 | 26958.29021 | 29421.90968 | 24585.90342 |
| Prevalence | North Africa and Middle East | 2013 | 27015.59289 | 29507.36781 | 24638.01791 |
| Prevalence | North Africa and Middle East | 2014 | 27087.00124 | 29605.72762 | 24698.75555 |
| Prevalence | North Africa and Middle East | 2015 | 27188.83779 | 29731.7884 | 24813.7519 |
| Prevalence | North Africa and Middle East | 2016 | 27363.56893 | 29879.84671 | 25021.81123 |
| Prevalence | North Africa and Middle East | 2017 | 27531.00485 | 30066.32577 | 25202.86908 |
| Prevalence | North Africa and Middle East | 2018 | 27644.40088 | 30186.49499 | 25341.4704 |
| Prevalence | North Africa and Middle East | 2019 | 27749.31093 | 30284.09495 | 25410.63391 |
| Prevalence | Oceania | 1990 | 15748.10715 | 17647.44418 | 14076.86319 |
| Prevalence | Oceania | 1991 | 15860.23255 | 17795.46386 | 14181.89815 |
| Prevalence | Oceania | 1992 | 15967.62215 | 17877.47633 | 14276.44089 |
| Prevalence | Oceania | 1993 | 16069.0176 | 17961.89523 | 14381.54086 |
| Prevalence | Oceania | 1994 | 16163.62417 | 18043.1817 | 14469.19131 |
| Prevalence | Oceania | 1995 | 16251.05175 | 18139.18047 | 14548.70408 |
| Prevalence | Oceania | 1996 | 16337.56912 | 18211.70656 | 14619.86612 |
| Prevalence | Oceania | 1997 | 16426.94542 | 18323.58515 | 14688.82617 |
| Prevalence | Oceania | 1998 | 16513.78741 | 18422.55736 | 14756.87824 |
| Prevalence | Oceania | 1999 | 16592.16458 | 18509.15212 | 14830.38177 |
| Prevalence | Oceania | 2000 | 16656.30608 | 18625.16264 | 14875.6471 |
| Prevalence | Oceania | 2001 | 16719.21726 | 18671.94574 | 14918.78763 |
| Prevalence | Oceania | 2002 | 16787.8029 | 18730.2043 | 14985.15 |
| Prevalence | Oceania | 2003 | 16850.13621 | 18793.63619 | 15057.40649 |
| Prevalence | Oceania | 2004 | 16894.26167 | 18837.276 | 15091.96431 |
| Prevalence | Oceania | 2005 | 16907.81956 | 18844.33297 | 15103.43372 |
| Prevalence | Oceania | 2006 | 16884.2382 | 18827.17006 | 15099.5329 |
| Prevalence | Oceania | 2007 | 16834.46602 | 18785.21331 | 15079.97815 |
| Prevalence | Oceania | 2008 | 16775.4695 | 18732.70941 | 15035.12506 |
| Prevalence | Oceania | 2009 | 16723.63102 | 18678.11966 | 15008.9972 |
| Prevalence | Oceania | 2010 | 16695.25613 | 18669.26649 | 15001.96885 |
| Prevalence | Oceania | 2011 | 16694.28985 | 18670.54934 | 15015.79897 |
| Prevalence | Oceania | 2012 | 16707.38132 | 18724.90486 | 14996.90692 |
| Prevalence | Oceania | 2013 | 16728.07679 | 18738.1264 | 15029.38935 |
| Prevalence | Oceania | 2014 | 16749.69251 | 18724.40341 | 15018.66315 |
| Prevalence | Oceania | 2015 | 16765.78528 | 18701.764 | 15019.28528 |
| Prevalence | Oceania | 2016 | 16797.51041 | 18706.89168 | 15093.0845 |
| Prevalence | Oceania | 2017 | 16827.93224 | 18719.6132 | 15110.06167 |
| Prevalence | Oceania | 2018 | 16846.24742 | 18733.47205 | 15133.15731 |
| Prevalence | Oceania | 2019 | 16869.47762 | 18727.12968 | 15133.71492 |
| Prevalence | South Asia | 1990 | 12250.88454 | 13935.43528 | 10869.30953 |
| Prevalence | South Asia | 1991 | 12275.06265 | 13977.35595 | 10895.93988 |
| Prevalence | South Asia | 1992 | 12305.24559 | 14026.62762 | 10918.80246 |
| Prevalence | South Asia | 1993 | 12339.51615 | 14079.1514 | 10945.71166 |
| Prevalence | South Asia | 1994 | 12376.07699 | 14131.08737 | 10975.22974 |
| Prevalence | South Asia | 1995 | 12413.58718 | 14148.68001 | 11005.90331 |
| Prevalence | South Asia | 1996 | 12468.82988 | 14214.58137 | 11058.13141 |
| Prevalence | South Asia | 1997 | 12548.30173 | 14309.68887 | 11131.82596 |
| Prevalence | South Asia | 1998 | 12633.96372 | 14402.07906 | 11211.272 |
| Prevalence | South Asia | 1999 | 12707.74668 | 14473.64424 | 11280.6487 |
| Prevalence | South Asia | 2000 | 12751.25622 | 14511.25662 | 11323.58925 |
| Prevalence | South Asia | 2001 | 12737.1153 | 14499.90532 | 11318.64281 |
| Prevalence | South Asia | 2002 | 12673.68566 | 14433.6306 | 11255.25804 |
| Prevalence | South Asia | 2003 | 12594.20584 | 14330.67191 | 11178.03608 |
| Prevalence | South Asia | 2004 | 12532.20474 | 14244.22258 | 11127.48875 |
| Prevalence | South Asia | 2005 | 12521.6095 | 14218.28108 | 11137.55554 |
| Prevalence | South Asia | 2006 | 12558.53814 | 14249.31294 | 11168.65601 |
| Prevalence | South Asia | 2007 | 12614.23314 | 14301.86923 | 11222.04808 |
| Prevalence | South Asia | 2008 | 12684.43487 | 14370.58372 | 11306.05223 |
| Prevalence | South Asia | 2009 | 12765.23298 | 14456.83126 | 11379.29436 |
| Prevalence | South Asia | 2010 | 12853.04308 | 14551.7463 | 11457.53851 |
| Prevalence | South Asia | 2011 | 13025.15275 | 14746.44378 | 11616.70823 |
| Prevalence | South Asia | 2012 | 13311.52555 | 15084.16678 | 11860.26002 |
| Prevalence | South Asia | 2013 | 13638.04767 | 15461.70874 | 12144.14498 |
| Prevalence | South Asia | 2014 | 13930.99394 | 15771.5315 | 12411.84555 |
| Prevalence | South Asia | 2015 | 14117.28203 | 15961.82968 | 12583.75952 |
| Prevalence | South Asia | 2016 | 14212.09566 | 16098.55021 | 12676.35318 |
| Prevalence | South Asia | 2017 | 14291.21899 | 16183.40735 | 12767.2993 |
| Prevalence | South Asia | 2018 | 14390.1624 | 16287.7585 | 12846.30591 |
| Prevalence | South Asia | 2019 | 14514.15914 | 16400.88741 | 12969.47874 |
| Prevalence | Southeast Asia | 1990 | 16105.96617 | 18017.03084 | 14459.63703 |
| Prevalence | Southeast Asia | 1991 | 16164.92664 | 18067.85617 | 14507.93783 |
| Prevalence | Southeast Asia | 1992 | 16223.49985 | 18115.549 | 14558.66007 |
| Prevalence | Southeast Asia | 1993 | 16280.12588 | 18160.68508 | 14621.9578 |
| Prevalence | Southeast Asia | 1994 | 16333.28807 | 18248.71408 | 14646.51708 |
| Prevalence | Southeast Asia | 1995 | 16381.89235 | 18291.17265 | 14686.16816 |
| Prevalence | Southeast Asia | 1996 | 16428.21277 | 18338.60025 | 14739.10164 |
| Prevalence | Southeast Asia | 1997 | 16477.24616 | 18389.77123 | 14797.15386 |
| Prevalence | Southeast Asia | 1998 | 16528.60704 | 18437.57268 | 14857.41199 |
| Prevalence | Southeast Asia | 1999 | 16582.14316 | 18494.59788 | 14881.75833 |
| Prevalence | Southeast Asia | 2000 | 16638.08106 | 18551.44348 | 14925.32372 |
| Prevalence | Southeast Asia | 2001 | 16707.69878 | 18617.90831 | 14993.20945 |
| Prevalence | Southeast Asia | 2002 | 16798.09449 | 18729.89882 | 15092.74382 |
| Prevalence | Southeast Asia | 2003 | 16898.27419 | 18852.81945 | 15181.6087 |
| Prevalence | Southeast Asia | 2004 | 16997.2079 | 18972.92417 | 15269.83821 |
| Prevalence | Southeast Asia | 2005 | 17085.28089 | 19077.90908 | 15348.25499 |
| Prevalence | Southeast Asia | 2006 | 17166.0554 | 19138.87099 | 15415.62413 |
| Prevalence | Southeast Asia | 2007 | 17250.3239 | 19207.48942 | 15482.18404 |
| Prevalence | Southeast Asia | 2008 | 17335.04028 | 19279.59069 | 15551.4026 |
| Prevalence | Southeast Asia | 2009 | 17417.69784 | 19354.77054 | 15629.42116 |
| Prevalence | Southeast Asia | 2010 | 17496.81622 | 19413.77096 | 15704.81956 |
| Prevalence | Southeast Asia | 2011 | 17578.15273 | 19504.6796 | 15785.97807 |
| Prevalence | Southeast Asia | 2012 | 17667.35158 | 19618.76988 | 15876.28131 |
| Prevalence | Southeast Asia | 2013 | 17760.36326 | 19731.46996 | 15972.83099 |
| Prevalence | Southeast Asia | 2014 | 17853.15689 | 19849.07358 | 16053.78012 |
| Prevalence | Southeast Asia | 2015 | 17941.86506 | 19959.57862 | 16126.25936 |
| Prevalence | Southeast Asia | 2016 | 18053.11769 | 20048.7792 | 16239.80906 |
| Prevalence | Southeast Asia | 2017 | 18153.95546 | 20163.30618 | 16344.30495 |
| Prevalence | Southeast Asia | 2018 | 18226.35223 | 20227.95409 | 16411.38232 |
| Prevalence | Southeast Asia | 2019 | 18299.94628 | 20310.25387 | 16509.31415 |
| Prevalence | Southern Latin America | 1990 | 6617.383484 | 7492.523468 | 5889.058445 |
| Prevalence | Southern Latin America | 1991 | 6701.885714 | 7573.174332 | 5964.069552 |
| Prevalence | Southern Latin America | 1992 | 6790.961668 | 7654.111918 | 6045.69826 |
| Prevalence | Southern Latin America | 1993 | 6883.929239 | 7749.708425 | 6129.535236 |
| Prevalence | Southern Latin America | 1994 | 6980.144896 | 7846.087418 | 6218.620144 |
| Prevalence | Southern Latin America | 1995 | 7079.143274 | 7946.945089 | 6306.344785 |
| Prevalence | Southern Latin America | 1996 | 7186.831274 | 8068.517774 | 6397.225467 |
| Prevalence | Southern Latin America | 1997 | 7304.511434 | 8195.428206 | 6495.119222 |
| Prevalence | Southern Latin America | 1998 | 7425.142981 | 8334.664364 | 6595.826748 |
| Prevalence | Southern Latin America | 1999 | 7541.456857 | 8473.569451 | 6701.912962 |
| Prevalence | Southern Latin America | 2000 | 7646.378932 | 8598.036814 | 6796.948448 |
| Prevalence | Southern Latin America | 2001 | 7744.10212 | 8693.775371 | 6891.296844 |
| Prevalence | Southern Latin America | 2002 | 7840.376628 | 8802.122286 | 6993.627687 |
| Prevalence | Southern Latin America | 2003 | 7931.126061 | 8903.816804 | 7081.807707 |
| Prevalence | Southern Latin America | 2004 | 8012.135767 | 8980.720656 | 7157.78625 |
| Prevalence | Southern Latin America | 2005 | 8079.121592 | 9044.168136 | 7216.529856 |
| Prevalence | Southern Latin America | 2006 | 8135.134196 | 9098.853116 | 7272.623523 |
| Prevalence | Southern Latin America | 2007 | 8185.928075 | 9126.530559 | 7326.051185 |
| Prevalence | Southern Latin America | 2008 | 8231.879429 | 9164.906666 | 7372.596332 |
| Prevalence | Southern Latin America | 2009 | 8273.235602 | 9194.106573 | 7409.625472 |
| Prevalence | Southern Latin America | 2010 | 8310.274024 | 9253.522327 | 7448.405877 |
| Prevalence | Southern Latin America | 2011 | 8346.630915 | 9286.340878 | 7479.455943 |
| Prevalence | Southern Latin America | 2012 | 8383.741683 | 9334.586315 | 7525.80785 |
| Prevalence | Southern Latin America | 2013 | 8419.149199 | 9398.517635 | 7565.514352 |
| Prevalence | Southern Latin America | 2014 | 8450.338426 | 9433.48796 | 7583.987703 |
| Prevalence | Southern Latin America | 2015 | 8475.286304 | 9449.563006 | 7613.215622 |
| Prevalence | Southern Latin America | 2016 | 8499.335827 | 9501.620422 | 7624.53937 |
| Prevalence | Southern Latin America | 2017 | 8523.270849 | 9547.422459 | 7622.02809 |
| Prevalence | Southern Latin America | 2018 | 8555.869162 | 9556.467237 | 7648.13237 |
| Prevalence | Southern Latin America | 2019 | 8602.744174 | 9564.43021 | 7719.554954 |
| Prevalence | Southern Sub-Saharan Africa | 1990 | 16003.49269 | 17873.10295 | 14363.86257 |
| Prevalence | Southern Sub-Saharan Africa | 1991 | 16094.51504 | 17975.82855 | 14456.95426 |
| Prevalence | Southern Sub-Saharan Africa | 1992 | 16184.22002 | 18088.33462 | 14527.63125 |
| Prevalence | Southern Sub-Saharan Africa | 1993 | 16269.83384 | 18188.72397 | 14582.808 |
| Prevalence | Southern Sub-Saharan Africa | 1994 | 16348.34809 | 18269.46466 | 14662.46984 |
| Prevalence | Southern Sub-Saharan Africa | 1995 | 16415.58394 | 18342.03908 | 14705.46898 |
| Prevalence | Southern Sub-Saharan Africa | 1996 | 16484.19268 | 18409.43952 | 14783.62038 |
| Prevalence | Southern Sub-Saharan Africa | 1997 | 16565.37771 | 18491.40715 | 14871.3917 |
| Prevalence | Southern Sub-Saharan Africa | 1998 | 16649.07741 | 18579.19285 | 14954.41327 |
| Prevalence | Southern Sub-Saharan Africa | 1999 | 16726.6731 | 18662.16747 | 15033.60441 |
| Prevalence | Southern Sub-Saharan Africa | 2000 | 16788.59498 | 18727.9415 | 15073.88355 |
| Prevalence | Southern Sub-Saharan Africa | 2001 | 16841.53558 | 18779.07612 | 15126.49036 |
| Prevalence | Southern Sub-Saharan Africa | 2002 | 16899.4255 | 18829.78139 | 15183.02832 |
| Prevalence | Southern Sub-Saharan Africa | 2003 | 16959.55662 | 18878.89252 | 15253.75762 |
| Prevalence | Southern Sub-Saharan Africa | 2004 | 17019.10029 | 18927.17738 | 15323.93984 |
| Prevalence | Southern Sub-Saharan Africa | 2005 | 17074.84885 | 18982.96844 | 15390.85961 |
| Prevalence | Southern Sub-Saharan Africa | 2006 | 17125.79383 | 19045.2593 | 15433.78914 |
| Prevalence | Southern Sub-Saharan Africa | 2007 | 17176.02119 | 19081.29537 | 15493.64545 |
| Prevalence | Southern Sub-Saharan Africa | 2008 | 17229.09049 | 19121.2764 | 15560.78581 |
| Prevalence | Southern Sub-Saharan Africa | 2009 | 17288.49932 | 19184.22775 | 15601.0404 |
| Prevalence | Southern Sub-Saharan Africa | 2010 | 17357.77736 | 19245.74789 | 15651.93129 |
| Prevalence | Southern Sub-Saharan Africa | 2011 | 17443.97326 | 19345.70943 | 15756.80156 |
| Prevalence | Southern Sub-Saharan Africa | 2012 | 17544.74455 | 19484.2485 | 15843.05871 |
| Prevalence | Southern Sub-Saharan Africa | 2013 | 17649.1169 | 19582.33106 | 15971.01292 |
| Prevalence | Southern Sub-Saharan Africa | 2014 | 17745.93199 | 19673.81774 | 16063.65762 |
| Prevalence | Southern Sub-Saharan Africa | 2015 | 17823.28285 | 19771.59575 | 16131.49058 |
| Prevalence | Southern Sub-Saharan Africa | 2016 | 17896.79784 | 19846.0281 | 16173.69761 |
| Prevalence | Southern Sub-Saharan Africa | 2017 | 17959.76468 | 19919.20416 | 16191.15672 |
| Prevalence | Southern Sub-Saharan Africa | 2018 | 18013.53627 | 19942.1605 | 16257.02993 |
| Prevalence | Southern Sub-Saharan Africa | 2019 | 18076.65849 | 19998.37239 | 16348.66978 |
| Prevalence | Tropical Latin America | 1990 | 13164.99107 | 14662.83235 | 11825.59248 |
| Prevalence | Tropical Latin America | 1991 | 13272.78078 | 14744.58459 | 11932.68532 |
| Prevalence | Tropical Latin America | 1992 | 13377.86899 | 14863.56535 | 12006.13387 |
| Prevalence | Tropical Latin America | 1993 | 13476.57858 | 14973.76303 | 12090.62715 |
| Prevalence | Tropical Latin America | 1994 | 13565.23035 | 15073.40304 | 12188.24514 |
| Prevalence | Tropical Latin America | 1995 | 13640.20788 | 15168.07207 | 12269.34475 |
| Prevalence | Tropical Latin America | 1996 | 13711.41301 | 15236.13791 | 12336.2737 |
| Prevalence | Tropical Latin America | 1997 | 13787.96523 | 15321.67886 | 12403.53725 |
| Prevalence | Tropical Latin America | 1998 | 13865.31409 | 15412.22066 | 12468.20989 |
| Prevalence | Tropical Latin America | 1999 | 13938.83926 | 15501.9999 | 12518.51902 |
| Prevalence | Tropical Latin America | 2000 | 14003.94924 | 15567.94622 | 12578.41715 |
| Prevalence | Tropical Latin America | 2001 | 14066.82388 | 15644.85451 | 12626.22645 |
| Prevalence | Tropical Latin America | 2002 | 14134.11991 | 15688.34351 | 12692.64711 |
| Prevalence | Tropical Latin America | 2003 | 14202.19827 | 15732.98739 | 12762.13265 |
| Prevalence | Tropical Latin America | 2004 | 14267.40914 | 15789.51545 | 12829.24374 |
| Prevalence | Tropical Latin America | 2005 | 14326.18818 | 15851.32915 | 12891.25778 |
| Prevalence | Tropical Latin America | 2006 | 14379.98431 | 15913.55703 | 12948.28496 |
| Prevalence | Tropical Latin America | 2007 | 14433.471 | 15961.21251 | 12987.97869 |
| Prevalence | Tropical Latin America | 2008 | 14487.80907 | 16028.35991 | 13045.13713 |
| Prevalence | Tropical Latin America | 2009 | 14544.20478 | 16107.09819 | 13106.28152 |
| Prevalence | Tropical Latin America | 2010 | 14603.91846 | 16177.07599 | 13139.69586 |
| Prevalence | Tropical Latin America | 2011 | 14682.87582 | 16252.63841 | 13221.12499 |
| Prevalence | Tropical Latin America | 2012 | 14784.85773 | 16345.85349 | 13319.63259 |
| Prevalence | Tropical Latin America | 2013 | 14892.67083 | 16464.28532 | 13406.84008 |
| Prevalence | Tropical Latin America | 2014 | 14989.20707 | 16556.74098 | 13489.81773 |
| Prevalence | Tropical Latin America | 2015 | 15057.44976 | 16622.63666 | 13559.68395 |
| Prevalence | Tropical Latin America | 2016 | 15106.08228 | 16685.71204 | 13591.23162 |
| Prevalence | Tropical Latin America | 2017 | 15145.33081 | 16789.02875 | 13619.77081 |
| Prevalence | Tropical Latin America | 2018 | 15187.21995 | 16770.9546 | 13655.42369 |
| Prevalence | Tropical Latin America | 2019 | 15241.34216 | 16819.06605 | 13723.31049 |
| Prevalence | Western Europe | 1990 | 7881.021589 | 8876.980843 | 7036.730656 |
| Prevalence | Western Europe | 1991 | 7998.870348 | 9007.05146 | 7149.574816 |
| Prevalence | Western Europe | 1992 | 8113.903272 | 9129.060029 | 7260.004092 |
| Prevalence | Western Europe | 1993 | 8225.632393 | 9235.341623 | 7364.176382 |
| Prevalence | Western Europe | 1994 | 8332.950153 | 9341.582629 | 7464.262148 |
| Prevalence | Western Europe | 1995 | 8434.993976 | 9438.347245 | 7562.30074 |
| Prevalence | Western Europe | 1996 | 8534.927731 | 9552.5445 | 7653.441801 |
| Prevalence | Western Europe | 1997 | 8634.541887 | 9669.960816 | 7744.235056 |
| Prevalence | Western Europe | 1998 | 8732.650438 | 9786.295137 | 7834.592432 |
| Prevalence | Western Europe | 1999 | 8827.322657 | 9868.901015 | 7913.319275 |
| Prevalence | Western Europe | 2000 | 8917.214407 | 9960.116792 | 7997.394133 |
| Prevalence | Western Europe | 2001 | 9013.75526 | 10041.14858 | 8089.732193 |
| Prevalence | Western Europe | 2002 | 9120.889826 | 10151.80098 | 8192.229996 |
| Prevalence | Western Europe | 2003 | 9228.327278 | 10252.51893 | 8294.647004 |
| Prevalence | Western Europe | 2004 | 9325.679482 | 10344.96348 | 8393.208529 |
| Prevalence | Western Europe | 2005 | 9401.188168 | 10423.91686 | 8464.058588 |
| Prevalence | Western Europe | 2006 | 9462.388621 | 10491.20382 | 8525.185259 |
| Prevalence | Western Europe | 2007 | 9521.995262 | 10557.7467 | 8578.790327 |
| Prevalence | Western Europe | 2008 | 9579.050479 | 10622.72989 | 8629.30494 |
| Prevalence | Western Europe | 2009 | 9631.375674 | 10682.87197 | 8679.224778 |
| Prevalence | Western Europe | 2010 | 9676.92935 | 10733.27447 | 8723.210227 |
| Prevalence | Western Europe | 2011 | 9729.369382 | 10800.15479 | 8764.782512 |
| Prevalence | Western Europe | 2012 | 9793.170433 | 10873.9386 | 8813.520296 |
| Prevalence | Western Europe | 2013 | 9857.315929 | 10959.35726 | 8861.158785 |
| Prevalence | Western Europe | 2014 | 9909.902906 | 11026.98825 | 8902.779504 |
| Prevalence | Western Europe | 2015 | 9939.13741 | 11066.99588 | 8915.856247 |
| Prevalence | Western Europe | 2016 | 9895.026207 | 11036.84649 | 8889.648174 |
| Prevalence | Western Europe | 2017 | 9849.604509 | 11003.7818 | 8845.606718 |
| Prevalence | Western Europe | 2018 | 9876.952074 | 11000.94258 | 8878.662616 |
| Prevalence | Western Europe | 2019 | 9933.374262 | 11044.6787 | 8932.597636 |
| Prevalence | Western Sub-Saharan Africa | 1990 | 13163.49967 | 14781.20985 | 11718.58917 |
| Prevalence | Western Sub-Saharan Africa | 1991 | 13255.50328 | 14893.98528 | 11801.33089 |
| Prevalence | Western Sub-Saharan Africa | 1992 | 13343.63504 | 14984.627 | 11892.71635 |
| Prevalence | Western Sub-Saharan Africa | 1993 | 13427.8564 | 15070.74487 | 11975.14628 |
| Prevalence | Western Sub-Saharan Africa | 1994 | 13507.04165 | 15156.27982 | 12052.30187 |
| Prevalence | Western Sub-Saharan Africa | 1995 | 13576.90802 | 15231.46263 | 12121.74724 |
| Prevalence | Western Sub-Saharan Africa | 1996 | 13646.76628 | 15309.39642 | 12190.63497 |
| Prevalence | Western Sub-Saharan Africa | 1997 | 13718.99972 | 15391.33914 | 12253.40384 |
| Prevalence | Western Sub-Saharan Africa | 1998 | 13788.77548 | 15460.29745 | 12299.34441 |
| Prevalence | Western Sub-Saharan Africa | 1999 | 13850.67652 | 15541.66483 | 12333.70671 |
| Prevalence | Western Sub-Saharan Africa | 2000 | 13896.47542 | 15595.81974 | 12374.03362 |
| Prevalence | Western Sub-Saharan Africa | 2001 | 13938.64107 | 15634.90036 | 12403.0656 |
| Prevalence | Western Sub-Saharan Africa | 2002 | 13984.15464 | 15690.26353 | 12446.71008 |
| Prevalence | Western Sub-Saharan Africa | 2003 | 14027.07135 | 15738.54531 | 12500.15387 |
| Prevalence | Western Sub-Saharan Africa | 2004 | 14061.12934 | 15742.74489 | 12545.11062 |
| Prevalence | Western Sub-Saharan Africa | 2005 | 14078.73648 | 15756.21757 | 12574.07935 |
| Prevalence | Western Sub-Saharan Africa | 2006 | 14082.01057 | 15752.24717 | 12574.87587 |
| Prevalence | Western Sub-Saharan Africa | 2007 | 14076.82002 | 15759.60498 | 12573.23591 |
| Prevalence | Western Sub-Saharan Africa | 2008 | 14067.3427 | 15733.73458 | 12569.47347 |
| Prevalence | Western Sub-Saharan Africa | 2009 | 14057.62274 | 15724.00867 | 12566.94305 |
| Prevalence | Western Sub-Saharan Africa | 2010 | 14049.78953 | 15717.74844 | 12566.76063 |
| Prevalence | Western Sub-Saharan Africa | 2011 | 14045.1687 | 15711.30875 | 12558.52677 |
| Prevalence | Western Sub-Saharan Africa | 2012 | 14041.86329 | 15705.24707 | 12551.45032 |
| Prevalence | Western Sub-Saharan Africa | 2013 | 14041.25967 | 15706.47539 | 12534.40389 |
| Prevalence | Western Sub-Saharan Africa | 2014 | 14043.87091 | 15701.52583 | 12517.27839 |
| Prevalence | Western Sub-Saharan Africa | 2015 | 14049.06407 | 15702.02661 | 12527.41787 |
| Prevalence | Western Sub-Saharan Africa | 2016 | 14099.39304 | 15759.82055 | 12560.88489 |
| Prevalence | Western Sub-Saharan Africa | 2017 | 14164.55343 | 15822.78637 | 12623.8813 |
| Prevalence | Western Sub-Saharan Africa | 2018 | 14216.93561 | 15875.48329 | 12669.17735 |
| Prevalence | Western Sub-Saharan Africa | 2019 | 14284.25316 | 15953.2061 | 12757.25407 |
| Incidence | Andean Latin America | 1990 | 3.438249357 | 4.965922299 | 2.386002781 |
| Incidence | Andean Latin America | 1991 | 3.559972616 | 5.081474317 | 2.484989087 |
| Incidence | Andean Latin America | 1992 | 3.678333822 | 5.266004505 | 2.569554037 |
| Incidence | Andean Latin America | 1993 | 3.79123981 | 5.446210171 | 2.646312463 |
| Incidence | Andean Latin America | 1994 | 3.897000789 | 5.584580132 | 2.720986113 |
| Incidence | Andean Latin America | 1995 | 3.993464209 | 5.728762697 | 2.78351011 |
| Incidence | Andean Latin America | 1996 | 4.092527012 | 5.814726809 | 2.851821904 |
| Incidence | Andean Latin America | 1997 | 4.201258179 | 5.949413689 | 2.931481728 |
| Incidence | Andean Latin America | 1998 | 4.30845423 | 6.084689647 | 3.01933992 |
| Incidence | Andean Latin America | 1999 | 4.406556163 | 6.222411048 | 3.092257494 |
| Incidence | Andean Latin America | 2000 | 4.486447153 | 6.336057361 | 3.133379374 |
| Incidence | Andean Latin America | 2001 | 4.548434945 | 6.42153815 | 3.156351028 |
| Incidence | Andean Latin America | 2002 | 4.606494869 | 6.478550339 | 3.215238865 |
| Incidence | Andean Latin America | 2003 | 4.662325373 | 6.590940348 | 3.240780839 |
| Incidence | Andean Latin America | 2004 | 4.721887225 | 6.707014685 | 3.274693395 |
| Incidence | Andean Latin America | 2005 | 4.788730678 | 6.81386193 | 3.341369643 |
| Incidence | Andean Latin America | 2006 | 4.877206807 | 6.93328853 | 3.389794603 |
| Incidence | Andean Latin America | 2007 | 4.992196833 | 7.071394311 | 3.461124611 |
| Incidence | Andean Latin America | 2008 | 5.114552829 | 7.219807147 | 3.546897031 |
| Incidence | Andean Latin America | 2009 | 5.224143909 | 7.367846906 | 3.631706877 |
| Incidence | Andean Latin America | 2010 | 5.300477161 | 7.476861949 | 3.669245513 |
| Incidence | Andean Latin America | 2011 | 5.352500346 | 7.524449597 | 3.700397183 |
| Incidence | Andean Latin America | 2012 | 5.402733427 | 7.607750726 | 3.733127381 |
| Incidence | Andean Latin America | 2013 | 5.451245548 | 7.699693806 | 3.758419095 |
| Incidence | Andean Latin America | 2014 | 5.493663783 | 7.763127447 | 3.799997484 |
| Incidence | Andean Latin America | 2015 | 5.52991612 | 7.793128436 | 3.816967861 |
| Incidence | Andean Latin America | 2016 | 5.568049369 | 7.874274311 | 3.853812173 |
| Incidence | Andean Latin America | 2017 | 5.59890745 | 7.938154123 | 3.862399835 |
| Incidence | Andean Latin America | 2018 | 5.61460411 | 7.930055439 | 3.889434216 |
| Incidence | Andean Latin America | 2019 | 5.622021172 | 7.974340049 | 3.855783169 |
| Incidence | Australasia | 1990 | 1.396754699 | 2.003192619 | 0.952566759 |
| Incidence | Australasia | 1991 | 1.408388101 | 2.002421712 | 0.96489732 |
| Incidence | Australasia | 1992 | 1.418859288 | 2.014787732 | 0.974335015 |
| Incidence | Australasia | 1993 | 1.428351153 | 2.012316067 | 0.986631279 |
| Incidence | Australasia | 1994 | 1.437059861 | 2.019343884 | 1.00158933 |
| Incidence | Australasia | 1995 | 1.445208845 | 2.031081491 | 1.010160814 |
| Incidence | Australasia | 1996 | 1.45021186 | 2.036151433 | 1.021828757 |
| Incidence | Australasia | 1997 | 1.450846823 | 2.026583421 | 1.029130134 |
| Incidence | Australasia | 1998 | 1.449727697 | 2.004052979 | 1.033798295 |
| Incidence | Australasia | 1999 | 1.449436443 | 1.998039963 | 1.039552372 |
| Incidence | Australasia | 2000 | 1.452562986 | 2.002214982 | 1.043368201 |
| Incidence | Australasia | 2001 | 1.45733651 | 1.994447465 | 1.053071592 |
| Incidence | Australasia | 2002 | 1.461337514 | 1.998095392 | 1.060326168 |
| Incidence | Australasia | 2003 | 1.466927875 | 2.003266408 | 1.067134202 |
| Incidence | Australasia | 2004 | 1.476440372 | 2.017465602 | 1.077284991 |
| Incidence | Australasia | 2005 | 1.492093084 | 2.037096244 | 1.092701254 |
| Incidence | Australasia | 2006 | 1.526253865 | 2.077828902 | 1.119782666 |
| Incidence | Australasia | 2007 | 1.580648129 | 2.149511575 | 1.155776427 |
| Incidence | Australasia | 2008 | 1.641880604 | 2.231804265 | 1.193877515 |
| Incidence | Australasia | 2009 | 1.696629585 | 2.304181227 | 1.235812215 |
| Incidence | Australasia | 2010 | 1.731402567 | 2.363302504 | 1.260656567 |
| Incidence | Australasia | 2011 | 1.753592926 | 2.367775471 | 1.281837509 |
| Incidence | Australasia | 2012 | 1.777026111 | 2.386715295 | 1.3059191 |
| Incidence | Australasia | 2013 | 1.797863797 | 2.398729698 | 1.323219573 |
| Incidence | Australasia | 2014 | 1.812298597 | 2.428260481 | 1.337277651 |
| Incidence | Australasia | 2015 | 1.816601873 | 2.458196694 | 1.34423741 |
| Incidence | Australasia | 2016 | 1.781577944 | 2.413822407 | 1.318426253 |
| Incidence | Australasia | 2017 | 1.743090752 | 2.361107387 | 1.274498566 |
| Incidence | Australasia | 2018 | 1.738233768 | 2.378261469 | 1.279819422 |
| Incidence | Australasia | 2019 | 1.744045234 | 2.4039773 | 1.269176388 |
| Incidence | Caribbean | 1990 | 2.739416084 | 3.866683165 | 1.946781639 |
| Incidence | Caribbean | 1991 | 2.742742044 | 3.871864312 | 1.926720512 |
| Incidence | Caribbean | 1992 | 2.742315235 | 3.851627369 | 1.933903812 |
| Incidence | Caribbean | 1993 | 2.739122661 | 3.856386987 | 1.929785578 |
| Incidence | Caribbean | 1994 | 2.732938466 | 3.861740872 | 1.922176205 |
| Incidence | Caribbean | 1995 | 2.724513345 | 3.866650242 | 1.914800966 |
| Incidence | Caribbean | 1996 | 2.707322664 | 3.85758024 | 1.897886927 |
| Incidence | Caribbean | 1997 | 2.677463245 | 3.826805189 | 1.853705351 |
| Incidence | Caribbean | 1998 | 2.644295897 | 3.79060048 | 1.818874591 |
| Incidence | Caribbean | 1999 | 2.615622412 | 3.764282964 | 1.777463302 |
| Incidence | Caribbean | 2000 | 2.599983993 | 3.77599831 | 1.755694894 |
| Incidence | Caribbean | 2001 | 2.592966191 | 3.749986248 | 1.749233642 |
| Incidence | Caribbean | 2002 | 2.585195452 | 3.742148574 | 1.743994636 |
| Incidence | Caribbean | 2003 | 2.579015654 | 3.735249756 | 1.747624546 |
| Incidence | Caribbean | 2004 | 2.57542245 | 3.733813425 | 1.753539954 |
| Incidence | Caribbean | 2005 | 2.575704258 | 3.736463417 | 1.750196063 |
| Incidence | Caribbean | 2006 | 2.580577247 | 3.74363913 | 1.754431775 |
| Incidence | Caribbean | 2007 | 2.590630231 | 3.762305863 | 1.76268307 |
| Incidence | Caribbean | 2008 | 2.603725449 | 3.759158397 | 1.777691708 |
| Incidence | Caribbean | 2009 | 2.617683742 | 3.762193297 | 1.788215542 |
| Incidence | Caribbean | 2010 | 2.629341123 | 3.782434391 | 1.80142774 |
| Incidence | Caribbean | 2011 | 2.641281821 | 3.810665215 | 1.81317931 |
| Incidence | Caribbean | 2012 | 2.659218082 | 3.840465042 | 1.824775343 |
| Incidence | Caribbean | 2013 | 2.680237455 | 3.875465361 | 1.842081225 |
| Incidence | Caribbean | 2014 | 2.702754274 | 3.90213332 | 1.85599642 |
| Incidence | Caribbean | 2015 | 2.725177532 | 3.946590063 | 1.875202512 |
| Incidence | Caribbean | 2016 | 2.773525318 | 4.008592394 | 1.900517967 |
| Incidence | Caribbean | 2017 | 2.826883437 | 4.081902158 | 1.934520869 |
| Incidence | Caribbean | 2018 | 2.861783512 | 4.155209316 | 1.96501233 |
| Incidence | Caribbean | 2019 | 2.897986118 | 4.222712907 | 1.997177963 |
| Incidence | Central Asia | 1990 | 1.807428227 | 2.740718109 | 1.175312217 |
| Incidence | Central Asia | 1991 | 1.863671319 | 2.805954896 | 1.228900638 |
| Incidence | Central Asia | 1992 | 1.921694596 | 2.843073373 | 1.29771873 |
| Incidence | Central Asia | 1993 | 1.9773852 | 2.919085623 | 1.341612295 |
| Incidence | Central Asia | 1994 | 2.02824436 | 2.96600907 | 1.378917679 |
| Incidence | Central Asia | 1995 | 2.074008122 | 3.032983206 | 1.404098788 |
| Incidence | Central Asia | 1996 | 2.141219748 | 3.115641111 | 1.453112148 |
| Incidence | Central Asia | 1997 | 2.232443991 | 3.256806276 | 1.524679324 |
| Incidence | Central Asia | 1998 | 2.330333614 | 3.406792908 | 1.599361466 |
| Incidence | Central Asia | 1999 | 2.422120422 | 3.531537694 | 1.669067218 |
| Incidence | Central Asia | 2000 | 2.498317318 | 3.636389234 | 1.722914583 |
| Incidence | Central Asia | 2001 | 2.575981801 | 3.762742856 | 1.776584958 |
| Incidence | Central Asia | 2002 | 2.658269052 | 3.86905156 | 1.836093908 |
| Incidence | Central Asia | 2003 | 2.739336739 | 3.975722871 | 1.898084384 |
| Incidence | Central Asia | 2004 | 2.817279257 | 4.092870347 | 1.953472771 |
| Incidence | Central Asia | 2005 | 2.89296447 | 4.196519593 | 2.002072222 |
| Incidence | Central Asia | 2006 | 2.993057179 | 4.337374988 | 2.075473092 |
| Incidence | Central Asia | 2007 | 3.128259943 | 4.535421596 | 2.173517472 |
| Incidence | Central Asia | 2008 | 3.276324695 | 4.753908766 | 2.28069953 |
| Incidence | Central Asia | 2009 | 3.417246076 | 4.978291005 | 2.379923333 |
| Incidence | Central Asia | 2010 | 3.531413065 | 5.160815835 | 2.466409564 |
| Incidence | Central Asia | 2011 | 3.624410569 | 5.270578582 | 2.53354728 |
| Incidence | Central Asia | 2012 | 3.723297331 | 5.403866094 | 2.582664343 |
| Incidence | Central Asia | 2013 | 3.821227493 | 5.537476963 | 2.64856848 |
| Incidence | Central Asia | 2014 | 3.912518596 | 5.661458102 | 2.709938861 |
| Incidence | Central Asia | 2015 | 3.990925454 | 5.763775372 | 2.76140843 |
| Incidence | Central Asia | 2016 | 4.040189414 | 5.837008406 | 2.793563779 |
| Incidence | Central Asia | 2017 | 4.068295658 | 5.885923274 | 2.80546158 |
| Incidence | Central Asia | 2018 | 4.117454741 | 5.962074823 | 2.822632772 |
| Incidence | Central Asia | 2019 | 4.191043672 | 6.076083791 | 2.866779694 |
| Incidence | Central Europe | 1990 | 1.452879227 | 2.061889164 | 1.042114869 |
| Incidence | Central Europe | 1991 | 1.447527768 | 2.060264568 | 1.03613962 |
| Incidence | Central Europe | 1992 | 1.440960645 | 2.054917683 | 1.028299527 |
| Incidence | Central Europe | 1993 | 1.433525366 | 2.050427316 | 1.019957947 |
| Incidence | Central Europe | 1994 | 1.425382481 | 2.04223138 | 1.009869919 |
| Incidence | Central Europe | 1995 | 1.416843461 | 2.034623732 | 0.997259669 |
| Incidence | Central Europe | 1996 | 1.404353941 | 2.02314574 | 0.982010546 |
| Incidence | Central Europe | 1997 | 1.38590592 | 2.007319069 | 0.964557404 |
| Incidence | Central Europe | 1998 | 1.368565367 | 1.992953089 | 0.945695776 |
| Incidence | Central Europe | 1999 | 1.355671855 | 1.984987251 | 0.92943771 |
| Incidence | Central Europe | 2000 | 1.352757868 | 1.993631819 | 0.920773851 |
| Incidence | Central Europe | 2001 | 1.363414159 | 2.011100888 | 0.929473026 |
| Incidence | Central Europe | 2002 | 1.38111717 | 2.037541036 | 0.942802936 |
| Incidence | Central Europe | 2003 | 1.402913953 | 2.073435605 | 0.95909256 |
| Incidence | Central Europe | 2004 | 1.420412895 | 2.098270734 | 0.971163823 |
| Incidence | Central Europe | 2005 | 1.427256661 | 2.108070092 | 0.976195999 |
| Incidence | Central Europe | 2006 | 1.426643023 | 2.100890623 | 0.974085321 |
| Incidence | Central Europe | 2007 | 1.425495287 | 2.10279606 | 0.972651333 |
| Incidence | Central Europe | 2008 | 1.426353022 | 2.108003959 | 0.976418994 |
| Incidence | Central Europe | 2009 | 1.425323538 | 2.110784341 | 0.977882857 |
| Incidence | Central Europe | 2010 | 1.421458102 | 2.103499065 | 0.975916363 |
| Incidence | Central Europe | 2011 | 1.413174704 | 2.092215198 | 0.969530847 |
| Incidence | Central Europe | 2012 | 1.401471243 | 2.074551455 | 0.960609775 |
| Incidence | Central Europe | 2013 | 1.389601319 | 2.053649516 | 0.949980663 |
| Incidence | Central Europe | 2014 | 1.375578021 | 2.038586578 | 0.939554081 |
| Incidence | Central Europe | 2015 | 1.361616153 | 2.025711679 | 0.926369279 |
| Incidence | Central Europe | 2016 | 1.341333887 | 1.994792801 | 0.917462018 |
| Incidence | Central Europe | 2017 | 1.323433356 | 1.971379182 | 0.901487762 |
| Incidence | Central Europe | 2018 | 1.311755281 | 1.941525765 | 0.897939139 |
| Incidence | Central Europe | 2019 | 1.29803076 | 1.927351756 | 0.888033541 |
| Incidence | Central Latin America | 1990 | 6.108476957 | 9.057819422 | 4.028528107 |
| Incidence | Central Latin America | 1991 | 6.081101442 | 8.965750917 | 4.041634608 |
| Incidence | Central Latin America | 1992 | 6.058099841 | 8.870795131 | 4.057187353 |
| Incidence | Central Latin America | 1993 | 6.039844279 | 8.872848822 | 4.054332858 |
| Incidence | Central Latin America | 1994 | 6.02599553 | 8.911585953 | 4.04646653 |
| Incidence | Central Latin America | 1995 | 6.019161088 | 8.867790068 | 4.101248954 |
| Incidence | Central Latin America | 1996 | 6.018133958 | 8.851816153 | 4.117072325 |
| Incidence | Central Latin America | 1997 | 6.014248708 | 8.824108954 | 4.130120624 |
| Incidence | Central Latin America | 1998 | 6.010399188 | 8.793574054 | 4.132456206 |
| Incidence | Central Latin America | 1999 | 6.010945737 | 8.776424827 | 4.13688436 |
| Incidence | Central Latin America | 2000 | 6.019686377 | 8.779723668 | 4.144287392 |
| Incidence | Central Latin America | 2001 | 6.038746444 | 8.72629367 | 4.166945738 |
| Incidence | Central Latin America | 2002 | 6.065989627 | 8.779422828 | 4.191715027 |
| Incidence | Central Latin America | 2003 | 6.100493024 | 8.841414032 | 4.219109205 |
| Incidence | Central Latin America | 2004 | 6.143016576 | 8.931277058 | 4.268179047 |
| Incidence | Central Latin America | 2005 | 6.192694737 | 9.039448851 | 4.313629727 |
| Incidence | Central Latin America | 2006 | 6.292123367 | 9.157957834 | 4.38933812 |
| Incidence | Central Latin America | 2007 | 6.459943197 | 9.353067042 | 4.529900223 |
| Incidence | Central Latin America | 2008 | 6.647838162 | 9.570607264 | 4.638930827 |
| Incidence | Central Latin America | 2009 | 6.809788604 | 9.823238312 | 4.762679435 |
| Incidence | Central Latin America | 2010 | 6.895777236 | 9.933334383 | 4.818728354 |
| Incidence | Central Latin America | 2011 | 6.901166105 | 9.949996936 | 4.826203062 |
| Incidence | Central Latin America | 2012 | 6.880728875 | 9.934977569 | 4.816048204 |
| Incidence | Central Latin America | 2013 | 6.852423 | 9.904110015 | 4.792099329 |
| Incidence | Central Latin America | 2014 | 6.834844088 | 9.861819304 | 4.778362293 |
| Incidence | Central Latin America | 2015 | 6.846066316 | 9.903511774 | 4.788493672 |
| Incidence | Central Latin America | 2016 | 6.925799473 | 10.03828532 | 4.823552549 |
| Incidence | Central Latin America | 2017 | 7.011045208 | 10.17517041 | 4.823535015 |
| Incidence | Central Latin America | 2018 | 6.988799851 | 10.14843971 | 4.792560289 |
| Incidence | Central Latin America | 2019 | 6.878561598 | 9.979098979 | 4.69925608 |
| Incidence | Central Sub-Saharan Africa | 1990 | 1.08515822 | 1.68865666 | 0.701573451 |
| Incidence | Central Sub-Saharan Africa | 1991 | 1.080865915 | 1.691085245 | 0.705353665 |
| Incidence | Central Sub-Saharan Africa | 1992 | 1.077498841 | 1.670330518 | 0.702487167 |
| Incidence | Central Sub-Saharan Africa | 1993 | 1.07512206 | 1.643112296 | 0.70687955 |
| Incidence | Central Sub-Saharan Africa | 1994 | 1.073841437 | 1.623917259 | 0.700647662 |
| Incidence | Central Sub-Saharan Africa | 1995 | 1.073723771 | 1.62283208 | 0.705904276 |
| Incidence | Central Sub-Saharan Africa | 1996 | 1.07507484 | 1.627392623 | 0.705709322 |
| Incidence | Central Sub-Saharan Africa | 1997 | 1.078099511 | 1.639473044 | 0.704168433 |
| Incidence | Central Sub-Saharan Africa | 1998 | 1.08210503 | 1.66146823 | 0.710961006 |
| Incidence | Central Sub-Saharan Africa | 1999 | 1.086263632 | 1.662150767 | 0.711129432 |
| Incidence | Central Sub-Saharan Africa | 2000 | 1.089625512 | 1.675125567 | 0.714337288 |
| Incidence | Central Sub-Saharan Africa | 2001 | 1.094040267 | 1.674176905 | 0.718082471 |
| Incidence | Central Sub-Saharan Africa | 2002 | 1.100456491 | 1.679985494 | 0.726719862 |
| Incidence | Central Sub-Saharan Africa | 2003 | 1.107271626 | 1.683672269 | 0.739141136 |
| Incidence | Central Sub-Saharan Africa | 2004 | 1.112872334 | 1.693327969 | 0.744773689 |
| Incidence | Central Sub-Saharan Africa | 2005 | 1.11515235 | 1.685636307 | 0.746068091 |
| Incidence | Central Sub-Saharan Africa | 2006 | 1.113817271 | 1.686718866 | 0.744165706 |
| Incidence | Central Sub-Saharan Africa | 2007 | 1.109486902 | 1.668942166 | 0.742749025 |
| Incidence | Central Sub-Saharan Africa | 2008 | 1.104583811 | 1.652706739 | 0.735761847 |
| Incidence | Central Sub-Saharan Africa | 2009 | 1.101417566 | 1.645693851 | 0.734496232 |
| Incidence | Central Sub-Saharan Africa | 2010 | 1.102612197 | 1.648081726 | 0.734219305 |
| Incidence | Central Sub-Saharan Africa | 2011 | 1.10732065 | 1.660819212 | 0.742960069 |
| Incidence | Central Sub-Saharan Africa | 2012 | 1.11414281 | 1.675934941 | 0.753521107 |
| Incidence | Central Sub-Saharan Africa | 2013 | 1.123931561 | 1.68312155 | 0.757888185 |
| Incidence | Central Sub-Saharan Africa | 2014 | 1.137655764 | 1.705663023 | 0.766143322 |
| Incidence | Central Sub-Saharan Africa | 2015 | 1.156268265 | 1.749045722 | 0.779871417 |
| Incidence | Central Sub-Saharan Africa | 2016 | 1.224650461 | 1.825348679 | 0.812561066 |
| Incidence | Central Sub-Saharan Africa | 2017 | 1.302248352 | 1.965111292 | 0.835363759 |
| Incidence | Central Sub-Saharan Africa | 2018 | 1.341431947 | 2.021830562 | 0.871195685 |
| Incidence | Central Sub-Saharan Africa | 2019 | 1.37272 | 2.101647119 | 0.891191921 |
| Incidence | East Asia | 1990 | 2.672498571 | 3.646511111 | 1.958400641 |
| Incidence | East Asia | 1991 | 2.648216473 | 3.57020886 | 1.970592081 |
| Incidence | East Asia | 1992 | 2.62226946 | 3.499496148 | 1.965596494 |
| Incidence | East Asia | 1993 | 2.596672851 | 3.439310121 | 1.960946088 |
| Incidence | East Asia | 1994 | 2.572919683 | 3.383892411 | 1.947915299 |
| Incidence | East Asia | 1995 | 2.553781137 | 3.340418374 | 1.945134005 |
| Incidence | East Asia | 1996 | 2.535795398 | 3.320537549 | 1.941605497 |
| Incidence | East Asia | 1997 | 2.513824013 | 3.300147329 | 1.92775458 |
| Incidence | East Asia | 1998 | 2.486531082 | 3.277160756 | 1.899218612 |
| Incidence | East Asia | 1999 | 2.452477375 | 3.255788863 | 1.872307538 |
| Incidence | East Asia | 2000 | 2.411121083 | 3.221522008 | 1.826581156 |
| Incidence | East Asia | 2001 | 2.328606901 | 3.122302693 | 1.763682001 |
| Incidence | East Asia | 2002 | 2.196482633 | 2.962916428 | 1.643405539 |
| Incidence | East Asia | 2003 | 2.051095887 | 2.819156256 | 1.512952339 |
| Incidence | East Asia | 2004 | 1.929089572 | 2.692818874 | 1.395540627 |
| Incidence | East Asia | 2005 | 1.867063886 | 2.627164137 | 1.332072939 |
| Incidence | East Asia | 2006 | 1.846902419 | 2.596815566 | 1.321146497 |
| Incidence | East Asia | 2007 | 1.828621961 | 2.577242551 | 1.31808278 |
| Incidence | East Asia | 2008 | 1.815553656 | 2.563865165 | 1.319012097 |
| Incidence | East Asia | 2009 | 1.811308432 | 2.550577926 | 1.317511813 |
| Incidence | East Asia | 2010 | 1.818884383 | 2.555726822 | 1.324428066 |
| Incidence | East Asia | 2011 | 1.841115708 | 2.580746679 | 1.342475998 |
| Incidence | East Asia | 2012 | 1.875067503 | 2.634191958 | 1.369100199 |
| Incidence | East Asia | 2013 | 1.91529916 | 2.679808429 | 1.39872207 |
| Incidence | East Asia | 2014 | 1.957111231 | 2.73715601 | 1.425581669 |
| Incidence | East Asia | 2015 | 1.995272326 | 2.797632882 | 1.450522161 |
| Incidence | East Asia | 2016 | 2.046163151 | 2.860289736 | 1.489626197 |
| Incidence | East Asia | 2017 | 2.086814698 | 2.911635057 | 1.513586563 |
| Incidence | East Asia | 2018 | 2.09808691 | 2.930640452 | 1.520559098 |
| Incidence | East Asia | 2019 | 2.099931809 | 2.933018292 | 1.505046408 |
| Incidence | Eastern Europe | 1990 | 1.175817218 | 1.882817933 | 0.740443011 |
| Incidence | Eastern Europe | 1991 | 1.176634681 | 1.872003991 | 0.746765731 |
| Incidence | Eastern Europe | 1992 | 1.181481552 | 1.869483196 | 0.751480089 |
| Incidence | Eastern Europe | 1993 | 1.190589614 | 1.890071543 | 0.759127656 |
| Incidence | Eastern Europe | 1994 | 1.205499153 | 1.927629245 | 0.767350194 |
| Incidence | Eastern Europe | 1995 | 1.224508725 | 1.968738556 | 0.777174186 |
| Incidence | Eastern Europe | 1996 | 1.253645073 | 2.014650119 | 0.788639901 |
| Incidence | Eastern Europe | 1997 | 1.295342648 | 2.083148718 | 0.805117204 |
| Incidence | Eastern Europe | 1998 | 1.343409652 | 2.175635998 | 0.831558343 |
| Incidence | Eastern Europe | 1999 | 1.391426931 | 2.268681064 | 0.854067895 |
| Incidence | Eastern Europe | 2000 | 1.430453868 | 2.333943667 | 0.877896501 |
| Incidence | Eastern Europe | 2001 | 1.461946102 | 2.38211472 | 0.904522272 |
| Incidence | Eastern Europe | 2002 | 1.491799552 | 2.424886485 | 0.92022342 |
| Incidence | Eastern Europe | 2003 | 1.522126063 | 2.474210278 | 0.935394897 |
| Incidence | Eastern Europe | 2004 | 1.554687022 | 2.518483446 | 0.959613656 |
| Incidence | Eastern Europe | 2005 | 1.591109987 | 2.563033478 | 0.986227184 |
| Incidence | Eastern Europe | 2006 | 1.641013263 | 2.635927667 | 1.020421718 |
| Incidence | Eastern Europe | 2007 | 1.705303425 | 2.747011503 | 1.054158821 |
| Incidence | Eastern Europe | 2008 | 1.77422673 | 2.858655731 | 1.095790302 |
| Incidence | Eastern Europe | 2009 | 1.837881825 | 2.96501016 | 1.134822635 |
| Incidence | Eastern Europe | 2010 | 1.886373454 | 3.050951406 | 1.168016005 |
| Incidence | Eastern Europe | 2011 | 1.92605261 | 3.112369739 | 1.186829035 |
| Incidence | Eastern Europe | 2012 | 1.96683601 | 3.164190938 | 1.207380253 |
| Incidence | Eastern Europe | 2013 | 2.004266112 | 3.208861386 | 1.226018721 |
| Incidence | Eastern Europe | 2014 | 2.033655544 | 3.246312188 | 1.238843311 |
| Incidence | Eastern Europe | 2015 | 2.051034222 | 3.274046378 | 1.244783618 |
| Incidence | Eastern Europe | 2016 | 2.042898851 | 3.314925254 | 1.239050846 |
| Incidence | Eastern Europe | 2017 | 2.027413489 | 3.317840878 | 1.21091346 |
| Incidence | Eastern Europe | 2018 | 2.026729457 | 3.319004386 | 1.212818432 |
| Incidence | Eastern Europe | 2019 | 2.037572768 | 3.318841107 | 1.224105772 |
| Incidence | Eastern Sub-Saharan Africa | 1990 | 1.914387274 | 2.925874431 | 1.182454176 |
| Incidence | Eastern Sub-Saharan Africa | 1991 | 1.913328633 | 2.893945106 | 1.191484619 |
| Incidence | Eastern Sub-Saharan Africa | 1992 | 1.912163693 | 2.880113093 | 1.203526413 |
| Incidence | Eastern Sub-Saharan Africa | 1993 | 1.910227148 | 2.874664461 | 1.21365735 |
| Incidence | Eastern Sub-Saharan Africa | 1994 | 1.91084804 | 2.859351626 | 1.225979898 |
| Incidence | Eastern Sub-Saharan Africa | 1995 | 1.914853191 | 2.855302953 | 1.23933658 |
| Incidence | Eastern Sub-Saharan Africa | 1996 | 1.922031376 | 2.853054679 | 1.250370095 |
| Incidence | Eastern Sub-Saharan Africa | 1997 | 1.931560626 | 2.864527878 | 1.259841629 |
| Incidence | Eastern Sub-Saharan Africa | 1998 | 1.941591121 | 2.877263966 | 1.266312932 |
| Incidence | Eastern Sub-Saharan Africa | 1999 | 1.95163718 | 2.889807342 | 1.280811859 |
| Incidence | Eastern Sub-Saharan Africa | 2000 | 1.959992696 | 2.900622107 | 1.286739581 |
| Incidence | Eastern Sub-Saharan Africa | 2001 | 1.96792709 | 2.912275056 | 1.287725938 |
| Incidence | Eastern Sub-Saharan Africa | 2002 | 1.976925077 | 2.925652874 | 1.292190251 |
| Incidence | Eastern Sub-Saharan Africa | 2003 | 1.985876092 | 2.94014677 | 1.292480127 |
| Incidence | Eastern Sub-Saharan Africa | 2004 | 1.993936963 | 2.956099669 | 1.294292377 |
| Incidence | Eastern Sub-Saharan Africa | 2005 | 1.999763661 | 2.969712193 | 1.302438645 |
| Incidence | Eastern Sub-Saharan Africa | 2006 | 2.003072668 | 2.96648673 | 1.309107364 |
| Incidence | Eastern Sub-Saharan Africa | 2007 | 2.004259193 | 2.95854935 | 1.315844591 |
| Incidence | Eastern Sub-Saharan Africa | 2008 | 2.005062526 | 2.957243845 | 1.323036404 |
| Incidence | Eastern Sub-Saharan Africa | 2009 | 2.007164798 | 2.947038291 | 1.330228067 |
| Incidence | Eastern Sub-Saharan Africa | 2010 | 2.012095268 | 2.942894239 | 1.330961695 |
| Incidence | Eastern Sub-Saharan Africa | 2011 | 2.027642786 | 2.976365776 | 1.338779082 |
| Incidence | Eastern Sub-Saharan Africa | 2012 | 2.055762369 | 3.032899288 | 1.357238402 |
| Incidence | Eastern Sub-Saharan Africa | 2013 | 2.090429805 | 3.089522273 | 1.378026258 |
| Incidence | Eastern Sub-Saharan Africa | 2014 | 2.125371217 | 3.147265257 | 1.400404382 |
| Incidence | Eastern Sub-Saharan Africa | 2015 | 2.154184679 | 3.187930677 | 1.418202802 |
| Incidence | Eastern Sub-Saharan Africa | 2016 | 2.191018969 | 3.242587044 | 1.416115167 |
| Incidence | Eastern Sub-Saharan Africa | 2017 | 2.228822062 | 3.359583289 | 1.417849861 |
| Incidence | Eastern Sub-Saharan Africa | 2018 | 2.26484683 | 3.421769323 | 1.444942911 |
| Incidence | Eastern Sub-Saharan Africa | 2019 | 2.312390008 | 3.498572416 | 1.48225601 |
| Incidence | Global | 1990 | 1.938241845 | 2.768330014 | 1.381595027 |
| Incidence | Global | 1991 | 1.946826337 | 2.76413417 | 1.402345728 |
| Incidence | Global | 1992 | 1.954296585 | 2.759298296 | 1.417642945 |
| Incidence | Global | 1993 | 1.960650421 | 2.755199413 | 1.436025788 |
| Incidence | Global | 1994 | 1.965975223 | 2.747268195 | 1.444182614 |
| Incidence | Global | 1995 | 1.970645338 | 2.752189829 | 1.455459926 |
| Incidence | Global | 1996 | 1.97358118 | 2.744677755 | 1.464643566 |
| Incidence | Global | 1997 | 1.973694161 | 2.733281206 | 1.470605092 |
| Incidence | Global | 1998 | 1.971278492 | 2.719559282 | 1.470143445 |
| Incidence | Global | 1999 | 1.966389655 | 2.713191152 | 1.467267472 |
| Incidence | Global | 2000 | 1.959080737 | 2.709819702 | 1.461313578 |
| Incidence | Global | 2001 | 1.942772133 | 2.693601161 | 1.45052228 |
| Incidence | Global | 2002 | 1.914628652 | 2.659808839 | 1.426498741 |
| Incidence | Global | 2003 | 1.883911318 | 2.616313897 | 1.397905276 |
| Incidence | Global | 2004 | 1.859380005 | 2.57998069 | 1.372776529 |
| Incidence | Global | 2005 | 1.849516465 | 2.562897228 | 1.361835085 |
| Incidence | Global | 2006 | 1.853227532 | 2.567510519 | 1.371065617 |
| Incidence | Global | 2007 | 1.861693669 | 2.577253491 | 1.383209073 |
| Incidence | Global | 2008 | 1.872974618 | 2.589504507 | 1.395131761 |
| Incidence | Global | 2009 | 1.88556702 | 2.603555614 | 1.40467187 |
| Incidence | Global | 2010 | 1.898134353 | 2.619265658 | 1.413404264 |
| Incidence | Global | 2011 | 1.913598422 | 2.638398156 | 1.423690695 |
| Incidence | Global | 2012 | 1.934823153 | 2.666340106 | 1.43892565 |
| Incidence | Global | 2013 | 1.958735621 | 2.698461059 | 1.457136779 |
| Incidence | Global | 2014 | 1.982623632 | 2.731039504 | 1.473894197 |
| Incidence | Global | 2015 | 2.003649807 | 2.762990029 | 1.483321523 |
| Incidence | Global | 2016 | 2.034196779 | 2.834227898 | 1.491107017 |
| Incidence | Global | 2017 | 2.061923251 | 2.903263935 | 1.509801649 |
| Incidence | Global | 2018 | 2.073072619 | 2.907385611 | 1.514556393 |
| Incidence | Global | 2019 | 2.07942373 | 2.925991658 | 1.517317984 |
| Incidence | High-income Asia Pacific | 1990 | 1.385415029 | 1.837372053 | 1.070150826 |
| Incidence | High-income Asia Pacific | 1991 | 1.417866172 | 1.857230488 | 1.106640306 |
| Incidence | High-income Asia Pacific | 1992 | 1.448473995 | 1.885281808 | 1.137629725 |
| Incidence | High-income Asia Pacific | 1993 | 1.47632118 | 1.926216336 | 1.163855078 |
| Incidence | High-income Asia Pacific | 1994 | 1.500299943 | 1.936077788 | 1.191489372 |
| Incidence | High-income Asia Pacific | 1995 | 1.519430155 | 1.95900624 | 1.21499906 |
| Incidence | High-income Asia Pacific | 1996 | 1.546872256 | 1.971675341 | 1.247562894 |
| Incidence | High-income Asia Pacific | 1997 | 1.588399725 | 2.002659467 | 1.29668414 |
| Incidence | High-income Asia Pacific | 1998 | 1.631666417 | 2.042569011 | 1.336818231 |
| Incidence | High-income Asia Pacific | 1999 | 1.663833479 | 2.063830084 | 1.380054356 |
| Incidence | High-income Asia Pacific | 2000 | 1.671973754 | 2.058223898 | 1.393041607 |
| Incidence | High-income Asia Pacific | 2001 | 1.660298345 | 2.038660891 | 1.380323814 |
| Incidence | High-income Asia Pacific | 2002 | 1.641709733 | 2.013623937 | 1.360076486 |
| Incidence | High-income Asia Pacific | 2003 | 1.620403242 | 1.991483153 | 1.342041917 |
| Incidence | High-income Asia Pacific | 2004 | 1.598000641 | 1.966630616 | 1.313315841 |
| Incidence | High-income Asia Pacific | 2005 | 1.577349726 | 1.945888176 | 1.290735384 |
| Incidence | High-income Asia Pacific | 2006 | 1.551827416 | 1.912934334 | 1.261862469 |
| Incidence | High-income Asia Pacific | 2007 | 1.516444297 | 1.868024062 | 1.233565921 |
| Incidence | High-income Asia Pacific | 2008 | 1.47784218 | 1.822190854 | 1.20315896 |
| Incidence | High-income Asia Pacific | 2009 | 1.442688388 | 1.781555077 | 1.17600053 |
| Incidence | High-income Asia Pacific | 2010 | 1.417203536 | 1.751731809 | 1.154208137 |
| Incidence | High-income Asia Pacific | 2011 | 1.400628804 | 1.729932044 | 1.134717109 |
| Incidence | High-income Asia Pacific | 2012 | 1.385840054 | 1.717366157 | 1.121788073 |
| Incidence | High-income Asia Pacific | 2013 | 1.372132663 | 1.704479421 | 1.107219862 |
| Incidence | High-income Asia Pacific | 2014 | 1.358268771 | 1.692126585 | 1.091067309 |
| Incidence | High-income Asia Pacific | 2015 | 1.343045499 | 1.682270425 | 1.073392691 |
| Incidence | High-income Asia Pacific | 2016 | 1.30421307 | 1.658865517 | 1.034634451 |
| Incidence | High-income Asia Pacific | 2017 | 1.273186305 | 1.641724057 | 0.997695555 |
| Incidence | High-income Asia Pacific | 2018 | 1.271741956 | 1.644030341 | 1.000891756 |
| Incidence | High-income Asia Pacific | 2019 | 1.274728772 | 1.653244297 | 0.994177501 |
| Incidence | High-income North America | 1990 | 1.902107825 | 2.945777097 | 1.23939396 |
| Incidence | High-income North America | 1991 | 1.954196959 | 3.028131161 | 1.292184717 |
| Incidence | High-income North America | 1992 | 1.999041312 | 3.041851548 | 1.338190138 |
| Incidence | High-income North America | 1993 | 2.034886913 | 3.081386657 | 1.370443609 |
| Incidence | High-income North America | 1994 | 2.060185944 | 3.111538259 | 1.40130307 |
| Incidence | High-income North America | 1995 | 2.072859527 | 3.090093368 | 1.415213876 |
| Incidence | High-income North America | 1996 | 2.070865788 | 3.081511676 | 1.418293355 |
| Incidence | High-income North America | 1997 | 2.057305556 | 3.057362515 | 1.412823127 |
| Incidence | High-income North America | 1998 | 2.039215116 | 3.026165381 | 1.410791401 |
| Incidence | High-income North America | 1999 | 2.023663223 | 2.995832552 | 1.40485823 |
| Incidence | High-income North America | 2000 | 2.017457807 | 2.97896168 | 1.406233451 |
| Incidence | High-income North America | 2001 | 2.017159465 | 2.965718976 | 1.411517667 |
| Incidence | High-income North America | 2002 | 2.01554167 | 2.951396341 | 1.413065442 |
| Incidence | High-income North America | 2003 | 2.014181082 | 2.935421684 | 1.415353212 |
| Incidence | High-income North America | 2004 | 2.014687665 | 2.925785966 | 1.421054476 |
| Incidence | High-income North America | 2005 | 2.018670519 | 2.917750184 | 1.424629909 |
| Incidence | High-income North America | 2006 | 2.029493365 | 2.924230844 | 1.444324951 |
| Incidence | High-income North America | 2007 | 2.048094801 | 2.940840082 | 1.465832263 |
| Incidence | High-income North America | 2008 | 2.072220874 | 2.975504063 | 1.488851264 |
| Incidence | High-income North America | 2009 | 2.099601405 | 2.988285943 | 1.520149706 |
| Incidence | High-income North America | 2010 | 2.128166444 | 3.031832784 | 1.542962235 |
| Incidence | High-income North America | 2011 | 2.162504631 | 3.070149321 | 1.565945368 |
| Incidence | High-income North America | 2012 | 2.206230391 | 3.123520036 | 1.595504133 |
| Incidence | High-income North America | 2013 | 2.255693333 | 3.184914903 | 1.627563234 |
| Incidence | High-income North America | 2014 | 2.307039204 | 3.251033877 | 1.66065907 |
| Incidence | High-income North America | 2015 | 2.356640064 | 3.321641508 | 1.686637301 |
| Incidence | High-income North America | 2016 | 2.464540555 | 3.503600665 | 1.76475311 |
| Incidence | High-income North America | 2017 | 2.564065566 | 3.670121079 | 1.828169005 |
| Incidence | High-income North America | 2018 | 2.586860296 | 3.767130541 | 1.835017858 |
| Incidence | High-income North America | 2019 | 2.583156637 | 3.788065982 | 1.807991652 |
| Incidence | North Africa and Middle East | 1990 | 1.946629947 | 2.760356921 | 1.366258883 |
| Incidence | North Africa and Middle East | 1991 | 1.959840622 | 2.769184264 | 1.378337876 |
| Incidence | North Africa and Middle East | 1992 | 1.973179106 | 2.777795675 | 1.395146816 |
| Incidence | North Africa and Middle East | 1993 | 1.986482786 | 2.786197007 | 1.414269137 |
| Incidence | North Africa and Middle East | 1994 | 1.999321809 | 2.797525139 | 1.4291579 |
| Incidence | North Africa and Middle East | 1995 | 2.011835753 | 2.812975413 | 1.440373617 |
| Incidence | North Africa and Middle East | 1996 | 2.022548848 | 2.814825037 | 1.449365063 |
| Incidence | North Africa and Middle East | 1997 | 2.032661504 | 2.819509505 | 1.457286241 |
| Incidence | North Africa and Middle East | 1998 | 2.044103532 | 2.830409119 | 1.472232556 |
| Incidence | North Africa and Middle East | 1999 | 2.057962116 | 2.848378172 | 1.477424871 |
| Incidence | North Africa and Middle East | 2000 | 2.075697596 | 2.874902853 | 1.495217494 |
| Incidence | North Africa and Middle East | 2001 | 2.107506365 | 2.935444801 | 1.519169214 |
| Incidence | North Africa and Middle East | 2002 | 2.158558541 | 3.00370009 | 1.563075322 |
| Incidence | North Africa and Middle East | 2003 | 2.219169857 | 3.09905808 | 1.614346578 |
| Incidence | North Africa and Middle East | 2004 | 2.279099004 | 3.191858243 | 1.657890665 |
| Incidence | North Africa and Middle East | 2005 | 2.32979799 | 3.260633813 | 1.695111611 |
| Incidence | North Africa and Middle East | 2006 | 2.376148782 | 3.31808712 | 1.731940632 |
| Incidence | North Africa and Middle East | 2007 | 2.430098674 | 3.364448402 | 1.774575354 |
| Incidence | North Africa and Middle East | 2008 | 2.486961292 | 3.426446147 | 1.81548184 |
| Incidence | North Africa and Middle East | 2009 | 2.54018367 | 3.499329476 | 1.852802912 |
| Incidence | North Africa and Middle East | 2010 | 2.58460869 | 3.566142969 | 1.883903632 |
| Incidence | North Africa and Middle East | 2011 | 2.626079866 | 3.625451714 | 1.911356929 |
| Incidence | North Africa and Middle East | 2012 | 2.675893314 | 3.705051679 | 1.95523074 |
| Incidence | North Africa and Middle East | 2013 | 2.728215218 | 3.790066332 | 1.9969553 |
| Incidence | North Africa and Middle East | 2014 | 2.777005345 | 3.855605508 | 2.030404246 |
| Incidence | North Africa and Middle East | 2015 | 2.815755681 | 3.929296304 | 2.052611567 |
| Incidence | North Africa and Middle East | 2016 | 2.851727081 | 3.946363339 | 2.056155349 |
| Incidence | North Africa and Middle East | 2017 | 2.887014221 | 4.059164146 | 2.056667154 |
| Incidence | North Africa and Middle East | 2018 | 2.93527577 | 4.123605219 | 2.087260143 |
| Incidence | North Africa and Middle East | 2019 | 3.007385973 | 4.222013911 | 2.138677627 |
| Incidence | Oceania | 1990 | 0.812585274 | 1.129281317 | 0.583892013 |
| Incidence | Oceania | 1991 | 0.815753724 | 1.134939393 | 0.5854737 |
| Incidence | Oceania | 1992 | 0.819040488 | 1.138452828 | 0.589998352 |
| Incidence | Oceania | 1993 | 0.821992622 | 1.142548768 | 0.59420244 |
| Incidence | Oceania | 1994 | 0.824778748 | 1.144572773 | 0.598214312 |
| Incidence | Oceania | 1995 | 0.827788539 | 1.15201086 | 0.59802097 |
| Incidence | Oceania | 1996 | 0.833300525 | 1.160494891 | 0.604260854 |
| Incidence | Oceania | 1997 | 0.841683152 | 1.168118041 | 0.612486014 |
| Incidence | Oceania | 1998 | 0.850405212 | 1.171224517 | 0.62009142 |
| Incidence | Oceania | 1999 | 0.857049286 | 1.175175309 | 0.628345784 |
| Incidence | Oceania | 2000 | 0.859274489 | 1.177891968 | 0.626560459 |
| Incidence | Oceania | 2001 | 0.858312776 | 1.175851395 | 0.629727746 |
| Incidence | Oceania | 2002 | 0.855849996 | 1.172749993 | 0.625563089 |
| Incidence | Oceania | 2003 | 0.85265705 | 1.168266553 | 0.623122954 |
| Incidence | Oceania | 2004 | 0.849364875 | 1.163615676 | 0.619297456 |
| Incidence | Oceania | 2005 | 0.846385871 | 1.159484989 | 0.615920529 |
| Incidence | Oceania | 2006 | 0.844217304 | 1.157720246 | 0.614365628 |
| Incidence | Oceania | 2007 | 0.841470272 | 1.156121536 | 0.615006351 |
| Incidence | Oceania | 2008 | 0.838591864 | 1.153432517 | 0.612611043 |
| Incidence | Oceania | 2009 | 0.835667508 | 1.150608863 | 0.61348461 |
| Incidence | Oceania | 2010 | 0.832986413 | 1.146538054 | 0.610697716 |
| Incidence | Oceania | 2011 | 0.831351879 | 1.141087857 | 0.614594369 |
| Incidence | Oceania | 2012 | 0.82937095 | 1.143173017 | 0.613701918 |
| Incidence | Oceania | 2013 | 0.827590578 | 1.137378586 | 0.611310712 |
| Incidence | Oceania | 2014 | 0.825787211 | 1.136317946 | 0.609184697 |
| Incidence | Oceania | 2015 | 0.82382267 | 1.129769363 | 0.606428541 |
| Incidence | Oceania | 2016 | 0.824920084 | 1.140106401 | 0.607850461 |
| Incidence | Oceania | 2017 | 0.825321385 | 1.135067107 | 0.602903989 |
| Incidence | Oceania | 2018 | 0.823841326 | 1.135402681 | 0.603058193 |
| Incidence | Oceania | 2019 | 0.823084617 | 1.130955432 | 0.599928644 |
| Incidence | South Asia | 1990 | 0.758773669 | 1.115671197 | 0.495655891 |
| Incidence | South Asia | 1991 | 0.768355391 | 1.124003182 | 0.506256117 |
| Incidence | South Asia | 1992 | 0.775292719 | 1.130980299 | 0.516593744 |
| Incidence | South Asia | 1993 | 0.779949018 | 1.136497045 | 0.524288762 |
| Incidence | South Asia | 1994 | 0.782846381 | 1.138193325 | 0.530414483 |
| Incidence | South Asia | 1995 | 0.784408554 | 1.138843806 | 0.534877193 |
| Incidence | South Asia | 1996 | 0.779465791 | 1.126847197 | 0.534991845 |
| Incidence | South Asia | 1997 | 0.765609087 | 1.099369388 | 0.527830609 |
| Incidence | South Asia | 1998 | 0.747440903 | 1.064631749 | 0.523840694 |
| Incidence | South Asia | 1999 | 0.729723511 | 1.036900119 | 0.515755959 |
| Incidence | South Asia | 2000 | 0.71702162 | 1.018340087 | 0.510576589 |
| Incidence | South Asia | 2001 | 0.707753058 | 1.000672793 | 0.506095363 |
| Incidence | South Asia | 2002 | 0.698359665 | 0.980434734 | 0.500236842 |
| Incidence | South Asia | 2003 | 0.690164422 | 0.964588014 | 0.497516152 |
| Incidence | South Asia | 2004 | 0.684499873 | 0.958383534 | 0.495419313 |
| Incidence | South Asia | 2005 | 0.682671349 | 0.952804404 | 0.493994738 |
| Incidence | South Asia | 2006 | 0.685982596 | 0.955584254 | 0.498599416 |
| Incidence | South Asia | 2007 | 0.693593367 | 0.970670283 | 0.508420482 |
| Incidence | South Asia | 2008 | 0.703712732 | 0.9918657 | 0.513532812 |
| Incidence | South Asia | 2009 | 0.714555637 | 1.011348021 | 0.522995481 |
| Incidence | South Asia | 2010 | 0.724327169 | 1.02397851 | 0.529118711 |
| Incidence | South Asia | 2011 | 0.73736547 | 1.04429905 | 0.5374449 |
| Incidence | South Asia | 2012 | 0.756902677 | 1.077175517 | 0.547489516 |
| Incidence | South Asia | 2013 | 0.779806601 | 1.105089563 | 0.56239314 |
| Incidence | South Asia | 2014 | 0.802895998 | 1.130351166 | 0.576603938 |
| Incidence | South Asia | 2015 | 0.823148075 | 1.158873482 | 0.585007486 |
| Incidence | South Asia | 2016 | 0.851612338 | 1.223688752 | 0.592581216 |
| Incidence | South Asia | 2017 | 0.878218284 | 1.271554852 | 0.599062891 |
| Incidence | South Asia | 2018 | 0.893596295 | 1.302673283 | 0.6127284 |
| Incidence | South Asia | 2019 | 0.908187412 | 1.333913872 | 0.621024687 |
| Incidence | Southeast Asia | 1990 | 2.150207632 | 3.139853879 | 1.436263153 |
| Incidence | Southeast Asia | 1991 | 2.160393141 | 3.149654878 | 1.459716361 |
| Incidence | Southeast Asia | 1992 | 2.169222391 | 3.164100682 | 1.488287053 |
| Incidence | Southeast Asia | 1993 | 2.1765928 | 3.169576985 | 1.504290951 |
| Incidence | Southeast Asia | 1994 | 2.182653218 | 3.164992536 | 1.528265745 |
| Incidence | Southeast Asia | 1995 | 2.188008928 | 3.161650204 | 1.546543888 |
| Incidence | Southeast Asia | 1996 | 2.190676665 | 3.156753084 | 1.550607625 |
| Incidence | Southeast Asia | 1997 | 2.191223609 | 3.150548518 | 1.549141658 |
| Incidence | Southeast Asia | 1998 | 2.191138047 | 3.148154946 | 1.552674434 |
| Incidence | Southeast Asia | 1999 | 2.191863484 | 3.146507509 | 1.552585545 |
| Incidence | Southeast Asia | 2000 | 2.195663356 | 3.143477866 | 1.554155272 |
| Incidence | Southeast Asia | 2001 | 2.203587817 | 3.157558369 | 1.56953327 |
| Incidence | Southeast Asia | 2002 | 2.216151094 | 3.171907869 | 1.586899862 |
| Incidence | Southeast Asia | 2003 | 2.231227652 | 3.183372084 | 1.607417536 |
| Incidence | Southeast Asia | 2004 | 2.246975509 | 3.192687952 | 1.618179392 |
| Incidence | Southeast Asia | 2005 | 2.261838876 | 3.200734399 | 1.639422451 |
| Incidence | Southeast Asia | 2006 | 2.274025171 | 3.198115354 | 1.649894349 |
| Incidence | Southeast Asia | 2007 | 2.285421234 | 3.17519935 | 1.66762762 |
| Incidence | Southeast Asia | 2008 | 2.294100122 | 3.172045492 | 1.679947165 |
| Incidence | Southeast Asia | 2009 | 2.301839513 | 3.155411304 | 1.686169994 |
| Incidence | Southeast Asia | 2010 | 2.315964702 | 3.166872662 | 1.702710026 |
| Incidence | Southeast Asia | 2011 | 2.339349047 | 3.193798792 | 1.724212075 |
| Incidence | Southeast Asia | 2012 | 2.369995883 | 3.237029467 | 1.752590296 |
| Incidence | Southeast Asia | 2013 | 2.402075775 | 3.285924657 | 1.773320148 |
| Incidence | Southeast Asia | 2014 | 2.43032842 | 3.330655309 | 1.796052564 |
| Incidence | Southeast Asia | 2015 | 2.44987394 | 3.364645938 | 1.807814028 |
| Incidence | Southeast Asia | 2016 | 2.468369099 | 3.3752925 | 1.825496132 |
| Incidence | Southeast Asia | 2017 | 2.487908619 | 3.412923862 | 1.829832216 |
| Incidence | Southeast Asia | 2018 | 2.510605411 | 3.467382919 | 1.846532246 |
| Incidence | Southeast Asia | 2019 | 2.543268955 | 3.522425947 | 1.858138639 |
| Incidence | Southern Latin America | 1990 | 1.957106499 | 2.994984717 | 1.238570803 |
| Incidence | Southern Latin America | 1991 | 2.003828894 | 3.071165636 | 1.266167743 |
| Incidence | Southern Latin America | 1992 | 2.048146077 | 3.120307753 | 1.295723137 |
| Incidence | Southern Latin America | 1993 | 2.090114586 | 3.170349549 | 1.330675994 |
| Incidence | Southern Latin America | 1994 | 2.128961128 | 3.213543326 | 1.355972459 |
| Incidence | Southern Latin America | 1995 | 2.164533222 | 3.272037325 | 1.376539332 |
| Incidence | Southern Latin America | 1996 | 2.197173395 | 3.313045312 | 1.401033474 |
| Incidence | Southern Latin America | 1997 | 2.227176723 | 3.362703887 | 1.424214059 |
| Incidence | Southern Latin America | 1998 | 2.254202788 | 3.42337609 | 1.445145846 |
| Incidence | Southern Latin America | 1999 | 2.277683772 | 3.446917151 | 1.460483397 |
| Incidence | Southern Latin America | 2000 | 2.297878551 | 3.450802387 | 1.470234546 |
| Incidence | Southern Latin America | 2001 | 2.316360648 | 3.499237496 | 1.492037737 |
| Incidence | Southern Latin America | 2002 | 2.334361752 | 3.530262414 | 1.512470149 |
| Incidence | Southern Latin America | 2003 | 2.351465349 | 3.56975251 | 1.532077569 |
| Incidence | Southern Latin America | 2004 | 2.367954754 | 3.592712491 | 1.53622767 |
| Incidence | Southern Latin America | 2005 | 2.384568615 | 3.627747723 | 1.535955068 |
| Incidence | Southern Latin America | 2006 | 2.40183059 | 3.664309721 | 1.557440809 |
| Incidence | Southern Latin America | 2007 | 2.419533578 | 3.680453936 | 1.569088214 |
| Incidence | Southern Latin America | 2008 | 2.436879607 | 3.715596289 | 1.580676166 |
| Incidence | Southern Latin America | 2009 | 2.454652932 | 3.751704719 | 1.592059375 |
| Incidence | Southern Latin America | 2010 | 2.473607021 | 3.771695878 | 1.6084748 |
| Incidence | Southern Latin America | 2011 | 2.499880004 | 3.809271937 | 1.62653653 |
| Incidence | Southern Latin America | 2012 | 2.534624179 | 3.84037529 | 1.656987603 |
| Incidence | Southern Latin America | 2013 | 2.570183611 | 3.881800026 | 1.687593598 |
| Incidence | Southern Latin America | 2014 | 2.599496703 | 3.906868172 | 1.714141341 |
| Incidence | Southern Latin America | 2015 | 2.616972682 | 3.937549287 | 1.726375864 |
| Incidence | Southern Latin America | 2016 | 2.629630622 | 3.957777114 | 1.716247057 |
| Incidence | Southern Latin America | 2017 | 2.636996332 | 3.980547158 | 1.705902464 |
| Incidence | Southern Latin America | 2018 | 2.642825591 | 4.02023726 | 1.721464927 |
| Incidence | Southern Latin America | 2019 | 2.654758935 | 4.05836971 | 1.718987983 |
| Incidence | Southern Sub-Saharan Africa | 1990 | 1.483298985 | 2.163571152 | 0.998888116 |
| Incidence | Southern Sub-Saharan Africa | 1991 | 1.520246277 | 2.190543974 | 1.042492398 |
| Incidence | Southern Sub-Saharan Africa | 1992 | 1.557419927 | 2.21887544 | 1.088572071 |
| Incidence | Southern Sub-Saharan Africa | 1993 | 1.592718648 | 2.237340746 | 1.132736862 |
| Incidence | Southern Sub-Saharan Africa | 1994 | 1.624554115 | 2.257785909 | 1.180333045 |
| Incidence | Southern Sub-Saharan Africa | 1995 | 1.650975124 | 2.255180344 | 1.216003322 |
| Incidence | Southern Sub-Saharan Africa | 1996 | 1.680508971 | 2.250262325 | 1.269951237 |
| Incidence | Southern Sub-Saharan Africa | 1997 | 1.71753392 | 2.250347311 | 1.327680475 |
| Incidence | Southern Sub-Saharan Africa | 1998 | 1.753565593 | 2.279564879 | 1.376978281 |
| Incidence | Southern Sub-Saharan Africa | 1999 | 1.780900654 | 2.296136356 | 1.409481808 |
| Incidence | Southern Sub-Saharan Africa | 2000 | 1.79208528 | 2.301927265 | 1.419944761 |
| Incidence | Southern Sub-Saharan Africa | 2001 | 1.787319789 | 2.298633481 | 1.416214858 |
| Incidence | Southern Sub-Saharan Africa | 2002 | 1.774255769 | 2.284270628 | 1.401961431 |
| Incidence | Southern Sub-Saharan Africa | 2003 | 1.756418518 | 2.257267293 | 1.38197978 |
| Incidence | Southern Sub-Saharan Africa | 2004 | 1.73744504 | 2.245754142 | 1.362877616 |
| Incidence | Southern Sub-Saharan Africa | 2005 | 1.721127121 | 2.236983856 | 1.346904275 |
| Incidence | Southern Sub-Saharan Africa | 2006 | 1.703905366 | 2.219816438 | 1.329540799 |
| Incidence | Southern Sub-Saharan Africa | 2007 | 1.682078069 | 2.194654485 | 1.313068851 |
| Incidence | Southern Sub-Saharan Africa | 2008 | 1.659059041 | 2.165766913 | 1.294194239 |
| Incidence | Southern Sub-Saharan Africa | 2009 | 1.63886251 | 2.145083072 | 1.277155566 |
| Incidence | Southern Sub-Saharan Africa | 2010 | 1.625551104 | 2.138014307 | 1.263855814 |
| Incidence | Southern Sub-Saharan Africa | 2011 | 1.619750551 | 2.130486985 | 1.259264581 |
| Incidence | Southern Sub-Saharan Africa | 2012 | 1.618477063 | 2.142790393 | 1.259921213 |
| Incidence | Southern Sub-Saharan Africa | 2013 | 1.619804874 | 2.143769474 | 1.259141215 |
| Incidence | Southern Sub-Saharan Africa | 2014 | 1.622388124 | 2.139246524 | 1.257808864 |
| Incidence | Southern Sub-Saharan Africa | 2015 | 1.624874422 | 2.141036112 | 1.260224008 |
| Incidence | Southern Sub-Saharan Africa | 2016 | 1.631312834 | 2.179626881 | 1.238287973 |
| Incidence | Southern Sub-Saharan Africa | 2017 | 1.634402139 | 2.182299189 | 1.218529341 |
| Incidence | Southern Sub-Saharan Africa | 2018 | 1.625699232 | 2.177449867 | 1.21092052 |
| Incidence | Southern Sub-Saharan Africa | 2019 | 1.610615639 | 2.170971191 | 1.199372684 |
| Incidence | Tropical Latin America | 1990 | 1.768490444 | 2.720026443 | 1.080465203 |
| Incidence | Tropical Latin America | 1991 | 1.769602808 | 2.703055166 | 1.096226759 |
| Incidence | Tropical Latin America | 1992 | 1.768014053 | 2.695206913 | 1.112496066 |
| Incidence | Tropical Latin America | 1993 | 1.764474648 | 2.683836807 | 1.128158082 |
| Incidence | Tropical Latin America | 1994 | 1.759624284 | 2.674320879 | 1.13901311 |
| Incidence | Tropical Latin America | 1995 | 1.754196597 | 2.668632601 | 1.145061488 |
| Incidence | Tropical Latin America | 1996 | 1.74457826 | 2.634685972 | 1.139878357 |
| Incidence | Tropical Latin America | 1997 | 1.72857973 | 2.600444707 | 1.134043539 |
| Incidence | Tropical Latin America | 1998 | 1.709200849 | 2.562035534 | 1.12878771 |
| Incidence | Tropical Latin America | 1999 | 1.68938887 | 2.523756248 | 1.120384365 |
| Incidence | Tropical Latin America | 2000 | 1.672175342 | 2.497337099 | 1.110857306 |
| Incidence | Tropical Latin America | 2001 | 1.653080291 | 2.46035575 | 1.102598457 |
| Incidence | Tropical Latin America | 2002 | 1.628792854 | 2.41701891 | 1.091436011 |
| Incidence | Tropical Latin America | 2003 | 1.604069507 | 2.37859364 | 1.077817535 |
| Incidence | Tropical Latin America | 2004 | 1.583543125 | 2.347931318 | 1.065897089 |
| Incidence | Tropical Latin America | 2005 | 1.571835621 | 2.332112945 | 1.060608974 |
| Incidence | Tropical Latin America | 2006 | 1.571401663 | 2.33897017 | 1.061889264 |
| Incidence | Tropical Latin America | 2007 | 1.579021281 | 2.327523981 | 1.06979421 |
| Incidence | Tropical Latin America | 2008 | 1.590369131 | 2.32988046 | 1.073101103 |
| Incidence | Tropical Latin America | 2009 | 1.601165653 | 2.337545139 | 1.075725931 |
| Incidence | Tropical Latin America | 2010 | 1.607164334 | 2.342605126 | 1.07724302 |
| Incidence | Tropical Latin America | 2011 | 1.607929481 | 2.344629463 | 1.078432196 |
| Incidence | Tropical Latin America | 2012 | 1.60735697 | 2.344279865 | 1.081291111 |
| Incidence | Tropical Latin America | 2013 | 1.607424489 | 2.353577982 | 1.084854195 |
| Incidence | Tropical Latin America | 2014 | 1.610112898 | 2.361963039 | 1.087418735 |
| Incidence | Tropical Latin America | 2015 | 1.617342887 | 2.37401779 | 1.090051233 |
| Incidence | Tropical Latin America | 2016 | 1.679218904 | 2.486633663 | 1.118903354 |
| Incidence | Tropical Latin America | 2017 | 1.740972547 | 2.588870848 | 1.136075577 |
| Incidence | Tropical Latin America | 2018 | 1.742992432 | 2.595009418 | 1.129040622 |
| Incidence | Tropical Latin America | 2019 | 1.724161397 | 2.562503125 | 1.097084207 |
| Incidence | Western Europe | 1990 | 2.707137305 | 4.049888929 | 1.818893274 |
| Incidence | Western Europe | 1991 | 2.739022894 | 4.080034529 | 1.852155064 |
| Incidence | Western Europe | 1992 | 2.768983971 | 4.12155163 | 1.872564613 |
| Incidence | Western Europe | 1993 | 2.795633664 | 4.152798003 | 1.888427231 |
| Incidence | Western Europe | 1994 | 2.818190373 | 4.186925653 | 1.903846337 |
| Incidence | Western Europe | 1995 | 2.835668729 | 4.203849807 | 1.913660208 |
| Incidence | Western Europe | 1996 | 2.850387331 | 4.242686311 | 1.922731564 |
| Incidence | Western Europe | 1997 | 2.865943899 | 4.276965352 | 1.933630314 |
| Incidence | Western Europe | 1998 | 2.879154109 | 4.302054775 | 1.943266146 |
| Incidence | Western Europe | 1999 | 2.886157082 | 4.299826673 | 1.956392166 |
| Incidence | Western Europe | 2000 | 2.885127008 | 4.280247832 | 1.958196464 |
| Incidence | Western Europe | 2001 | 2.874445237 | 4.26284223 | 1.958547226 |
| Incidence | Western Europe | 2002 | 2.858038621 | 4.227922688 | 1.943889735 |
| Incidence | Western Europe | 2003 | 2.838942933 | 4.182117884 | 1.934195879 |
| Incidence | Western Europe | 2004 | 2.818299895 | 4.15499893 | 1.925293817 |
| Incidence | Western Europe | 2005 | 2.798666306 | 4.119972993 | 1.912145781 |
| Incidence | Western Europe | 2006 | 2.776051744 | 4.083101167 | 1.901555687 |
| Incidence | Western Europe | 2007 | 2.74796944 | 4.031961397 | 1.887130878 |
| Incidence | Western Europe | 2008 | 2.718410225 | 3.976327658 | 1.868358116 |
| Incidence | Western Europe | 2009 | 2.690144233 | 3.924324913 | 1.84786518 |
| Incidence | Western Europe | 2010 | 2.666675261 | 3.88548491 | 1.828175156 |
| Incidence | Western Europe | 2011 | 2.646007851 | 3.852722132 | 1.813474955 |
| Incidence | Western Europe | 2012 | 2.625928025 | 3.842212937 | 1.801375383 |
| Incidence | Western Europe | 2013 | 2.607282826 | 3.818459457 | 1.790155711 |
| Incidence | Western Europe | 2014 | 2.590481804 | 3.790589372 | 1.779258288 |
| Incidence | Western Europe | 2015 | 2.576021014 | 3.770179597 | 1.776364194 |
| Incidence | Western Europe | 2016 | 2.551579768 | 3.733998882 | 1.760798397 |
| Incidence | Western Europe | 2017 | 2.528125787 | 3.709065168 | 1.742035867 |
| Incidence | Western Europe | 2018 | 2.500825223 | 3.68300071 | 1.719704184 |
| Incidence | Western Europe | 2019 | 2.455483008 | 3.62259848 | 1.699797981 |
| Incidence | Western Sub-Saharan Africa | 1990 | 1.523513484 | 2.242435447 | 0.997960396 |
| Incidence | Western Sub-Saharan Africa | 1991 | 1.530030141 | 2.24783165 | 1.014778636 |
| Incidence | Western Sub-Saharan Africa | 1992 | 1.535676294 | 2.261820608 | 1.029275665 |
| Incidence | Western Sub-Saharan Africa | 1993 | 1.539925684 | 2.264976237 | 1.045520761 |
| Incidence | Western Sub-Saharan Africa | 1994 | 1.543089489 | 2.264056467 | 1.05859419 |
| Incidence | Western Sub-Saharan Africa | 1995 | 1.545560581 | 2.249207297 | 1.06570448 |
| Incidence | Western Sub-Saharan Africa | 1996 | 1.545822297 | 2.246540462 | 1.067534465 |
| Incidence | Western Sub-Saharan Africa | 1997 | 1.544091771 | 2.240831137 | 1.069991531 |
| Incidence | Western Sub-Saharan Africa | 1998 | 1.541355434 | 2.23326913 | 1.066062981 |
| Incidence | Western Sub-Saharan Africa | 1999 | 1.539492169 | 2.225801533 | 1.06793681 |
| Incidence | Western Sub-Saharan Africa | 2000 | 1.539451954 | 2.222611257 | 1.070579947 |
| Incidence | Western Sub-Saharan Africa | 2001 | 1.544246245 | 2.227846704 | 1.076373591 |
| Incidence | Western Sub-Saharan Africa | 2002 | 1.554043501 | 2.235528796 | 1.08331902 |
| Incidence | Western Sub-Saharan Africa | 2003 | 1.56584881 | 2.251191235 | 1.091595191 |
| Incidence | Western Sub-Saharan Africa | 2004 | 1.577275241 | 2.26948125 | 1.098816774 |
| Incidence | Western Sub-Saharan Africa | 2005 | 1.585053358 | 2.278836435 | 1.100844066 |
| Incidence | Western Sub-Saharan Africa | 2006 | 1.590257274 | 2.28805376 | 1.107588005 |
| Incidence | Western Sub-Saharan Africa | 2007 | 1.595595005 | 2.297016784 | 1.111961435 |
| Incidence | Western Sub-Saharan Africa | 2008 | 1.600980837 | 2.307180112 | 1.116834184 |
| Incidence | Western Sub-Saharan Africa | 2009 | 1.606751391 | 2.316462772 | 1.120128761 |
| Incidence | Western Sub-Saharan Africa | 2010 | 1.614418611 | 2.330143764 | 1.127751488 |
| Incidence | Western Sub-Saharan Africa | 2011 | 1.625017722 | 2.34223716 | 1.13336026 |
| Incidence | Western Sub-Saharan Africa | 2012 | 1.639193459 | 2.363106058 | 1.146531217 |
| Incidence | Western Sub-Saharan Africa | 2013 | 1.655758766 | 2.393005604 | 1.161484086 |
| Incidence | Western Sub-Saharan Africa | 2014 | 1.673323448 | 2.419660102 | 1.168081582 |
| Incidence | Western Sub-Saharan Africa | 2015 | 1.690794555 | 2.449333029 | 1.182097917 |
| Incidence | Western Sub-Saharan Africa | 2016 | 1.732647339 | 2.520445399 | 1.192244916 |
| Incidence | Western Sub-Saharan Africa | 2017 | 1.776596403 | 2.580297908 | 1.190395644 |
| Incidence | Western Sub-Saharan Africa | 2018 | 1.802386531 | 2.623734747 | 1.207638171 |
| Incidence | Western Sub-Saharan Africa | 2019 | 1.829642457 | 2.656496245 | 1.220380978 |
| DALYs | Andean Latin America | 1990 | 153.9986922 | 209.7764003 | 110.2532519 |
| DALYs | Andean Latin America | 1991 | 151.6850094 | 206.7795689 | 109.1578361 |
| DALYs | Andean Latin America | 1992 | 156.7107117 | 213.0656536 | 110.9502926 |
| DALYs | Andean Latin America | 1993 | 157.2688293 | 213.1270136 | 111.1069865 |
| DALYs | Andean Latin America | 1994 | 157.4350494 | 212.8898819 | 113.8013016 |
| DALYs | Andean Latin America | 1995 | 157.721452 | 210.9499548 | 114.2297657 |
| DALYs | Andean Latin America | 1996 | 154.3561311 | 204.6243866 | 111.533142 |
| DALYs | Andean Latin America | 1997 | 152.4803511 | 205.5281859 | 110.521342 |
| DALYs | Andean Latin America | 1998 | 151.3485404 | 203.9831267 | 109.3838205 |
| DALYs | Andean Latin America | 1999 | 145.5775774 | 196.0641756 | 104.8152571 |
| DALYs | Andean Latin America | 2000 | 143.0056916 | 191.1361927 | 103.3581143 |
| DALYs | Andean Latin America | 2001 | 137.3656737 | 185.2750503 | 99.5217838 |
| DALYs | Andean Latin America | 2002 | 138.2266896 | 186.9605355 | 100.1250385 |
| DALYs | Andean Latin America | 2003 | 139.054395 | 187.7648821 | 101.8413833 |
| DALYs | Andean Latin America | 2004 | 136.7117409 | 185.1627259 | 99.40610034 |
| DALYs | Andean Latin America | 2005 | 136.0149106 | 183.5148177 | 98.51371839 |
| DALYs | Andean Latin America | 2006 | 134.4639657 | 178.9972572 | 97.13439667 |
| DALYs | Andean Latin America | 2007 | 132.6102506 | 178.1187101 | 96.44317877 |
| DALYs | Andean Latin America | 2008 | 133.2634857 | 177.8244886 | 97.04968528 |
| DALYs | Andean Latin America | 2009 | 139.0188782 | 185.6602785 | 100.7494626 |
| DALYs | Andean Latin America | 2010 | 139.6059079 | 185.520407 | 100.3752516 |
| DALYs | Andean Latin America | 2011 | 137.5393045 | 184.4613931 | 98.95957513 |
| DALYs | Andean Latin America | 2012 | 136.326961 | 182.0359674 | 98.28877878 |
| DALYs | Andean Latin America | 2013 | 134.1853408 | 179.4870638 | 97.22847474 |
| DALYs | Andean Latin America | 2014 | 131.2194831 | 176.8067891 | 94.45481409 |
| DALYs | Andean Latin America | 2015 | 129.51135 | 175.9913985 | 93.17446051 |
| DALYs | Andean Latin America | 2016 | 129.6221643 | 176.4364682 | 91.74022363 |
| DALYs | Andean Latin America | 2017 | 129.4305157 | 174.5283838 | 91.24016907 |
| DALYs | Andean Latin America | 2018 | 129.0889582 | 176.7243454 | 90.00015568 |
| DALYs | Andean Latin America | 2019 | 128.6743142 | 178.8466676 | 88.55516068 |
| DALYs | Australasia | 1990 | 29.90193135 | 40.29379694 | 21.92332204 |
| DALYs | Australasia | 1991 | 29.19973024 | 39.01998208 | 21.4924145 |
| DALYs | Australasia | 1992 | 28.71964844 | 37.9854143 | 21.16798421 |
| DALYs | Australasia | 1993 | 28.27400798 | 37.08196576 | 20.91821744 |
| DALYs | Australasia | 1994 | 28.23792603 | 36.92022298 | 21.02085014 |
| DALYs | Australasia | 1995 | 27.96729771 | 36.48510034 | 20.89988519 |
| DALYs | Australasia | 1996 | 27.90500561 | 36.51968224 | 21.0483278 |
| DALYs | Australasia | 1997 | 27.6114196 | 35.93606808 | 20.92077642 |
| DALYs | Australasia | 1998 | 26.96663247 | 35.17730231 | 20.56133605 |
| DALYs | Australasia | 1999 | 26.59962686 | 34.64756137 | 20.27443589 |
| DALYs | Australasia | 2000 | 26.28051205 | 34.17515541 | 20.11063139 |
| DALYs | Australasia | 2001 | 26.24887376 | 33.91417093 | 20.20586172 |
| DALYs | Australasia | 2002 | 26.82621328 | 34.44475902 | 20.57250939 |
| DALYs | Australasia | 2003 | 26.97614617 | 34.53107165 | 20.64727186 |
| DALYs | Australasia | 2004 | 26.90154043 | 34.45060785 | 20.56535768 |
| DALYs | Australasia | 2005 | 26.92010638 | 34.44772036 | 20.48269517 |
| DALYs | Australasia | 2006 | 27.28961605 | 35.05829018 | 20.78908866 |
| DALYs | Australasia | 2007 | 28.49158433 | 36.57527816 | 21.81321265 |
| DALYs | Australasia | 2008 | 29.77266396 | 38.27394415 | 22.82189718 |
| DALYs | Australasia | 2009 | 30.79988763 | 39.75303164 | 23.55965462 |
| DALYs | Australasia | 2010 | 31.38132159 | 40.46254313 | 24.14911449 |
| DALYs | Australasia | 2011 | 31.76292442 | 40.85595435 | 24.52009343 |
| DALYs | Australasia | 2012 | 31.76219159 | 40.76694684 | 24.52202206 |
| DALYs | Australasia | 2013 | 32.44675945 | 41.67042187 | 25.1003743 |
| DALYs | Australasia | 2014 | 33.14870129 | 42.48351752 | 25.73596872 |
| DALYs | Australasia | 2015 | 33.47575492 | 43.14045174 | 25.9363735 |
| DALYs | Australasia | 2016 | 32.85559269 | 42.14311438 | 25.24347657 |
| DALYs | Australasia | 2017 | 32.39508297 | 41.4598014 | 24.84869615 |
| DALYs | Australasia | 2018 | 32.32500996 | 40.81650311 | 24.98322444 |
| DALYs | Australasia | 2019 | 32.07612499 | 40.71582603 | 24.94354633 |
| DALYs | Caribbean | 1990 | 104.1471652 | 138.3733152 | 76.66665973 |
| DALYs | Caribbean | 1991 | 102.6907176 | 136.702369 | 75.10470764 |
| DALYs | Caribbean | 1992 | 102.4196748 | 135.9529175 | 74.54585907 |
| DALYs | Caribbean | 1993 | 103.2643196 | 137.4412318 | 75.45447579 |
| DALYs | Caribbean | 1994 | 101.1816877 | 134.6786785 | 74.05453783 |
| DALYs | Caribbean | 1995 | 99.74570003 | 133.1582268 | 73.39958803 |
| DALYs | Caribbean | 1996 | 97.37826586 | 130.7076207 | 71.40400798 |
| DALYs | Caribbean | 1997 | 94.11343272 | 125.9763398 | 68.64991868 |
| DALYs | Caribbean | 1998 | 92.9295927 | 124.7467064 | 67.79386413 |
| DALYs | Caribbean | 1999 | 89.59172886 | 120.370834 | 65.58893266 |
| DALYs | Caribbean | 2000 | 85.94277385 | 115.9074364 | 63.16781709 |
| DALYs | Caribbean | 2001 | 85.13358702 | 115.058308 | 62.14451621 |
| DALYs | Caribbean | 2002 | 82.40055754 | 110.7232287 | 60.49841858 |
| DALYs | Caribbean | 2003 | 82.37000904 | 111.4556406 | 59.81390549 |
| DALYs | Caribbean | 2004 | 82.3054186 | 112.7142908 | 59.61727535 |
| DALYs | Caribbean | 2005 | 82.00916153 | 111.9571258 | 59.38554793 |
| DALYs | Caribbean | 2006 | 79.59762129 | 109.6561276 | 57.85923429 |
| DALYs | Caribbean | 2007 | 78.63584223 | 108.6988995 | 56.88913495 |
| DALYs | Caribbean | 2008 | 77.89687597 | 108.1592592 | 56.40151576 |
| DALYs | Caribbean | 2009 | 77.3550461 | 107.0881004 | 55.36715615 |
| DALYs | Caribbean | 2010 | 76.36566921 | 104.9373209 | 54.81340901 |
| DALYs | Caribbean | 2011 | 76.63905889 | 105.3583765 | 55.09014309 |
| DALYs | Caribbean | 2012 | 78.13353154 | 107.3582305 | 56.46492061 |
| DALYs | Caribbean | 2013 | 80.57473991 | 110.5606059 | 57.57156084 |
| DALYs | Caribbean | 2014 | 82.79920254 | 114.5238729 | 59.27560739 |
| DALYs | Caribbean | 2015 | 84.6984985 | 117.0136713 | 59.90934737 |
| DALYs | Caribbean | 2016 | 86.10032439 | 119.114953 | 61.41266283 |
| DALYs | Caribbean | 2017 | 86.95490722 | 121.7662386 | 61.06520784 |
| DALYs | Caribbean | 2018 | 87.42267599 | 122.3175999 | 60.23065187 |
| DALYs | Caribbean | 2019 | 87.48358403 | 123.7122152 | 59.85501633 |
| DALYs | Central Asia | 1990 | 55.31557084 | 74.55922907 | 39.54482358 |
| DALYs | Central Asia | 1991 | 58.93085997 | 79.19370622 | 42.59268511 |
| DALYs | Central Asia | 1992 | 63.41721846 | 84.32668002 | 46.071932 |
| DALYs | Central Asia | 1993 | 69.55354771 | 92.54983107 | 50.80101731 |
| DALYs | Central Asia | 1994 | 75.5557693 | 100.7923536 | 55.08130436 |
| DALYs | Central Asia | 1995 | 79.57070371 | 106.5827043 | 58.06256835 |
| DALYs | Central Asia | 1996 | 82.79251346 | 110.8944609 | 60.3966281 |
| DALYs | Central Asia | 1997 | 83.96447419 | 111.3628251 | 61.65861983 |
| DALYs | Central Asia | 1998 | 84.72110777 | 111.9616907 | 62.07981258 |
| DALYs | Central Asia | 1999 | 85.63136963 | 113.0221164 | 62.69910827 |
| DALYs | Central Asia | 2000 | 88.55386227 | 117.0442775 | 65.25060513 |
| DALYs | Central Asia | 2001 | 91.19098174 | 119.8279312 | 66.93521297 |
| DALYs | Central Asia | 2002 | 94.51990115 | 124.7431149 | 69.50666767 |
| DALYs | Central Asia | 2003 | 98.21168484 | 130.3011265 | 72.14486571 |
| DALYs | Central Asia | 2004 | 102.802641 | 135.936056 | 75.59341392 |
| DALYs | Central Asia | 2005 | 107.2249056 | 142.3957906 | 78.85006672 |
| DALYs | Central Asia | 2006 | 107.7651952 | 143.407582 | 79.57745144 |
| DALYs | Central Asia | 2007 | 109.0772037 | 143.8867441 | 80.04964179 |
| DALYs | Central Asia | 2008 | 109.505941 | 144.30178 | 81.0136517 |
| DALYs | Central Asia | 2009 | 109.3428326 | 144.3842899 | 80.88208109 |
| DALYs | Central Asia | 2010 | 111.1109842 | 147.0679189 | 82.67340516 |
| DALYs | Central Asia | 2011 | 113.8371952 | 150.3334157 | 84.4542664 |
| DALYs | Central Asia | 2012 | 116.2621951 | 153.6650242 | 86.24048163 |
| DALYs | Central Asia | 2013 | 115.7932385 | 152.8833085 | 85.84257962 |
| DALYs | Central Asia | 2014 | 116.2638103 | 153.6003317 | 86.49544588 |
| DALYs | Central Asia | 2015 | 116.2380053 | 152.9354472 | 86.103449 |
| DALYs | Central Asia | 2016 | 115.4520866 | 153.1522012 | 85.39103992 |
| DALYs | Central Asia | 2017 | 114.5075334 | 153.4258625 | 84.72585142 |
| DALYs | Central Asia | 2018 | 113.2525926 | 150.7004139 | 82.29685424 |
| DALYs | Central Asia | 2019 | 112.3914948 | 151.1673999 | 81.58327247 |
| DALYs | Central Europe | 1990 | 40.27859127 | 52.95561576 | 30.09189781 |
| DALYs | Central Europe | 1991 | 40.9768327 | 54.14178171 | 30.41851109 |
| DALYs | Central Europe | 1992 | 42.23100086 | 56.04817008 | 31.15183704 |
| DALYs | Central Europe | 1993 | 42.70904559 | 57.09791239 | 31.35741362 |
| DALYs | Central Europe | 1994 | 43.11459675 | 57.84220064 | 31.51940902 |
| DALYs | Central Europe | 1995 | 43.66536938 | 58.81048556 | 31.76734983 |
| DALYs | Central Europe | 1996 | 42.75774944 | 57.46888695 | 30.90742703 |
| DALYs | Central Europe | 1997 | 42.55610695 | 57.33843449 | 30.68502878 |
| DALYs | Central Europe | 1998 | 41.25379718 | 55.68591775 | 29.55171823 |
| DALYs | Central Europe | 1999 | 40.01750661 | 54.02218156 | 28.64111235 |
| DALYs | Central Europe | 2000 | 38.61220964 | 52.18098361 | 27.45461655 |
| DALYs | Central Europe | 2001 | 38.67403285 | 52.20385902 | 27.60399071 |
| DALYs | Central Europe | 2002 | 39.07584776 | 52.64082615 | 27.89980041 |
| DALYs | Central Europe | 2003 | 39.79865516 | 53.73337616 | 28.47706756 |
| DALYs | Central Europe | 2004 | 39.9898431 | 53.93306583 | 28.79159624 |
| DALYs | Central Europe | 2005 | 40.55052581 | 54.8660409 | 29.25132489 |
| DALYs | Central Europe | 2006 | 40.5765051 | 54.82423331 | 29.336634 |
| DALYs | Central Europe | 2007 | 41.41514367 | 55.81489598 | 29.92725283 |
| DALYs | Central Europe | 2008 | 41.99932827 | 56.69406131 | 30.50850606 |
| DALYs | Central Europe | 2009 | 42.068542 | 56.77048813 | 30.57143888 |
| DALYs | Central Europe | 2010 | 40.91517737 | 55.26872226 | 29.68022465 |
| DALYs | Central Europe | 2011 | 39.1546216 | 52.8577089 | 28.1485439 |
| DALYs | Central Europe | 2012 | 38.52219414 | 52.00396709 | 27.87474415 |
| DALYs | Central Europe | 2013 | 37.11115804 | 50.09360286 | 26.7580973 |
| DALYs | Central Europe | 2014 | 36.89290029 | 49.83057893 | 26.6209268 |
| DALYs | Central Europe | 2015 | 36.90707377 | 49.95163377 | 26.72324009 |
| DALYs | Central Europe | 2016 | 36.48163702 | 49.38956169 | 26.39006139 |
| DALYs | Central Europe | 2017 | 36.38699797 | 49.86566543 | 26.20832033 |
| DALYs | Central Europe | 2018 | 36.30193712 | 50.26879263 | 25.83461147 |
| DALYs | Central Europe | 2019 | 36.13192101 | 49.93033618 | 25.46252841 |
| DALYs | Central Latin America | 1990 | 188.7611508 | 244.2620161 | 141.5892434 |
| DALYs | Central Latin America | 1991 | 188.6914346 | 244.4800743 | 141.848694 |
| DALYs | Central Latin America | 1992 | 189.537839 | 244.9880227 | 142.8784515 |
| DALYs | Central Latin America | 1993 | 190.8158693 | 247.2325363 | 143.643231 |
| DALYs | Central Latin America | 1994 | 189.9794923 | 245.0408827 | 142.5531767 |
| DALYs | Central Latin America | 1995 | 187.3353047 | 241.5054549 | 140.4893831 |
| DALYs | Central Latin America | 1996 | 183.1705653 | 236.8094354 | 136.8065443 |
| DALYs | Central Latin America | 1997 | 181.7233729 | 234.4627518 | 136.4139124 |
| DALYs | Central Latin America | 1998 | 181.4021539 | 233.4536594 | 135.6167495 |
| DALYs | Central Latin America | 1999 | 176.2595485 | 227.6464982 | 132.286498 |
| DALYs | Central Latin America | 2000 | 171.9944505 | 222.3358359 | 128.7739236 |
| DALYs | Central Latin America | 2001 | 168.3796419 | 217.6391735 | 125.6936649 |
| DALYs | Central Latin America | 2002 | 168.3998989 | 217.6892127 | 125.6539988 |
| DALYs | Central Latin America | 2003 | 167.2243938 | 216.0427825 | 124.7632703 |
| DALYs | Central Latin America | 2004 | 162.1858625 | 209.7355538 | 121.1114104 |
| DALYs | Central Latin America | 2005 | 161.4750504 | 208.7633337 | 120.897755 |
| DALYs | Central Latin America | 2006 | 158.8422232 | 205.5278668 | 118.655064 |
| DALYs | Central Latin America | 2007 | 158.4849302 | 204.2646741 | 118.8888445 |
| DALYs | Central Latin America | 2008 | 163.7575913 | 210.23756 | 122.5034463 |
| DALYs | Central Latin America | 2009 | 166.4852078 | 213.251673 | 124.9549867 |
| DALYs | Central Latin America | 2010 | 161.4031379 | 205.997803 | 121.5112833 |
| DALYs | Central Latin America | 2011 | 157.2408159 | 201.4097187 | 118.8545534 |
| DALYs | Central Latin America | 2012 | 155.3153287 | 199.3937539 | 117.3487624 |
| DALYs | Central Latin America | 2013 | 156.5865795 | 201.332562 | 118.019166 |
| DALYs | Central Latin America | 2014 | 155.1656472 | 199.3796755 | 116.6220325 |
| DALYs | Central Latin America | 2015 | 154.3151588 | 197.4280579 | 115.7700385 |
| DALYs | Central Latin America | 2016 | 156.2007547 | 200.9670858 | 117.4866463 |
| DALYs | Central Latin America | 2017 | 159.6702893 | 204.4717235 | 120.7602697 |
| DALYs | Central Latin America | 2018 | 160.4854514 | 209.5433493 | 120.0333022 |
| DALYs | Central Latin America | 2019 | 161.3913983 | 212.3770358 | 118.9694523 |
| DALYs | Central Sub-Saharan Africa | 1990 | 85.74021432 | 122.0218302 | 58.63319988 |
| DALYs | Central Sub-Saharan Africa | 1991 | 85.86947485 | 122.7298481 | 58.71800899 |
| DALYs | Central Sub-Saharan Africa | 1992 | 85.42028807 | 121.7690396 | 58.75533493 |
| DALYs | Central Sub-Saharan Africa | 1993 | 84.81318513 | 119.8451321 | 58.07542195 |
| DALYs | Central Sub-Saharan Africa | 1994 | 84.636585 | 119.8060379 | 58.09727365 |
| DALYs | Central Sub-Saharan Africa | 1995 | 84.79854596 | 120.7561208 | 58.2781544 |
| DALYs | Central Sub-Saharan Africa | 1996 | 84.93192659 | 120.1860812 | 56.95731856 |
| DALYs | Central Sub-Saharan Africa | 1997 | 83.34749017 | 117.9175748 | 55.44842055 |
| DALYs | Central Sub-Saharan Africa | 1998 | 83.50367512 | 118.5460586 | 56.13320142 |
| DALYs | Central Sub-Saharan Africa | 1999 | 83.02584715 | 119.1051708 | 54.79934018 |
| DALYs | Central Sub-Saharan Africa | 2000 | 81.91020774 | 115.9912319 | 54.0032696 |
| DALYs | Central Sub-Saharan Africa | 2001 | 80.26508339 | 114.571985 | 53.69618462 |
| DALYs | Central Sub-Saharan Africa | 2002 | 78.47872477 | 111.611094 | 52.45406578 |
| DALYs | Central Sub-Saharan Africa | 2003 | 78.27522635 | 112.2913708 | 52.42096236 |
| DALYs | Central Sub-Saharan Africa | 2004 | 77.23099243 | 111.9732032 | 52.21095771 |
| DALYs | Central Sub-Saharan Africa | 2005 | 75.97058746 | 111.3257518 | 50.69359874 |
| DALYs | Central Sub-Saharan Africa | 2006 | 75.2375137 | 110.0071838 | 49.99120898 |
| DALYs | Central Sub-Saharan Africa | 2007 | 74.31538233 | 107.8276392 | 49.35913233 |
| DALYs | Central Sub-Saharan Africa | 2008 | 74.24477378 | 107.9978426 | 49.20828606 |
| DALYs | Central Sub-Saharan Africa | 2009 | 74.20130315 | 108.1511106 | 48.79751978 |
| DALYs | Central Sub-Saharan Africa | 2010 | 74.00098542 | 107.6644178 | 48.46577699 |
| DALYs | Central Sub-Saharan Africa | 2011 | 73.56246511 | 106.4910158 | 48.07054797 |
| DALYs | Central Sub-Saharan Africa | 2012 | 72.71986412 | 105.0244603 | 47.46882298 |
| DALYs | Central Sub-Saharan Africa | 2013 | 71.47784025 | 103.7301213 | 47.29784588 |
| DALYs | Central Sub-Saharan Africa | 2014 | 70.02370374 | 103.1048811 | 45.88144997 |
| DALYs | Central Sub-Saharan Africa | 2015 | 68.70730932 | 101.2752667 | 44.73241486 |
| DALYs | Central Sub-Saharan Africa | 2016 | 70.38283478 | 104.1010926 | 45.93469413 |
| DALYs | Central Sub-Saharan Africa | 2017 | 73.92523728 | 109.3987907 | 47.50490348 |
| DALYs | Central Sub-Saharan Africa | 2018 | 74.21168922 | 110.9078535 | 47.29087116 |
| DALYs | Central Sub-Saharan Africa | 2019 | 74.23809659 | 110.7465465 | 47.50893404 |
| DALYs | East Asia | 1990 | 65.25362524 | 81.38445786 | 51.77343681 |
| DALYs | East Asia | 1991 | 65.20035686 | 80.90126293 | 51.67689343 |
| DALYs | East Asia | 1992 | 64.64723992 | 80.15328168 | 51.3819589 |
| DALYs | East Asia | 1993 | 64.05974772 | 78.64381284 | 51.62915577 |
| DALYs | East Asia | 1994 | 63.29503402 | 76.42087646 | 51.21681584 |
| DALYs | East Asia | 1995 | 62.3355961 | 75.299409 | 51.3347027 |
| DALYs | East Asia | 1996 | 61.11798645 | 73.99423445 | 50.05186016 |
| DALYs | East Asia | 1997 | 59.35753054 | 71.66827763 | 48.84442006 |
| DALYs | East Asia | 1998 | 57.86861096 | 69.49990764 | 47.60049711 |
| DALYs | East Asia | 1999 | 56.40377821 | 67.63610953 | 46.31250497 |
| DALYs | East Asia | 2000 | 54.05476293 | 65.16094146 | 44.55727591 |
| DALYs | East Asia | 2001 | 50.21165206 | 60.41161943 | 40.84190172 |
| DALYs | East Asia | 2002 | 45.92115593 | 56.24887856 | 37.24379954 |
| DALYs | East Asia | 2003 | 42.30309793 | 51.64723938 | 33.95688458 |
| DALYs | East Asia | 2004 | 39.66391965 | 49.52812481 | 31.56628388 |
| DALYs | East Asia | 2005 | 37.59309446 | 46.85472462 | 29.70684969 |
| DALYs | East Asia | 2006 | 35.65293621 | 44.65738059 | 28.24895157 |
| DALYs | East Asia | 2007 | 33.97250617 | 42.37040199 | 26.89500371 |
| DALYs | East Asia | 2008 | 32.9220208 | 40.93581016 | 26.23276482 |
| DALYs | East Asia | 2009 | 31.57817026 | 38.91016408 | 25.1190791 |
| DALYs | East Asia | 2010 | 30.37067673 | 37.49541858 | 24.21577243 |
| DALYs | East Asia | 2011 | 29.51289182 | 37.10048343 | 23.3584198 |
| DALYs | East Asia | 2012 | 29.27804325 | 36.09990189 | 23.31570438 |
| DALYs | East Asia | 2013 | 29.57011002 | 36.64156085 | 23.33878863 |
| DALYs | East Asia | 2014 | 30.19513867 | 37.59702395 | 24.26452912 |
| DALYs | East Asia | 2015 | 30.33805515 | 37.47579112 | 24.11149591 |
| DALYs | East Asia | 2016 | 30.39301125 | 38.20705215 | 24.04041344 |
| DALYs | East Asia | 2017 | 30.46844305 | 38.0323474 | 23.87867517 |
| DALYs | East Asia | 2018 | 30.24596235 | 37.99574227 | 23.59379073 |
| DALYs | East Asia | 2019 | 30.2848142 | 37.3862658 | 23.40104371 |
| DALYs | Eastern Europe | 1990 | 30.49468726 | 40.59657371 | 22.13138585 |
| DALYs | Eastern Europe | 1991 | 32.33952351 | 43.18738601 | 23.32500928 |
| DALYs | Eastern Europe | 1992 | 35.47835638 | 47.54867337 | 25.54956722 |
| DALYs | Eastern Europe | 1993 | 42.98636604 | 58.09876755 | 30.75884859 |
| DALYs | Eastern Europe | 1994 | 49.87048133 | 67.82854927 | 35.49531239 |
| DALYs | Eastern Europe | 1995 | 51.84286643 | 70.29679117 | 36.86337969 |
| DALYs | Eastern Europe | 1996 | 49.52244392 | 67.32345689 | 35.20350139 |
| DALYs | Eastern Europe | 1997 | 46.87809292 | 63.20124634 | 33.18823205 |
| DALYs | Eastern Europe | 1998 | 46.36407185 | 62.63901283 | 32.94531221 |
| DALYs | Eastern Europe | 1999 | 51.93735414 | 70.33740199 | 36.92577469 |
| DALYs | Eastern Europe | 2000 | 58.45982912 | 79.73851956 | 41.28295424 |
| DALYs | Eastern Europe | 2001 | 64.42286354 | 87.53069229 | 45.73408538 |
| DALYs | Eastern Europe | 2002 | 72.52241317 | 98.88435064 | 51.27124415 |
| DALYs | Eastern Europe | 2003 | 80.83626894 | 110.3671516 | 57.33710542 |
| DALYs | Eastern Europe | 2004 | 87.80221554 | 119.7225521 | 62.35973428 |
| DALYs | Eastern Europe | 2005 | 98.63655296 | 134.6194546 | 70.0004222 |
| DALYs | Eastern Europe | 2006 | 96.27410208 | 131.0745214 | 67.98393834 |
| DALYs | Eastern Europe | 2007 | 98.2515274 | 133.6921181 | 69.37750937 |
| DALYs | Eastern Europe | 2008 | 101.9244226 | 138.2666923 | 72.1643462 |
| DALYs | Eastern Europe | 2009 | 96.08827283 | 130.3026454 | 68.54318706 |
| DALYs | Eastern Europe | 2010 | 96.46565058 | 130.4645121 | 68.41288381 |
| DALYs | Eastern Europe | 2011 | 92.06526351 | 124.6642623 | 65.46252036 |
| DALYs | Eastern Europe | 2012 | 92.82566431 | 125.246163 | 66.1267468 |
| DALYs | Eastern Europe | 2013 | 95.45241195 | 129.8892711 | 67.79540742 |
| DALYs | Eastern Europe | 2014 | 98.83018456 | 133.7778041 | 70.2695986 |
| DALYs | Eastern Europe | 2015 | 105.029169 | 141.8247174 | 74.56903439 |
| DALYs | Eastern Europe | 2016 | 100.5905803 | 136.0618571 | 71.62020738 |
| DALYs | Eastern Europe | 2017 | 94.43990123 | 128.6402913 | 67.44069212 |
| DALYs | Eastern Europe | 2018 | 92.39726865 | 127.3900306 | 64.84906399 |
| DALYs | Eastern Europe | 2019 | 91.22181713 | 127.0858411 | 63.75438956 |
| DALYs | Eastern Sub-Saharan Africa | 1990 | 120.2293515 | 167.0822046 | 83.43026876 |
| DALYs | Eastern Sub-Saharan Africa | 1991 | 120.0958123 | 167.1078338 | 83.86355337 |
| DALYs | Eastern Sub-Saharan Africa | 1992 | 119.9804033 | 167.2152299 | 83.85654565 |
| DALYs | Eastern Sub-Saharan Africa | 1993 | 119.4763348 | 164.5836445 | 84.04811994 |
| DALYs | Eastern Sub-Saharan Africa | 1994 | 119.0090236 | 163.8427848 | 83.14085679 |
| DALYs | Eastern Sub-Saharan Africa | 1995 | 119.130338 | 163.6640421 | 83.24319636 |
| DALYs | Eastern Sub-Saharan Africa | 1996 | 118.5537066 | 161.1650401 | 83.38751239 |
| DALYs | Eastern Sub-Saharan Africa | 1997 | 117.8387633 | 162.2003002 | 82.93318198 |
| DALYs | Eastern Sub-Saharan Africa | 1998 | 117.6060044 | 159.6077141 | 83.49529062 |
| DALYs | Eastern Sub-Saharan Africa | 1999 | 117.1657329 | 161.2887625 | 83.07316336 |
| DALYs | Eastern Sub-Saharan Africa | 2000 | 116.2381308 | 158.6787011 | 82.53398899 |
| DALYs | Eastern Sub-Saharan Africa | 2001 | 114.5356824 | 156.4974235 | 81.3776898 |
| DALYs | Eastern Sub-Saharan Africa | 2002 | 113.6375252 | 155.4149821 | 81.04776852 |
| DALYs | Eastern Sub-Saharan Africa | 2003 | 113.1837286 | 154.504686 | 80.79401529 |
| DALYs | Eastern Sub-Saharan Africa | 2004 | 112.6850195 | 152.1610168 | 80.41481725 |
| DALYs | Eastern Sub-Saharan Africa | 2005 | 111.7734107 | 151.1473027 | 80.76010119 |
| DALYs | Eastern Sub-Saharan Africa | 2006 | 110.0146666 | 150.3148122 | 79.33896658 |
| DALYs | Eastern Sub-Saharan Africa | 2007 | 108.1902707 | 146.9671861 | 78.23860711 |
| DALYs | Eastern Sub-Saharan Africa | 2008 | 106.6592593 | 143.4319807 | 77.45206947 |
| DALYs | Eastern Sub-Saharan Africa | 2009 | 105.3444453 | 142.4956178 | 76.28745245 |
| DALYs | Eastern Sub-Saharan Africa | 2010 | 104.4253361 | 140.9282674 | 75.36516967 |
| DALYs | Eastern Sub-Saharan Africa | 2011 | 103.1267944 | 139.8544358 | 74.22306939 |
| DALYs | Eastern Sub-Saharan Africa | 2012 | 102.5934513 | 138.8861586 | 73.30170269 |
| DALYs | Eastern Sub-Saharan Africa | 2013 | 102.7962722 | 139.1767102 | 73.48962638 |
| DALYs | Eastern Sub-Saharan Africa | 2014 | 102.6760842 | 140.5911335 | 73.10776195 |
| DALYs | Eastern Sub-Saharan Africa | 2015 | 101.9770268 | 139.7322054 | 72.30325734 |
| DALYs | Eastern Sub-Saharan Africa | 2016 | 101.4544883 | 139.3311322 | 72.05582384 |
| DALYs | Eastern Sub-Saharan Africa | 2017 | 101.8158456 | 139.4046914 | 72.14404295 |
| DALYs | Eastern Sub-Saharan Africa | 2018 | 101.4470841 | 138.418048 | 71.68831603 |
| DALYs | Eastern Sub-Saharan Africa | 2019 | 101.3206442 | 139.1942129 | 71.93952237 |
| DALYs | Global | 1990 | 63.27695445 | 80.86191797 | 48.57578191 |
| DALYs | Global | 1991 | 63.40766764 | 80.47155246 | 48.73902102 |
| DALYs | Global | 1992 | 63.58901241 | 81.75094599 | 49.13441042 |
| DALYs | Global | 1993 | 64.21842754 | 81.96901359 | 49.47041377 |
| DALYs | Global | 1994 | 64.6269623 | 82.50147667 | 49.54493763 |
| DALYs | Global | 1995 | 64.44968144 | 81.91798756 | 50.07739315 |
| DALYs | Global | 1996 | 63.78457133 | 81.84482645 | 49.33681803 |
| DALYs | Global | 1997 | 62.89076152 | 80.18000974 | 48.55102172 |
| DALYs | Global | 1998 | 61.93091515 | 77.99970675 | 47.47336722 |
| DALYs | Global | 1999 | 61.08056302 | 77.06894748 | 47.49754847 |
| DALYs | Global | 2000 | 60.11542991 | 76.18292113 | 46.55468782 |
| DALYs | Global | 2001 | 58.99578891 | 74.87176602 | 45.82030848 |
| DALYs | Global | 2002 | 58.07382269 | 73.46443967 | 44.54984111 |
| DALYs | Global | 2003 | 57.24940926 | 72.59929773 | 43.65692518 |
| DALYs | Global | 2004 | 56.21810773 | 71.92393595 | 42.89886855 |
| DALYs | Global | 2005 | 55.97703892 | 71.54830512 | 42.63506383 |
| DALYs | Global | 2006 | 55.06090214 | 70.05009927 | 41.95297328 |
| DALYs | Global | 2007 | 54.56538511 | 69.45399536 | 41.46565428 |
| DALYs | Global | 2008 | 54.59242021 | 69.78404001 | 41.67176881 |
| DALYs | Global | 2009 | 53.93084502 | 68.84170285 | 41.30511097 |
| DALYs | Global | 2010 | 53.35614147 | 67.90144433 | 41.0423737 |
| DALYs | Global | 2011 | 52.82459074 | 67.22502064 | 40.27504018 |
| DALYs | Global | 2012 | 52.72877206 | 67.36748394 | 40.31195159 |
| DALYs | Global | 2013 | 52.96092066 | 67.39469881 | 40.52737199 |
| DALYs | Global | 2014 | 53.13555531 | 67.6987777 | 40.51264884 |
| DALYs | Global | 2015 | 53.45247784 | 67.97524216 | 41.21105647 |
| DALYs | Global | 2016 | 53.60313636 | 68.26292694 | 41.12070754 |
| DALYs | Global | 2017 | 53.77686116 | 68.72818884 | 40.88091026 |
| DALYs | Global | 2018 | 53.55825682 | 68.27580765 | 41.08285641 |
| DALYs | Global | 2019 | 53.3267928 | 68.28922947 | 40.7299943 |
| DALYs | High-income Asia Pacific | 1990 | 27.97234997 | 34.19824467 | 22.6302811 |
| DALYs | High-income Asia Pacific | 1991 | 27.99294831 | 34.03745605 | 22.72118946 |
| DALYs | High-income Asia Pacific | 1992 | 28.11244787 | 34.14076677 | 22.85276695 |
| DALYs | High-income Asia Pacific | 1993 | 28.23308943 | 34.30478749 | 23.06636189 |
| DALYs | High-income Asia Pacific | 1994 | 28.23215567 | 34.12742277 | 23.23028665 |
| DALYs | High-income Asia Pacific | 1995 | 28.57967005 | 34.21901271 | 23.60836959 |
| DALYs | High-income Asia Pacific | 1996 | 28.58987826 | 33.87356419 | 23.83491488 |
| DALYs | High-income Asia Pacific | 1997 | 28.95745157 | 33.95134022 | 24.26207377 |
| DALYs | High-income Asia Pacific | 1998 | 29.85775942 | 34.86625053 | 25.08962137 |
| DALYs | High-income Asia Pacific | 1999 | 30.3668698 | 35.43526893 | 25.58055231 |
| DALYs | High-income Asia Pacific | 2000 | 30.03455607 | 34.83289132 | 25.35485704 |
| DALYs | High-income Asia Pacific | 2001 | 29.24734537 | 34.0474589 | 24.64198023 |
| DALYs | High-income Asia Pacific | 2002 | 28.22329152 | 32.80943219 | 23.7551001 |
| DALYs | High-income Asia Pacific | 2003 | 27.25253248 | 31.68681017 | 22.87657305 |
| DALYs | High-income Asia Pacific | 2004 | 26.05056109 | 30.40689596 | 21.89316657 |
| DALYs | High-income Asia Pacific | 2005 | 24.90628589 | 29.06487085 | 20.91496526 |
| DALYs | High-income Asia Pacific | 2006 | 23.81116973 | 27.81079107 | 19.96815783 |
| DALYs | High-income Asia Pacific | 2007 | 22.79217287 | 26.63140384 | 19.05734391 |
| DALYs | High-income Asia Pacific | 2008 | 21.85294353 | 25.57510422 | 18.23966685 |
| DALYs | High-income Asia Pacific | 2009 | 20.98270974 | 24.63266542 | 17.53812527 |
| DALYs | High-income Asia Pacific | 2010 | 20.32288324 | 23.92778678 | 16.9742067 |
| DALYs | High-income Asia Pacific | 2011 | 19.81658272 | 23.3492175 | 16.52937803 |
| DALYs | High-income Asia Pacific | 2012 | 19.32504274 | 22.74621944 | 16.09460318 |
| DALYs | High-income Asia Pacific | 2013 | 18.92851203 | 22.37226685 | 15.64143258 |
| DALYs | High-income Asia Pacific | 2014 | 18.54278905 | 21.87619411 | 15.25494078 |
| DALYs | High-income Asia Pacific | 2015 | 18.08677436 | 21.42263066 | 14.81364264 |
| DALYs | High-income Asia Pacific | 2016 | 17.54758148 | 21.01829829 | 14.32066186 |
| DALYs | High-income Asia Pacific | 2017 | 17.17909628 | 20.74978584 | 13.88156548 |
| DALYs | High-income Asia Pacific | 2018 | 17.23136119 | 20.81910763 | 13.95646349 |
| DALYs | High-income Asia Pacific | 2019 | 17.2472455 | 20.85610486 | 13.93949859 |
| DALYs | High-income North America | 1990 | 35.82766377 | 47.45982054 | 26.33329314 |
| DALYs | High-income North America | 1991 | 35.55868695 | 47.01207993 | 26.16881908 |
| DALYs | High-income North America | 1992 | 35.22570727 | 46.32820273 | 25.97364985 |
| DALYs | High-income North America | 1993 | 35.62440829 | 46.83787829 | 26.40038544 |
| DALYs | High-income North America | 1994 | 35.62484737 | 46.86322284 | 26.5146438 |
| DALYs | High-income North America | 1995 | 35.69335586 | 46.89491148 | 26.56141132 |
| DALYs | High-income North America | 1996 | 35.11201513 | 46.02061674 | 26.20579291 |
| DALYs | High-income North America | 1997 | 34.25582466 | 44.84941181 | 25.61064684 |
| DALYs | High-income North America | 1998 | 33.48220056 | 43.75806393 | 25.07377156 |
| DALYs | High-income North America | 1999 | 33.09321616 | 43.15726987 | 24.94334448 |
| DALYs | High-income North America | 2000 | 33.36275215 | 43.39556309 | 25.14682849 |
| DALYs | High-income North America | 2001 | 34.14617207 | 44.42865359 | 25.76973254 |
| DALYs | High-income North America | 2002 | 34.92813732 | 45.41345656 | 26.34538999 |
| DALYs | High-income North America | 2003 | 35.54978995 | 46.20562377 | 26.90053247 |
| DALYs | High-income North America | 2004 | 35.55381222 | 46.15002381 | 26.95690486 |
| DALYs | High-income North America | 2005 | 36.2886907 | 47.10471256 | 27.49764448 |
| DALYs | High-income North America | 2006 | 36.50436324 | 47.29684089 | 27.71008531 |
| DALYs | High-income North America | 2007 | 36.42586604 | 47.12265473 | 27.62903277 |
| DALYs | High-income North America | 2008 | 36.55748049 | 47.26308855 | 27.76730515 |
| DALYs | High-income North America | 2009 | 36.70111177 | 47.68206641 | 27.91224581 |
| DALYs | High-income North America | 2010 | 36.60009125 | 47.5714838 | 27.83869417 |
| DALYs | High-income North America | 2011 | 37.58021357 | 48.8841464 | 28.64464289 |
| DALYs | High-income North America | 2012 | 38.91522491 | 50.77668314 | 29.6205262 |
| DALYs | High-income North America | 2013 | 40.51730003 | 53.11007378 | 30.72611553 |
| DALYs | High-income North America | 2014 | 42.15607464 | 55.27585083 | 31.99724129 |
| DALYs | High-income North America | 2015 | 43.28577244 | 56.89894444 | 32.80969439 |
| DALYs | High-income North America | 2016 | 44.08102851 | 57.9263975 | 33.49136208 |
| DALYs | High-income North America | 2017 | 43.71656294 | 57.37225552 | 33.08611893 |
| DALYs | High-income North America | 2018 | 43.43233228 | 56.87722122 | 32.80659504 |
| DALYs | High-income North America | 2019 | 43.04106919 | 56.59551157 | 32.53807419 |
| DALYs | North Africa and Middle East | 1990 | 82.70197774 | 110.8164569 | 59.8469655 |
| DALYs | North Africa and Middle East | 1991 | 81.13348251 | 108.5841287 | 58.42687622 |
| DALYs | North Africa and Middle East | 1992 | 79.63058236 | 105.6652542 | 57.40546027 |
| DALYs | North Africa and Middle East | 1993 | 78.80740957 | 104.2467927 | 57.22347369 |
| DALYs | North Africa and Middle East | 1994 | 77.92941536 | 102.835948 | 56.95545864 |
| DALYs | North Africa and Middle East | 1995 | 76.28424825 | 100.6635051 | 56.1280086 |
| DALYs | North Africa and Middle East | 1996 | 75.10501759 | 98.59540237 | 55.72333437 |
| DALYs | North Africa and Middle East | 1997 | 74.53158489 | 97.6975634 | 55.17693551 |
| DALYs | North Africa and Middle East | 1998 | 73.79833247 | 96.59433662 | 54.64697802 |
| DALYs | North Africa and Middle East | 1999 | 72.97069936 | 95.32698571 | 54.10499208 |
| DALYs | North Africa and Middle East | 2000 | 71.15935467 | 92.64321147 | 52.74243125 |
| DALYs | North Africa and Middle East | 2001 | 72.30492077 | 93.89952302 | 53.59983592 |
| DALYs | North Africa and Middle East | 2002 | 73.90499843 | 96.38099351 | 55.00836575 |
| DALYs | North Africa and Middle East | 2003 | 75.9539736 | 99.46071342 | 56.39951801 |
| DALYs | North Africa and Middle East | 2004 | 76.65717659 | 100.510703 | 56.72909789 |
| DALYs | North Africa and Middle East | 2005 | 76.16553364 | 99.92144185 | 56.49646161 |
| DALYs | North Africa and Middle East | 2006 | 75.51574064 | 98.94909391 | 55.91183501 |
| DALYs | North Africa and Middle East | 2007 | 75.08455645 | 98.48458108 | 55.66463127 |
| DALYs | North Africa and Middle East | 2008 | 75.66327569 | 98.88346834 | 56.05195738 |
| DALYs | North Africa and Middle East | 2009 | 76.51754525 | 99.03485458 | 56.46598085 |
| DALYs | North Africa and Middle East | 2010 | 76.4962562 | 99.41963096 | 56.49413075 |
| DALYs | North Africa and Middle East | 2011 | 76.26267963 | 99.4293448 | 56.0708733 |
| DALYs | North Africa and Middle East | 2012 | 76.82618692 | 100.0623768 | 56.28399904 |
| DALYs | North Africa and Middle East | 2013 | 76.20521217 | 99.4404086 | 56.381764 |
| DALYs | North Africa and Middle East | 2014 | 76.64393331 | 99.98455798 | 56.1657341 |
| DALYs | North Africa and Middle East | 2015 | 77.36770586 | 101.3123565 | 56.01894683 |
| DALYs | North Africa and Middle East | 2016 | 77.36774319 | 102.9750636 | 55.71705248 |
| DALYs | North Africa and Middle East | 2017 | 76.79245267 | 103.8067169 | 54.4319661 |
| DALYs | North Africa and Middle East | 2018 | 76.45027733 | 103.9084595 | 54.20777501 |
| DALYs | North Africa and Middle East | 2019 | 76.38758828 | 104.6067308 | 53.55784509 |
| DALYs | Oceania | 1990 | 42.59301746 | 59.09497958 | 30.35734526 |
| DALYs | Oceania | 1991 | 42.63681969 | 60.13601114 | 30.25328478 |
| DALYs | Oceania | 1992 | 42.46601729 | 59.67385775 | 30.20679521 |
| DALYs | Oceania | 1993 | 42.12087926 | 59.06994683 | 29.9209038 |
| DALYs | Oceania | 1994 | 41.81023665 | 58.19374278 | 29.55630481 |
| DALYs | Oceania | 1995 | 41.46852743 | 57.75731709 | 29.34121329 |
| DALYs | Oceania | 1996 | 41.55568081 | 57.39461015 | 29.52796204 |
| DALYs | Oceania | 1997 | 41.73101009 | 57.62377979 | 29.77806357 |
| DALYs | Oceania | 1998 | 42.10950647 | 57.98807535 | 30.0434273 |
| DALYs | Oceania | 1999 | 42.64175281 | 58.96653943 | 30.3673916 |
| DALYs | Oceania | 2000 | 42.37145629 | 58.38217164 | 30.28092349 |
| DALYs | Oceania | 2001 | 41.90063195 | 58.32606739 | 30.17857957 |
| DALYs | Oceania | 2002 | 41.60860093 | 57.78503151 | 29.76411227 |
| DALYs | Oceania | 2003 | 41.5860185 | 58.0405261 | 29.67472412 |
| DALYs | Oceania | 2004 | 41.32937423 | 57.35258936 | 29.37498954 |
| DALYs | Oceania | 2005 | 41.2821627 | 57.28112601 | 29.26647701 |
| DALYs | Oceania | 2006 | 41.42743298 | 57.09969592 | 29.20726176 |
| DALYs | Oceania | 2007 | 41.49175299 | 57.73655651 | 29.08440972 |
| DALYs | Oceania | 2008 | 41.2550593 | 57.89628018 | 28.91024272 |
| DALYs | Oceania | 2009 | 41.13300812 | 57.84006908 | 28.6525481 |
| DALYs | Oceania | 2010 | 41.36430186 | 58.46896411 | 29.02037588 |
| DALYs | Oceania | 2011 | 41.66719519 | 58.45461035 | 29.16921994 |
| DALYs | Oceania | 2012 | 41.56898835 | 58.44855078 | 29.04317499 |
| DALYs | Oceania | 2013 | 41.29529722 | 57.91953812 | 28.79665426 |
| DALYs | Oceania | 2014 | 41.16910502 | 58.09003273 | 28.441215 |
| DALYs | Oceania | 2015 | 41.12795778 | 57.89937046 | 28.33158592 |
| DALYs | Oceania | 2016 | 40.73759381 | 56.88513386 | 27.82316477 |
| DALYs | Oceania | 2017 | 40.59645261 | 56.93146797 | 27.57408134 |
| DALYs | Oceania | 2018 | 40.35220876 | 56.62872136 | 27.31294012 |
| DALYs | Oceania | 2019 | 40.14831903 | 56.21910985 | 27.1112278 |
| DALYs | South Asia | 1990 | 54.98087235 | 72.78469908 | 40.84595195 |
| DALYs | South Asia | 1991 | 55.31615346 | 73.70990046 | 41.20481867 |
| DALYs | South Asia | 1992 | 55.63311213 | 74.22794713 | 41.49690756 |
| DALYs | South Asia | 1993 | 56.52754377 | 74.46237667 | 41.67370581 |
| DALYs | South Asia | 1994 | 57.37765065 | 76.32211548 | 42.93109174 |
| DALYs | South Asia | 1995 | 57.26411506 | 75.16926999 | 42.8953045 |
| DALYs | South Asia | 1996 | 57.24474734 | 75.57966353 | 43.16435919 |
| DALYs | South Asia | 1997 | 56.860936 | 74.80768844 | 42.88267668 |
| DALYs | South Asia | 1998 | 54.1927397 | 70.16867073 | 40.95447307 |
| DALYs | South Asia | 1999 | 51.1267095 | 65.97521374 | 39.00164174 |
| DALYs | South Asia | 2000 | 49.14100764 | 62.93572748 | 37.49486294 |
| DALYs | South Asia | 2001 | 47.76301714 | 61.48203385 | 36.47904304 |
| DALYs | South Asia | 2002 | 46.06937462 | 59.09230234 | 34.98229257 |
| DALYs | South Asia | 2003 | 43.85334138 | 56.48630647 | 33.32858708 |
| DALYs | South Asia | 2004 | 41.73356065 | 54.35741515 | 31.70207461 |
| DALYs | South Asia | 2005 | 41.71225822 | 54.05731038 | 31.41839528 |
| DALYs | South Asia | 2006 | 41.72130369 | 53.46392496 | 31.42048705 |
| DALYs | South Asia | 2007 | 41.7046425 | 54.05071461 | 31.77965011 |
| DALYs | South Asia | 2008 | 41.7172996 | 54.06604259 | 31.64429614 |
| DALYs | South Asia | 2009 | 40.37571229 | 51.95588909 | 31.02180717 |
| DALYs | South Asia | 2010 | 40.06097788 | 51.50046622 | 30.7894361 |
| DALYs | South Asia | 2011 | 40.25384347 | 51.53164439 | 30.81351119 |
| DALYs | South Asia | 2012 | 40.07358388 | 51.57965262 | 30.5555238 |
| DALYs | South Asia | 2013 | 40.36628268 | 52.01267093 | 30.70505181 |
| DALYs | South Asia | 2014 | 39.91764749 | 51.11678297 | 30.81184724 |
| DALYs | South Asia | 2015 | 40.52860788 | 52.40948247 | 30.98984276 |
| DALYs | South Asia | 2016 | 41.48641321 | 53.51589284 | 31.02264111 |
| DALYs | South Asia | 2017 | 42.7647083 | 56.82551502 | 31.36335449 |
| DALYs | South Asia | 2018 | 42.52559996 | 56.11344746 | 31.54142068 |
| DALYs | South Asia | 2019 | 41.62189596 | 54.68835748 | 31.43364886 |
| DALYs | Southeast Asia | 1990 | 109.7586456 | 144.4869507 | 81.1524913 |
| DALYs | Southeast Asia | 1991 | 109.4167865 | 144.5500068 | 81.08200743 |
| DALYs | Southeast Asia | 1992 | 109.1854358 | 141.9802586 | 80.86604009 |
| DALYs | Southeast Asia | 1993 | 108.9151422 | 143.1679123 | 81.0392256 |
| DALYs | Southeast Asia | 1994 | 108.7425899 | 143.4409981 | 80.80266286 |
| DALYs | Southeast Asia | 1995 | 108.4436346 | 142.170089 | 80.43474909 |
| DALYs | Southeast Asia | 1996 | 108.0059275 | 140.2593691 | 80.05134882 |
| DALYs | Southeast Asia | 1997 | 106.2713259 | 138.0486045 | 79.87285808 |
| DALYs | Southeast Asia | 1998 | 105.4031261 | 137.6008913 | 78.514753 |
| DALYs | Southeast Asia | 1999 | 105.2500678 | 136.2578699 | 79.14696345 |
| DALYs | Southeast Asia | 2000 | 104.5143186 | 136.5561975 | 78.62367866 |
| DALYs | Southeast Asia | 2001 | 102.9619014 | 134.1992299 | 77.49198931 |
| DALYs | Southeast Asia | 2002 | 102.3857842 | 132.1543085 | 76.93933922 |
| DALYs | Southeast Asia | 2003 | 101.6505595 | 132.0483619 | 76.44608464 |
| DALYs | Southeast Asia | 2004 | 100.9697558 | 130.1166606 | 76.30354768 |
| DALYs | Southeast Asia | 2005 | 100.2602396 | 129.3196217 | 75.78383497 |
| DALYs | Southeast Asia | 2006 | 99.994759 | 127.5313409 | 75.50922117 |
| DALYs | Southeast Asia | 2007 | 100.1622854 | 129.3008859 | 75.95971839 |
| DALYs | Southeast Asia | 2008 | 100.9630276 | 129.2441581 | 76.57239775 |
| DALYs | Southeast Asia | 2009 | 101.5952663 | 130.5978506 | 76.91527263 |
| DALYs | Southeast Asia | 2010 | 101.7424032 | 131.3739283 | 77.60281821 |
| DALYs | Southeast Asia | 2011 | 101.7217282 | 129.5440775 | 76.5583148 |
| DALYs | Southeast Asia | 2012 | 101.2437279 | 129.3229196 | 76.96925041 |
| DALYs | Southeast Asia | 2013 | 101.0235851 | 128.9591558 | 77.02594763 |
| DALYs | Southeast Asia | 2014 | 100.9654712 | 128.2342569 | 76.32126316 |
| DALYs | Southeast Asia | 2015 | 99.76570646 | 127.4537589 | 76.08027579 |
| DALYs | Southeast Asia | 2016 | 99.48690322 | 127.5356337 | 75.25233499 |
| DALYs | Southeast Asia | 2017 | 99.5115418 | 128.4639292 | 75.12365308 |
| DALYs | Southeast Asia | 2018 | 98.91650995 | 127.8716462 | 73.61758392 |
| DALYs | Southeast Asia | 2019 | 98.1617076 | 126.9188742 | 73.27571723 |
| DALYs | Southern Latin America | 1990 | 55.24465835 | 77.69433827 | 38.07690996 |
| DALYs | Southern Latin America | 1991 | 52.44387327 | 73.24214084 | 36.15671074 |
| DALYs | Southern Latin America | 1992 | 50.52524169 | 70.2480316 | 34.86502374 |
| DALYs | Southern Latin America | 1993 | 49.27540659 | 68.16679199 | 34.07679902 |
| DALYs | Southern Latin America | 1994 | 48.74010591 | 67.6807593 | 33.56553024 |
| DALYs | Southern Latin America | 1995 | 48.87982368 | 67.88995419 | 33.48628472 |
| DALYs | Southern Latin America | 1996 | 48.84767448 | 67.81464759 | 33.48874403 |
| DALYs | Southern Latin America | 1997 | 48.02330975 | 66.98302284 | 32.85808103 |
| DALYs | Southern Latin America | 1998 | 47.23917801 | 65.90419964 | 32.5969343 |
| DALYs | Southern Latin America | 1999 | 46.33357668 | 64.68982723 | 32.06494624 |
| DALYs | Southern Latin America | 2000 | 45.0283331 | 62.8924492 | 31.28108722 |
| DALYs | Southern Latin America | 2001 | 45.18281309 | 62.95886008 | 31.28603029 |
| DALYs | Southern Latin America | 2002 | 45.04642392 | 63.08608799 | 31.16387888 |
| DALYs | Southern Latin America | 2003 | 45.00220069 | 63.03724979 | 31.21959285 |
| DALYs | Southern Latin America | 2004 | 44.02555478 | 61.83999208 | 30.57679123 |
| DALYs | Southern Latin America | 2005 | 43.24630515 | 60.36274297 | 30.01770546 |
| DALYs | Southern Latin America | 2006 | 43.37832212 | 60.56053188 | 30.31986479 |
| DALYs | Southern Latin America | 2007 | 44.54804587 | 61.82910221 | 31.21805901 |
| DALYs | Southern Latin America | 2008 | 44.37933445 | 61.53435377 | 31.1687682 |
| DALYs | Southern Latin America | 2009 | 45.09095206 | 62.81141651 | 31.56344471 |
| DALYs | Southern Latin America | 2010 | 45.3085587 | 62.99982415 | 31.80438882 |
| DALYs | Southern Latin America | 2011 | 45.53143914 | 63.02389972 | 31.98950042 |
| DALYs | Southern Latin America | 2012 | 46.22681267 | 64.0239074 | 32.5318729 |
| DALYs | Southern Latin America | 2013 | 46.94903788 | 64.69580438 | 33.10135897 |
| DALYs | Southern Latin America | 2014 | 47.03722125 | 64.58498675 | 33.18097039 |
| DALYs | Southern Latin America | 2015 | 47.72643077 | 65.46328605 | 33.74110611 |
| DALYs | Southern Latin America | 2016 | 48.29099692 | 66.35613931 | 34.09097481 |
| DALYs | Southern Latin America | 2017 | 48.26573888 | 66.60249372 | 33.85781744 |
| DALYs | Southern Latin America | 2018 | 47.79974441 | 66.24998755 | 33.33221742 |
| DALYs | Southern Latin America | 2019 | 47.55766503 | 65.98746294 | 33.38912504 |
| DALYs | Southern Sub-Saharan Africa | 1990 | 66.16880003 | 90.53605362 | 46.96457484 |
| DALYs | Southern Sub-Saharan Africa | 1991 | 66.80572843 | 90.71254095 | 46.98598731 |
| DALYs | Southern Sub-Saharan Africa | 1992 | 69.82225916 | 93.15011718 | 50.47011731 |
| DALYs | Southern Sub-Saharan Africa | 1993 | 68.72308692 | 92.25454604 | 48.80540635 |
| DALYs | Southern Sub-Saharan Africa | 1994 | 71.83793241 | 95.21954055 | 51.56735191 |
| DALYs | Southern Sub-Saharan Africa | 1995 | 72.63645388 | 96.15604747 | 52.98064959 |
| DALYs | Southern Sub-Saharan Africa | 1996 | 75.0073356 | 97.49925112 | 55.76768577 |
| DALYs | Southern Sub-Saharan Africa | 1997 | 80.98048701 | 103.3612906 | 62.07014954 |
| DALYs | Southern Sub-Saharan Africa | 1998 | 82.93354333 | 104.1088518 | 64.46134785 |
| DALYs | Southern Sub-Saharan Africa | 1999 | 82.07754455 | 102.119737 | 64.18017242 |
| DALYs | Southern Sub-Saharan Africa | 2000 | 83.96799912 | 103.9792681 | 66.10241423 |
| DALYs | Southern Sub-Saharan Africa | 2001 | 82.7530247 | 101.9490695 | 65.6227144 |
| DALYs | Southern Sub-Saharan Africa | 2002 | 83.23687275 | 103.026159 | 65.5830267 |
| DALYs | Southern Sub-Saharan Africa | 2003 | 84.20913816 | 105.3628023 | 66.5850981 |
| DALYs | Southern Sub-Saharan Africa | 2004 | 84.24049918 | 106.2337146 | 66.5484175 |
| DALYs | Southern Sub-Saharan Africa | 2005 | 82.96281624 | 105.4032213 | 64.75420062 |
| DALYs | Southern Sub-Saharan Africa | 2006 | 82.69971171 | 105.1833702 | 64.77613096 |
| DALYs | Southern Sub-Saharan Africa | 2007 | 79.98371674 | 102.1492358 | 62.21907608 |
| DALYs | Southern Sub-Saharan Africa | 2008 | 78.95059578 | 100.5857362 | 61.83105257 |
| DALYs | Southern Sub-Saharan Africa | 2009 | 77.60129973 | 98.58736218 | 60.474711 |
| DALYs | Southern Sub-Saharan Africa | 2010 | 75.32617896 | 95.32874665 | 58.91107464 |
| DALYs | Southern Sub-Saharan Africa | 2011 | 71.10766783 | 90.24674001 | 55.66298673 |
| DALYs | Southern Sub-Saharan Africa | 2012 | 68.57272107 | 86.52758804 | 54.05956255 |
| DALYs | Southern Sub-Saharan Africa | 2013 | 66.83696347 | 84.92973437 | 52.63117028 |
| DALYs | Southern Sub-Saharan Africa | 2014 | 66.31254362 | 84.02460279 | 52.18356341 |
| DALYs | Southern Sub-Saharan Africa | 2015 | 65.02591563 | 83.10262957 | 50.89114938 |
| DALYs | Southern Sub-Saharan Africa | 2016 | 63.25477075 | 81.34258889 | 49.63598231 |
| DALYs | Southern Sub-Saharan Africa | 2017 | 62.72400858 | 80.43873178 | 48.69237364 |
| DALYs | Southern Sub-Saharan Africa | 2018 | 60.92863154 | 76.99280527 | 47.81519453 |
| DALYs | Southern Sub-Saharan Africa | 2019 | 59.96550401 | 75.86641012 | 47.09964955 |
| DALYs | Tropical Latin America | 1990 | 60.38428068 | 79.54521612 | 44.39748144 |
| DALYs | Tropical Latin America | 1991 | 58.58808907 | 76.87820255 | 43.10052401 |
| DALYs | Tropical Latin America | 1992 | 57.82006441 | 76.07144259 | 42.31141064 |
| DALYs | Tropical Latin America | 1993 | 58.37244842 | 76.88226403 | 43.11998972 |
| DALYs | Tropical Latin America | 1994 | 57.42695425 | 75.57529054 | 42.17696531 |
| DALYs | Tropical Latin America | 1995 | 56.39717619 | 74.28991839 | 41.51992973 |
| DALYs | Tropical Latin America | 1996 | 55.14216005 | 72.55677445 | 40.57767564 |
| DALYs | Tropical Latin America | 1997 | 53.62504969 | 70.54989007 | 39.57584903 |
| DALYs | Tropical Latin America | 1998 | 52.98771919 | 69.85416586 | 38.973091 |
| DALYs | Tropical Latin America | 1999 | 52.18811484 | 68.73163886 | 38.53006899 |
| DALYs | Tropical Latin America | 2000 | 51.27131393 | 67.71701113 | 37.76448139 |
| DALYs | Tropical Latin America | 2001 | 50.69923282 | 66.82973659 | 37.46370221 |
| DALYs | Tropical Latin America | 2002 | 50.23412594 | 66.18135574 | 37.02167578 |
| DALYs | Tropical Latin America | 2003 | 49.90215287 | 65.87990447 | 36.94490928 |
| DALYs | Tropical Latin America | 2004 | 49.72359214 | 65.42425896 | 36.5319797 |
| DALYs | Tropical Latin America | 2005 | 48.3999763 | 63.83646962 | 35.9499614 |
| DALYs | Tropical Latin America | 2006 | 48.50836622 | 64.09514583 | 35.81525136 |
| DALYs | Tropical Latin America | 2007 | 49.36493804 | 64.78578245 | 36.42503566 |
| DALYs | Tropical Latin America | 2008 | 50.67216443 | 66.61051272 | 37.64186343 |
| DALYs | Tropical Latin America | 2009 | 51.79945849 | 67.91843298 | 38.12589724 |
| DALYs | Tropical Latin America | 2010 | 52.22555235 | 68.44824512 | 38.54153454 |
| DALYs | Tropical Latin America | 2011 | 52.14274938 | 68.16607089 | 38.56271135 |
| DALYs | Tropical Latin America | 2012 | 51.34449918 | 67.20270597 | 38.12790452 |
| DALYs | Tropical Latin America | 2013 | 50.87601945 | 66.31757018 | 37.65395267 |
| DALYs | Tropical Latin America | 2014 | 50.07123534 | 64.77306765 | 37.29946208 |
| DALYs | Tropical Latin America | 2015 | 49.63747925 | 64.39611759 | 36.6950628 |
| DALYs | Tropical Latin America | 2016 | 51.60833155 | 67.83040629 | 37.9930097 |
| DALYs | Tropical Latin America | 2017 | 51.67619569 | 67.79474778 | 38.35879659 |
| DALYs | Tropical Latin America | 2018 | 51.64844462 | 67.78156712 | 37.86345215 |
| DALYs | Tropical Latin America | 2019 | 51.56439939 | 67.46261455 | 37.8763052 |
| DALYs | Western Europe | 1990 | 49.06584047 | 65.56490635 | 35.48238665 |
| DALYs | Western Europe | 1991 | 49.10217735 | 65.42890744 | 35.63533217 |
| DALYs | Western Europe | 1992 | 48.54763892 | 64.63123408 | 35.31233335 |
| DALYs | Western Europe | 1993 | 48.28589117 | 64.16081317 | 35.12054155 |
| DALYs | Western Europe | 1994 | 47.76279952 | 63.44725747 | 34.7296192 |
| DALYs | Western Europe | 1995 | 47.34418243 | 62.74260382 | 34.54328678 |
| DALYs | Western Europe | 1996 | 46.85159119 | 62.1856939 | 34.125608 |
| DALYs | Western Europe | 1997 | 46.2977042 | 61.37841931 | 33.75909772 |
| DALYs | Western Europe | 1998 | 45.94713485 | 60.6868987 | 33.47994116 |
| DALYs | Western Europe | 1999 | 45.4981769 | 60.0272567 | 33.17401942 |
| DALYs | Western Europe | 2000 | 44.66538033 | 59.01082126 | 32.65406321 |
| DALYs | Western Europe | 2001 | 43.88840932 | 57.93563056 | 32.1940158 |
| DALYs | Western Europe | 2002 | 43.3387847 | 57.18134517 | 31.84656129 |
| DALYs | Western Europe | 2003 | 42.76195078 | 56.61788575 | 31.56844444 |
| DALYs | Western Europe | 2004 | 41.15704102 | 54.51081026 | 30.40535106 |
| DALYs | Western Europe | 2005 | 40.22122541 | 53.35978944 | 29.8023312 |
| DALYs | Western Europe | 2006 | 39.21519989 | 51.98715425 | 29.03283854 |
| DALYs | Western Europe | 2007 | 38.46176204 | 50.74029242 | 28.50281387 |
| DALYs | Western Europe | 2008 | 37.83239489 | 49.82933031 | 28.04010559 |
| DALYs | Western Europe | 2009 | 37.15714789 | 48.89025172 | 27.63326615 |
| DALYs | Western Europe | 2010 | 36.29111283 | 47.62463761 | 27.15560304 |
| DALYs | Western Europe | 2011 | 35.52849335 | 46.57940784 | 26.60220191 |
| DALYs | Western Europe | 2012 | 34.87110001 | 45.7944091 | 26.11867798 |
| DALYs | Western Europe | 2013 | 34.38335841 | 44.95972507 | 25.82722171 |
| DALYs | Western Europe | 2014 | 33.68274307 | 43.88788832 | 25.23309761 |
| DALYs | Western Europe | 2015 | 33.82609856 | 44.54421483 | 25.4394018 |
| DALYs | Western Europe | 2016 | 33.22123129 | 43.44442383 | 24.90651757 |
| DALYs | Western Europe | 2017 | 33.09226092 | 43.14139466 | 24.74494866 |
| DALYs | Western Europe | 2018 | 32.91802669 | 43.02743111 | 24.67211963 |
| DALYs | Western Europe | 2019 | 32.80194087 | 42.86851702 | 24.61313552 |
| DALYs | Western Sub-Saharan Africa | 1990 | 107.5088105 | 159.0309673 | 72.73185868 |
| DALYs | Western Sub-Saharan Africa | 1991 | 107.3389291 | 154.272888 | 73.28385181 |
| DALYs | Western Sub-Saharan Africa | 1992 | 107.5266023 | 155.6027194 | 72.93593209 |
| DALYs | Western Sub-Saharan Africa | 1993 | 107.328718 | 155.6190859 | 73.79256176 |
| DALYs | Western Sub-Saharan Africa | 1994 | 106.8346287 | 154.5373524 | 74.17761001 |
| DALYs | Western Sub-Saharan Africa | 1995 | 105.8776139 | 150.8858173 | 73.22853369 |
| DALYs | Western Sub-Saharan Africa | 1996 | 104.723497 | 148.2741769 | 73.5991138 |
| DALYs | Western Sub-Saharan Africa | 1997 | 104.1220771 | 147.4396299 | 72.94746043 |
| DALYs | Western Sub-Saharan Africa | 1998 | 103.2688799 | 144.6080337 | 72.81796601 |
| DALYs | Western Sub-Saharan Africa | 1999 | 102.5364568 | 141.2959931 | 71.39288464 |
| DALYs | Western Sub-Saharan Africa | 2000 | 101.8386114 | 143.0306128 | 71.15291612 |
| DALYs | Western Sub-Saharan Africa | 2001 | 101.8588113 | 142.0686591 | 70.48742987 |
| DALYs | Western Sub-Saharan Africa | 2002 | 102.208151 | 143.6490133 | 71.56569044 |
| DALYs | Western Sub-Saharan Africa | 2003 | 102.0153242 | 142.7939759 | 71.36558963 |
| DALYs | Western Sub-Saharan Africa | 2004 | 102.1668548 | 145.3139488 | 72.15781664 |
| DALYs | Western Sub-Saharan Africa | 2005 | 101.9689436 | 143.2394808 | 71.64482501 |
| DALYs | Western Sub-Saharan Africa | 2006 | 100.6205053 | 140.4360632 | 71.17694206 |
| DALYs | Western Sub-Saharan Africa | 2007 | 99.36081953 | 139.9920051 | 69.21171544 |
| DALYs | Western Sub-Saharan Africa | 2008 | 97.56296511 | 138.4876312 | 67.5330315 |
| DALYs | Western Sub-Saharan Africa | 2009 | 96.70277801 | 135.1543982 | 66.54663646 |
| DALYs | Western Sub-Saharan Africa | 2010 | 96.07298399 | 133.6958137 | 66.06187995 |
| DALYs | Western Sub-Saharan Africa | 2011 | 95.41778843 | 135.8462941 | 66.00206529 |
| DALYs | Western Sub-Saharan Africa | 2012 | 95.41860392 | 135.2904903 | 66.20338938 |
| DALYs | Western Sub-Saharan Africa | 2013 | 95.27858921 | 133.1059978 | 65.89981856 |
| DALYs | Western Sub-Saharan Africa | 2014 | 94.37912063 | 132.7667122 | 65.64855276 |
| DALYs | Western Sub-Saharan Africa | 2015 | 92.33487102 | 129.3434807 | 64.11755132 |
| DALYs | Western Sub-Saharan Africa | 2016 | 91.73927788 | 129.3651387 | 63.73646692 |
| DALYs | Western Sub-Saharan Africa | 2017 | 92.08332739 | 128.3878849 | 64.02944169 |
| DALYs | Western Sub-Saharan Africa | 2018 | 90.6741187 | 126.6802213 | 63.28699062 |
| DALYs | Western Sub-Saharan Africa | 2019 | 89.75134339 | 124.8818644 | 62.20063485 |
| Deaths | Andean Latin America | 1990 | 6.100543189 | 8.192672046 | 4.450189422 |
| Deaths | Andean Latin America | 1991 | 6.011441048 | 7.959695397 | 4.462636717 |
| Deaths | Andean Latin America | 1992 | 6.220822028 | 8.291451689 | 4.455515048 |
| Deaths | Andean Latin America | 1993 | 6.26222454 | 8.28967769 | 4.540414556 |
| Deaths | Andean Latin America | 1994 | 6.290562756 | 8.255777662 | 4.567576889 |
| Deaths | Andean Latin America | 1995 | 6.338083679 | 8.361569236 | 4.67641087 |
| Deaths | Andean Latin America | 1996 | 6.230315983 | 8.19856041 | 4.600819621 |
| Deaths | Andean Latin America | 1997 | 6.195288629 | 8.173257945 | 4.561774472 |
| Deaths | Andean Latin America | 1998 | 6.203114536 | 8.179438651 | 4.54102824 |
| Deaths | Andean Latin America | 1999 | 6.00317692 | 7.987278122 | 4.368757985 |
| Deaths | Andean Latin America | 2000 | 5.940843332 | 7.837745656 | 4.329968714 |
| Deaths | Andean Latin America | 2001 | 5.747249727 | 7.537168889 | 4.178250215 |
| Deaths | Andean Latin America | 2002 | 5.820465284 | 7.675994239 | 4.227922325 |
| Deaths | Andean Latin America | 2003 | 5.89368851 | 7.74592915 | 4.311487922 |
| Deaths | Andean Latin America | 2004 | 5.825516912 | 7.73764849 | 4.24602146 |
| Deaths | Andean Latin America | 2005 | 5.817902631 | 7.720886093 | 4.272346625 |
| Deaths | Andean Latin America | 2006 | 5.777583842 | 7.687177827 | 4.211578897 |
| Deaths | Andean Latin America | 2007 | 5.713109079 | 7.562401847 | 4.194955603 |
| Deaths | Andean Latin America | 2008 | 5.742547591 | 7.522141822 | 4.212015568 |
| Deaths | Andean Latin America | 2009 | 5.994776391 | 7.941692363 | 4.396459048 |
| Deaths | Andean Latin America | 2010 | 6.053676766 | 8.105734452 | 4.446633691 |
| Deaths | Andean Latin America | 2011 | 5.997201497 | 7.998070951 | 4.376016914 |
| Deaths | Andean Latin America | 2012 | 5.99097506 | 7.945418814 | 4.397448412 |
| Deaths | Andean Latin America | 2013 | 5.94367949 | 7.857995378 | 4.331118629 |
| Deaths | Andean Latin America | 2014 | 5.832291311 | 7.716679142 | 4.23637964 |
| Deaths | Andean Latin America | 2015 | 5.749886996 | 7.593797023 | 4.14741303 |
| Deaths | Andean Latin America | 2016 | 5.746584258 | 7.72500582 | 4.126957824 |
| Deaths | Andean Latin America | 2017 | 5.734668443 | 7.669076367 | 4.073772507 |
| Deaths | Andean Latin America | 2018 | 5.71169845 | 7.625497686 | 4.030546617 |
| Deaths | Andean Latin America | 2019 | 5.675061131 | 7.710199653 | 3.932728667 |
| Deaths | Australasia | 1990 | 1.146625227 | 1.476288487 | 0.844444657 |
| Deaths | Australasia | 1991 | 1.127026002 | 1.446991805 | 0.831028019 |
| Deaths | Australasia | 1992 | 1.119603348 | 1.443229167 | 0.831903647 |
| Deaths | Australasia | 1993 | 1.110180288 | 1.429113082 | 0.829219846 |
| Deaths | Australasia | 1994 | 1.118831474 | 1.441505258 | 0.839727943 |
| Deaths | Australasia | 1995 | 1.113038964 | 1.418429025 | 0.842278398 |
| Deaths | Australasia | 1996 | 1.11429599 | 1.416510642 | 0.849441225 |
| Deaths | Australasia | 1997 | 1.101892009 | 1.398616431 | 0.844706576 |
| Deaths | Australasia | 1998 | 1.074278798 | 1.363853431 | 0.826551942 |
| Deaths | Australasia | 1999 | 1.067523504 | 1.351622756 | 0.826081381 |
| Deaths | Australasia | 2000 | 1.059734758 | 1.335551265 | 0.821730847 |
| Deaths | Australasia | 2001 | 1.06205463 | 1.334634291 | 0.822574629 |
| Deaths | Australasia | 2002 | 1.087106159 | 1.359632609 | 0.842086664 |
| Deaths | Australasia | 2003 | 1.091598644 | 1.359967015 | 0.84338361 |
| Deaths | Australasia | 2004 | 1.090979243 | 1.34798596 | 0.844555439 |
| Deaths | Australasia | 2005 | 1.091653714 | 1.352934332 | 0.845635783 |
| Deaths | Australasia | 2006 | 1.10748667 | 1.370795782 | 0.859112283 |
| Deaths | Australasia | 2007 | 1.155312858 | 1.425639713 | 0.902454741 |
| Deaths | Australasia | 2008 | 1.207456918 | 1.484833453 | 0.949657472 |
| Deaths | Australasia | 2009 | 1.242728077 | 1.531030186 | 0.974837447 |
| Deaths | Australasia | 2010 | 1.265703129 | 1.559310668 | 0.991886166 |
| Deaths | Australasia | 2011 | 1.28811404 | 1.59301706 | 1.012338074 |
| Deaths | Australasia | 2012 | 1.293735895 | 1.606085594 | 1.015428861 |
| Deaths | Australasia | 2013 | 1.320318127 | 1.639403138 | 1.033447512 |
| Deaths | Australasia | 2014 | 1.348417775 | 1.67274285 | 1.059994979 |
| Deaths | Australasia | 2015 | 1.361175419 | 1.690391637 | 1.067422265 |
| Deaths | Australasia | 2016 | 1.337868076 | 1.662019254 | 1.052362209 |
| Deaths | Australasia | 2017 | 1.316168837 | 1.638702622 | 1.033074399 |
| Deaths | Australasia | 2018 | 1.32158404 | 1.640393698 | 1.036078345 |
| Deaths | Australasia | 2019 | 1.316046469 | 1.63036633 | 1.040064686 |
| Deaths | Caribbean | 1990 | 4.083659933 | 5.234937586 | 3.078043148 |
| Deaths | Caribbean | 1991 | 4.023792038 | 5.18628963 | 3.018243663 |
| Deaths | Caribbean | 1992 | 4.016706559 | 5.190336917 | 3.004566107 |
| Deaths | Caribbean | 1993 | 4.046087339 | 5.225019097 | 3.017573762 |
| Deaths | Caribbean | 1994 | 3.960216228 | 5.115807584 | 2.979716355 |
| Deaths | Caribbean | 1995 | 3.891100736 | 5.047153709 | 2.925654695 |
| Deaths | Caribbean | 1996 | 3.806866096 | 4.931590742 | 2.84563557 |
| Deaths | Caribbean | 1997 | 3.661692938 | 4.801392835 | 2.702630681 |
| Deaths | Caribbean | 1998 | 3.613332005 | 4.7434221 | 2.668047127 |
| Deaths | Caribbean | 1999 | 3.500891932 | 4.607882494 | 2.601811411 |
| Deaths | Caribbean | 2000 | 3.371059061 | 4.425448019 | 2.50679594 |
| Deaths | Caribbean | 2001 | 3.340931854 | 4.387937225 | 2.473516128 |
| Deaths | Caribbean | 2002 | 3.232867419 | 4.249802684 | 2.403349777 |
| Deaths | Caribbean | 2003 | 3.240094857 | 4.265595105 | 2.409799616 |
| Deaths | Caribbean | 2004 | 3.250122434 | 4.311795844 | 2.424774522 |
| Deaths | Caribbean | 2005 | 3.246676615 | 4.298849803 | 2.411814674 |
| Deaths | Caribbean | 2006 | 3.13893159 | 4.175818877 | 2.312199505 |
| Deaths | Caribbean | 2007 | 3.088288655 | 4.159086529 | 2.267776071 |
| Deaths | Caribbean | 2008 | 3.067351478 | 4.140154006 | 2.254338298 |
| Deaths | Caribbean | 2009 | 3.057682615 | 4.123668573 | 2.229959329 |
| Deaths | Caribbean | 2010 | 3.024181883 | 4.068669005 | 2.22258319 |
| Deaths | Caribbean | 2011 | 3.031154579 | 4.075687391 | 2.230902176 |
| Deaths | Caribbean | 2012 | 3.078370044 | 4.08814289 | 2.281173498 |
| Deaths | Caribbean | 2013 | 3.167387064 | 4.202552994 | 2.346556356 |
| Deaths | Caribbean | 2014 | 3.248318269 | 4.323119673 | 2.391634192 |
| Deaths | Caribbean | 2015 | 3.315592085 | 4.399925971 | 2.44644888 |
| Deaths | Caribbean | 2016 | 3.367947702 | 4.476072747 | 2.483154594 |
| Deaths | Caribbean | 2017 | 3.406925832 | 4.573126858 | 2.479389587 |
| Deaths | Caribbean | 2018 | 3.432157943 | 4.617968611 | 2.467526529 |
| Deaths | Caribbean | 2019 | 3.429963988 | 4.68598832 | 2.435245692 |
| Deaths | Central Asia | 1990 | 2.081279993 | 2.811377221 | 1.477798099 |
| Deaths | Central Asia | 1991 | 2.207922367 | 2.974113339 | 1.576320485 |
| Deaths | Central Asia | 1992 | 2.365968439 | 3.170171558 | 1.693409594 |
| Deaths | Central Asia | 1993 | 2.581817629 | 3.463385285 | 1.857405469 |
| Deaths | Central Asia | 1994 | 2.781220672 | 3.716522929 | 2.023624015 |
| Deaths | Central Asia | 1995 | 2.892742549 | 3.841711898 | 2.106965825 |
| Deaths | Central Asia | 1996 | 2.986859367 | 3.959116219 | 2.172302349 |
| Deaths | Central Asia | 1997 | 3.039587323 | 4.022490193 | 2.224009588 |
| Deaths | Central Asia | 1998 | 3.087068631 | 4.093114201 | 2.262083744 |
| Deaths | Central Asia | 1999 | 3.136561113 | 4.154387168 | 2.289171373 |
| Deaths | Central Asia | 2000 | 3.249066596 | 4.298648198 | 2.394154706 |
| Deaths | Central Asia | 2001 | 3.349512257 | 4.428515 | 2.488512407 |
| Deaths | Central Asia | 2002 | 3.478648358 | 4.590784893 | 2.574234976 |
| Deaths | Central Asia | 2003 | 3.62091525 | 4.774349272 | 2.673078285 |
| Deaths | Central Asia | 2004 | 3.775579555 | 4.941624661 | 2.797519947 |
| Deaths | Central Asia | 2005 | 3.91456773 | 5.111688309 | 2.900936776 |
| Deaths | Central Asia | 2006 | 3.934035042 | 5.116406033 | 2.911695876 |
| Deaths | Central Asia | 2007 | 3.959570793 | 5.149757309 | 2.949472257 |
| Deaths | Central Asia | 2008 | 4.001556077 | 5.23002811 | 2.997540693 |
| Deaths | Central Asia | 2009 | 4.029041211 | 5.201951809 | 2.998850186 |
| Deaths | Central Asia | 2010 | 4.118152907 | 5.295593459 | 3.07214289 |
| Deaths | Central Asia | 2011 | 4.238665651 | 5.51164414 | 3.147811085 |
| Deaths | Central Asia | 2012 | 4.344651833 | 5.6290017 | 3.244289836 |
| Deaths | Central Asia | 2013 | 4.336214102 | 5.599796087 | 3.232988744 |
| Deaths | Central Asia | 2014 | 4.365134303 | 5.677878916 | 3.232832599 |
| Deaths | Central Asia | 2015 | 4.384570711 | 5.627825182 | 3.244586851 |
| Deaths | Central Asia | 2016 | 4.371578443 | 5.683352653 | 3.230922191 |
| Deaths | Central Asia | 2017 | 4.344913569 | 5.704964681 | 3.193142443 |
| Deaths | Central Asia | 2018 | 4.275606595 | 5.649656833 | 3.147986111 |
| Deaths | Central Asia | 2019 | 4.224719155 | 5.58311633 | 3.111703476 |
| Deaths | Central Europe | 1990 | 1.533973467 | 1.965837881 | 1.165292109 |
| Deaths | Central Europe | 1991 | 1.549800077 | 1.992142744 | 1.171135391 |
| Deaths | Central Europe | 1992 | 1.5795049 | 2.040443859 | 1.188222394 |
| Deaths | Central Europe | 1993 | 1.583479361 | 2.048748816 | 1.185833196 |
| Deaths | Central Europe | 1994 | 1.584396532 | 2.05598638 | 1.179670362 |
| Deaths | Central Europe | 1995 | 1.59250585 | 2.071243579 | 1.18011946 |
| Deaths | Central Europe | 1996 | 1.557842173 | 2.042104685 | 1.146666261 |
| Deaths | Central Europe | 1997 | 1.539033918 | 2.027519832 | 1.132199506 |
| Deaths | Central Europe | 1998 | 1.485228633 | 1.964374073 | 1.083884778 |
| Deaths | Central Europe | 1999 | 1.43585725 | 1.902666248 | 1.044305573 |
| Deaths | Central Europe | 2000 | 1.389509999 | 1.83888285 | 1.013413552 |
| Deaths | Central Europe | 2001 | 1.394510579 | 1.848595131 | 1.019871601 |
| Deaths | Central Europe | 2002 | 1.413192582 | 1.867258763 | 1.034606152 |
| Deaths | Central Europe | 2003 | 1.441495172 | 1.89715488 | 1.053366239 |
| Deaths | Central Europe | 2004 | 1.449202497 | 1.904646475 | 1.055760333 |
| Deaths | Central Europe | 2005 | 1.46818157 | 1.937992645 | 1.070479633 |
| Deaths | Central Europe | 2006 | 1.467112438 | 1.927953361 | 1.078534537 |
| Deaths | Central Europe | 2007 | 1.492250091 | 1.965923323 | 1.098432627 |
| Deaths | Central Europe | 2008 | 1.509981238 | 1.987484625 | 1.110546975 |
| Deaths | Central Europe | 2009 | 1.514172847 | 1.991922736 | 1.106750595 |
| Deaths | Central Europe | 2010 | 1.477368899 | 1.943810522 | 1.080696758 |
| Deaths | Central Europe | 2011 | 1.418989907 | 1.855957593 | 1.043482317 |
| Deaths | Central Europe | 2012 | 1.400207794 | 1.840699584 | 1.02934935 |
| Deaths | Central Europe | 2013 | 1.35401382 | 1.779685202 | 0.997757089 |
| Deaths | Central Europe | 2014 | 1.348103498 | 1.783221373 | 0.992938596 |
| Deaths | Central Europe | 2015 | 1.34929527 | 1.785605672 | 0.996808075 |
| Deaths | Central Europe | 2016 | 1.333590252 | 1.768468051 | 0.987882427 |
| Deaths | Central Europe | 2017 | 1.331905945 | 1.781232197 | 0.9927362 |
| Deaths | Central Europe | 2018 | 1.329359599 | 1.776914147 | 0.969205793 |
| Deaths | Central Europe | 2019 | 1.323022903 | 1.783077882 | 0.95516106 |
| Deaths | Central Latin America | 1990 | 6.96166154 | 8.808344588 | 5.276356464 |
| Deaths | Central Latin America | 1991 | 6.961019111 | 8.804895393 | 5.248421665 |
| Deaths | Central Latin America | 1992 | 6.997299936 | 8.869378192 | 5.293377479 |
| Deaths | Central Latin America | 1993 | 7.057347797 | 8.988866697 | 5.32809932 |
| Deaths | Central Latin America | 1994 | 7.053747752 | 8.998855205 | 5.330116177 |
| Deaths | Central Latin America | 1995 | 7.032462362 | 8.930659969 | 5.341182262 |
| Deaths | Central Latin America | 1996 | 6.934152805 | 8.797757539 | 5.264313405 |
| Deaths | Central Latin America | 1997 | 6.871886573 | 8.736929158 | 5.233342686 |
| Deaths | Central Latin America | 1998 | 6.834722906 | 8.703501136 | 5.180206395 |
| Deaths | Central Latin America | 1999 | 6.65903906 | 8.490218573 | 5.040301342 |
| Deaths | Central Latin America | 2000 | 6.517033991 | 8.302587899 | 4.944815734 |
| Deaths | Central Latin America | 2001 | 6.415216892 | 8.183049379 | 4.83811334 |
| Deaths | Central Latin America | 2002 | 6.46032995 | 8.222169128 | 4.868896727 |
| Deaths | Central Latin America | 2003 | 6.445087989 | 8.173821943 | 4.851943791 |
| Deaths | Central Latin America | 2004 | 6.289121761 | 7.988857593 | 4.742260248 |
| Deaths | Central Latin America | 2005 | 6.281655752 | 7.945430402 | 4.747317473 |
| Deaths | Central Latin America | 2006 | 6.19365719 | 7.836744275 | 4.671226003 |
| Deaths | Central Latin America | 2007 | 6.185914854 | 7.805140406 | 4.66379839 |
| Deaths | Central Latin America | 2008 | 6.397571984 | 8.051153201 | 4.820068638 |
| Deaths | Central Latin America | 2009 | 6.532009789 | 8.184738806 | 4.947978284 |
| Deaths | Central Latin America | 2010 | 6.370068566 | 7.977278923 | 4.843090253 |
| Deaths | Central Latin America | 2011 | 6.213835125 | 7.794005884 | 4.719069499 |
| Deaths | Central Latin America | 2012 | 6.144832954 | 7.692876718 | 4.661609459 |
| Deaths | Central Latin America | 2013 | 6.192228248 | 7.764697952 | 4.71171808 |
| Deaths | Central Latin America | 2014 | 6.12842193 | 7.690538281 | 4.633368703 |
| Deaths | Central Latin America | 2015 | 6.081312691 | 7.644845478 | 4.631699906 |
| Deaths | Central Latin America | 2016 | 6.111045022 | 7.688378106 | 4.656371906 |
| Deaths | Central Latin America | 2017 | 6.200955778 | 7.787557908 | 4.71157842 |
| Deaths | Central Latin America | 2018 | 6.205101143 | 7.893026465 | 4.704506346 |
| Deaths | Central Latin America | 2019 | 6.243274495 | 8.018097314 | 4.653528139 |
| Deaths | Central Sub-Saharan Africa | 1990 | 3.3239968 | 4.703699945 | 2.303649539 |
| Deaths | Central Sub-Saharan Africa | 1991 | 3.328197395 | 4.647819087 | 2.328285809 |
| Deaths | Central Sub-Saharan Africa | 1992 | 3.315269962 | 4.633050457 | 2.304595841 |
| Deaths | Central Sub-Saharan Africa | 1993 | 3.295818662 | 4.553998206 | 2.305256533 |
| Deaths | Central Sub-Saharan Africa | 1994 | 3.291977439 | 4.521243135 | 2.29336401 |
| Deaths | Central Sub-Saharan Africa | 1995 | 3.296906109 | 4.558725661 | 2.306955103 |
| Deaths | Central Sub-Saharan Africa | 1996 | 3.304161023 | 4.576319308 | 2.304189072 |
| Deaths | Central Sub-Saharan Africa | 1997 | 3.251762021 | 4.518403878 | 2.235529813 |
| Deaths | Central Sub-Saharan Africa | 1998 | 3.251960357 | 4.495363242 | 2.222788584 |
| Deaths | Central Sub-Saharan Africa | 1999 | 3.224577585 | 4.448716403 | 2.208767028 |
| Deaths | Central Sub-Saharan Africa | 2000 | 3.180437517 | 4.382792215 | 2.178338114 |
| Deaths | Central Sub-Saharan Africa | 2001 | 3.11679651 | 4.328320105 | 2.122892975 |
| Deaths | Central Sub-Saharan Africa | 2002 | 3.051629613 | 4.301027897 | 2.082263782 |
| Deaths | Central Sub-Saharan Africa | 2003 | 3.037484674 | 4.239773033 | 2.060875823 |
| Deaths | Central Sub-Saharan Africa | 2004 | 2.999416645 | 4.192408395 | 2.009075354 |
| Deaths | Central Sub-Saharan Africa | 2005 | 2.954224123 | 4.151952702 | 1.975873204 |
| Deaths | Central Sub-Saharan Africa | 2006 | 2.926899105 | 4.118140639 | 1.970253187 |
| Deaths | Central Sub-Saharan Africa | 2007 | 2.890687146 | 4.099687066 | 1.935573671 |
| Deaths | Central Sub-Saharan Africa | 2008 | 2.886732955 | 4.088351505 | 1.93193007 |
| Deaths | Central Sub-Saharan Africa | 2009 | 2.882801386 | 4.109352174 | 1.918805639 |
| Deaths | Central Sub-Saharan Africa | 2010 | 2.873980824 | 4.074650861 | 1.910198289 |
| Deaths | Central Sub-Saharan Africa | 2011 | 2.854572981 | 4.041930948 | 1.898061302 |
| Deaths | Central Sub-Saharan Africa | 2012 | 2.818504904 | 4.011093427 | 1.875950102 |
| Deaths | Central Sub-Saharan Africa | 2013 | 2.767480438 | 3.941321837 | 1.815547838 |
| Deaths | Central Sub-Saharan Africa | 2014 | 2.708439138 | 3.910094181 | 1.788033066 |
| Deaths | Central Sub-Saharan Africa | 2015 | 2.661580217 | 3.863261834 | 1.761531542 |
| Deaths | Central Sub-Saharan Africa | 2016 | 2.728683888 | 3.941162947 | 1.812053888 |
| Deaths | Central Sub-Saharan Africa | 2017 | 2.869698302 | 4.14675169 | 1.916264268 |
| Deaths | Central Sub-Saharan Africa | 2018 | 2.883360982 | 4.208704992 | 1.903020474 |
| Deaths | Central Sub-Saharan Africa | 2019 | 2.889636851 | 4.239646648 | 1.896835408 |
| Deaths | East Asia | 1990 | 2.528685458 | 3.087733117 | 2.004993705 |
| Deaths | East Asia | 1991 | 2.537566532 | 3.100455408 | 2.031248916 |
| Deaths | East Asia | 1992 | 2.523239957 | 3.129031396 | 2.016032823 |
| Deaths | East Asia | 1993 | 2.508765978 | 3.042865523 | 2.045294585 |
| Deaths | East Asia | 1994 | 2.477617978 | 2.996954499 | 2.018726608 |
| Deaths | East Asia | 1995 | 2.441917524 | 2.929579167 | 2.010158212 |
| Deaths | East Asia | 1996 | 2.406115719 | 2.89477132 | 1.987193413 |
| Deaths | East Asia | 1997 | 2.346982667 | 2.81675815 | 1.937864293 |
| Deaths | East Asia | 1998 | 2.296555024 | 2.74875421 | 1.887921694 |
| Deaths | East Asia | 1999 | 2.250743581 | 2.671322504 | 1.851513929 |
| Deaths | East Asia | 2000 | 2.172865607 | 2.638281395 | 1.785035093 |
| Deaths | East Asia | 2001 | 2.032185638 | 2.454551047 | 1.660153914 |
| Deaths | East Asia | 2002 | 1.875554681 | 2.290041223 | 1.518285974 |
| Deaths | East Asia | 2003 | 1.744232685 | 2.142139943 | 1.410276804 |
| Deaths | East Asia | 2004 | 1.647388382 | 2.054118362 | 1.31121983 |
| Deaths | East Asia | 2005 | 1.572800132 | 1.945413051 | 1.247624882 |
| Deaths | East Asia | 2006 | 1.49516387 | 1.868772206 | 1.196483443 |
| Deaths | East Asia | 2007 | 1.428197639 | 1.77119947 | 1.13340103 |
| Deaths | East Asia | 2008 | 1.386481186 | 1.716710506 | 1.10703414 |
| Deaths | East Asia | 2009 | 1.335526356 | 1.645080543 | 1.080536582 |
| Deaths | East Asia | 2010 | 1.289120877 | 1.598710183 | 1.027309674 |
| Deaths | East Asia | 2011 | 1.255795852 | 1.549527349 | 0.995172656 |
| Deaths | East Asia | 2012 | 1.246939603 | 1.529144369 | 0.98710245 |
| Deaths | East Asia | 2013 | 1.260680722 | 1.549606716 | 1.002218628 |
| Deaths | East Asia | 2014 | 1.288931395 | 1.603796056 | 1.02181581 |
| Deaths | East Asia | 2015 | 1.294803355 | 1.585198696 | 1.036055601 |
| Deaths | East Asia | 2016 | 1.295118136 | 1.619158075 | 1.029154054 |
| Deaths | East Asia | 2017 | 1.296391924 | 1.610572178 | 1.02812129 |
| Deaths | East Asia | 2018 | 1.284937147 | 1.598833118 | 1.001508109 |
| Deaths | East Asia | 2019 | 1.287097121 | 1.582959753 | 1.006368727 |
| Deaths | Eastern Europe | 1990 | 1.089219363 | 1.444471133 | 0.799575051 |
| Deaths | Eastern Europe | 1991 | 1.137142836 | 1.512638025 | 0.834012494 |
| Deaths | Eastern Europe | 1992 | 1.220010529 | 1.615344002 | 0.893407959 |
| Deaths | Eastern Europe | 1993 | 1.432079464 | 1.915830618 | 1.047972238 |
| Deaths | Eastern Europe | 1994 | 1.616938185 | 2.171071173 | 1.17541822 |
| Deaths | Eastern Europe | 1995 | 1.658285772 | 2.223248693 | 1.201916493 |
| Deaths | Eastern Europe | 1996 | 1.584409051 | 2.114088514 | 1.146003897 |
| Deaths | Eastern Europe | 1997 | 1.504300991 | 2.00542343 | 1.084415504 |
| Deaths | Eastern Europe | 1998 | 1.479255638 | 1.97542062 | 1.067040207 |
| Deaths | Eastern Europe | 1999 | 1.624507457 | 2.172078834 | 1.170458886 |
| Deaths | Eastern Europe | 2000 | 1.787113273 | 2.40374298 | 1.287826872 |
| Deaths | Eastern Europe | 2001 | 1.936194701 | 2.60937283 | 1.392033041 |
| Deaths | Eastern Europe | 2002 | 2.14553346 | 2.910475605 | 1.539925568 |
| Deaths | Eastern Europe | 2003 | 2.353279417 | 3.188926418 | 1.686378294 |
| Deaths | Eastern Europe | 2004 | 2.51531436 | 3.41147676 | 1.80228761 |
| Deaths | Eastern Europe | 2005 | 2.791443403 | 3.779475321 | 2.002188198 |
| Deaths | Eastern Europe | 2006 | 2.719020765 | 3.692894437 | 1.955815966 |
| Deaths | Eastern Europe | 2007 | 2.766800897 | 3.73936848 | 2.002309752 |
| Deaths | Eastern Europe | 2008 | 2.870688185 | 3.867388458 | 2.078309979 |
| Deaths | Eastern Europe | 2009 | 2.735089658 | 3.687071819 | 1.983774578 |
| Deaths | Eastern Europe | 2010 | 2.762546209 | 3.723847836 | 2.004577961 |
| Deaths | Eastern Europe | 2011 | 2.652469062 | 3.546038214 | 1.933627535 |
| Deaths | Eastern Europe | 2012 | 2.680875102 | 3.577506523 | 1.95993811 |
| Deaths | Eastern Europe | 2013 | 2.753297273 | 3.671712102 | 2.023617315 |
| Deaths | Eastern Europe | 2014 | 2.851689721 | 3.798722448 | 2.098137198 |
| Deaths | Eastern Europe | 2015 | 3.014188326 | 4.021343949 | 2.205230731 |
| Deaths | Eastern Europe | 2016 | 2.907937022 | 3.890065159 | 2.130550938 |
| Deaths | Eastern Europe | 2017 | 2.739485256 | 3.636048381 | 2.010897065 |
| Deaths | Eastern Europe | 2018 | 2.681789021 | 3.590398028 | 1.932438232 |
| Deaths | Eastern Europe | 2019 | 2.647009358 | 3.555501082 | 1.907230016 |
| Deaths | Eastern Sub-Saharan Africa | 1990 | 5.017387866 | 6.940367588 | 3.529003834 |
| Deaths | Eastern Sub-Saharan Africa | 1991 | 5.008234472 | 6.930700654 | 3.548943026 |
| Deaths | Eastern Sub-Saharan Africa | 1992 | 5.004237539 | 6.907114609 | 3.536751191 |
| Deaths | Eastern Sub-Saharan Africa | 1993 | 4.986261246 | 6.826433849 | 3.547637179 |
| Deaths | Eastern Sub-Saharan Africa | 1994 | 4.965573308 | 6.807134017 | 3.553620047 |
| Deaths | Eastern Sub-Saharan Africa | 1995 | 4.977887536 | 6.891581914 | 3.555248479 |
| Deaths | Eastern Sub-Saharan Africa | 1996 | 4.960969107 | 6.850679445 | 3.515340478 |
| Deaths | Eastern Sub-Saharan Africa | 1997 | 4.942646055 | 6.750523167 | 3.519691435 |
| Deaths | Eastern Sub-Saharan Africa | 1998 | 4.940203729 | 6.708354466 | 3.515515691 |
| Deaths | Eastern Sub-Saharan Africa | 1999 | 4.937141281 | 6.717564922 | 3.494975551 |
| Deaths | Eastern Sub-Saharan Africa | 2000 | 4.919411656 | 6.721023421 | 3.515257114 |
| Deaths | Eastern Sub-Saharan Africa | 2001 | 4.861711504 | 6.690456339 | 3.48418883 |
| Deaths | Eastern Sub-Saharan Africa | 2002 | 4.837822767 | 6.59753052 | 3.484708095 |
| Deaths | Eastern Sub-Saharan Africa | 2003 | 4.81821735 | 6.648425933 | 3.454675989 |
| Deaths | Eastern Sub-Saharan Africa | 2004 | 4.798996434 | 6.556522025 | 3.43350809 |
| Deaths | Eastern Sub-Saharan Africa | 2005 | 4.76626922 | 6.528526217 | 3.407977175 |
| Deaths | Eastern Sub-Saharan Africa | 2006 | 4.703157825 | 6.446417693 | 3.363992004 |
| Deaths | Eastern Sub-Saharan Africa | 2007 | 4.631652665 | 6.295579419 | 3.323924792 |
| Deaths | Eastern Sub-Saharan Africa | 2008 | 4.578584691 | 6.244545616 | 3.271534381 |
| Deaths | Eastern Sub-Saharan Africa | 2009 | 4.532902301 | 6.171262078 | 3.250147153 |
| Deaths | Eastern Sub-Saharan Africa | 2010 | 4.504669765 | 6.142885021 | 3.219023692 |
| Deaths | Eastern Sub-Saharan Africa | 2011 | 4.453917485 | 6.048579964 | 3.199449245 |
| Deaths | Eastern Sub-Saharan Africa | 2012 | 4.432294672 | 6.018009177 | 3.154279584 |
| Deaths | Eastern Sub-Saharan Africa | 2013 | 4.44105685 | 6.057861425 | 3.179468757 |
| Deaths | Eastern Sub-Saharan Africa | 2014 | 4.430399199 | 6.05924713 | 3.146702013 |
| Deaths | Eastern Sub-Saharan Africa | 2015 | 4.405380283 | 5.986452465 | 3.134481111 |
| Deaths | Eastern Sub-Saharan Africa | 2016 | 4.388191046 | 5.966555261 | 3.091505895 |
| Deaths | Eastern Sub-Saharan Africa | 2017 | 4.415408094 | 6.02688368 | 3.128843937 |
| Deaths | Eastern Sub-Saharan Africa | 2018 | 4.403295655 | 5.982607599 | 3.116681848 |
| Deaths | Eastern Sub-Saharan Africa | 2019 | 4.400543283 | 5.943937097 | 3.135193216 |
| Deaths | Global | 1990 | 2.394932462 | 3.050516968 | 1.839684075 |
| Deaths | Global | 1991 | 2.401078877 | 3.036186737 | 1.847968356 |
| Deaths | Global | 1992 | 2.408936818 | 3.053527367 | 1.860573535 |
| Deaths | Global | 1993 | 2.434035325 | 3.075028144 | 1.88539382 |
| Deaths | Global | 1994 | 2.445671579 | 3.078471588 | 1.885082119 |
| Deaths | Global | 1995 | 2.441083947 | 3.082075902 | 1.894581314 |
| Deaths | Global | 1996 | 2.422579008 | 3.043328285 | 1.876488995 |
| Deaths | Global | 1997 | 2.397044713 | 3.005959548 | 1.857757255 |
| Deaths | Global | 1998 | 2.371227674 | 2.965732267 | 1.854978247 |
| Deaths | Global | 1999 | 2.345522099 | 2.93051409 | 1.839040504 |
| Deaths | Global | 2000 | 2.313005963 | 2.88768694 | 1.791408097 |
| Deaths | Global | 2001 | 2.276110633 | 2.85383574 | 1.758494759 |
| Deaths | Global | 2002 | 2.250023199 | 2.825531032 | 1.743642329 |
| Deaths | Global | 2003 | 2.224990198 | 2.811776003 | 1.716337659 |
| Deaths | Global | 2004 | 2.185592447 | 2.755834432 | 1.685690257 |
| Deaths | Global | 2005 | 2.17438608 | 2.741718793 | 1.663056051 |
| Deaths | Global | 2006 | 2.14161623 | 2.689165174 | 1.653753273 |
| Deaths | Global | 2007 | 2.123831227 | 2.66293538 | 1.630521776 |
| Deaths | Global | 2008 | 2.127440054 | 2.675255567 | 1.632349709 |
| Deaths | Global | 2009 | 2.107425763 | 2.646997418 | 1.627618117 |
| Deaths | Global | 2010 | 2.088179105 | 2.624011334 | 1.613397482 |
| Deaths | Global | 2011 | 2.072012071 | 2.599078636 | 1.596380689 |
| Deaths | Global | 2012 | 2.072016905 | 2.59826921 | 1.61591868 |
| Deaths | Global | 2013 | 2.082699635 | 2.609568773 | 1.603818531 |
| Deaths | Global | 2014 | 2.0913015 | 2.627903495 | 1.612916706 |
| Deaths | Global | 2015 | 2.101607283 | 2.640942179 | 1.620884052 |
| Deaths | Global | 2016 | 2.102886684 | 2.642039818 | 1.621802725 |
| Deaths | Global | 2017 | 2.103031339 | 2.638750882 | 1.610200798 |
| Deaths | Global | 2018 | 2.093932275 | 2.634348239 | 1.610761295 |
| Deaths | Global | 2019 | 2.087758117 | 2.599749087 | 1.613922804 |
| Deaths | High-income Asia Pacific | 1990 | 1.17774846 | 1.420181275 | 0.94932067 |
| Deaths | High-income Asia Pacific | 1991 | 1.178659271 | 1.42155784 | 0.951321033 |
| Deaths | High-income Asia Pacific | 1992 | 1.185924733 | 1.430853856 | 0.962715983 |
| Deaths | High-income Asia Pacific | 1993 | 1.194754991 | 1.43782214 | 0.973652553 |
| Deaths | High-income Asia Pacific | 1994 | 1.199738389 | 1.443482177 | 0.980152325 |
| Deaths | High-income Asia Pacific | 1995 | 1.222646833 | 1.462935023 | 1.006222165 |
| Deaths | High-income Asia Pacific | 1996 | 1.227745702 | 1.454774557 | 1.015325202 |
| Deaths | High-income Asia Pacific | 1997 | 1.249250385 | 1.469064634 | 1.038160924 |
| Deaths | High-income Asia Pacific | 1998 | 1.294106683 | 1.514152662 | 1.080136154 |
| Deaths | High-income Asia Pacific | 1999 | 1.321539299 | 1.54403781 | 1.105098946 |
| Deaths | High-income Asia Pacific | 2000 | 1.309505773 | 1.524972267 | 1.092040775 |
| Deaths | High-income Asia Pacific | 2001 | 1.276599953 | 1.486681446 | 1.064704256 |
| Deaths | High-income Asia Pacific | 2002 | 1.23367353 | 1.435036819 | 1.031595971 |
| Deaths | High-income Asia Pacific | 2003 | 1.192714397 | 1.387930789 | 0.997678889 |
| Deaths | High-income Asia Pacific | 2004 | 1.14574344 | 1.332866712 | 0.953749864 |
| Deaths | High-income Asia Pacific | 2005 | 1.104243623 | 1.287339939 | 0.916290614 |
| Deaths | High-income Asia Pacific | 2006 | 1.064895293 | 1.243695146 | 0.882129781 |
| Deaths | High-income Asia Pacific | 2007 | 1.027444152 | 1.20692535 | 0.844962998 |
| Deaths | High-income Asia Pacific | 2008 | 0.992217312 | 1.168484727 | 0.813779725 |
| Deaths | High-income Asia Pacific | 2009 | 0.957830226 | 1.129161542 | 0.787449201 |
| Deaths | High-income Asia Pacific | 2010 | 0.934363839 | 1.102454073 | 0.767991685 |
| Deaths | High-income Asia Pacific | 2011 | 0.919305896 | 1.089270155 | 0.753508695 |
| Deaths | High-income Asia Pacific | 2012 | 0.904707257 | 1.073100321 | 0.737096991 |
| Deaths | High-income Asia Pacific | 2013 | 0.892169979 | 1.06352415 | 0.721208528 |
| Deaths | High-income Asia Pacific | 2014 | 0.878914586 | 1.052671171 | 0.707990209 |
| Deaths | High-income Asia Pacific | 2015 | 0.86129369 | 1.028493903 | 0.69167997 |
| Deaths | High-income Asia Pacific | 2016 | 0.838304489 | 1.003794985 | 0.67222485 |
| Deaths | High-income Asia Pacific | 2017 | 0.820793923 | 0.98376944 | 0.658500339 |
| Deaths | High-income Asia Pacific | 2018 | 0.82180475 | 0.983962355 | 0.66147476 |
| Deaths | High-income Asia Pacific | 2019 | 0.821626573 | 0.988874095 | 0.657976914 |
| Deaths | High-income North America | 1990 | 1.317285404 | 1.710665516 | 0.983122822 |
| Deaths | High-income North America | 1991 | 1.318832349 | 1.711667049 | 0.987306811 |
| Deaths | High-income North America | 1992 | 1.318619898 | 1.700916536 | 0.989067325 |
| Deaths | High-income North America | 1993 | 1.344411181 | 1.727137563 | 1.012245037 |
| Deaths | High-income North America | 1994 | 1.351913642 | 1.730515526 | 1.020919327 |
| Deaths | High-income North America | 1995 | 1.360091578 | 1.740389302 | 1.027415115 |
| Deaths | High-income North America | 1996 | 1.342737374 | 1.71376425 | 1.020844951 |
| Deaths | High-income North America | 1997 | 1.315863269 | 1.675504058 | 1.007779453 |
| Deaths | High-income North America | 1998 | 1.293792801 | 1.642243415 | 0.997147803 |
| Deaths | High-income North America | 1999 | 1.284826313 | 1.627205131 | 0.994696716 |
| Deaths | High-income North America | 2000 | 1.292613016 | 1.639189465 | 1.004062787 |
| Deaths | High-income North America | 2001 | 1.316912236 | 1.670310645 | 1.02072153 |
| Deaths | High-income North America | 2002 | 1.342988696 | 1.700737216 | 1.04090767 |
| Deaths | High-income North America | 2003 | 1.362634819 | 1.723636756 | 1.057385596 |
| Deaths | High-income North America | 2004 | 1.3619183 | 1.712616192 | 1.05674454 |
| Deaths | High-income North America | 2005 | 1.389082844 | 1.738263218 | 1.080024996 |
| Deaths | High-income North America | 2006 | 1.397240857 | 1.744111738 | 1.089305453 |
| Deaths | High-income North America | 2007 | 1.398370912 | 1.744851803 | 1.089750382 |
| Deaths | High-income North America | 2008 | 1.407011812 | 1.752089081 | 1.098256064 |
| Deaths | High-income North America | 2009 | 1.412801617 | 1.762407087 | 1.103107527 |
| Deaths | High-income North America | 2010 | 1.414216236 | 1.758829111 | 1.105895868 |
| Deaths | High-income North America | 2011 | 1.454444304 | 1.822423605 | 1.137078315 |
| Deaths | High-income North America | 2012 | 1.507642612 | 1.894536519 | 1.175949116 |
| Deaths | High-income North America | 2013 | 1.569799128 | 1.980708143 | 1.22130847 |
| Deaths | High-income North America | 2014 | 1.632756113 | 2.065740546 | 1.26921608 |
| Deaths | High-income North America | 2015 | 1.674474185 | 2.115406726 | 1.301617681 |
| Deaths | High-income North America | 2016 | 1.688980741 | 2.141273787 | 1.306943982 |
| Deaths | High-income North America | 2017 | 1.659150501 | 2.108788452 | 1.281994403 |
| Deaths | High-income North America | 2018 | 1.654249406 | 2.100528194 | 1.273628135 |
| Deaths | High-income North America | 2019 | 1.649382528 | 2.103654801 | 1.268195914 |
| Deaths | North Africa and Middle East | 1990 | 4.241330688 | 5.860453478 | 2.923206522 |
| Deaths | North Africa and Middle East | 1991 | 4.157494806 | 5.72030264 | 2.875736266 |
| Deaths | North Africa and Middle East | 1992 | 4.080060492 | 5.609474431 | 2.827976187 |
| Deaths | North Africa and Middle East | 1993 | 4.031959061 | 5.535534323 | 2.816290563 |
| Deaths | North Africa and Middle East | 1994 | 3.985499726 | 5.471159571 | 2.793967655 |
| Deaths | North Africa and Middle East | 1995 | 3.899936283 | 5.350282079 | 2.755844894 |
| Deaths | North Africa and Middle East | 1996 | 3.82710035 | 5.194216723 | 2.710721767 |
| Deaths | North Africa and Middle East | 1997 | 3.795487945 | 5.126959263 | 2.710693798 |
| Deaths | North Africa and Middle East | 1998 | 3.768387637 | 5.099558606 | 2.685247233 |
| Deaths | North Africa and Middle East | 1999 | 3.718924103 | 5.059075419 | 2.664248858 |
| Deaths | North Africa and Middle East | 2000 | 3.614397865 | 4.909293104 | 2.586819751 |
| Deaths | North Africa and Middle East | 2001 | 3.667762251 | 4.985793694 | 2.653345009 |
| Deaths | North Africa and Middle East | 2002 | 3.768919865 | 5.112369745 | 2.726228185 |
| Deaths | North Africa and Middle East | 2003 | 3.913406175 | 5.312803989 | 2.829487252 |
| Deaths | North Africa and Middle East | 2004 | 3.950216827 | 5.351587515 | 2.850504552 |
| Deaths | North Africa and Middle East | 2005 | 3.892802867 | 5.253438523 | 2.800771282 |
| Deaths | North Africa and Middle East | 2006 | 3.824983472 | 5.153247624 | 2.752264522 |
| Deaths | North Africa and Middle East | 2007 | 3.803773979 | 5.140758128 | 2.7364802 |
| Deaths | North Africa and Middle East | 2008 | 3.837620235 | 5.194412909 | 2.776962761 |
| Deaths | North Africa and Middle East | 2009 | 3.862761102 | 5.23135341 | 2.80850158 |
| Deaths | North Africa and Middle East | 2010 | 3.85847033 | 5.213524323 | 2.813798739 |
| Deaths | North Africa and Middle East | 2011 | 3.849754058 | 5.2074169 | 2.803638285 |
| Deaths | North Africa and Middle East | 2012 | 3.887269155 | 5.211593433 | 2.823431605 |
| Deaths | North Africa and Middle East | 2013 | 3.860777499 | 5.169244126 | 2.804088095 |
| Deaths | North Africa and Middle East | 2014 | 3.875917311 | 5.196208743 | 2.810198037 |
| Deaths | North Africa and Middle East | 2015 | 3.910389304 | 5.241868175 | 2.832556487 |
| Deaths | North Africa and Middle East | 2016 | 3.905168206 | 5.323243738 | 2.786198758 |
| Deaths | North Africa and Middle East | 2017 | 3.860352569 | 5.241669171 | 2.705974206 |
| Deaths | North Africa and Middle East | 2018 | 3.839717975 | 5.271785308 | 2.688778031 |
| Deaths | North Africa and Middle East | 2019 | 3.840503811 | 5.302899043 | 2.712724135 |
| Deaths | Oceania | 1990 | 1.509317798 | 2.047164587 | 1.113021824 |
| Deaths | Oceania | 1991 | 1.5098783 | 2.051707325 | 1.11154343 |
| Deaths | Oceania | 1992 | 1.504085957 | 2.044681465 | 1.103713998 |
| Deaths | Oceania | 1993 | 1.493410479 | 2.024073685 | 1.09827383 |
| Deaths | Oceania | 1994 | 1.48221652 | 1.9974436 | 1.084025766 |
| Deaths | Oceania | 1995 | 1.469264426 | 1.969290471 | 1.077776426 |
| Deaths | Oceania | 1996 | 1.470512765 | 1.964900967 | 1.08084566 |
| Deaths | Oceania | 1997 | 1.476371544 | 1.962127542 | 1.092360872 |
| Deaths | Oceania | 1998 | 1.490649523 | 1.994523945 | 1.102061429 |
| Deaths | Oceania | 1999 | 1.508666642 | 2.015292234 | 1.118707721 |
| Deaths | Oceania | 2000 | 1.500125758 | 2.000763238 | 1.111375172 |
| Deaths | Oceania | 2001 | 1.484475668 | 1.979257847 | 1.098784696 |
| Deaths | Oceania | 2002 | 1.475405227 | 1.963358185 | 1.088016862 |
| Deaths | Oceania | 2003 | 1.476709654 | 1.970147193 | 1.08387763 |
| Deaths | Oceania | 2004 | 1.46521139 | 1.963471218 | 1.075589828 |
| Deaths | Oceania | 2005 | 1.458253653 | 1.951089857 | 1.068937221 |
| Deaths | Oceania | 2006 | 1.466423832 | 1.942210934 | 1.0697124 |
| Deaths | Oceania | 2007 | 1.469205304 | 1.956985632 | 1.069485013 |
| Deaths | Oceania | 2008 | 1.464949816 | 1.962544384 | 1.061760673 |
| Deaths | Oceania | 2009 | 1.464307997 | 1.966957031 | 1.05542983 |
| Deaths | Oceania | 2010 | 1.471714087 | 1.966131803 | 1.067324063 |
| Deaths | Oceania | 2011 | 1.487012159 | 1.977937255 | 1.084233131 |
| Deaths | Oceania | 2012 | 1.488187632 | 1.96817558 | 1.088175214 |
| Deaths | Oceania | 2013 | 1.482130249 | 1.97077572 | 1.07583561 |
| Deaths | Oceania | 2014 | 1.478340581 | 1.962192819 | 1.074036191 |
| Deaths | Oceania | 2015 | 1.477127604 | 1.964982583 | 1.065335664 |
| Deaths | Oceania | 2016 | 1.469452358 | 1.958672621 | 1.0646195 |
| Deaths | Oceania | 2017 | 1.465937118 | 1.95403341 | 1.061823115 |
| Deaths | Oceania | 2018 | 1.458818819 | 1.957409001 | 1.040356947 |
| Deaths | Oceania | 2019 | 1.456489449 | 1.960807091 | 1.041887436 |
| Deaths | South Asia | 1990 | 2.009420891 | 2.617558445 | 1.507281631 |
| Deaths | South Asia | 1991 | 2.025131457 | 2.658895192 | 1.529781463 |
| Deaths | South Asia | 1992 | 2.038569803 | 2.654722513 | 1.539124076 |
| Deaths | South Asia | 1993 | 2.074152673 | 2.706227841 | 1.57514211 |
| Deaths | South Asia | 1994 | 2.099394699 | 2.71457262 | 1.583883898 |
| Deaths | South Asia | 1995 | 2.09731237 | 2.716473277 | 1.572885979 |
| Deaths | South Asia | 1996 | 2.104618096 | 2.721929631 | 1.584017538 |
| Deaths | South Asia | 1997 | 2.109503661 | 2.702853201 | 1.60871993 |
| Deaths | South Asia | 1998 | 2.03940935 | 2.604604866 | 1.551381916 |
| Deaths | South Asia | 1999 | 1.942018274 | 2.491844173 | 1.473828404 |
| Deaths | South Asia | 2000 | 1.890838733 | 2.408169145 | 1.435939819 |
| Deaths | South Asia | 2001 | 1.864157278 | 2.386425799 | 1.411248852 |
| Deaths | South Asia | 2002 | 1.822075398 | 2.32865921 | 1.371856028 |
| Deaths | South Asia | 2003 | 1.75084255 | 2.240605091 | 1.324184884 |
| Deaths | South Asia | 2004 | 1.672206928 | 2.150467016 | 1.256351653 |
| Deaths | South Asia | 2005 | 1.678611195 | 2.163643731 | 1.256965879 |
| Deaths | South Asia | 2006 | 1.685138009 | 2.159001923 | 1.273245124 |
| Deaths | South Asia | 2007 | 1.681705748 | 2.142502294 | 1.280351639 |
| Deaths | South Asia | 2008 | 1.677362014 | 2.136015639 | 1.282117145 |
| Deaths | South Asia | 2009 | 1.602729278 | 2.06393505 | 1.222070528 |
| Deaths | South Asia | 2010 | 1.567889598 | 2.00726562 | 1.195716606 |
| Deaths | South Asia | 2011 | 1.560216318 | 2.025412514 | 1.174690383 |
| Deaths | South Asia | 2012 | 1.541922088 | 1.97210419 | 1.17846479 |
| Deaths | South Asia | 2013 | 1.563490673 | 2.001499487 | 1.198868622 |
| Deaths | South Asia | 2014 | 1.555144875 | 1.967100597 | 1.193897184 |
| Deaths | South Asia | 2015 | 1.567702297 | 1.991468636 | 1.199085543 |
| Deaths | South Asia | 2016 | 1.588362321 | 2.012406512 | 1.203481687 |
| Deaths | South Asia | 2017 | 1.626664109 | 2.104057036 | 1.216939092 |
| Deaths | South Asia | 2018 | 1.626431342 | 2.110867915 | 1.230219717 |
| Deaths | South Asia | 2019 | 1.600159699 | 2.049901711 | 1.212113061 |
| Deaths | Southeast Asia | 1990 | 4.2491734 | 5.645757456 | 3.117694047 |
| Deaths | Southeast Asia | 1991 | 4.244772692 | 5.614765426 | 3.159836281 |
| Deaths | Southeast Asia | 1992 | 4.244686219 | 5.597756168 | 3.148389932 |
| Deaths | Southeast Asia | 1993 | 4.241483517 | 5.580169341 | 3.161429635 |
| Deaths | Southeast Asia | 1994 | 4.246083085 | 5.610033102 | 3.16970182 |
| Deaths | Southeast Asia | 1995 | 4.243012333 | 5.567610881 | 3.136755565 |
| Deaths | Southeast Asia | 1996 | 4.231914532 | 5.502819846 | 3.13776101 |
| Deaths | Southeast Asia | 1997 | 4.178185448 | 5.43778977 | 3.121985587 |
| Deaths | Southeast Asia | 1998 | 4.15448701 | 5.376392075 | 3.09195511 |
| Deaths | Southeast Asia | 1999 | 4.157582773 | 5.420692983 | 3.123011185 |
| Deaths | Southeast Asia | 2000 | 4.136369371 | 5.319983749 | 3.099104077 |
| Deaths | Southeast Asia | 2001 | 4.083212559 | 5.287829368 | 3.079151061 |
| Deaths | Southeast Asia | 2002 | 4.070822714 | 5.224931251 | 3.06699816 |
| Deaths | Southeast Asia | 2003 | 4.049122092 | 5.183278061 | 3.054497896 |
| Deaths | Southeast Asia | 2004 | 4.037539763 | 5.133529563 | 3.097489626 |
| Deaths | Southeast Asia | 2005 | 4.030764779 | 5.16777107 | 3.067308751 |
| Deaths | Southeast Asia | 2006 | 4.044229931 | 5.153884578 | 3.095284272 |
| Deaths | Southeast Asia | 2007 | 4.076871135 | 5.203849791 | 3.10351732 |
| Deaths | Southeast Asia | 2008 | 4.140983314 | 5.243234745 | 3.180441782 |
| Deaths | Southeast Asia | 2009 | 4.195740406 | 5.357648025 | 3.185294443 |
| Deaths | Southeast Asia | 2010 | 4.232387916 | 5.352187476 | 3.242774802 |
| Deaths | Southeast Asia | 2011 | 4.255536874 | 5.387566188 | 3.231803711 |
| Deaths | Southeast Asia | 2012 | 4.263226358 | 5.408469373 | 3.258442501 |
| Deaths | Southeast Asia | 2013 | 4.270042702 | 5.417757044 | 3.291616878 |
| Deaths | Southeast Asia | 2014 | 4.283190679 | 5.47075564 | 3.293462372 |
| Deaths | Southeast Asia | 2015 | 4.226336782 | 5.413096043 | 3.223981785 |
| Deaths | Southeast Asia | 2016 | 4.217179957 | 5.364786622 | 3.197388308 |
| Deaths | Southeast Asia | 2017 | 4.218439227 | 5.422344224 | 3.185447729 |
| Deaths | Southeast Asia | 2018 | 4.197630928 | 5.402386432 | 3.185715667 |
| Deaths | Southeast Asia | 2019 | 4.178370119 | 5.36584038 | 3.142281436 |
| Deaths | Southern Latin America | 1990 | 2.052288576 | 2.780479551 | 1.447747845 |
| Deaths | Southern Latin America | 1991 | 1.966357017 | 2.65493352 | 1.388371513 |
| Deaths | Southern Latin America | 1992 | 1.916172034 | 2.579089811 | 1.353231882 |
| Deaths | Southern Latin America | 1993 | 1.882286185 | 2.529753808 | 1.3370465 |
| Deaths | Southern Latin America | 1994 | 1.860234805 | 2.504297288 | 1.315758723 |
| Deaths | Southern Latin America | 1995 | 1.875116753 | 2.535627185 | 1.323644933 |
| Deaths | Southern Latin America | 1996 | 1.881008214 | 2.547197357 | 1.321094456 |
| Deaths | Southern Latin America | 1997 | 1.868081484 | 2.530384421 | 1.309117989 |
| Deaths | Southern Latin America | 1998 | 1.865648601 | 2.520424864 | 1.314905706 |
| Deaths | Southern Latin America | 1999 | 1.850943085 | 2.51128938 | 1.308392906 |
| Deaths | Southern Latin America | 2000 | 1.804853435 | 2.442060573 | 1.2843461 |
| Deaths | Southern Latin America | 2001 | 1.815104416 | 2.452209559 | 1.299493966 |
| Deaths | Southern Latin America | 2002 | 1.817814771 | 2.464493577 | 1.308078641 |
| Deaths | Southern Latin America | 2003 | 1.82872642 | 2.47160572 | 1.313800317 |
| Deaths | Southern Latin America | 2004 | 1.795970564 | 2.422785756 | 1.29088418 |
| Deaths | Southern Latin America | 2005 | 1.763954164 | 2.384368586 | 1.266881825 |
| Deaths | Southern Latin America | 2006 | 1.767969742 | 2.387258258 | 1.274710294 |
| Deaths | Southern Latin America | 2007 | 1.815777149 | 2.438840274 | 1.301553646 |
| Deaths | Southern Latin America | 2008 | 1.799945926 | 2.420055159 | 1.288575006 |
| Deaths | Southern Latin America | 2009 | 1.829998137 | 2.448556835 | 1.306179608 |
| Deaths | Southern Latin America | 2010 | 1.856265125 | 2.484437674 | 1.331253066 |
| Deaths | Southern Latin America | 2011 | 1.873668281 | 2.505812847 | 1.344434968 |
| Deaths | Southern Latin America | 2012 | 1.901456935 | 2.546500253 | 1.370612734 |
| Deaths | Southern Latin America | 2013 | 1.929528469 | 2.58887223 | 1.389436835 |
| Deaths | Southern Latin America | 2014 | 1.93707134 | 2.594237091 | 1.393683746 |
| Deaths | Southern Latin America | 2015 | 1.970387228 | 2.654044069 | 1.423666237 |
| Deaths | Southern Latin America | 2016 | 2.001945592 | 2.696257748 | 1.450859881 |
| Deaths | Southern Latin America | 2017 | 2.004256005 | 2.695332195 | 1.455873246 |
| Deaths | Southern Latin America | 2018 | 1.977505315 | 2.659427325 | 1.434681706 |
| Deaths | Southern Latin America | 2019 | 1.964812918 | 2.644973331 | 1.41876285 |
| Deaths | Southern Sub-Saharan Africa | 1990 | 2.506338593 | 3.413430564 | 1.720237376 |
| Deaths | Southern Sub-Saharan Africa | 1991 | 2.537218473 | 3.432895245 | 1.76594031 |
| Deaths | Southern Sub-Saharan Africa | 1992 | 2.651222583 | 3.543834131 | 1.876626748 |
| Deaths | Southern Sub-Saharan Africa | 1993 | 2.645686669 | 3.532261037 | 1.857043419 |
| Deaths | Southern Sub-Saharan Africa | 1994 | 2.779828655 | 3.660686471 | 1.998468976 |
| Deaths | Southern Sub-Saharan Africa | 1995 | 2.838234199 | 3.718067209 | 2.065911754 |
| Deaths | Southern Sub-Saharan Africa | 1996 | 2.984415177 | 3.823964161 | 2.221077279 |
| Deaths | Southern Sub-Saharan Africa | 1997 | 3.221508094 | 4.014326012 | 2.476348092 |
| Deaths | Southern Sub-Saharan Africa | 1998 | 3.296414732 | 4.085795654 | 2.566185481 |
| Deaths | Southern Sub-Saharan Africa | 1999 | 3.259322276 | 4.014095081 | 2.554761824 |
| Deaths | Southern Sub-Saharan Africa | 2000 | 3.294246998 | 4.039347474 | 2.619485394 |
| Deaths | Southern Sub-Saharan Africa | 2001 | 3.235397324 | 3.941577298 | 2.581909125 |
| Deaths | Southern Sub-Saharan Africa | 2002 | 3.217766069 | 3.922163619 | 2.572173442 |
| Deaths | Southern Sub-Saharan Africa | 2003 | 3.212348351 | 3.953455038 | 2.586032207 |
| Deaths | Southern Sub-Saharan Africa | 2004 | 3.180657809 | 3.908590128 | 2.549393087 |
| Deaths | Southern Sub-Saharan Africa | 2005 | 3.121886746 | 3.841939412 | 2.481508022 |
| Deaths | Southern Sub-Saharan Africa | 2006 | 3.117479628 | 3.844167557 | 2.485403088 |
| Deaths | Southern Sub-Saharan Africa | 2007 | 3.046148135 | 3.76772162 | 2.419835812 |
| Deaths | Southern Sub-Saharan Africa | 2008 | 3.024324692 | 3.758446205 | 2.390725657 |
| Deaths | Southern Sub-Saharan Africa | 2009 | 2.998412867 | 3.724770013 | 2.369285537 |
| Deaths | Southern Sub-Saharan Africa | 2010 | 2.944555597 | 3.684429754 | 2.334290891 |
| Deaths | Southern Sub-Saharan Africa | 2011 | 2.819464643 | 3.497950651 | 2.226301392 |
| Deaths | Southern Sub-Saharan Africa | 2012 | 2.734650212 | 3.403461506 | 2.168127336 |
| Deaths | Southern Sub-Saharan Africa | 2013 | 2.677711318 | 3.317575211 | 2.117089049 |
| Deaths | Southern Sub-Saharan Africa | 2014 | 2.666223258 | 3.321545763 | 2.128879733 |
| Deaths | Southern Sub-Saharan Africa | 2015 | 2.630741602 | 3.291438392 | 2.083581664 |
| Deaths | Southern Sub-Saharan Africa | 2016 | 2.57793003 | 3.243934371 | 2.041055801 |
| Deaths | Southern Sub-Saharan Africa | 2017 | 2.556497046 | 3.2062273 | 2.020488844 |
| Deaths | Southern Sub-Saharan Africa | 2018 | 2.460558561 | 3.060026613 | 1.943983158 |
| Deaths | Southern Sub-Saharan Africa | 2019 | 2.406085862 | 3.009544606 | 1.905563786 |
| Deaths | Tropical Latin America | 1990 | 2.253011277 | 2.963601343 | 1.694516169 |
| Deaths | Tropical Latin America | 1991 | 2.187382385 | 2.830457789 | 1.642482013 |
| Deaths | Tropical Latin America | 1992 | 2.163298889 | 2.832121553 | 1.624546062 |
| Deaths | Tropical Latin America | 1993 | 2.190939137 | 2.86327584 | 1.635872792 |
| Deaths | Tropical Latin America | 1994 | 2.151330822 | 2.792159748 | 1.60129805 |
| Deaths | Tropical Latin America | 1995 | 2.102392235 | 2.74035423 | 1.564175322 |
| Deaths | Tropical Latin America | 1996 | 2.044274521 | 2.648802698 | 1.527793209 |
| Deaths | Tropical Latin America | 1997 | 1.984473884 | 2.585044476 | 1.490262403 |
| Deaths | Tropical Latin America | 1998 | 1.964318377 | 2.548132262 | 1.473150545 |
| Deaths | Tropical Latin America | 1999 | 1.934244267 | 2.500161506 | 1.447447442 |
| Deaths | Tropical Latin America | 2000 | 1.901911949 | 2.479344888 | 1.430474888 |
| Deaths | Tropical Latin America | 2001 | 1.885314025 | 2.438207417 | 1.416055495 |
| Deaths | Tropical Latin America | 2002 | 1.876145764 | 2.436053271 | 1.412541478 |
| Deaths | Tropical Latin America | 2003 | 1.873147941 | 2.430189291 | 1.409283752 |
| Deaths | Tropical Latin America | 2004 | 1.870233801 | 2.425082739 | 1.410709459 |
| Deaths | Tropical Latin America | 2005 | 1.82523874 | 2.358623801 | 1.374359428 |
| Deaths | Tropical Latin America | 2006 | 1.832855739 | 2.375352641 | 1.381180182 |
| Deaths | Tropical Latin America | 2007 | 1.866011396 | 2.412212115 | 1.406009201 |
| Deaths | Tropical Latin America | 2008 | 1.915680357 | 2.481459488 | 1.447108766 |
| Deaths | Tropical Latin America | 2009 | 1.964159579 | 2.539543836 | 1.481846734 |
| Deaths | Tropical Latin America | 2010 | 1.991500458 | 2.57218194 | 1.506842941 |
| Deaths | Tropical Latin America | 2011 | 1.997293758 | 2.584953177 | 1.502188296 |
| Deaths | Tropical Latin America | 2012 | 1.974345232 | 2.565302507 | 1.498719828 |
| Deaths | Tropical Latin America | 2013 | 1.963571986 | 2.541853962 | 1.489684464 |
| Deaths | Tropical Latin America | 2014 | 1.942401958 | 2.496730872 | 1.464266643 |
| Deaths | Tropical Latin America | 2015 | 1.937767202 | 2.500134434 | 1.466696849 |
| Deaths | Tropical Latin America | 2016 | 2.014715413 | 2.600803784 | 1.524949416 |
| Deaths | Tropical Latin America | 2017 | 2.015798844 | 2.603081672 | 1.513591941 |
| Deaths | Tropical Latin America | 2018 | 2.005814279 | 2.572494503 | 1.511230759 |
| Deaths | Tropical Latin America | 2019 | 1.997310662 | 2.55878266 | 1.503457977 |
| Deaths | Western Europe | 1990 | 1.904360595 | 2.46407761 | 1.394096862 |
| Deaths | Western Europe | 1991 | 1.90542809 | 2.462969094 | 1.394546986 |
| Deaths | Western Europe | 1992 | 1.887859446 | 2.444819702 | 1.384237239 |
| Deaths | Western Europe | 1993 | 1.885299203 | 2.443236054 | 1.383579099 |
| Deaths | Western Europe | 1994 | 1.870094541 | 2.422453943 | 1.377699626 |
| Deaths | Western Europe | 1995 | 1.860031155 | 2.414420214 | 1.37359463 |
| Deaths | Western Europe | 1996 | 1.848581476 | 2.397513142 | 1.369113724 |
| Deaths | Western Europe | 1997 | 1.832207138 | 2.370042464 | 1.358599197 |
| Deaths | Western Europe | 1998 | 1.827179549 | 2.364852979 | 1.358228557 |
| Deaths | Western Europe | 1999 | 1.813814927 | 2.339697839 | 1.35414291 |
| Deaths | Western Europe | 2000 | 1.781619548 | 2.304859736 | 1.332935665 |
| Deaths | Western Europe | 2001 | 1.753974102 | 2.261525179 | 1.319840784 |
| Deaths | Western Europe | 2002 | 1.74185224 | 2.231093979 | 1.311839737 |
| Deaths | Western Europe | 2003 | 1.7267084 | 2.206358654 | 1.304957353 |
| Deaths | Western Europe | 2004 | 1.664552247 | 2.130414708 | 1.258197543 |
| Deaths | Western Europe | 2005 | 1.6313564 | 2.083285251 | 1.232889781 |
| Deaths | Western Europe | 2006 | 1.593546479 | 2.032369982 | 1.202877638 |
| Deaths | Western Europe | 2007 | 1.56916519 | 2.006523489 | 1.184179732 |
| Deaths | Western Europe | 2008 | 1.550031586 | 1.976751766 | 1.173514953 |
| Deaths | Western Europe | 2009 | 1.525712359 | 1.940763244 | 1.153887438 |
| Deaths | Western Europe | 2010 | 1.493715034 | 1.901289653 | 1.131456069 |
| Deaths | Western Europe | 2011 | 1.469390064 | 1.871287834 | 1.113927755 |
| Deaths | Western Europe | 2012 | 1.452632431 | 1.849789658 | 1.104156591 |
| Deaths | Western Europe | 2013 | 1.434027661 | 1.821862593 | 1.090475278 |
| Deaths | Western Europe | 2014 | 1.407181567 | 1.787603158 | 1.070888846 |
| Deaths | Western Europe | 2015 | 1.417491643 | 1.802165047 | 1.079727231 |
| Deaths | Western Europe | 2016 | 1.393623295 | 1.777985579 | 1.064578481 |
| Deaths | Western Europe | 2017 | 1.386204332 | 1.77181536 | 1.056017307 |
| Deaths | Western Europe | 2018 | 1.380115666 | 1.75685481 | 1.052275314 |
| Deaths | Western Europe | 2019 | 1.375359468 | 1.741443871 | 1.046635068 |
| Deaths | Western Sub-Saharan Africa | 1990 | 4.490877997 | 6.532930913 | 3.062049581 |
| Deaths | Western Sub-Saharan Africa | 1991 | 4.4853351 | 6.377939247 | 3.072706489 |
| Deaths | Western Sub-Saharan Africa | 1992 | 4.497228283 | 6.461798876 | 3.114061754 |
| Deaths | Western Sub-Saharan Africa | 1993 | 4.495801859 | 6.449889323 | 3.100960471 |
| Deaths | Western Sub-Saharan Africa | 1994 | 4.481907615 | 6.383659435 | 3.091055313 |
| Deaths | Western Sub-Saharan Africa | 1995 | 4.448863978 | 6.228947117 | 3.105067523 |
| Deaths | Western Sub-Saharan Africa | 1996 | 4.406448244 | 6.19322802 | 3.095881655 |
| Deaths | Western Sub-Saharan Africa | 1997 | 4.387319716 | 6.200528287 | 3.106292003 |
| Deaths | Western Sub-Saharan Africa | 1998 | 4.357209771 | 6.080950692 | 3.078711665 |
| Deaths | Western Sub-Saharan Africa | 1999 | 4.32784876 | 6.026195124 | 3.046752401 |
| Deaths | Western Sub-Saharan Africa | 2000 | 4.309360747 | 5.932117658 | 3.034262199 |
| Deaths | Western Sub-Saharan Africa | 2001 | 4.296314131 | 5.952438095 | 3.017579941 |
| Deaths | Western Sub-Saharan Africa | 2002 | 4.324431285 | 6.080041566 | 3.039337696 |
| Deaths | Western Sub-Saharan Africa | 2003 | 4.311426702 | 5.917819636 | 3.023282189 |
| Deaths | Western Sub-Saharan Africa | 2004 | 4.322592211 | 5.977875432 | 3.043123662 |
| Deaths | Western Sub-Saharan Africa | 2005 | 4.324436797 | 5.976275378 | 3.057232317 |
| Deaths | Western Sub-Saharan Africa | 2006 | 4.285088651 | 5.857262819 | 3.055864509 |
| Deaths | Western Sub-Saharan Africa | 2007 | 4.21973576 | 5.799535727 | 2.981558144 |
| Deaths | Western Sub-Saharan Africa | 2008 | 4.16476658 | 5.739667838 | 2.898253186 |
| Deaths | Western Sub-Saharan Africa | 2009 | 4.123330038 | 5.63159154 | 2.89780627 |
| Deaths | Western Sub-Saharan Africa | 2010 | 4.093771131 | 5.526712586 | 2.88565257 |
| Deaths | Western Sub-Saharan Africa | 2011 | 4.058834063 | 5.605348659 | 2.868816588 |
| Deaths | Western Sub-Saharan Africa | 2012 | 4.051668513 | 5.623148216 | 2.876242001 |
| Deaths | Western Sub-Saharan Africa | 2013 | 4.037849189 | 5.50827806 | 2.865256794 |
| Deaths | Western Sub-Saharan Africa | 2014 | 3.978952424 | 5.472846218 | 2.823213801 |
| Deaths | Western Sub-Saharan Africa | 2015 | 3.90670829 | 5.311544968 | 2.787936641 |
| Deaths | Western Sub-Saharan Africa | 2016 | 3.870897793 | 5.311514081 | 2.76734322 |
| Deaths | Western Sub-Saharan Africa | 2017 | 3.890007472 | 5.375908612 | 2.77058864 |
| Deaths | Western Sub-Saharan Africa | 2018 | 3.839906713 | 5.247412976 | 2.688196438 |
| Deaths | Western Sub-Saharan Africa | 2019 | 3.804205172 | 5.192757117 | 2.681047651 |

**Supplementary Table 5.** Global age-specific prevalence, incidence, DALYs, and Deaths rate, number of NAFLD in both sexes in 2019.

| **Measure** | **Age** | Rate | 95% Upper UI | 95% Lowe UI | **Number** | 95% Upper UI | 95% Lowe UI |
| --- | --- | --- | --- | --- | --- | --- | --- |
| Prevalence | 1-4 | 0 | 0 | 0 | 0 | 0 | 0 |
| Prevalence | 5-9 | 0 | 0 | 0 | 0 | 0 | 0 |
| Prevalence | 10-14 | 0 | 0 | 0 | 0 | 0 | 0 |
| Prevalence | 15-19 | 4706.954577 | 6916.286559 | 3142.073113 | 29161512.43 | 42849229.39 | 19466430.5 |
| Prevalence | 20-24 | 10199.99417 | 14400.82603 | 6973.26259 | 61214700.42 | 86425760.34 | 41849649.46 |
| Prevalence | 25-29 | 15421.33254 | 21193.24715 | 11237.26029 | 93371557.91 | 128318775.2 | 68038251.4 |
| Prevalence | 30-34 | 19893.94122 | 25941.54039 | 14627.4294 | 119708300.6 | 156098667.5 | 88017989.84 |
| Prevalence | 35-39 | 22622.83112 | 28937.64228 | 16361.44741 | 122384114.4 | 156545734.9 | 88511523.62 |
| Prevalence | 40-44 | 24118.00454 | 30439.30942 | 17657.72023 | 119008637.1 | 150200681.9 | 87130807.79 |
| Prevalence | 45-49 | 26800.15671 | 33832.01724 | 20183.15731 | 126979428 | 160296458.1 | 95628014.35 |
| Prevalence | 50-54 | 28856.36164 | 36332.56351 | 22548.02146 | 126049442.5 | 158706750 | 98493551.21 |
| Prevalence | 55-59 | 29655.71585 | 36962.99278 | 23255.36741 | 110026407.9 | 137137317.5 | 86280316.17 |
| Prevalence | 60-64 | 30390.88847 | 39071.58699 | 22182.22968 | 94982282.18 | 122112537.2 | 69327318.32 |
| Prevalence | 65-69 | 32079.12096 | 41315.7259 | 24594.54168 | 82951358.96 | 106835708.3 | 63597461.34 |
| Prevalence | 70-74 | 33457.35268 | 41871.14483 | 25498.5625 | 62594401.58 | 78335524 | 47704529.28 |
| Prevalence | 75-79 | 33580.8665 | 42132.29301 | 25535.56533 | 42665744.12 | 53530650.64 | 32443888.73 |
| Prevalence | 80-84 | 32279.6743 | 41140.31287 | 24767.37321 | 27251248.93 | 34731605.3 | 20909190.3 |
| Prevalence | 85-89 | 28083.81071 | 35721.50989 | 20884.31025 | 12211094.22 | 15532034.72 | 9080686.479 |
| Prevalence | 90-94 | 24094.71764 | 32266.97147 | 17351.28071 | 4061787.276 | 5439431.834 | 2925006.728 |
| Prevalence | 95+ | 22578.09576 | 32350.18386 | 15503.41041 | 1077699.595 | 1544141.739 | 740010.1098 |
| Incidence | 1-4 | 0 | 0 | 0 | 0 | 0 | 0 |
| Incidence | 5-9 | 0 | 0 | 0 | 0 | 0 | 0 |
| Incidence | 10-14 | 0 | 0 | 0 | 0 | 0 | 0 |
| Incidence | 15-19 | 0.213302795 | 0.477518484 | 0.084720113 | 1321.498224 | 2958.422685 | 524.875819 |
| Incidence | 20-24 | 0.508298436 | 1.068328141 | 0.205394527 | 3050.524931 | 6411.512203 | 1232.663887 |
| Incidence | 25-29 | 1.08828321 | 2.383299017 | 0.392948758 | 6589.229465 | 14430.163 | 2379.187245 |
| Incidence | 30-34 | 2.091687412 | 4.263588674 | 0.89485192 | 12586.36199 | 25655.39674 | 5384.614414 |
| Incidence | 35-39 | 3.335807141 | 6.372024063 | 1.354217145 | 18045.9201 | 34471.12864 | 7325.99139 |
| Incidence | 40-44 | 4.744325544 | 8.477983356 | 2.110586959 | 23410.54857 | 41834.02663 | 10414.5464 |
| Incidence | 45-49 | 5.796671875 | 11.32478626 | 2.406934368 | 27464.6931 | 53656.95792 | 11404.08068 |
| Incidence | 50-54 | 5.688190936 | 10.89280259 | 2.519945511 | 24846.975 | 47581.59433 | 11007.54595 |
| Incidence | 55-59 | 4.570211111 | 8.863048054 | 1.832147493 | 16956.05375 | 32883.0147 | 6797.495914 |
| Incidence | 60-64 | 3.498084609 | 7.151021015 | 1.571861603 | 10932.75242 | 22349.4715 | 4912.623811 |
| Incidence | 65-69 | 2.978355996 | 5.950964558 | 1.722998553 | 7701.541374 | 15388.22082 | 4455.392391 |
| Incidence | 70-74 | 3.298262643 | 5.150868718 | 2.223101299 | 6170.624984 | 9636.612558 | 4159.136461 |
| Incidence | 75-79 | 4.317302705 | 6.314115089 | 3.013484499 | 5485.294207 | 8022.318861 | 3828.744518 |
| Incidence | 80-84 | 5.147089452 | 7.328460799 | 3.541163345 | 4345.292167 | 6186.856398 | 2989.532141 |
| Incidence | 85-89 | 5.464827072 | 7.958747421 | 3.807147763 | 2376.156104 | 3460.535168 | 1655.382188 |
| Incidence | 90-94 | 4.545769993 | 7.470345637 | 2.785167007 | 766.3069971 | 1259.319794 | 469.511869 |
| Incidence | 95+ | 5.861805536 | 14.61307625 | 2.529776403 | 279.7962026 | 697.5126038 | 120.7515034 |
| DALYs | 1-4 | 0 | 0 | 0 | 0 | 0 | 0 |
| DALYs | 5-9 | 0 | 0 | 0 | 0 | 0 | 0 |
| DALYs | 10-14 | 0 | 0 | 0 | 0 | 0 | 0 |
| DALYs | 15-19 | 4.754668153 | 7.870406194 | 2.828765577 | 29457.11758 | 48760.39152 | 17525.36193 |
| DALYs | 20-24 | 10.40560593 | 16.69957252 | 6.081324612 | 62448.66804 | 100221.5601 | 36496.73305 |
| DALYs | 25-29 | 19.64959129 | 33.3695296 | 10.50039856 | 118972.4005 | 202042.525 | 63576.77392 |
| DALYs | 30-34 | 30.6060804 | 47.05132254 | 18.53963757 | 184166.7186 | 283123.0776 | 111559.0161 |
| DALYs | 35-39 | 43.59643334 | 72.19804715 | 23.69065358 | 235846.2942 | 390574.1953 | 128160.7789 |
| DALYs | 40-44 | 63.94687142 | 100.8410565 | 35.38621157 | 315541.4454 | 497593.2681 | 174610.8308 |
| DALYs | 45-49 | 87.56790246 | 143.0808129 | 48.36840894 | 414897.6547 | 677918.4157 | 229170.0368 |
| DALYs | 50-54 | 115.998126 | 186.8715002 | 65.52214728 | 506699.33 | 816286.152 | 286211.7628 |
| DALYs | 55-59 | 141.4079802 | 217.809337 | 84.82561579 | 524641.259 | 808099.8307 | 314713.6238 |
| DALYs | 60-64 | 166.6512088 | 262.8461021 | 100.9403079 | 520844.0074 | 821487.0934 | 315474.1861 |
| DALYs | 65-69 | 186.9396743 | 263.5031238 | 128.2059444 | 483395.4162 | 681375.9716 | 331519.5989 |
| DALYs | 70-74 | 209.2779681 | 283.5760166 | 147.6171103 | 391532.1484 | 530534.2366 | 276172.6179 |
| DALYs | 75-79 | 232.585634 | 326.8537601 | 154.3154025 | 295508.7281 | 415279.9002 | 196063.4779 |
| DALYs | 80-84 | 233.8369741 | 324.00016 | 156.943533 | 197410.5912 | 273528.4418 | 132495.3667 |
| DALYs | 85-89 | 217.6484555 | 298.9160381 | 149.7130631 | 94635.51169 | 129971.3897 | 65096.59028 |
| DALYs | 90-94 | 189.3476775 | 284.6793624 | 117.3826311 | 31919.44388 | 47990.06277 | 19787.87569 |
| DALYs | 95+ | 196.1236663 | 323.1475774 | 99.13702454 | 9361.391592 | 15424.50777 | 4732.016924 |
| Deaths | 1-4 | 0 | 0 | 0 | 403.8569048 | 674.9083686 | 235.4075161 |
| Deaths | 5-9 | 0 | 0 | 0 | 923.7606308 | 1491.914428 | 534.2275782 |
| Deaths | 10-14 | 0 | 0 | 0 | 1908.947171 | 3255.004959 | 1004.071293 |
| Deaths | 15-19 | 0.065186472 | 0.108936841 | 0.037997086 | 3217.77408 | 4967.740615 | 1934.214111 |
| Deaths | 20-24 | 0.153923044 | 0.24859255 | 0.089016497 | 4516.616533 | 7511.684627 | 2428.723592 |
| Deaths | 25-29 | 0.315283474 | 0.537599618 | 0.16583334 | 6677.004953 | 10579.26957 | 3676.685719 |
| Deaths | 30-34 | 0.534751626 | 0.825572991 | 0.321440883 | 9795.373733 | 16036.59839 | 5358.66062 |
| Deaths | 35-39 | 0.834901274 | 1.388542734 | 0.448952088 | 13486.12105 | 21876.50643 | 7542.02061 |
| Deaths | 40-44 | 1.353145786 | 2.143969361 | 0.745108297 | 15954.12877 | 24691.47058 | 9450.173112 |
| Deaths | 45-49 | 2.067402218 | 3.38466913 | 1.130993789 | 18441.37471 | 29197.09374 | 10951.57299 |
| Deaths | 50-54 | 3.087363011 | 5.008164802 | 1.726586568 | 20394.26594 | 28942.31881 | 13959.86891 |
| Deaths | 55-59 | 4.300159558 | 6.655158971 | 2.547130766 | 20204.67357 | 27470.71488 | 14230.37348 |
| Deaths | 60-64 | 5.900571658 | 9.342011997 | 3.504106511 | 19294.62325 | 27206.44954 | 12775.20465 |
| Deaths | 65-69 | 7.886912669 | 11.19263334 | 5.398589353 | 16783.73993 | 23347.39359 | 11265.82105 |
| Deaths | 70-74 | 10.79960623 | 14.68338019 | 7.606281269 | 10552.51317 | 14556.58586 | 7200.65717 |
| Deaths | 75-79 | 15.18619166 | 21.41334152 | 10.05496214 | 4609.564329 | 6966.741595 | 2839.42845 |
| Deaths | 80-84 | 19.88069098 | 27.65547601 | 13.34460065 | 1805.02359 | 2985.948719 | 905.8939548 |
| Deaths | 85-89 | 24.26930602 | 33.47811381 | 16.5605055 | 403.8569048 | 674.9083686 | 235.4075161 |
| Deaths | 90-94 | 27.34415748 | 41.32704648 | 16.84362624 | 923.7606308 | 1491.914428 | 534.2275782 |
| Deaths | 95+ | 37.81572865 | 62.55642704 | 18.97872148 | 1908.947171 | 3255.004959 | 1004.071293 |

**Supplementary Table 6.** Age- standardized prevalence, incidence, DALYs, and Death rate of NAFLD by SDI quintiles from 1990 to 2019.

| Measure | Location | Year | Rate | 95% Upper UI | 95% Lower UI |
| --- | --- | --- | --- | --- | --- |
| Prevalence | Global | 1990 | 12065.6273 | 13536.97376 | 10779.51408 |
| Prevalence | Global | 1991 | 12158.39245 | 13637.19148 | 10866.7718 |
| Prevalence | Global | 1992 | 12246.28598 | 13731.69949 | 10947.98828 |
| Prevalence | Global | 1993 | 12330.19867 | 13828.42504 | 11019.94637 |
| Prevalence | Global | 1994 | 12409.05801 | 13915.40885 | 11087.3694 |
| Prevalence | Global | 1995 | 12482.51645 | 13987.744 | 11149.88329 |
| Prevalence | Global | 1996 | 12559.2526 | 14077.32428 | 11224.9455 |
| Prevalence | Global | 1997 | 12638.61403 | 14169.88886 | 11302.97119 |
| Prevalence | Global | 1998 | 12715.04477 | 14259.28422 | 11370.86386 |
| Prevalence | Global | 1999 | 12782.14117 | 14337.36341 | 11435.78723 |
| Prevalence | Global | 2000 | 12833.12183 | 14393.43783 | 11486.00299 |
| Prevalence | Global | 2001 | 12852.35681 | 14401.06555 | 11510.75332 |
| Prevalence | Global | 2002 | 12839.98156 | 14371.51812 | 11507.57556 |
| Prevalence | Global | 2003 | 12820.5031 | 14335.15901 | 11497.6967 |
| Prevalence | Global | 2004 | 12817.17068 | 14317.97028 | 11501.73063 |
| Prevalence | Global | 2005 | 12853.55156 | 14352.05084 | 11540.89133 |
| Prevalence | Global | 2006 | 12959.83423 | 14469.21966 | 11633.85259 |
| Prevalence | Global | 2007 | 13128.07935 | 14655.04248 | 11783.54563 |
| Prevalence | Global | 2008 | 13328.89125 | 14879.81131 | 11964.39647 |
| Prevalence | Global | 2009 | 13531.98346 | 15107.71463 | 12151.46453 |
| Prevalence | Global | 2010 | 13708.48074 | 15302.11761 | 12313.79574 |
| Prevalence | Global | 2011 | 13884.32381 | 15497.45279 | 12477.70716 |
| Prevalence | Global | 2012 | 14085.54905 | 15718.91856 | 12651.47731 |
| Prevalence | Global | 2013 | 14292.69612 | 15948.04344 | 12823.56223 |
| Prevalence | Global | 2014 | 14486.07608 | 16165.27806 | 12996.04836 |
| Prevalence | Global | 2015 | 14645.75962 | 16339.74825 | 13138.76873 |
| Prevalence | Global | 2016 | 14843.5792 | 16559.33418 | 13316.7408 |
| Prevalence | Global | 2017 | 15015.96007 | 16749.73423 | 13464.14904 |
| Prevalence | Global | 2018 | 15062.42283 | 16797.84659 | 13527.19461 |
| Prevalence | Global | 2019 | 15023.47346 | 16764.84154 | 13493.72762 |
| Prevalence | High SDI | 1990 | 7642.630487 | 8614.165341 | 6801.621763 |
| Prevalence | High SDI | 1991 | 7707.008386 | 8682.488672 | 6863.203862 |
| Prevalence | High SDI | 1992 | 7773.389736 | 8756.082755 | 6926.955651 |
| Prevalence | High SDI | 1993 | 7841.249945 | 8824.17565 | 6993.346253 |
| Prevalence | High SDI | 1994 | 7909.051794 | 8891.122617 | 7060.061671 |
| Prevalence | High SDI | 1995 | 7977.183225 | 8958.58195 | 7127.771291 |
| Prevalence | High SDI | 1996 | 8049.458449 | 9024.689108 | 7194.323893 |
| Prevalence | High SDI | 1997 | 8127.208084 | 9097.490249 | 7272.602325 |
| Prevalence | High SDI | 1998 | 8207.700037 | 9178.081897 | 7348.992599 |
| Prevalence | High SDI | 1999 | 8288.389086 | 9262.841561 | 7421.174726 |
| Prevalence | High SDI | 2000 | 8367.518763 | 9348.273595 | 7494.146479 |
| Prevalence | High SDI | 2001 | 8453.844276 | 9446.817923 | 7574.901021 |
| Prevalence | High SDI | 2002 | 8554.115735 | 9559.542635 | 7669.028943 |
| Prevalence | High SDI | 2003 | 8663.269031 | 9682.764531 | 7771.714938 |
| Prevalence | High SDI | 2004 | 8775.546696 | 9808.727038 | 7871.831632 |
| Prevalence | High SDI | 2005 | 8885.482284 | 9931.495127 | 7968.479598 |
| Prevalence | High SDI | 2006 | 9009.383352 | 10067.30997 | 8075.428661 |
| Prevalence | High SDI | 2007 | 9159.325161 | 10232.48723 | 8211.548294 |
| Prevalence | High SDI | 2008 | 9326.448518 | 10417.50865 | 8365.961118 |
| Prevalence | High SDI | 2009 | 9486.976484 | 10595.18779 | 8505.656956 |
| Prevalence | High SDI | 2010 | 9618.268556 | 10732.85303 | 8624.214065 |
| Prevalence | High SDI | 2011 | 9752.40468 | 10870.43481 | 8745.425541 |
| Prevalence | High SDI | 2012 | 9900.034504 | 11034.29223 | 8875.310109 |
| Prevalence | High SDI | 2013 | 10046.7389 | 11196.55285 | 9009.868159 |
| Prevalence | High SDI | 2014 | 10177.64123 | 11341.33399 | 9121.135229 |
| Prevalence | High SDI | 2015 | 10279.01544 | 11452.13285 | 9204.555639 |
| Prevalence | High SDI | 2016 | 10340.51176 | 11527.08229 | 9258.171531 |
| Prevalence | High SDI | 2017 | 10391.13999 | 11585.52287 | 9301.99292 |
| Prevalence | High SDI | 2018 | 10458.9704 | 11655.2627 | 9356.794497 |
| Prevalence | High SDI | 2019 | 10529.77088 | 11725.32782 | 9427.493943 |
| Prevalence | High-middle SDI | 1990 | 12266.09067 | 13752.98234 | 10959.30149 |
| Prevalence | High-middle SDI | 1991 | 12378.25844 | 13876.8442 | 11059.14283 |
| Prevalence | High-middle SDI | 1992 | 12480.85983 | 13981.01705 | 11163.33752 |
| Prevalence | High-middle SDI | 1993 | 12574.28707 | 14067.7249 | 11255.5218 |
| Prevalence | High-middle SDI | 1994 | 12658.95864 | 14163.95943 | 11337.50876 |
| Prevalence | High-middle SDI | 1995 | 12735.19039 | 14262.06801 | 11411.19067 |
| Prevalence | High-middle SDI | 1996 | 12808.34549 | 14332.87707 | 11483.9354 |
| Prevalence | High-middle SDI | 1997 | 12876.2367 | 14396.73786 | 11544.54886 |
| Prevalence | High-middle SDI | 1998 | 12935.00691 | 14449.17735 | 11596.23841 |
| Prevalence | High-middle SDI | 1999 | 12982.13294 | 14488.57284 | 11653.42486 |
| Prevalence | High-middle SDI | 2000 | 13015.31593 | 14521.78316 | 11689.41881 |
| Prevalence | High-middle SDI | 2001 | 13010.83906 | 14517.35702 | 11693.90934 |
| Prevalence | High-middle SDI | 2002 | 12962.77243 | 14463.16248 | 11647.36112 |
| Prevalence | High-middle SDI | 2003 | 12900.9447 | 14398.21718 | 11593.61701 |
| Prevalence | High-middle SDI | 2004 | 12857.3431 | 14351.49408 | 11564.32565 |
| Prevalence | High-middle SDI | 2005 | 12865.58943 | 14349.57353 | 11570.02211 |
| Prevalence | High-middle SDI | 2006 | 12966.06885 | 14453.1175 | 11661.17145 |
| Prevalence | High-middle SDI | 2007 | 13151.6574 | 14651.10629 | 11828.10133 |
| Prevalence | High-middle SDI | 2008 | 13384.20207 | 14901.72254 | 12038.34836 |
| Prevalence | High-middle SDI | 2009 | 13625.1135 | 15163.24023 | 12251.02968 |
| Prevalence | High-middle SDI | 2010 | 13837.27043 | 15395.21748 | 12445.39498 |
| Prevalence | High-middle SDI | 2011 | 14044.89994 | 15645.61182 | 12625.55419 |
| Prevalence | High-middle SDI | 2012 | 14281.19596 | 15900.48687 | 12835.92618 |
| Prevalence | High-middle SDI | 2013 | 14523.37925 | 16175.94207 | 13056.52215 |
| Prevalence | High-middle SDI | 2014 | 14752.47096 | 16418.3354 | 13266.02803 |
| Prevalence | High-middle SDI | 2015 | 14951.4152 | 16626.98767 | 13438.41351 |
| Prevalence | High-middle SDI | 2016 | 15223.64851 | 16921.39604 | 13668.44393 |
| Prevalence | High-middle SDI | 2017 | 15457.22941 | 17195.17134 | 13884.4141 |
| Prevalence | High-middle SDI | 2018 | 15476.27849 | 17218.94881 | 13906.85941 |
| Prevalence | High-middle SDI | 2019 | 15336.60084 | 17095.37348 | 13799.25332 |
| Prevalence | Low SDI | 1990 | 12871.7655 | 14519.97905 | 11418.30802 |
| Prevalence | Low SDI | 1991 | 12905.38102 | 14558.13993 | 11450.416 |
| Prevalence | Low SDI | 1992 | 12951.20866 | 14610.78238 | 11496.87596 |
| Prevalence | Low SDI | 1993 | 12994.64749 | 14652.7136 | 11540.98409 |
| Prevalence | Low SDI | 1994 | 13027.24975 | 14690.74235 | 11576.47556 |
| Prevalence | Low SDI | 1995 | 13055.62167 | 14730.21837 | 11613.55011 |
| Prevalence | Low SDI | 1996 | 13090.28694 | 14771.62054 | 11645.48559 |
| Prevalence | Low SDI | 1997 | 13137.65079 | 14821.68347 | 11688.08425 |
| Prevalence | Low SDI | 1998 | 13188.9619 | 14875.95716 | 11733.44457 |
| Prevalence | Low SDI | 1999 | 13236.35348 | 14925.50493 | 11775.04789 |
| Prevalence | Low SDI | 2000 | 13274.58062 | 14965.53173 | 11808.33639 |
| Prevalence | Low SDI | 2001 | 13301.09036 | 14991.6832 | 11832.31512 |
| Prevalence | Low SDI | 2002 | 13319.68641 | 15013.21031 | 11850.63486 |
| Prevalence | Low SDI | 2003 | 13332.2882 | 15016.79558 | 11864.62162 |
| Prevalence | Low SDI | 2004 | 13346.78623 | 15025.3515 | 11879.75883 |
| Prevalence | Low SDI | 2005 | 13370.00353 | 15046.84048 | 11902.68999 |
| Prevalence | Low SDI | 2006 | 13400.77632 | 15085.27833 | 11935.78103 |
| Prevalence | Low SDI | 2007 | 13437.26235 | 15130.21906 | 11967.71684 |
| Prevalence | Low SDI | 2008 | 13477.3831 | 15177.51641 | 12001.27489 |
| Prevalence | Low SDI | 2009 | 13519.02509 | 15231.28162 | 12043.14255 |
| Prevalence | Low SDI | 2010 | 13562.13177 | 15287.45211 | 12077.52274 |
| Prevalence | Low SDI | 2011 | 13630.40977 | 15352.83814 | 12147.41801 |
| Prevalence | Low SDI | 2012 | 13734.19847 | 15460.54396 | 12250.68781 |
| Prevalence | Low SDI | 2013 | 13850.94117 | 15583.24929 | 12344.83899 |
| Prevalence | Low SDI | 2014 | 13956.9093 | 15694.97582 | 12444.0581 |
| Prevalence | Low SDI | 2015 | 14030.71753 | 15772.03044 | 12517.48277 |
| Prevalence | Low SDI | 2016 | 14096.33506 | 15831.32652 | 12579.82676 |
| Prevalence | Low SDI | 2017 | 14157.86769 | 15885.73065 | 12665.32842 |
| Prevalence | Low SDI | 2018 | 14212.90061 | 15983.43781 | 12703.05557 |
| Prevalence | Low SDI | 2019 | 14278.87191 | 16053.59562 | 12742.25978 |
| Prevalence | Low-middle SDI | 1990 | 12935.7097 | 14688.07903 | 11517.83119 |
| Prevalence | Low-middle SDI | 1991 | 12984.38464 | 14729.85567 | 11565.43316 |
| Prevalence | Low-middle SDI | 1992 | 13034.07043 | 14768.82065 | 11615.00604 |
| Prevalence | Low-middle SDI | 1993 | 13083.24521 | 14818.30394 | 11664.53752 |
| Prevalence | Low-middle SDI | 1994 | 13130.18869 | 14860.75948 | 11709.67796 |
| Prevalence | Low-middle SDI | 1995 | 13174.19113 | 14899.73422 | 11747.6748 |
| Prevalence | Low-middle SDI | 1996 | 13227.08903 | 14951.13299 | 11799.53315 |
| Prevalence | Low-middle SDI | 1997 | 13293.86502 | 15018.59315 | 11861.21541 |
| Prevalence | Low-middle SDI | 1998 | 13362.41943 | 15088.53111 | 11922.78159 |
| Prevalence | Low-middle SDI | 1999 | 13420.73343 | 15147.64844 | 11975.22492 |
| Prevalence | Low-middle SDI | 2000 | 13457.81233 | 15183.45159 | 12009.00882 |
| Prevalence | Low-middle SDI | 2001 | 13451.67215 | 15180.65651 | 11999.27101 |
| Prevalence | Low-middle SDI | 2002 | 13407.25444 | 15133.28884 | 11963.17684 |
| Prevalence | Low-middle SDI | 2003 | 13352.10863 | 15076.7827 | 11919.10305 |
| Prevalence | Low-middle SDI | 2004 | 13313.88377 | 15039.21394 | 11888.73745 |
| Prevalence | Low-middle SDI | 2005 | 13320.68451 | 15049.06675 | 11897.72885 |
| Prevalence | Low-middle SDI | 2006 | 13382.44335 | 15113.69325 | 11960.78747 |
| Prevalence | Low-middle SDI | 2007 | 13481.01309 | 15219.03271 | 12057.0985 |
| Prevalence | Low-middle SDI | 2008 | 13601.25135 | 15349.9857 | 12176.33402 |
| Prevalence | Low-middle SDI | 2009 | 13728.82991 | 15484.3132 | 12301.12483 |
| Prevalence | Low-middle SDI | 2010 | 13850.10851 | 15611.23497 | 12410.69918 |
| Prevalence | Low-middle SDI | 2011 | 14015.94887 | 15783.54964 | 12563.30452 |
| Prevalence | Low-middle SDI | 2012 | 14252.535 | 16040.71498 | 12764.23549 |
| Prevalence | Low-middle SDI | 2013 | 14510.82719 | 16323.58995 | 13002.18681 |
| Prevalence | Low-middle SDI | 2014 | 14743.02714 | 16573.04072 | 13225.06163 |
| Prevalence | Low-middle SDI | 2015 | 14901.8418 | 16731.13809 | 13371.33635 |
| Prevalence | Low-middle SDI | 2016 | 15029.84826 | 16874.7935 | 13475.88761 |
| Prevalence | Low-middle SDI | 2017 | 15137.25241 | 17018.11686 | 13579.0118 |
| Prevalence | Low-middle SDI | 2018 | 15196.75834 | 17046.42006 | 13618.59396 |
| Prevalence | Low-middle SDI | 2019 | 15233.1151 | 17078.82098 | 13662.82653 |
| Prevalence | Middle SDI | 1990 | 14627.60151 | 16372.33661 | 13111.88368 |
| Prevalence | Middle SDI | 1991 | 14722.29044 | 16473.45594 | 13201.48442 |
| Prevalence | Middle SDI | 1992 | 14808.80534 | 16558.22569 | 13270.80713 |
| Prevalence | Middle SDI | 1993 | 14888.97113 | 16639.16542 | 13329.10215 |
| Prevalence | Middle SDI | 1994 | 14962.72694 | 16717.43655 | 13403.52799 |
| Prevalence | Middle SDI | 1995 | 15031.51292 | 16785.62037 | 13487.08241 |
| Prevalence | Middle SDI | 1996 | 15101.63167 | 16863.84709 | 13541.9108 |
| Prevalence | Middle SDI | 1997 | 15175.17007 | 16949.31439 | 13598.20308 |
| Prevalence | Middle SDI | 1998 | 15242.58021 | 17034.81804 | 13648.22339 |
| Prevalence | Middle SDI | 1999 | 15294.70629 | 17102.77807 | 13699.71354 |
| Prevalence | Middle SDI | 2000 | 15324.06814 | 17144.40756 | 13730.72068 |
| Prevalence | Middle SDI | 2001 | 15299.23436 | 17107.8601 | 13721.88457 |
| Prevalence | Middle SDI | 2002 | 15224.94523 | 17015.66183 | 13668.61682 |
| Prevalence | Middle SDI | 2003 | 15140.90805 | 16910.75005 | 13605.84033 |
| Prevalence | Middle SDI | 2004 | 15084.36574 | 16834.62002 | 13569.69276 |
| Prevalence | Middle SDI | 2005 | 15094.14373 | 16831.134 | 13587.36314 |
| Prevalence | Middle SDI | 2006 | 15215.45598 | 16958.92192 | 13705.4455 |
| Prevalence | Middle SDI | 2007 | 15436.3556 | 17198.57452 | 13903.09287 |
| Prevalence | Middle SDI | 2008 | 15706.47656 | 17480.74419 | 14148.79068 |
| Prevalence | Middle SDI | 2009 | 15977.8215 | 17770.0333 | 14395.67273 |
| Prevalence | Middle SDI | 2010 | 16204.25602 | 18011.93851 | 14601.19285 |
| Prevalence | Middle SDI | 2011 | 16396.62604 | 18252.62008 | 14774.55888 |
| Prevalence | Middle SDI | 2012 | 16593.21272 | 18463.78727 | 14930.63903 |
| Prevalence | Middle SDI | 2013 | 16788.79684 | 18680.32779 | 15111.48225 |
| Prevalence | Middle SDI | 2014 | 16977.90326 | 18888.35335 | 15291.21187 |
| Prevalence | Middle SDI | 2015 | 17152.7061 | 19072.22607 | 15447.3699 |
| Prevalence | Middle SDI | 2016 | 17431.35222 | 19353.81548 | 15702.4102 |
| Prevalence | Middle SDI | 2017 | 17678.37687 | 19642.40309 | 15926.34053 |
| Prevalence | Middle SDI | 2018 | 17709.17564 | 19663.25833 | 15964.54505 |
| Prevalence | Middle SDI | 2019 | 17597.06289 | 19525.98019 | 15842.78146 |
| Incidence | Global | 1990 | 1.938241845 | 2.768330014 | 1.381595027 |
| Incidence | Global | 1991 | 1.946826337 | 2.76413417 | 1.402345728 |
| Incidence | Global | 1992 | 1.954296585 | 2.759298296 | 1.417642945 |
| Incidence | Global | 1993 | 1.960650421 | 2.755199413 | 1.436025788 |
| Incidence | Global | 1994 | 1.965975223 | 2.747268195 | 1.444182614 |
| Incidence | Global | 1995 | 1.970645338 | 2.752189829 | 1.455459926 |
| Incidence | Global | 1996 | 1.97358118 | 2.744677755 | 1.464643566 |
| Incidence | Global | 1997 | 1.973694161 | 2.733281206 | 1.470605092 |
| Incidence | Global | 1998 | 1.971278492 | 2.719559282 | 1.470143445 |
| Incidence | Global | 1999 | 1.966389655 | 2.713191152 | 1.467267472 |
| Incidence | Global | 2000 | 1.959080737 | 2.709819702 | 1.461313578 |
| Incidence | Global | 2001 | 1.942772133 | 2.693601161 | 1.45052228 |
| Incidence | Global | 2002 | 1.914628652 | 2.659808839 | 1.426498741 |
| Incidence | Global | 2003 | 1.883911318 | 2.616313897 | 1.397905276 |
| Incidence | Global | 2004 | 1.859380005 | 2.57998069 | 1.372776529 |
| Incidence | Global | 2005 | 1.849516465 | 2.562897228 | 1.361835085 |
| Incidence | Global | 2006 | 1.853227532 | 2.567510519 | 1.371065617 |
| Incidence | Global | 2007 | 1.861693669 | 2.577253491 | 1.383209073 |
| Incidence | Global | 2008 | 1.872974618 | 2.589504507 | 1.395131761 |
| Incidence | Global | 2009 | 1.88556702 | 2.603555614 | 1.40467187 |
| Incidence | Global | 2010 | 1.898134353 | 2.619265658 | 1.413404264 |
| Incidence | Global | 2011 | 1.913598422 | 2.638398156 | 1.423690695 |
| Incidence | Global | 2012 | 1.934823153 | 2.666340106 | 1.43892565 |
| Incidence | Global | 2013 | 1.958735621 | 2.698461059 | 1.457136779 |
| Incidence | Global | 2014 | 1.982623632 | 2.731039504 | 1.473894197 |
| Incidence | Global | 2015 | 2.003649807 | 2.762990029 | 1.483321523 |
| Incidence | Global | 2016 | 2.034196779 | 2.834227898 | 1.491107017 |
| Incidence | Global | 2017 | 2.061923251 | 2.903263935 | 1.509801649 |
| Incidence | Global | 2018 | 2.073072619 | 2.907385611 | 1.514556393 |
| Incidence | Global | 2019 | 2.07942373 | 2.925991658 | 1.517317984 |
| Incidence | High SDI | 1990 | 1.869575184 | 2.746698347 | 1.295368591 |
| Incidence | High SDI | 1991 | 1.911184577 | 2.779500245 | 1.333516462 |
| Incidence | High SDI | 1992 | 1.9513784 | 2.835912686 | 1.376543524 |
| Incidence | High SDI | 1993 | 1.986978479 | 2.869857962 | 1.4047851 |
| Incidence | High SDI | 1994 | 2.016960158 | 2.917299907 | 1.430324126 |
| Incidence | High SDI | 1995 | 2.039776484 | 2.944282982 | 1.448417482 |
| Incidence | High SDI | 1996 | 2.059216066 | 2.965794232 | 1.466952843 |
| Incidence | High SDI | 1997 | 2.079482918 | 2.9860219 | 1.490058995 |
| Incidence | High SDI | 1998 | 2.097552704 | 2.997422027 | 1.512674318 |
| Incidence | High SDI | 1999 | 2.112376216 | 2.996923357 | 1.527612844 |
| Incidence | High SDI | 2000 | 2.121520377 | 2.994332649 | 1.5404821 |
| Incidence | High SDI | 2001 | 2.124754929 | 3.002191818 | 1.541246395 |
| Incidence | High SDI | 2002 | 2.124080277 | 3.003204302 | 1.540849172 |
| Incidence | High SDI | 2003 | 2.121103404 | 3.001257456 | 1.53943476 |
| Incidence | High SDI | 2004 | 2.11717032 | 2.987825257 | 1.539775309 |
| Incidence | High SDI | 2005 | 2.113763427 | 2.975605986 | 1.536167848 |
| Incidence | High SDI | 2006 | 2.109387091 | 2.967888801 | 1.52994149 |
| Incidence | High SDI | 2007 | 2.10320002 | 2.955077466 | 1.52192846 |
| Incidence | High SDI | 2008 | 2.096374684 | 2.941066596 | 1.517350979 |
| Incidence | High SDI | 2009 | 2.091360528 | 2.931622402 | 1.513391071 |
| Incidence | High SDI | 2010 | 2.089690397 | 2.92537167 | 1.510234349 |
| Incidence | High SDI | 2011 | 2.09227933 | 2.927203989 | 1.519488487 |
| Incidence | High SDI | 2012 | 2.099767408 | 2.928569376 | 1.530471131 |
| Incidence | High SDI | 2013 | 2.109816409 | 2.94507269 | 1.536550593 |
| Incidence | High SDI | 2014 | 2.120544275 | 2.970838249 | 1.542905903 |
| Incidence | High SDI | 2015 | 2.129950914 | 2.988751122 | 1.544587154 |
| Incidence | High SDI | 2016 | 2.149452779 | 3.025096197 | 1.563638233 |
| Incidence | High SDI | 2017 | 2.168877993 | 3.052025032 | 1.582112513 |
| Incidence | High SDI | 2018 | 2.170027029 | 3.057121434 | 1.578160355 |
| Incidence | High SDI | 2019 | 2.161758558 | 3.056302664 | 1.579832038 |
| Incidence | High-middle SDI | 1990 | 1990 | 2.077958666 | 2.943945408 |
| Incidence | High-middle SDI | 1991 | 1991 | 2.069517584 | 2.918615877 |
| Incidence | High-middle SDI | 1992 | 1992 | 2.06107694 | 2.899555367 |
| Incidence | High-middle SDI | 1993 | 1993 | 2.054660596 | 2.884937907 |
| Incidence | High-middle SDI | 1994 | 1994 | 2.049886446 | 2.877915511 |
| Incidence | High-middle SDI | 1995 | 1995 | 2.048126393 | 2.865610571 |
| Incidence | High-middle SDI | 1996 | 1996 | 2.047723106 | 2.864395123 |
| Incidence | High-middle SDI | 1997 | 1997 | 2.048334489 | 2.877461945 |
| Incidence | High-middle SDI | 1998 | 1998 | 2.047326468 | 2.886518355 |
| Incidence | High-middle SDI | 1999 | 1999 | 2.042247395 | 2.874057632 |
| Incidence | High-middle SDI | 2000 | 2000 | 2.031459278 | 2.856535972 |
| Incidence | High-middle SDI | 2001 | 2001 | 2.003572635 | 2.822246342 |
| Incidence | High-middle SDI | 2002 | 2002 | 1.955516571 | 2.763211705 |
| Incidence | High-middle SDI | 2003 | 2003 | 1.901152011 | 2.701716395 |
| Incidence | High-middle SDI | 2004 | 2004 | 1.855722262 | 2.654133683 |
| Incidence | High-middle SDI | 2005 | 2005 | 1.833790097 | 2.636518733 |
| Incidence | High-middle SDI | 2006 | 2006 | 1.832359735 | 2.632351261 |
| Incidence | High-middle SDI | 2007 | 2007 | 1.835204623 | 2.644882302 |
| Incidence | High-middle SDI | 2008 | 2008 | 1.841671888 | 2.650967014 |
| Incidence | High-middle SDI | 2009 | 2009 | 1.8512518 | 2.667317378 |
| Incidence | High-middle SDI | 2010 | 2010 | 1.862555143 | 2.684267225 |
| Incidence | High-middle SDI | 2011 | 2011 | 1.876772708 | 2.711421049 |
| Incidence | High-middle SDI | 2012 | 2012 | 1.894321651 | 2.74217228 |
| Incidence | High-middle SDI | 2013 | 2013 | 1.91384766 | 2.776380321 |
| Incidence | High-middle SDI | 2014 | 2014 | 1.93363261 | 2.811650703 |
| Incidence | High-middle SDI | 2015 | 2015 | 1.951714724 | 2.842807707 |
| Incidence | High-middle SDI | 2016 | 2016 | 1.973874133 | 2.87655776 |
| Incidence | High-middle SDI | 2017 | 2017 | 1.990476873 | 2.901535412 |
| Incidence | High-middle SDI | 2018 | 2018 | 1.992813792 | 2.918057215 |
| Incidence | High-middle SDI | 2019 | 2019 | 1.98933414 | 2.923393605 |
| Incidence | Low SDI | 1990 | 1.283387351 | 1.892583222 | 0.85453391 |
| Incidence | Low SDI | 1991 | 1.28293797 | 1.896242918 | 0.867875447 |
| Incidence | Low SDI | 1992 | 1.281502809 | 1.877722139 | 0.878790375 |
| Incidence | Low SDI | 1993 | 1.27894966 | 1.864076713 | 0.881527668 |
| Incidence | Low SDI | 1994 | 1.277885323 | 1.863267128 | 0.890616197 |
| Incidence | Low SDI | 1995 | 1.278366225 | 1.857009357 | 0.89320923 |
| Incidence | Low SDI | 1996 | 1.278130176 | 1.851352261 | 0.897127396 |
| Incidence | Low SDI | 1997 | 1.275699397 | 1.842962921 | 0.898122996 |
| Incidence | Low SDI | 1998 | 1.272030021 | 1.837468913 | 0.896934021 |
| Incidence | Low SDI | 1999 | 1.268836586 | 1.831989158 | 0.895033353 |
| Incidence | Low SDI | 2000 | 1.267207966 | 1.829478184 | 0.895372029 |
| Incidence | Low SDI | 2001 | 1.267543942 | 1.826662453 | 0.897626564 |
| Incidence | Low SDI | 2002 | 1.269998962 | 1.826379529 | 0.901480831 |
| Incidence | Low SDI | 2003 | 1.273689888 | 1.830041539 | 0.905644007 |
| Incidence | Low SDI | 2004 | 1.277423659 | 1.827701146 | 0.909214584 |
| Incidence | Low SDI | 2005 | 1.279805439 | 1.832299198 | 0.911509319 |
| Incidence | Low SDI | 2006 | 1.28240864 | 1.835843411 | 0.913749299 |
| Incidence | Low SDI | 2007 | 1.285982671 | 1.839455122 | 0.914202053 |
| Incidence | Low SDI | 2008 | 1.2903547 | 1.841194152 | 0.918596356 |
| Incidence | Low SDI | 2009 | 1.295067261 | 1.85002027 | 0.925360432 |
| Incidence | Low SDI | 2010 | 1.299309468 | 1.858147166 | 0.9282617 |
| Incidence | Low SDI | 2011 | 1.30695009 | 1.866767082 | 0.932715881 |
| Incidence | Low SDI | 2012 | 1.319065866 | 1.883156723 | 0.941180912 |
| Incidence | Low SDI | 2013 | 1.333793636 | 1.905791095 | 0.949270605 |
| Incidence | Low SDI | 2014 | 1.34948075 | 1.931042586 | 0.959173993 |
| Incidence | Low SDI | 2015 | 1.364656727 | 1.954057745 | 0.971578076 |
| Incidence | Low SDI | 2016 | 1.39914615 | 2.025875559 | 0.982299888 |
| Incidence | Low SDI | 2017 | 1.435673825 | 2.10309288 | 0.993948063 |
| Incidence | Low SDI | 2018 | 1.458174394 | 2.138951977 | 1.007940692 |
| Incidence | Low SDI | 2019 | 1.48291259 | 2.171471877 | 1.027064885 |
| Incidence | Low-middle SDI | 1990 | 1.400277538 | 2.004391961 | 0.994099179 |
| Incidence | Low-middle SDI | 1991 | 1.407405515 | 2.005592075 | 1.008660375 |
| Incidence | Low-middle SDI | 1992 | 1.412484847 | 2.004878513 | 1.021266277 |
| Incidence | Low-middle SDI | 1993 | 1.415856151 | 2.006309888 | 1.025957488 |
| Incidence | Low-middle SDI | 1994 | 1.418253408 | 2.015620263 | 1.025955315 |
| Incidence | Low-middle SDI | 1995 | 1.41991166 | 2.023208923 | 1.029607296 |
| Incidence | Low-middle SDI | 1996 | 1.416408828 | 2.012946279 | 1.029572744 |
| Incidence | Low-middle SDI | 1997 | 1.405755775 | 1.994679146 | 1.024718792 |
| Incidence | Low-middle SDI | 1998 | 1.39165138 | 1.974414589 | 1.023171006 |
| Incidence | Low-middle SDI | 1999 | 1.377366588 | 1.953582144 | 1.017015888 |
| Incidence | Low-middle SDI | 2000 | 1.366350657 | 1.938977676 | 1.010919203 |
| Incidence | Low-middle SDI | 2001 | 1.354584728 | 1.924803799 | 1.000268566 |
| Incidence | Low-middle SDI | 2002 | 1.338871846 | 1.904813753 | 0.986671116 |
| Incidence | Low-middle SDI | 2003 | 1.323402532 | 1.884685579 | 0.971974173 |
| Incidence | Low-middle SDI | 2004 | 1.312172354 | 1.871018654 | 0.962983465 |
| Incidence | Low-middle SDI | 2005 | 1.309393853 | 1.869740815 | 0.960679551 |
| Incidence | Low-middle SDI | 2006 | 1.315219678 | 1.878414924 | 0.96925458 |
| Incidence | Low-middle SDI | 2007 | 1.32597285 | 1.888739856 | 0.97364972 |
| Incidence | Low-middle SDI | 2008 | 1.340072753 | 1.898360034 | 0.985046781 |
| Incidence | Low-middle SDI | 2009 | 1.355814435 | 1.911640081 | 0.993833064 |
| Incidence | Low-middle SDI | 2010 | 1.371659699 | 1.951924442 | 1.00769345 |
| Incidence | Low-middle SDI | 2011 | 1.393669612 | 1.973723723 | 1.024175387 |
| Incidence | Low-middle SDI | 2012 | 1.425060093 | 2.006515764 | 1.047663958 |
| Incidence | Low-middle SDI | 2013 | 1.461056112 | 2.057899002 | 1.073440295 |
| Incidence | Low-middle SDI | 2014 | 1.49682738 | 2.113403483 | 1.10067758 |
| Incidence | Low-middle SDI | 2015 | 1.527704142 | 2.161104879 | 1.121116837 |
| Incidence | Low-middle SDI | 2016 | 1.571745115 | 2.227052606 | 1.142667053 |
| Incidence | Low-middle SDI | 2017 | 1.6126394 | 2.307815501 | 1.154265837 |
| Incidence | Low-middle SDI | 2018 | 1.635237036 | 2.346978711 | 1.179467721 |
| Incidence | Low-middle SDI | 2019 | 1.655863703 | 2.385688362 | 1.188461704 |
| Incidence | Middle SDI | 1990 | 2.494519407 | 3.441091298 | 1.796266593 |
| Incidence | Middle SDI | 1991 | 2.492404211 | 3.423668491 | 1.820835252 |
| Incidence | Middle SDI | 1992 | 2.488906851 | 3.417740674 | 1.827538858 |
| Incidence | Middle SDI | 1993 | 2.484642384 | 3.41162091 | 1.829311037 |
| Incidence | Middle SDI | 1994 | 2.479987823 | 3.406854167 | 1.835060933 |
| Incidence | Middle SDI | 1995 | 2.476380243 | 3.382391906 | 1.838355247 |
| Incidence | Middle SDI | 1996 | 2.470387463 | 3.369454266 | 1.837679354 |
| Incidence | Middle SDI | 1997 | 2.459278371 | 3.3577452 | 1.836295585 |
| Incidence | Middle SDI | 1998 | 2.444555924 | 3.342102353 | 1.83309356 |
| Incidence | Middle SDI | 1999 | 2.427619727 | 3.325156448 | 1.823659613 |
| Incidence | Middle SDI | 2000 | 2.411033675 | 3.304203879 | 1.80146965 |
| Incidence | Middle SDI | 2001 | 2.380973095 | 3.266699746 | 1.767808842 |
| Incidence | Middle SDI | 2002 | 2.333536374 | 3.217482519 | 1.724528538 |
| Incidence | Middle SDI | 2003 | 2.283518013 | 3.16357629 | 1.676295111 |
| Incidence | Middle SDI | 2004 | 2.245619426 | 3.120755939 | 1.639787407 |
| Incidence | Middle SDI | 2005 | 2.234956415 | 3.110512128 | 1.632285193 |
| Incidence | Middle SDI | 2006 | 2.247214181 | 3.119830113 | 1.651798326 |
| Incidence | Middle SDI | 2007 | 2.268311228 | 3.15276476 | 1.673708417 |
| Incidence | Middle SDI | 2008 | 2.293355768 | 3.187097092 | 1.699002536 |
| Incidence | Middle SDI | 2009 | 2.318722963 | 3.216952012 | 1.723268321 |
| Incidence | Middle SDI | 2010 | 2.342077281 | 3.243043151 | 1.743360432 |
| Incidence | Middle SDI | 2011 | 2.366049386 | 3.276725839 | 1.756875489 |
| Incidence | Middle SDI | 2012 | 2.39589404 | 3.311143611 | 1.781257775 |
| Incidence | Middle SDI | 2013 | 2.428176567 | 3.353545406 | 1.80741232 |
| Incidence | Middle SDI | 2014 | 2.460125547 | 3.406210753 | 1.828044651 |
| Incidence | Middle SDI | 2015 | 2.48828639 | 3.446036372 | 1.846624633 |
| Incidence | Middle SDI | 2016 | 2.525979554 | 3.509639554 | 1.858736469 |
| Incidence | Middle SDI | 2017 | 2.558987445 | 3.553674872 | 1.88385643 |
| Incidence | Middle SDI | 2018 | 2.571995064 | 3.546944255 | 1.887539249 |
| Incidence | Middle SDI | 2019 | 2.578783951 | 3.581303708 | 1.899914942 |
| DALYs | Global | 1990 | 63.27695445 | 80.86191797 | 48.57578191 |
| DALYs | Global | 1991 | 63.40766764 | 80.47155246 | 48.73902102 |
| DALYs | Global | 1992 | 63.58901241 | 81.75094599 | 49.13441042 |
| DALYs | Global | 1993 | 64.21842754 | 81.96901359 | 49.47041377 |
| DALYs | Global | 1994 | 64.6269623 | 82.50147667 | 49.54493763 |
| DALYs | Global | 1995 | 64.44968144 | 81.91798756 | 50.07739315 |
| DALYs | Global | 1996 | 63.78457133 | 81.84482645 | 49.33681803 |
| DALYs | Global | 1997 | 62.89076152 | 80.18000974 | 48.55102172 |
| DALYs | Global | 1998 | 61.93091515 | 77.99970675 | 47.47336722 |
| DALYs | Global | 1999 | 61.08056302 | 77.06894748 | 47.49754847 |
| DALYs | Global | 2000 | 60.11542991 | 76.18292113 | 46.55468782 |
| DALYs | Global | 2001 | 58.99578891 | 74.87176602 | 45.82030848 |
| DALYs | Global | 2002 | 58.07382269 | 73.46443967 | 44.54984111 |
| DALYs | Global | 2003 | 57.24940926 | 72.59929773 | 43.65692518 |
| DALYs | Global | 2004 | 56.21810773 | 71.92393595 | 42.89886855 |
| DALYs | Global | 2005 | 55.97703892 | 71.54830512 | 42.63506383 |
| DALYs | Global | 2006 | 55.06090214 | 70.05009927 | 41.95297328 |
| DALYs | Global | 2007 | 54.56538511 | 69.45399536 | 41.46565428 |
| DALYs | Global | 2008 | 54.59242021 | 69.78404001 | 41.67176881 |
| DALYs | Global | 2009 | 53.93084502 | 68.84170285 | 41.30511097 |
| DALYs | Global | 2010 | 53.35614147 | 67.90144433 | 41.0423737 |
| DALYs | Global | 2011 | 52.82459074 | 67.22502064 | 40.27504018 |
| DALYs | Global | 2012 | 52.72877206 | 67.36748394 | 40.31195159 |
| DALYs | Global | 2013 | 52.96092066 | 67.39469881 | 40.52737199 |
| DALYs | Global | 2014 | 53.13555531 | 67.6987777 | 40.51264884 |
| DALYs | Global | 2015 | 53.45247784 | 67.97524216 | 41.21105647 |
| DALYs | Global | 2016 | 53.60313636 | 68.26292694 | 41.12070754 |
| DALYs | Global | 2017 | 53.77686116 | 68.72818884 | 40.88091026 |
| DALYs | Global | 2018 | 53.55825682 | 68.27580765 | 41.08285641 |
| DALYs | Global | 2019 | 53.3267928 | 68.28922947 | 40.7299943 |
| DALYs | High SDI | 1990 | 37.26166612 | 48.86630439 | 27.74158041 |
| DALYs | High SDI | 1991 | 37.35135444 | 48.93575759 | 27.8576006 |
| DALYs | High SDI | 1992 | 37.28540476 | 48.76060253 | 27.8659971 |
| DALYs | High SDI | 1993 | 37.61158874 | 49.05508497 | 28.2795135 |
| DALYs | High SDI | 1994 | 37.66213231 | 49.08312955 | 28.38322171 |
| DALYs | High SDI | 1995 | 37.88026516 | 49.1842821 | 28.62559711 |
| DALYs | High SDI | 1996 | 37.67117673 | 48.74991832 | 28.57938069 |
| DALYs | High SDI | 1997 | 37.36339363 | 48.24645147 | 28.48282117 |
| DALYs | High SDI | 1998 | 37.39557515 | 48.23869739 | 28.74109801 |
| DALYs | High SDI | 1999 | 37.35331834 | 48.0015658 | 28.7643446 |
| DALYs | High SDI | 2000 | 37.26600677 | 47.77913554 | 28.71260132 |
| DALYs | High SDI | 2001 | 37.2111423 | 47.76364814 | 28.61174635 |
| DALYs | High SDI | 2002 | 37.04830357 | 47.72711084 | 28.43112762 |
| DALYs | High SDI | 2003 | 36.97600268 | 47.65552278 | 28.45207406 |
| DALYs | High SDI | 2004 | 36.25332761 | 46.6775885 | 27.86477599 |
| DALYs | High SDI | 2005 | 36.03332936 | 46.39422059 | 27.7283851 |
| DALYs | High SDI | 2006 | 35.56601929 | 45.79703436 | 27.35354607 |
| DALYs | High SDI | 2007 | 35.1325344 | 45.1096972 | 27.00981635 |
| DALYs | High SDI | 2008 | 34.77610239 | 44.65766146 | 26.75616132 |
| DALYs | High SDI | 2009 | 34.35245962 | 44.28293508 | 26.47377439 |
| DALYs | High SDI | 2010 | 33.84082937 | 43.539221 | 26.01943888 |
| DALYs | High SDI | 2011 | 33.8215167 | 43.58214927 | 26.00500064 |
| DALYs | High SDI | 2012 | 33.88227892 | 43.60323373 | 26.13966957 |
| DALYs | High SDI | 2013 | 34.22302264 | 43.95555273 | 26.27595989 |
| DALYs | High SDI | 2014 | 34.48525355 | 44.36970024 | 26.51228368 |
| DALYs | High SDI | 2015 | 34.79435797 | 44.90453273 | 26.81405055 |
| DALYs | High SDI | 2016 | 34.80943301 | 45.13895602 | 26.78349379 |
| DALYs | High SDI | 2017 | 34.50724964 | 44.67841926 | 26.36156957 |
| DALYs | High SDI | 2018 | 34.33909506 | 44.68954351 | 26.20776052 |
| DALYs | High SDI | 2019 | 34.15800397 | 44.20998003 | 26.14064621 |
| DALYs | High-middle SDI | 1990 | 52.08574673 | 65.58268768 | 40.1094805 |
| DALYs | High-middle SDI | 1991 | 52.35001955 | 66.23536058 | 40.69401288 |
| DALYs | High-middle SDI | 1992 | 52.7788586 | 67.82310605 | 41.00433197 |
| DALYs | High-middle SDI | 1993 | 54.23218626 | 69.13648966 | 42.19581737 |
| DALYs | High-middle SDI | 1994 | 55.38374835 | 70.31361653 | 42.7974059 |
| DALYs | High-middle SDI | 1995 | 55.30742448 | 70.09692241 | 43.14115647 |
| DALYs | High-middle SDI | 1996 | 54.06518183 | 69.43715137 | 41.98048874 |
| DALYs | High-middle SDI | 1997 | 52.7688581 | 66.65406441 | 41.05070924 |
| DALYs | High-middle SDI | 1998 | 51.76958512 | 65.26890842 | 40.50553711 |
| DALYs | High-middle SDI | 1999 | 51.98022287 | 66.02254156 | 40.75169948 |
| DALYs | High-middle SDI | 2000 | 51.75845092 | 65.36810509 | 40.29293466 |
| DALYs | High-middle SDI | 2001 | 51.03567796 | 64.92947602 | 39.53531925 |
| DALYs | High-middle SDI | 2002 | 50.65774469 | 64.84547057 | 38.98434336 |
| DALYs | High-middle SDI | 2003 | 50.47525496 | 65.32229124 | 38.28380847 |
| DALYs | High-middle SDI | 2004 | 50.07039212 | 65.08573503 | 37.80008241 |
| DALYs | High-middle SDI | 2005 | 50.74017485 | 66.17040616 | 38.50623304 |
| DALYs | High-middle SDI | 2006 | 49.11929188 | 64.18122361 | 37.26539502 |
| DALYs | High-middle SDI | 2007 | 48.63153629 | 63.03292704 | 36.86823776 |
| DALYs | High-middle SDI | 2008 | 48.67100853 | 63.2067036 | 37.18064192 |
| DALYs | High-middle SDI | 2009 | 46.83978483 | 60.40654844 | 35.73447802 |
| DALYs | High-middle SDI | 2010 | 45.90578389 | 59.40826756 | 35.17180132 |
| DALYs | High-middle SDI | 2011 | 44.32830718 | 57.30512976 | 33.73191759 |
| DALYs | High-middle SDI | 2012 | 43.81686487 | 56.38350238 | 33.26218463 |
| DALYs | High-middle SDI | 2013 | 43.69044051 | 56.4332092 | 33.12754007 |
| DALYs | High-middle SDI | 2014 | 43.93486281 | 56.70369317 | 33.50714341 |
| DALYs | High-middle SDI | 2015 | 44.59063773 | 57.53399911 | 33.99950207 |
| DALYs | High-middle SDI | 2016 | 43.71935625 | 56.71070912 | 33.36299335 |
| DALYs | High-middle SDI | 2017 | 42.57657231 | 54.69032259 | 32.46006865 |
| DALYs | High-middle SDI | 2018 | 42.01870307 | 54.19889747 | 31.50591376 |
| DALYs | High-middle SDI | 2019 | 41.66773893 | 53.59908727 | 31.71073229 |
| DALYs | Low SDI | 1990 | 86.64762107 | 117.9089149 | 62.74278695 |
| DALYs | Low SDI | 1991 | 86.59240015 | 117.9393501 | 62.98741185 |
| DALYs | Low SDI | 1992 | 86.53949265 | 116.8966816 | 62.89646502 |
| DALYs | Low SDI | 1993 | 86.52694241 | 117.2283305 | 62.82530169 |
| DALYs | Low SDI | 1994 | 86.48028037 | 117.465349 | 62.98280489 |
| DALYs | Low SDI | 1995 | 86.18703692 | 116.3356592 | 62.57566152 |
| DALYs | Low SDI | 1996 | 85.67534982 | 115.7746857 | 62.72822288 |
| DALYs | Low SDI | 1997 | 84.85840605 | 114.8773616 | 61.91880362 |
| DALYs | Low SDI | 1998 | 83.72680866 | 113.3648332 | 60.57889845 |
| DALYs | Low SDI | 1999 | 82.48627088 | 110.2698025 | 60.09610301 |
| DALYs | Low SDI | 2000 | 81.37597812 | 109.4493925 | 59.14056638 |
| DALYs | Low SDI | 2001 | 80.37787975 | 107.5643619 | 58.85580392 |
| DALYs | Low SDI | 2002 | 79.48569081 | 106.1249974 | 58.12028432 |
| DALYs | Low SDI | 2003 | 78.82471696 | 105.3747896 | 57.4233485 |
| DALYs | Low SDI | 2004 | 77.95571283 | 104.0057644 | 57.04331083 |
| DALYs | Low SDI | 2005 | 77.30345984 | 103.6357537 | 56.69687095 |
| DALYs | Low SDI | 2006 | 75.83703977 | 100.9984522 | 55.48686377 |
| DALYs | Low SDI | 2007 | 74.8359099 | 100.2044665 | 54.81829917 |
| DALYs | Low SDI | 2008 | 73.76657047 | 97.83544275 | 53.97730336 |
| DALYs | Low SDI | 2009 | 72.56888539 | 97.25310049 | 52.99479181 |
| DALYs | Low SDI | 2010 | 71.51623249 | 95.04838707 | 52.33395649 |
| DALYs | Low SDI | 2011 | 70.3775055 | 93.65457106 | 51.71504218 |
| DALYs | Low SDI | 2012 | 69.72384832 | 92.68220736 | 51.61468903 |
| DALYs | Low SDI | 2013 | 69.43172531 | 93.3657883 | 50.72987454 |
| DALYs | Low SDI | 2014 | 68.67389113 | 91.94183207 | 50.11610036 |
| DALYs | Low SDI | 2015 | 68.15402874 | 90.37172161 | 49.60309257 |
| DALYs | Low SDI | 2016 | 68.22803116 | 90.93629287 | 49.60474208 |
| DALYs | Low SDI | 2017 | 69.17314644 | 92.62752863 | 49.99695188 |
| DALYs | Low SDI | 2018 | 68.73358325 | 92.82015605 | 49.80348063 |
| DALYs | Low SDI | 2019 | 68.06351286 | 91.89982437 | 49.41655797 |
| DALYs | Low-middle SDI | 1990 | 73.79505321 | 95.91491907 | 55.05175844 |
| DALYs | Low-middle SDI | 1991 | 73.93474708 | 95.99120905 | 54.89602481 |
| DALYs | Low-middle SDI | 1992 | 74.09436635 | 96.64594165 | 55.40129631 |
| DALYs | Low-middle SDI | 1993 | 74.63358466 | 97.09256679 | 56.00371125 |
| DALYs | Low-middle SDI | 1994 | 74.95720449 | 97.59669039 | 56.18694353 |
| DALYs | Low-middle SDI | 1995 | 74.49095251 | 96.39658288 | 56.2415893 |
| DALYs | Low-middle SDI | 1996 | 73.92187926 | 96.29672105 | 55.95143551 |
| DALYs | Low-middle SDI | 1997 | 73.3712202 | 94.47214707 | 55.08717546 |
| DALYs | Low-middle SDI | 1998 | 71.44826149 | 91.7319151 | 53.81470736 |
| DALYs | Low-middle SDI | 1999 | 68.97553471 | 88.33781031 | 51.9557982 |
| DALYs | Low-middle SDI | 2000 | 67.54429998 | 87.00358802 | 50.69775202 |
| DALYs | Low-middle SDI | 2001 | 66.39031181 | 85.69519372 | 50.07280976 |
| DALYs | Low-middle SDI | 2002 | 65.01567317 | 83.62159594 | 48.61087372 |
| DALYs | Low-middle SDI | 2003 | 63.28949166 | 81.77880893 | 47.39525795 |
| DALYs | Low-middle SDI | 2004 | 61.5908239 | 79.91110433 | 45.80593178 |
| DALYs | Low-middle SDI | 2005 | 61.26899143 | 79.60411391 | 45.80745355 |
| DALYs | Low-middle SDI | 2006 | 61.02369821 | 79.40059897 | 45.44041029 |
| DALYs | Low-middle SDI | 2007 | 60.60885267 | 78.61109789 | 45.4917069 |
| DALYs | Low-middle SDI | 2008 | 60.45238208 | 78.79754274 | 45.20695134 |
| DALYs | Low-middle SDI | 2009 | 59.59389993 | 76.94518792 | 44.63290825 |
| DALYs | Low-middle SDI | 2010 | 59.28446526 | 76.66558129 | 44.70958441 |
| DALYs | Low-middle SDI | 2011 | 59.53679114 | 77.42238662 | 44.64916347 |
| DALYs | Low-middle SDI | 2012 | 59.86770386 | 77.77938532 | 44.86698845 |
| DALYs | Low-middle SDI | 2013 | 60.54345592 | 78.68992005 | 45.30198731 |
| DALYs | Low-middle SDI | 2014 | 60.88205975 | 78.23499868 | 46.0855854 |
| DALYs | Low-middle SDI | 2015 | 61.63704496 | 79.98069593 | 46.37035492 |
| DALYs | Low-middle SDI | 2016 | 62.72263471 | 81.28982577 | 46.70865615 |
| DALYs | Low-middle SDI | 2017 | 64.16539319 | 83.50166171 | 47.66747168 |
| DALYs | Low-middle SDI | 2018 | 63.92374557 | 83.42461737 | 47.55740276 |
| DALYs | Low-middle SDI | 2019 | 63.25244151 | 82.85135649 | 47.34869749 |
| DALYs | Middle SDI | 1990 | 86.10490545 | 107.5732722 | 67.00712451 |
| DALYs | Middle SDI | 1991 | 85.81509288 | 107.2951934 | 67.24908279 |
| DALYs | Middle SDI | 1992 | 85.6881799 | 107.6023406 | 67.21887247 |
| DALYs | Middle SDI | 1993 | 85.66653208 | 109.0326874 | 67.0316598 |
| DALYs | Middle SDI | 1994 | 85.44358819 | 107.6675788 | 66.95916156 |
| DALYs | Middle SDI | 1995 | 84.6930358 | 105.5758725 | 66.44757169 |
| DALYs | Middle SDI | 1996 | 83.74190446 | 104.7672021 | 65.86053586 |
| DALYs | Middle SDI | 1997 | 82.25470589 | 102.4346041 | 65.00442978 |
| DALYs | Middle SDI | 1998 | 80.87666329 | 100.4509466 | 63.68312147 |
| DALYs | Middle SDI | 1999 | 79.21356565 | 98.12698053 | 62.89683611 |
| DALYs | Middle SDI | 2000 | 77.07300294 | 96.29785522 | 60.78855663 |
| DALYs | Middle SDI | 2001 | 74.64721904 | 93.30269274 | 58.7380476 |
| DALYs | Middle SDI | 2002 | 72.86714178 | 91.0732045 | 56.90507238 |
| DALYs | Middle SDI | 2003 | 71.2922516 | 89.53814996 | 55.54895481 |
| DALYs | Middle SDI | 2004 | 69.66059211 | 87.36879468 | 53.96935514 |
| DALYs | Middle SDI | 2005 | 68.61189919 | 86.22840967 | 52.97730045 |
| DALYs | Middle SDI | 2006 | 67.45870545 | 84.73032785 | 52.17928963 |
| DALYs | Middle SDI | 2007 | 66.74452219 | 84.23097971 | 51.39788025 |
| DALYs | Middle SDI | 2008 | 67.18014585 | 84.70675577 | 52.22514032 |
| DALYs | Middle SDI | 2009 | 67.25804839 | 84.05123711 | 52.20472723 |
| DALYs | Middle SDI | 2010 | 66.59053893 | 83.77327605 | 51.59351933 |
| DALYs | Middle SDI | 2011 | 65.88095472 | 82.69335512 | 51.11707792 |
| DALYs | Middle SDI | 2012 | 65.62399194 | 81.88652332 | 51.40164643 |
| DALYs | Middle SDI | 2013 | 65.71904461 | 81.85581436 | 51.08421233 |
| DALYs | Middle SDI | 2014 | 65.6912305 | 82.14184519 | 51.11971194 |
| DALYs | Middle SDI | 2015 | 65.45537969 | 80.74265071 | 51.26650514 |
| DALYs | Middle SDI | 2016 | 65.58672271 | 82.70158852 | 51.17946719 |
| DALYs | Middle SDI | 2017 | 65.79941684 | 82.26105615 | 51.00429481 |
| DALYs | Middle SDI | 2018 | 65.53123678 | 82.02891711 | 50.71973135 |
| DALYs | Middle SDI | 2019 | 65.3989016 | 82.14015122 | 50.65947861 |
| Deaths | Global | 1990 | 2.394932462 | 3.050516968 | 1.839684075 |
| Deaths | Global | 1991 | 2.401078877 | 3.036186737 | 1.847968356 |
| Deaths | Global | 1992 | 2.408936818 | 3.053527367 | 1.860573535 |
| Deaths | Global | 1993 | 2.434035325 | 3.075028144 | 1.88539382 |
| Deaths | Global | 1994 | 2.445671579 | 3.078471588 | 1.885082119 |
| Deaths | Global | 1995 | 2.441083947 | 3.082075902 | 1.894581314 |
| Deaths | Global | 1996 | 2.422579008 | 3.043328285 | 1.876488995 |
| Deaths | Global | 1997 | 2.397044713 | 3.005959548 | 1.857757255 |
| Deaths | Global | 1998 | 2.371227674 | 2.965732267 | 1.854978247 |
| Deaths | Global | 1999 | 2.345522099 | 2.93051409 | 1.839040504 |
| Deaths | Global | 2000 | 2.313005963 | 2.88768694 | 1.791408097 |
| Deaths | Global | 2001 | 2.276110633 | 2.85383574 | 1.758494759 |
| Deaths | Global | 2002 | 2.250023199 | 2.825531032 | 1.743642329 |
| Deaths | Global | 2003 | 2.224990198 | 2.811776003 | 1.716337659 |
| Deaths | Global | 2004 | 2.185592447 | 2.755834432 | 1.685690257 |
| Deaths | Global | 2005 | 2.17438608 | 2.741718793 | 1.663056051 |
| Deaths | Global | 2006 | 2.14161623 | 2.689165174 | 1.653753273 |
| Deaths | Global | 2007 | 2.123831227 | 2.66293538 | 1.630521776 |
| Deaths | Global | 2008 | 2.127440054 | 2.675255567 | 1.632349709 |
| Deaths | Global | 2009 | 2.107425763 | 2.646997418 | 1.627618117 |
| Deaths | Global | 2010 | 2.088179105 | 2.624011334 | 1.613397482 |
| Deaths | Global | 2011 | 2.072012071 | 2.599078636 | 1.596380689 |
| Deaths | Global | 2012 | 2.072016905 | 2.59826921 | 1.61591868 |
| Deaths | Global | 2013 | 2.082699635 | 2.609568773 | 1.603818531 |
| Deaths | Global | 2014 | 2.0913015 | 2.627903495 | 1.612916706 |
| Deaths | Global | 2015 | 2.101607283 | 2.640942179 | 1.620884052 |
| Deaths | Global | 2016 | 2.102886684 | 2.642039818 | 1.621802725 |
| Deaths | Global | 2017 | 2.103031339 | 2.638750882 | 1.610200798 |
| Deaths | Global | 2018 | 2.093932275 | 2.634348239 | 1.610761295 |
| Deaths | Global | 2019 | 2.087758117 | 2.599749087 | 1.613922804 |
| Deaths | High SDI | 1990 | 1.414200914 | 1.817302718 | 1.067528302 |
| Deaths | High SDI | 1991 | 1.419965649 | 1.820721673 | 1.077851466 |
| Deaths | High SDI | 1992 | 1.422720625 | 1.820812246 | 1.08189324 |
| Deaths | High SDI | 1993 | 1.440649134 | 1.833028995 | 1.099259491 |
| Deaths | High SDI | 1994 | 1.447195276 | 1.838535859 | 1.106509442 |
| Deaths | High SDI | 1995 | 1.461584378 | 1.851432222 | 1.120340122 |
| Deaths | High SDI | 1996 | 1.459131439 | 1.84225525 | 1.123788886 |
| Deaths | High SDI | 1997 | 1.45252729 | 1.825318882 | 1.121298686 |
| Deaths | High SDI | 1998 | 1.460588739 | 1.828302896 | 1.136451981 |
| Deaths | High SDI | 1999 | 1.465385375 | 1.826513265 | 1.148012904 |
| Deaths | High SDI | 2000 | 1.462773662 | 1.816552022 | 1.145671699 |
| Deaths | High SDI | 2001 | 1.460297121 | 1.806573881 | 1.141502071 |
| Deaths | High SDI | 2002 | 1.456734911 | 1.80096293 | 1.140043314 |
| Deaths | High SDI | 2003 | 1.454841603 | 1.796426132 | 1.136311482 |
| Deaths | High SDI | 2004 | 1.427942015 | 1.761730376 | 1.115869724 |
| Deaths | High SDI | 2005 | 1.420130193 | 1.751054966 | 1.110169888 |
| Deaths | High SDI | 2006 | 1.403274373 | 1.730846628 | 1.098250834 |
| Deaths | High SDI | 2007 | 1.390104855 | 1.714678103 | 1.087930409 |
| Deaths | High SDI | 2008 | 1.380215615 | 1.702355938 | 1.082692977 |
| Deaths | High SDI | 2009 | 1.36566026 | 1.689184607 | 1.071004014 |
| Deaths | High SDI | 2010 | 1.349944623 | 1.668450503 | 1.056059999 |
| Deaths | High SDI | 2011 | 1.353248598 | 1.677377281 | 1.059897113 |
| Deaths | High SDI | 2012 | 1.361492062 | 1.686602384 | 1.066231861 |
| Deaths | High SDI | 2013 | 1.376647809 | 1.703696078 | 1.07456592 |
| Deaths | High SDI | 2014 | 1.387429709 | 1.727371134 | 1.081607939 |
| Deaths | High SDI | 2015 | 1.400700135 | 1.750815532 | 1.090943748 |
| Deaths | High SDI | 2016 | 1.397059319 | 1.747766374 | 1.089451506 |
| Deaths | High SDI | 2017 | 1.379299744 | 1.72197825 | 1.075171006 |
| Deaths | High SDI | 2018 | 1.374884378 | 1.72095915 | 1.071815717 |
| Deaths | High SDI | 2019 | 1.371451108 | 1.719749453 | 1.068380086 |
| Deaths | High-middle SDI | 1990 | 2.023044068 | 2.531558669 | 1.563799913 |
| Deaths | High-middle SDI | 1991 | 2.031498371 | 2.546914144 | 1.593641128 |
| Deaths | High-middle SDI | 1992 | 2.041569541 | 2.574818887 | 1.587130765 |
| Deaths | High-middle SDI | 1993 | 2.086337915 | 2.617727072 | 1.619178884 |
| Deaths | High-middle SDI | 1994 | 2.113184997 | 2.664783022 | 1.64281257 |
| Deaths | High-middle SDI | 1995 | 2.10321059 | 2.640383109 | 1.644530206 |
| Deaths | High-middle SDI | 1996 | 2.062502892 | 2.571564592 | 1.619795554 |
| Deaths | High-middle SDI | 1997 | 2.020789206 | 2.507038964 | 1.581189322 |
| Deaths | High-middle SDI | 1998 | 1.988662104 | 2.474536312 | 1.569118756 |
| Deaths | High-middle SDI | 1999 | 1.98794231 | 2.462082266 | 1.558632925 |
| Deaths | High-middle SDI | 2000 | 1.968563787 | 2.447275288 | 1.534157395 |
| Deaths | High-middle SDI | 2001 | 1.935357291 | 2.432817142 | 1.504304459 |
| Deaths | High-middle SDI | 2002 | 1.916031466 | 2.41411169 | 1.491635433 |
| Deaths | High-middle SDI | 2003 | 1.900460075 | 2.417056714 | 1.460381396 |
| Deaths | High-middle SDI | 2004 | 1.869006485 | 2.381857181 | 1.435861215 |
| Deaths | High-middle SDI | 2005 | 1.875665731 | 2.388889063 | 1.433555483 |
| Deaths | High-middle SDI | 2006 | 1.818547146 | 2.316233268 | 1.400154527 |
| Deaths | High-middle SDI | 2007 | 1.798543054 | 2.270851929 | 1.380916722 |
| Deaths | High-middle SDI | 2008 | 1.796925599 | 2.278940691 | 1.394295293 |
| Deaths | High-middle SDI | 2009 | 1.74089805 | 2.203177652 | 1.347164835 |
| Deaths | High-middle SDI | 2010 | 1.709813417 | 2.161644105 | 1.320255354 |
| Deaths | High-middle SDI | 2011 | 1.661155044 | 2.103086086 | 1.275652224 |
| Deaths | High-middle SDI | 2012 | 1.644074703 | 2.070103074 | 1.268084657 |
| Deaths | High-middle SDI | 2013 | 1.6361955 | 2.057895793 | 1.263271788 |
| Deaths | High-middle SDI | 2014 | 1.642533522 | 2.060281354 | 1.258312535 |
| Deaths | High-middle SDI | 2015 | 1.660658431 | 2.088929895 | 1.2908554 |
| Deaths | High-middle SDI | 2016 | 1.635740899 | 2.058368656 | 1.26816866 |
| Deaths | High-middle SDI | 2017 | 1.601346435 | 2.008747774 | 1.227288502 |
| Deaths | High-middle SDI | 2018 | 1.581948069 | 1.979527355 | 1.223983979 |
| Deaths | High-middle SDI | 2019 | 1.570774764 | 1.968414847 | 1.216592552 |
| Deaths | Low SDI | 1990 | 3.470056283 | 4.666381037 | 2.487829285 |
| Deaths | Low SDI | 1991 | 3.467199362 | 4.644531139 | 2.506337576 |
| Deaths | Low SDI | 1992 | 3.466818645 | 4.622198736 | 2.493526017 |
| Deaths | Low SDI | 1993 | 3.468083762 | 4.664978984 | 2.522990261 |
| Deaths | Low SDI | 1994 | 3.4634795 | 4.631877213 | 2.502394033 |
| Deaths | Low SDI | 1995 | 3.455024591 | 4.602706212 | 2.495988179 |
| Deaths | Low SDI | 1996 | 3.438381389 | 4.561432786 | 2.493176348 |
| Deaths | Low SDI | 1997 | 3.416767608 | 4.560609649 | 2.508502536 |
| Deaths | Low SDI | 1998 | 3.384554531 | 4.507669633 | 2.439444131 |
| Deaths | Low SDI | 1999 | 3.345576663 | 4.447516847 | 2.430348982 |
| Deaths | Low SDI | 2000 | 3.315835104 | 4.409239385 | 2.409617163 |
| Deaths | Low SDI | 2001 | 3.282024797 | 4.330964948 | 2.393058821 |
| Deaths | Low SDI | 2002 | 3.258064693 | 4.288755887 | 2.376243993 |
| Deaths | Low SDI | 2003 | 3.233590449 | 4.268955416 | 2.368743978 |
| Deaths | Low SDI | 2004 | 3.200908134 | 4.232896788 | 2.325661715 |
| Deaths | Low SDI | 2005 | 3.179887989 | 4.225685896 | 2.310539508 |
| Deaths | Low SDI | 2006 | 3.126138511 | 4.123228576 | 2.286540842 |
| Deaths | Low SDI | 2007 | 3.080902705 | 4.06004813 | 2.258604234 |
| Deaths | Low SDI | 2008 | 3.042343725 | 4.016135744 | 2.222013277 |
| Deaths | Low SDI | 2009 | 2.991470795 | 3.96493209 | 2.178648737 |
| Deaths | Low SDI | 2010 | 2.945755472 | 3.904878515 | 2.152561363 |
| Deaths | Low SDI | 2011 | 2.898339233 | 3.826916788 | 2.119567659 |
| Deaths | Low SDI | 2012 | 2.867171521 | 3.786007234 | 2.108522836 |
| Deaths | Low SDI | 2013 | 2.853751522 | 3.772303979 | 2.102010703 |
| Deaths | Low SDI | 2014 | 2.816664617 | 3.747769749 | 2.071551375 |
| Deaths | Low SDI | 2015 | 2.792791634 | 3.709526733 | 2.058088583 |
| Deaths | Low SDI | 2016 | 2.79065092 | 3.711833311 | 2.043746566 |
| Deaths | Low SDI | 2017 | 2.82542251 | 3.746112759 | 2.056925267 |
| Deaths | Low SDI | 2018 | 2.809936886 | 3.725325287 | 2.06949986 |
| Deaths | Low SDI | 2019 | 2.786022651 | 3.736560675 | 2.045174153 |
| Deaths | Low-middle SDI | 1990 | 2.792101263 | 3.625272279 | 2.086876142 |
| Deaths | Low-middle SDI | 1991 | 2.79990461 | 3.62204683 | 2.092284216 |
| Deaths | Low-middle SDI | 1992 | 2.808351195 | 3.619380236 | 2.108684361 |
| Deaths | Low-middle SDI | 1993 | 2.832032442 | 3.673555595 | 2.139175416 |
| Deaths | Low-middle SDI | 1994 | 2.841592659 | 3.648411375 | 2.129455357 |
| Deaths | Low-middle SDI | 1995 | 2.827566002 | 3.633593694 | 2.13025723 |
| Deaths | Low-middle SDI | 1996 | 2.812260845 | 3.602167417 | 2.114480371 |
| Deaths | Low-middle SDI | 1997 | 2.803494923 | 3.573275575 | 2.118985118 |
| Deaths | Low-middle SDI | 1998 | 2.750679523 | 3.537022825 | 2.084713494 |
| Deaths | Low-middle SDI | 1999 | 2.673828744 | 3.417861827 | 2.012461579 |
| Deaths | Low-middle SDI | 2000 | 2.634089292 | 3.357431356 | 1.979956444 |
| Deaths | Low-middle SDI | 2001 | 2.602876616 | 3.33313259 | 1.954541533 |
| Deaths | Low-middle SDI | 2002 | 2.56798468 | 3.292055575 | 1.924977806 |
| Deaths | Low-middle SDI | 2003 | 2.513813878 | 3.228742205 | 1.885645342 |
| Deaths | Low-middle SDI | 2004 | 2.452027211 | 3.145040522 | 1.840079854 |
| Deaths | Low-middle SDI | 2005 | 2.445749943 | 3.130920143 | 1.825284854 |
| Deaths | Low-middle SDI | 2006 | 2.438160806 | 3.13205776 | 1.820704177 |
| Deaths | Low-middle SDI | 2007 | 2.41920684 | 3.100674085 | 1.81254234 |
| Deaths | Low-middle SDI | 2008 | 2.413063002 | 3.087298806 | 1.813913384 |
| Deaths | Low-middle SDI | 2009 | 2.37257033 | 3.059259382 | 1.776533479 |
| Deaths | Low-middle SDI | 2010 | 2.349009984 | 3.01971505 | 1.764491671 |
| Deaths | Low-middle SDI | 2011 | 2.346950226 | 3.019488983 | 1.762843056 |
| Deaths | Low-middle SDI | 2012 | 2.351405047 | 3.034858779 | 1.77096167 |
| Deaths | Low-middle SDI | 2013 | 2.382980332 | 3.074371044 | 1.78427159 |
| Deaths | Low-middle SDI | 2014 | 2.399977557 | 3.074899273 | 1.822849636 |
| Deaths | Low-middle SDI | 2015 | 2.423289616 | 3.095264236 | 1.842193551 |
| Deaths | Low-middle SDI | 2016 | 2.451624621 | 3.137666042 | 1.853110038 |
| Deaths | Low-middle SDI | 2017 | 2.496044041 | 3.20756998 | 1.895023593 |
| Deaths | Low-middle SDI | 2018 | 2.488270402 | 3.179721549 | 1.865817457 |
| Deaths | Low-middle SDI | 2019 | 2.468388237 | 3.162030939 | 1.879878459 |
| Deaths | Middle SDI | 1990 | 3.513650721 | 4.423300911 | 2.733207313 |
| Deaths | Middle SDI | 1991 | 3.503118443 | 4.397215877 | 2.73999671 |
| Deaths | Middle SDI | 1992 | 3.499865129 | 4.451961144 | 2.723302849 |
| Deaths | Middle SDI | 1993 | 3.50334063 | 4.406603506 | 2.727607932 |
| Deaths | Middle SDI | 1994 | 3.493270012 | 4.384585102 | 2.727299615 |
| Deaths | Middle SDI | 1995 | 3.467019434 | 4.341975548 | 2.720344041 |
| Deaths | Middle SDI | 1996 | 3.437191768 | 4.281613205 | 2.698907831 |
| Deaths | Middle SDI | 1997 | 3.385278343 | 4.242579983 | 2.632726307 |
| Deaths | Middle SDI | 1998 | 3.337800013 | 4.158995321 | 2.641783524 |
| Deaths | Middle SDI | 1999 | 3.279577256 | 4.047802554 | 2.581721887 |
| Deaths | Middle SDI | 2000 | 3.205136179 | 3.99323332 | 2.536918345 |
| Deaths | Middle SDI | 2001 | 3.124562849 | 3.906792689 | 2.436296337 |
| Deaths | Middle SDI | 2002 | 3.074811813 | 3.848053275 | 2.409523439 |
| Deaths | Middle SDI | 2003 | 3.034206296 | 3.817579568 | 2.349584097 |
| Deaths | Middle SDI | 2004 | 2.979913371 | 3.759251367 | 2.316774266 |
| Deaths | Middle SDI | 2005 | 2.939571411 | 3.730349165 | 2.256769753 |
| Deaths | Middle SDI | 2006 | 2.89036905 | 3.649991279 | 2.240641575 |
| Deaths | Middle SDI | 2007 | 2.8627233 | 3.606153881 | 2.21569264 |
| Deaths | Middle SDI | 2008 | 2.887275598 | 3.64034538 | 2.238877646 |
| Deaths | Middle SDI | 2009 | 2.898710553 | 3.629060381 | 2.258215139 |
| Deaths | Middle SDI | 2010 | 2.880358548 | 3.585380883 | 2.235035367 |
| Deaths | Middle SDI | 2011 | 2.855457148 | 3.559277375 | 2.219593834 |
| Deaths | Middle SDI | 2012 | 2.85149856 | 3.552783149 | 2.211822795 |
| Deaths | Middle SDI | 2013 | 2.858141647 | 3.556660583 | 2.228495059 |
| Deaths | Middle SDI | 2014 | 2.859929866 | 3.567219518 | 2.232648033 |
| Deaths | Middle SDI | 2015 | 2.844518528 | 3.528027348 | 2.221601895 |
| Deaths | Middle SDI | 2016 | 2.836686428 | 3.551520282 | 2.196698719 |
| Deaths | Middle SDI | 2017 | 2.831145035 | 3.560694409 | 2.189361907 |
| Deaths | Middle SDI | 2018 | 2.812763839 | 3.539283809 | 2.168509414 |
| Deaths | Middle SDI | 2019 | 2.80453692 | 3.516724457 | 2.173498745 |

Supplementary Table 7. Global age-specific prevalence, incidence, DALYs, and Death percentage of annual rate of change from 1990 to 2019.

| **Measure** | **Age** | **Percent** | **95% Upper UI** | **95% Lowe UI** |
| --- | --- | --- | --- | --- |
| Prevalence | 1-4 | 0 | 0 | 0 |
| Prevalence | 5-9 | 0 | 0 | 0 |
| Prevalence | 10-14 | 0 | 0 | 0 |
| Prevalence | 15-19 | 0.278181479 | 0.310164573 | 0.249992889 |
| Prevalence | 20-24 | 0.278056168 | 0.306230734 | 0.246746437 |
| Prevalence | 25-29 | 0.274616741 | 0.304342728 | 0.245508233 |
| Prevalence | 30-34 | 0.285804645 | 0.318732117 | 0.255615458 |
| Prevalence | 35-39 | 0.2900019 | 0.327589314 | 0.255166511 |
| Prevalence | 40-44 | 0.294707413 | 0.338258118 | 0.256248121 |
| Prevalence | 45-49 | 0.256080054 | 0.300750883 | 0.216451589 |
| Prevalence | 50-54 | 0.230015366 | 0.27247873 | 0.182302763 |
| Prevalence | 55-59 | 0.207656196 | 0.250946836 | 0.156650719 |
| Prevalence | 60-64 | 0.208409056 | 0.254348352 | 0.150620263 |
| Prevalence | 65-69 | 0.207942336 | 0.254565705 | 0.150054191 |
| Prevalence | 70-74 | 0.17878803 | 0.226167183 | 0.123066983 |
| Prevalence | 75-79 | 0.202281086 | 0.244030849 | 0.151332506 |
| Prevalence | 80-84 | 0.214173616 | 0.255100106 | 0.162606984 |
| Prevalence | 85-89 | 0.218045945 | 0.251852103 | 0.179206726 |
| Prevalence | 90-94 | 0.210973017 | 0.24366831 | 0.179362176 |
| Prevalence | 95+ | 0.187703485 | 0.221643396 | 0.148467543 |
| Incidence | 1-4 | 0 | 0 | 0 |
| Incidence | 5-9 | 0 | 0 | 0 |
| Incidence | 10-14 | 0 | 0 | 0 |
| Incidence | 15-19 | 0.050289564 | 0.143331246 | -0.037770367 |
| Incidence | 20-24 | 0.027329311 | 0.116279006 | -0.041613664 |
| Incidence | 25-29 | 0.013166729 | 0.086809545 | -0.055187941 |
| Incidence | 30-34 | -0.021081964 | 0.039018942 | -0.082190142 |
| Incidence | 35-39 | -0.054216832 | 0.01092116 | -0.116220413 |
| Incidence | 40-44 | -0.004915274 | 0.093398411 | -0.068337836 |
| Incidence | 45-49 | 0.073560343 | 0.200103018 | -0.003386828 |
| Incidence | 50-54 | 0.175036957 | 0.382330341 | 0.065156488 |
| Incidence | 55-59 | 0.247277584 | 0.534564537 | 0.076740925 |
| Incidence | 60-64 | 0.228140791 | 0.438799453 | 0.048614569 |
| Incidence | 65-69 | 0.046222407 | 0.203677573 | -0.1026426 |
| Incidence | 70-74 | 0.00988983 | 0.168599434 | -0.115322276 |
| Incidence | 75-79 | 0.132686595 | 0.299057048 | 0.000770325 |
| Incidence | 80-84 | 0.338693461 | 0.538841771 | 0.137038118 |
| Incidence | 85-89 | 0.459287919 | 0.710008904 | 0.121776928 |
| Incidence | 90-94 | 0.392717135 | 0.768826281 | -0.080882136 |
| Incidence | 95+ | 0.123521394 | 0.956839459 | -0.25773657 |
| DALYs | 1-4 | 0.094505286 | 0.287963233 | -0.06004469 |
| DALYs | 5-9 | 0.105143983 | 0.332461121 | -0.050615683 |
| DALYs | 10-14 | 0.098707755 | 0.267436369 | -0.028918805 |
| DALYs | 15-19 | 0.0468001 | 0.167302299 | -0.044131243 |
| DALYs | 20-24 | 0.010665983 | 0.107775245 | -0.073725061 |
| DALYs | 25-29 | -0.009227839 | 0.072099544 | -0.085610621 |
| DALYs | 30-34 | -0.013701309 | 0.06808913 | -0.079637516 |
| DALYs | 35-39 | 0.026055543 | 0.108976717 | -0.044243123 |
| DALYs | 40-44 | 0.074480139 | 0.162145663 | 0.002450393 |
| DALYs | 45-49 | 0.135308116 | 0.237023819 | 0.060804623 |
| DALYs | 50-54 | 0.113372468 | 0.211854378 | 0.040322079 |
| DALYs | 55-59 | 0.161507397 | 0.272054464 | 0.072396702 |
| DALYs | 60-64 | 0.240653268 | 0.363845367 | 0.136449783 |
| DALYs | 65-69 | 0.24866054 | 0.392448714 | 0.106615355 |
| DALYs | 70-74 | 0.207180303 | 0.346284207 | 0.06917242 |
| DALYs | 75-79 | 0.156213139 | 0.311301638 | 0.008409342 |
| DALYs | 80-84 | 0.1636668 | 0.340102635 | 0.014043789 |
| DALYs | 85-89 | 0.094505286 | 0.287963233 | -0.06004469 |
| DALYs | 90-94 | 0.105143983 | 0.332461121 | -0.050615683 |
| DALYs | 95+ | 0.098707755 | 0.267436369 | -0.028918805 |
| Deaths | 1-4 | 0.361651076 | 0.596261951 | 0.174890572 |
| Deaths | 5-9 | 0.298163928 | 0.571971907 | 0.124977446 |
| Deaths | 10-14 | 0.291430155 | 0.493982793 | 0.148698951 |
| Deaths | 15-19 | 0.210011603 | 0.332202324 | 0.1135289 |
| Deaths | 20-24 | 0.152354634 | 0.253223695 | 0.062553812 |
| Deaths | 25-29 | 0.1273414 | 0.215004894 | 0.054168142 |
| Deaths | 30-34 | 0.134168757 | 0.221234997 | 0.067413235 |
| Deaths | 35-39 | 0.175233099 | 0.258425139 | 0.105421325 |
| Deaths | 40-44 | 0.214455749 | 0.305261223 | 0.14049028 |
| Deaths | 45-49 | 0.270822702 | 0.381676152 | 0.195862876 |
| Deaths | 50-54 | 0.23574337 | 0.340886978 | 0.161131866 |
| Deaths | 55-59 | 0.278973054 | 0.394630818 | 0.186566656 |
| Deaths | 60-64 | 0.350961983 | 0.478915449 | 0.238523583 |
| Deaths | 65-69 | 0.328962219 | 0.472428921 | 0.174850242 |
| Deaths | 70-74 | 0.269225154 | 0.41684231 | 0.122483939 |
| Deaths | 75-79 | 0.204257539 | 0.362952342 | 0.050780577 |
| Deaths | 80-84 | 0.199938809 | 0.383278532 | 0.04546037 |
| Deaths | 85-89 | 0.361651076 | 0.596261951 | 0.174890572 |
| Deaths | 90-94 | 0.298163928 | 0.571971907 | 0.124977446 |
| Deaths | 95+ | 0.291430155 | 0.493982793 | 0.148698951 |
